# Supplementary material for: De novo genome assembly of Geosmithia morbida, the causal agent of thousand cankers disease
Source: PeerJ. 2016 May 2;4:e1952. doi: 10.7717/peerj.1952 (PMC4860301; doi:10.7717/peerj.1952)
Supplement: Supplemental Information 7 [file peerj-04-1952-s007.rtf]

#                                                                                   --- full sequence --- -------------- this domain -------------   hmm coord   ali coord   env coord# target name        accession   tlen query name                  accession   qlen   E-value  score  bias   #  of  c-Evalue  i-Evalue  score  bias  from    to  from    to  from    to  acc description of target#------------------- ---------- -----        -------------------- ---------- ----- --------- ------ ----- --- --- --------- --------- ------ ----- ----- ----- ----- ----- ----- ----- ---- ---------------------CBM_14               PF01607.20    53 gi|1563717|emb|CAA69643.1| -            135   2.1e-07   30.8   6.3   1   1   2.3e-11   3.7e-07   30.1   6.3     1    53    50   109    50   109 0.90 Chitin binding Peritrophin-A domainHce2                 PF14856.2    102 gi|2561|emb|CAA78401.1| -            165   1.7e-15   57.0   0.2   1   1   3.4e-19   2.8e-15   56.3   0.2     2   102    34   139    33   139 0.82 Pathogen effector; putative necrosis-inducing factorPapC_C               PF13953.2     65 gi|2561|emb|CAA78401.1| -            165      0.06   13.1   0.2   1   1   1.5e-05      0.12   12.1   0.2    17    48   121   152   101   158 0.85 PapC C-terminal domainLPAM_1               PF08139.8     35 gi|90264934|emb|CAJ29326.1| -            205       3.2    8.2   4.7   1   3      0.11   1.9e+03   -0.5   0.5    21    31     7    17     3    18 0.73 Prokaryotic membrane lipoprotein lipid attachment siteLPAM_1               PF08139.8     35 gi|90264934|emb|CAJ29326.1| -            205       3.2    8.2   4.7   2   3    0.0001       1.7    9.1   0.1     2    24    64    86    64    86 0.93 Prokaryotic membrane lipoprotein lipid attachment siteLPAM_1               PF08139.8     35 gi|90264934|emb|CAJ29326.1| -            205       3.2    8.2   4.7   3   3      0.72   1.2e+04   -3.1   0.0    15    23   191   199   191   202 0.78 Prokaryotic membrane lipoprotein lipid attachment siteDAP_epimerase        PF01678.15   121 gi|38520867|emb|CAE55866.1| -            284      0.14   12.1   0.2   1   1   1.6e-05      0.26   11.3   0.2    22   106   111   198   103   204 0.72 Diaminopimelate epimerasePeptidase_M35        PF02102.11   359 tr|C1KJI7|C1KJI7_MAGOR -            224   1.4e-12   47.0   0.0   1   1   1.1e-16   1.8e-12   46.7   0.0   177   347    50   218    36   222 0.85 Deuterolysin metalloprotease (M35) familyketoacyl-synt        PF00109.22   253 gi|47109414|emb|CAG28797.1| -           4034   4.6e-77  258.9   0.0   1   1   1.4e-79   9.2e-77  257.9   0.0     2   252    11   260    10   261 0.96 Beta-ketoacyl synthase, N-terminal domainAMP-binding          PF00501.24   421 gi|47109414|emb|CAG28797.1| -           4034   2.3e-69  233.9   0.0   1   1     7e-72   4.7e-69  232.8   0.0     7   415  3073  3473  3067  3481 0.82 AMP-binding enzymeKR                   PF08659.6    180 gi|47109414|emb|CAG28797.1| -           4034   3.2e-52  176.9   0.0   1   2   1.1e-52   7.6e-50  169.2   0.0     2   178  2144  2317  2143  2319 0.97 KR domainKR                   PF08659.6    180 gi|47109414|emb|CAG28797.1| -           4034   3.2e-52  176.9   0.0   2   2     0.029        19    4.9   0.0     3    36  3717  3751  3716  3774 0.81 KR domainCondensation         PF00668.16   301 gi|47109414|emb|CAG28797.1| -           4034   1.1e-51  175.7   0.0   1   1   2.4e-54   1.6e-51  175.0   0.0     4   300  2608  2899  2606  2900 0.96 Condensation domainPS-DH                PF14765.2    298 gi|47109414|emb|CAG28797.1| -           4034   3.5e-50  170.8   0.0   1   1     1e-52     7e-50  169.8   0.0     2   292   952  1252   951  1257 0.87 Polyketide synthase dehydrataseNAD_binding_4        PF07993.8    257 gi|47109414|emb|CAG28797.1| -           4034   3.3e-42  144.3   0.0   1   1   3.2e-44   2.2e-41  141.6   0.0     1   255  3719  3944  3719  3946 0.89 Male sterility proteinAcyl_transf_1        PF00698.17   319 gi|47109414|emb|CAG28797.1| -           4034   3.2e-41  141.7   0.0   1   1   1.1e-43   7.3e-41  140.6   0.0     3   301   560   882   559   899 0.85 Acyl transferase domainKetoacyl-synt_C      PF02801.18   118 gi|47109414|emb|CAG28797.1| -           4034   8.9e-30  103.0   0.0   1   1   2.8e-32   1.9e-29  101.9   0.0     3   117   271   396   269   397 0.91 Beta-ketoacyl synthase, C-terminal domainMethyltransf_12      PF08242.8     99 gi|47109414|emb|CAG28797.1| -           4034   2.1e-16   60.2   0.0   1   1     1e-18   6.9e-16   58.6   0.0     1    99  1432  1533  1432  1533 0.86 Methyltransferase domainEpimerase            PF01370.17   241 gi|47109414|emb|CAG28797.1| -           4034   2.5e-15   56.5   0.0   1   2      0.96   6.5e+02   -0.6   0.0     4    61  2148  2212  2146  2230 0.87 NAD dependent epimerase/dehydratase familyEpimerase            PF01370.17   241 gi|47109414|emb|CAG28797.1| -           4034   2.5e-15   56.5   0.0   2   2   2.5e-17   1.7e-14   53.7   0.0     1   226  3717  3950  3717  3966 0.79 NAD dependent epimerase/dehydratase familyPP-binding           PF00550.21    67 gi|47109414|emb|CAG28797.1| -           4034   2.6e-14   53.2   0.6   1   2   3.6e-07   0.00024   21.2   0.8    11    67  2445  2501  2435  2501 0.85 Phosphopantetheine attachment sitePP-binding           PF00550.21    67 gi|47109414|emb|CAG28797.1| -           4034   2.6e-14   53.2   0.6   2   2   1.6e-09   1.1e-06   28.8   0.0     6    67  3610  3674  3607  3674 0.92 Phosphopantetheine attachment siteMethyltransf_31      PF13847.2    152 gi|47109414|emb|CAG28797.1| -           4034   4.4e-14   52.4   0.0   1   2   2.3e-16   1.5e-13   50.7   0.0     4   114  1428  1541  1426  1552 0.92 Methyltransferase domainMethyltransf_31      PF13847.2    152 gi|47109414|emb|CAG28797.1| -           4034   4.4e-14   52.4   0.0   2   2       8.6   5.8e+03   -3.2   0.0    87   113  3258  3290  3241  3335 0.69 Methyltransferase domainMethyltransf_11      PF08241.8     95 gi|47109414|emb|CAG28797.1| -           4034   2.2e-13   50.5   0.1   1   1   1.2e-15   7.9e-13   48.7   0.1     1    95  1432  1535  1432  1535 0.85 Methyltransferase domainadh_short            PF00106.21   195 gi|47109414|emb|CAG28797.1| -           4034   9.5e-13   47.9   2.7   1   4       8.7   5.9e+03   -3.6   0.2   101   151   616   664   610   665 0.74 short chain dehydrogenaseadh_short            PF00106.21   195 gi|47109414|emb|CAG28797.1| -           4034   9.5e-13   47.9   2.7   2   4       5.6   3.8e+03   -3.0   0.0    62    91  1413  1442  1411  1465 0.77 short chain dehydrogenaseadh_short            PF00106.21   195 gi|47109414|emb|CAG28797.1| -           4034   9.5e-13   47.9   2.7   3   4   2.4e-15   1.6e-12   47.2   0.1     5   182  2147  2317  2143  2328 0.92 short chain dehydrogenaseadh_short            PF00106.21   195 gi|47109414|emb|CAG28797.1| -           4034   9.5e-13   47.9   2.7   4   4       6.4   4.3e+03   -3.2   0.0   145   165  3866  3886  3862  3889 0.80 short chain dehydrogenaseHxxPF_rpt            PF13745.2     91 gi|47109414|emb|CAG28797.1| -           4034   2.8e-11   43.7   0.0   1   1   1.3e-13   8.6e-11   42.1   0.0     1    87  2921  3008  2921  3012 0.95 HxxPF-repeated domainKAsynt_C_assoc       PF16197.1    113 gi|47109414|emb|CAG28797.1| -           4034   8.7e-11   41.8   0.0   1   1   4.1e-13   2.8e-10   40.2   0.0    11   100   408   508   400   518 0.78 Ketoacyl-synthetase C-terminal extensionMethyltransf_23      PF13489.2    165 gi|47109414|emb|CAG28797.1| -           4034   1.1e-10   41.5   0.0   1   1   4.2e-13   2.9e-10   40.2   0.0    21   160  1426  1584  1407  1588 0.66 Methyltransferase domainMethyltransf_18      PF12847.3    110 gi|47109414|emb|CAG28797.1| -           4034   6.1e-08   33.4   0.0   1   2   8.1e-10   5.5e-07   30.3   0.0     4   107  1430  1535  1427  1538 0.80 Methyltransferase domainMethyltransf_18      PF12847.3    110 gi|47109414|emb|CAG28797.1| -           4034   6.1e-08   33.4   0.0   2   2       6.7   4.5e+03   -1.6   0.0     5    34  2013  2043  2010  2062 0.79 Methyltransferase domain3Beta_HSD            PF01073.15   282 gi|47109414|emb|CAG28797.1| -           4034   8.9e-07   28.0   0.0   1   2      0.18   1.2e+02    1.3   0.0     5    79  2150  2225  2147  2290 0.75 3-beta hydroxysteroid dehydrogenase/isomerase family3Beta_HSD            PF01073.15   282 gi|47109414|emb|CAG28797.1| -           4034   8.9e-07   28.0   0.0   2   2   2.8e-08   1.9e-05   23.7   0.0     2   232  3719  3951  3718  3966 0.76 3-beta hydroxysteroid dehydrogenase/isomerase familyAMP-binding_C        PF13193.2     71 gi|47109414|emb|CAG28797.1| -           4034   4.6e-05   24.2   0.0   1   1   2.8e-07   0.00019   22.3   0.0     2    71  3490  3573  3489  3573 0.83 AMP-binding enzyme C-terminal domainThiolase_N           PF00108.19   260 gi|47109414|emb|CAG28797.1| -           4034   7.1e-05   22.2   0.0   1   1   3.2e-07   0.00021   20.6   0.0    77   113   175   211   161   230 0.91 Thiolase, N-terminal domainUbie_methyltran      PF01209.14   233 gi|47109414|emb|CAG28797.1| -           4034   7.6e-05   22.0   0.0   1   1   2.4e-07   0.00016   20.9   0.0    46   155  1426  1539  1413  1549 0.82 ubiE/COQ5 methyltransferase familyMethyltransf_16      PF10294.5    174 gi|47109414|emb|CAG28797.1| -           4034   0.00048   19.8   0.0   1   2   2.4e-06    0.0016   18.0   0.0    42   153  1423  1534  1404  1542 0.82 Lysine methyltransferaseMethyltransf_16      PF10294.5    174 gi|47109414|emb|CAG28797.1| -           4034   0.00048   19.8   0.0   2   2       6.6   4.5e+03   -2.9   0.1    55    67  3109  3121  3101  3128 0.81 Lysine methyltransferaseGDP_Man_Dehyd        PF16363.1    332 gi|47109414|emb|CAG28797.1| -           4034    0.0018   17.6   0.0   1   2      0.93   6.3e+02   -0.7   0.0     9    73  2154  2213  2148  2217 0.83 GDP-mannose 4,6 dehydrataseGDP_Man_Dehyd        PF16363.1    332 gi|47109414|emb|CAG28797.1| -           4034    0.0018   17.6   0.0   2   2   1.4e-05    0.0094   15.2   0.0     1   182  3718  3899  3718  3954 0.74 GDP-mannose 4,6 dehydrataseNIP_1                PF08995.6     82 tr|Q02039|Q02039_RHYSE -             82   3.4e-57  190.6   9.2   1   1   2.3e-61   3.7e-57  190.5   9.2     1    82     1    82     1    82 1.00 Necrosis inducing protein-1AvrL567-A            PF11529.4    127 gi|45504829|gb|AAS66948.1| -            150   1.3e-81  270.9   0.0   1   1   9.4e-86   1.5e-81  270.7   0.0     1   127    24   150    24   150 1.00 Melampsora lini avirulence protein AvrL567-ANAD_binding_4        PF07993.8    257 gi|83026457|gb|ABB96264.1| -            210   0.00055   19.0   0.1   1   1   1.7e-07    0.0009   18.3   0.1   130   191    68   133    56   140 0.74 Male sterility proteinTBPIP                PF07106.9    169 gi|83026457|gb|ABB96264.1| -            210    0.0043   16.7   1.9   1   1   1.5e-06    0.0081   15.8   1.9   107   163    70   127    60   129 0.92 Tat binding protein 1(TBP-1)-interacting protein (TBPIP)DUF2492              PF10678.5     77 gi|83026457|gb|ABB96264.1| -            210      0.14   12.4   0.1   1   2   0.00033       1.8    8.8   0.0    29    62    54    88    26    99 0.83 Protein of unknown function (DUF2492)DUF2492              PF10678.5     77 gi|83026457|gb|ABB96264.1| -            210      0.14   12.4   0.1   2   2      0.11   6.1e+02    0.7   0.0    15    36   130   151   125   156 0.76 Protein of unknown function (DUF2492)DUF4559              PF15112.2    311 gi|83026451|gb|ABB96261.1| -            199     0.079   12.6   5.3   1   2     0.049   2.6e+02    1.0   2.6   220   279    61   120    21   145 0.60 Domain of unknown function (DUF4559)DUF4559              PF15112.2    311 gi|83026451|gb|ABB96261.1| -            199     0.079   12.6   5.3   2   2     2e-05      0.11   12.2   0.1   150   183   156   189   141   198 0.87 Domain of unknown function (DUF4559)SVWC                 PF15430.2     66 gi|83026451|gb|ABB96261.1| -            199     0.098   12.8   5.5   1   1   2.9e-05      0.16   12.1   5.5    12    56    59   103    49   104 0.81 Single domain von Willebrand factor type CDUF4407              PF14362.2    298 gi|83026451|gb|ABB96261.1| -            199      0.83    8.8   3.6   1   1    0.0002       1.1    8.4   3.6   152   252    54   162    33   176 0.76 Domain of unknown function (DUF4407)## Program:         hmmscan# Version:         hmmer3.1-snap20121016 (October 2012)# Pipeline mode:   SCAN# Query file:      effector_prots.fasta# Target file:     Pfam-A.hmm# Option settings: hmmscan --domtblout effector_prots.pfam --cpu 8 Pfam-A.hmm effector_prots.fasta # Current dir:     /fungi/taruna/shared/bin/hmmer# Date:            Thu Nov  5 16:19:26 2015# [ok]
