# Supplementary material for: De novo genome assembly of Geosmithia morbida, the causal agent of thousand cankers disease
Source: PeerJ. 2016 May 2;4:e1952. doi: 10.7717/peerj.1952 (PMC4860301; doi:10.7717/peerj.1952)
Supplement: Supplemental Information 8 [file peerj-04-1952-s008.rtf]

#                                                                                                  --- full sequence --- -------------- this domain -------------   hmm coord   ali coord   env coord# target name        accession   tlen query name                                 accession   qlen   E-value  score  bias   #  of  c-Evalue  i-Evalue  score  bias  from    to  from    to  from    to  acc description of target#------------------- ---------- -----                       -------------------- ---------- ----- --------- ------ ----- --- --- --------- --------- ------ ----- ----- ----- ----- ----- ----- ----- ---- ---------------------ADH_N                PF08240.8    109 maker-scaffold_25-augustus-gene-0.4-mRNA-1 -            366   1.1e-27   95.9   3.0   1   1   1.1e-30   1.8e-27   95.2   3.0     1   106    31   148    31   151 0.94 Alcohol dehydrogenase GroES-like domainADH_zinc_N           PF00107.22   130 maker-scaffold_25-augustus-gene-0.4-mRNA-1 -            366   1.5e-27   95.9   0.5   1   1   1.5e-30   2.4e-27   95.2   0.5     1   129   193   321   193   322 0.98 Zinc-binding dehydrogenaseADH_zinc_N_2         PF13602.2    134 maker-scaffold_25-augustus-gene-0.4-mRNA-1 -            366   1.1e-07   32.8   0.1   1   1   1.2e-10   1.9e-07   32.1   0.1     2   106   227   328   226   342 0.78 Zinc-binding dehydrogenaseGlu_dehyd_C          PF16912.1    212 maker-scaffold_25-augustus-gene-0.4-mRNA-1 -            366   1.3e-07   31.2   0.3   1   1   1.1e-10   1.7e-07   30.8   0.3     2   212   158   362   157   362 0.76 Glucose dehydrogenase C-terminusNAD_binding_7        PF13241.2    105 maker-scaffold_25-augustus-gene-0.4-mRNA-1 -            366    0.0094   16.2   0.1   1   1   1.5e-05     0.024   14.9   0.1     9    93   185   282   182   288 0.72 Putative NAD(P)-bindingADH_N_assoc          PF13823.2     23 maker-scaffold_25-augustus-gene-0.4-mRNA-1 -            366     0.014   15.2   0.1   1   1   1.9e-05     0.031   14.1   0.1     2    22     9    29     9    29 0.88 Alcohol dehydrogenase GroES-associatedShikimate_DH         PF01488.16   138 maker-scaffold_25-augustus-gene-0.4-mRNA-1 -            366     0.016   15.1   0.0   1   1   1.8e-05     0.029   14.3   0.0    12    70   183   240   175   283 0.73 Shikimate / quinate 5-dehydrogenaseTrkA_N               PF02254.14   116 maker-scaffold_25-augustus-gene-0.4-mRNA-1 -            366     0.031   14.4   0.0   1   1   3.5e-05     0.056   13.6   0.0     2    55   187   240   186   244 0.78 TrkA-N domainFAD_binding_3        PF01494.15   356 maker-scaffold_25-augustus-gene-0.4-mRNA-1 -            366     0.045   12.9   0.1   1   1     4e-05     0.066   12.3   0.1     3    39   185   221   183   234 0.89 FAD binding domainPyr_redox_3          PF13738.2    214 maker-scaffold_25-augustus-gene-0.4-mRNA-1 -            366      0.12   12.3   0.1   1   1   0.00013      0.22   11.4   0.1     1    30   187   216   187   241 0.89 Pyridine nucleotide-disulphide oxidoreductaseRRM_1                PF00076.18    70 snap_masked-scaffold_25-processed-gene-0.20-mRNA-1 -            380   4.1e-15   55.2   0.0   1   1   2.1e-18   1.2e-14   53.8   0.0     1    69   154   223   154   224 0.97 RNA recognition motif. (a.k.a. RRM, RBD, or RNP domain)RRM_5                PF13893.2     56 snap_masked-scaffold_25-processed-gene-0.20-mRNA-1 -            380   6.6e-08   32.8   0.1   1   1   3.4e-11   1.8e-07   31.3   0.1     3    54   170   226   169   228 0.95 RNA recognition motif. (a.k.a. RRM, RBD, or RNP domain)RRM_6                PF14259.2     66 snap_masked-scaffold_25-processed-gene-0.20-mRNA-1 -            380   4.3e-06   26.6   0.4   1   2       2.6   1.4e+04   -3.8   0.1    45    60    57    72    56    74 0.76 RNA recognition motif (a.k.a. RRM, RBD, or RNP domain)RRM_6                PF14259.2     66 snap_masked-scaffold_25-processed-gene-0.20-mRNA-1 -            380   4.3e-06   26.6   0.4   2   2   1.6e-09   8.6e-06   25.7   0.0     1    62   154   220   154   224 0.91 RNA recognition motif (a.k.a. RRM, RBD, or RNP domain)Asp                  PF00026.19   317 snap_masked-scaffold_25-processed-gene-0.23-mRNA-1 -            515   9.6e-65  219.0   3.5   1   1     3e-68   1.2e-64  218.6   3.5     1   316   120   427   120   428 0.95 Eukaryotic aspartyl proteaseTAXi_N               PF14543.2    167 snap_masked-scaffold_25-processed-gene-0.23-mRNA-1 -            515   7.4e-09   35.9   2.3   1   2   3.2e-12   1.3e-08   35.1   0.2     1   167   121   275   121   275 0.79 Xylanase inhibitor N-terminalTAXi_N               PF14543.2    167 snap_masked-scaffold_25-processed-gene-0.23-mRNA-1 -            515   7.4e-09   35.9   2.3   2   2      0.19   7.7e+02    0.0   0.2   112   141   480   509   420   514 0.58 Xylanase inhibitor N-terminalTAXi_C               PF14541.2    162 snap_masked-scaffold_25-processed-gene-0.23-mRNA-1 -            515    0.0031   17.1   0.0   1   2      0.29   1.2e+03   -1.0   0.0    29    54   314   339   298   355 0.75 Xylanase inhibitor C-terminalTAXi_C               PF14541.2    162 snap_masked-scaffold_25-processed-gene-0.23-mRNA-1 -            515    0.0031   17.1   0.0   2   2   2.9e-06     0.012   15.2   0.0    96   161   362   426   342   427 0.89 Xylanase inhibitor C-terminalgag-asp_proteas      PF13975.2     72 snap_masked-scaffold_25-processed-gene-0.23-mRNA-1 -            515      0.12   12.3   1.1   1   3    0.0013       5.2    7.1   0.0    11    31   123   145   117   151 0.85 gag-polyprotein putative aspartyl proteasegag-asp_proteas      PF13975.2     72 snap_masked-scaffold_25-processed-gene-0.23-mRNA-1 -            515      0.12   12.3   1.1   2   3     0.057   2.3e+02    1.8   0.4    23    34   230   241   227   241 0.86 gag-polyprotein putative aspartyl proteasegag-asp_proteas      PF13975.2     72 snap_masked-scaffold_25-processed-gene-0.23-mRNA-1 -            515      0.12   12.3   1.1   3   3      0.54   2.2e+03   -1.4   0.0    29    47   452   470   451   476 0.79 gag-polyprotein putative aspartyl proteasePPTA                 PF01239.18    28 augustus_masked-scaffold_25-processed-gene-0.9-mRNA-1 -            329   0.00015   21.3  20.0   1   4   3.5e-07    0.0057   16.3   0.4     8    27   148   167   143   168 0.81 Protein prenyltransferase alpha subunit repeatPPTA                 PF01239.18    28 augustus_masked-scaffold_25-processed-gene-0.9-mRNA-1 -            329   0.00015   21.3  20.0   2   4   1.2e-05      0.19   11.4   1.7    11    26   192   207   191   208 0.89 Protein prenyltransferase alpha subunit repeatPPTA                 PF01239.18    28 augustus_masked-scaffold_25-processed-gene-0.9-mRNA-1 -            329   0.00015   21.3  20.0   3   4     0.051   8.2e+02   -0.1   0.1    22    27   226   231   225   232 0.85 Protein prenyltransferase alpha subunit repeatPPTA                 PF01239.18    28 augustus_masked-scaffold_25-processed-gene-0.9-mRNA-1 -            329   0.00015   21.3  20.0   4   4    0.0019        30    4.4   0.9    14    25   275   286   274   287 0.90 Protein prenyltransferase alpha subunit repeatGRAB                 PF10375.5     19 snap_masked-scaffold_25-processed-gene-0.21-mRNA-1 -            730   7.1e-09   35.0   0.1   1   1     2e-11   1.4e-08   34.1   0.1     1    19   357   375   357   375 0.97 GRIP-related Arf-binding domainSpc7                 PF08317.7    310 snap_masked-scaffold_25-processed-gene-0.21-mRNA-1 -            730     0.003   16.4  24.3   1   3   0.00034      0.25   10.1   7.2   169   238    50   119    44   120 0.91 Spc7 kinetochore proteinSpc7                 PF08317.7    310 snap_masked-scaffold_25-processed-gene-0.21-mRNA-1 -            730     0.003   16.4  24.3   2   3     4e-06     0.003   16.4  24.3   135   292    62   220    60   230 0.92 Spc7 kinetochore proteinSpc7                 PF08317.7    310 snap_masked-scaffold_25-processed-gene-0.21-mRNA-1 -            730     0.003   16.4  24.3   3   3      0.33   2.4e+02    0.2   8.7   169   245   223   303   213   341 0.52 Spc7 kinetochore proteinF_actin_bind         PF08919.6    110 snap_masked-scaffold_25-processed-gene-0.21-mRNA-1 -            730     0.014   15.3   9.0   1   4      0.12        85    3.2   0.1    74   108    81   117    54   119 0.87 F-actin bindingF_actin_bind         PF08919.6    110 snap_masked-scaffold_25-processed-gene-0.21-mRNA-1 -            730     0.014   15.3   9.0   2   4     0.016        12    5.9   1.3    65   106   132   172   130   204 0.87 F-actin bindingF_actin_bind         PF08919.6    110 snap_masked-scaffold_25-processed-gene-0.21-mRNA-1 -            730     0.014   15.3   9.0   3   4         1   7.7e+02    0.1   0.0     3    25   186   208   184   232 0.79 F-actin bindingF_actin_bind         PF08919.6    110 snap_masked-scaffold_25-processed-gene-0.21-mRNA-1 -            730     0.014   15.3   9.0   4   4    0.0057       4.2    7.4   0.1    64   107   226   271   217   274 0.79 F-actin bindingDUF1664              PF07889.8    124 snap_masked-scaffold_25-processed-gene-0.21-mRNA-1 -            730     0.021   14.7  22.9   1   4   0.00088      0.65    9.9   2.7    54   122    50   115    37   116 0.84 Protein of unknown function (DUF1664)DUF1664              PF07889.8    124 snap_masked-scaffold_25-processed-gene-0.21-mRNA-1 -            730     0.021   14.7  22.9   2   4    0.0005      0.37   10.6   1.8    49   106   113   173   104   181 0.52 Protein of unknown function (DUF1664)DUF1664              PF07889.8    124 snap_masked-scaffold_25-processed-gene-0.21-mRNA-1 -            730     0.021   14.7  22.9   3   4      0.55   4.1e+02    0.8   0.8    83   120   231   268   177   271 0.54 Protein of unknown function (DUF1664)DUF1664              PF07889.8    124 snap_masked-scaffold_25-processed-gene-0.21-mRNA-1 -            730     0.021   14.7  22.9   4   4    0.0047       3.4    7.5   1.4    41    88   252   300   248   328 0.83 Protein of unknown function (DUF1664)NPV_P10              PF05531.8     75 snap_masked-scaffold_25-processed-gene-0.21-mRNA-1 -            730      0.15   12.4  12.7   1   4     0.014        10    6.6   1.1    22    66    59   118    46   127 0.50 Nucleopolyhedrovirus P10 proteinNPV_P10              PF05531.8     75 snap_masked-scaffold_25-processed-gene-0.21-mRNA-1 -            730      0.15   12.4  12.7   2   4       1.2   8.7e+02    0.3   0.4    32    51   126   145   109   204 0.67 Nucleopolyhedrovirus P10 proteinNPV_P10              PF05531.8     75 snap_masked-scaffold_25-processed-gene-0.21-mRNA-1 -            730      0.15   12.4  12.7   3   4   0.00065      0.48   10.8   0.4    16    63   226   270   220   276 0.89 Nucleopolyhedrovirus P10 proteinNPV_P10              PF05531.8     75 snap_masked-scaffold_25-processed-gene-0.21-mRNA-1 -            730      0.15   12.4  12.7   4   4     0.048        35    4.8   1.2    17    60   255   296   253   326 0.90 Nucleopolyhedrovirus P10 proteinFilament             PF00038.17   312 snap_masked-scaffold_25-processed-gene-0.21-mRNA-1 -            730      0.18   11.2  40.3   1   2     0.013       9.4    5.6  23.7   119   277    57   187    44   205 0.52 Intermediate filament proteinFilament             PF00038.17   312 snap_masked-scaffold_25-processed-gene-0.21-mRNA-1 -            730      0.18   11.2  40.3   2   2    0.0029       2.1    7.7   9.1    52   147   233   328   211   356 0.75 Intermediate filament proteinFib_alpha            PF08702.6    143 snap_masked-scaffold_25-processed-gene-0.21-mRNA-1 -            730      0.26   11.3  20.4   1   3   0.00017      0.12   12.4   7.5    25   111    52   142    49   151 0.78 Fibrinogen alpha/beta chain familyFib_alpha            PF08702.6    143 snap_masked-scaffold_25-processed-gene-0.21-mRNA-1 -            730      0.26   11.3  20.4   2   3     0.021        15    5.6   1.6    23   100   223   297   221   304 0.68 Fibrinogen alpha/beta chain familyFib_alpha            PF08702.6    143 snap_masked-scaffold_25-processed-gene-0.21-mRNA-1 -            730      0.26   11.3  20.4   3   3     0.046        34    4.5   1.8    30   109   258   337   246   352 0.63 Fibrinogen alpha/beta chain familyBaculo_PEP_C         PF04513.8    140 snap_masked-scaffold_25-processed-gene-0.21-mRNA-1 -            730      0.38   10.7  14.5   1   3   0.00032      0.24   11.3   1.8    23    88    58   122    40   141 0.80 Baculovirus polyhedron envelope protein, PEP, C terminusBaculo_PEP_C         PF04513.8    140 snap_masked-scaffold_25-processed-gene-0.21-mRNA-1 -            730      0.38   10.7  14.5   2   3     0.031        23    4.9   1.9    32   118   106   198   104   213 0.76 Baculovirus polyhedron envelope protein, PEP, C terminusBaculo_PEP_C         PF04513.8    140 snap_masked-scaffold_25-processed-gene-0.21-mRNA-1 -            730      0.38   10.7  14.5   3   3     0.024        18    5.2   0.7    45    88   228   277   214   295 0.62 Baculovirus polyhedron envelope protein, PEP, C terminusDUF4407              PF14362.2    298 snap_masked-scaffold_25-processed-gene-0.21-mRNA-1 -            730      0.51    9.5  39.5   1   2    0.0031       2.3    7.4  22.8   130   233    39   177    11   216 0.70 Domain of unknown function (DUF4407)DUF4407              PF14362.2    298 snap_masked-scaffold_25-processed-gene-0.21-mRNA-1 -            730      0.51    9.5  39.5   2   2     0.012       9.2    5.4   8.4   120   227   230   336   210   376 0.64 Domain of unknown function (DUF4407)TPR_MLP1_2           PF07926.8    129 snap_masked-scaffold_25-processed-gene-0.21-mRNA-1 -            730      0.54   10.2  55.4   1   4     0.025        18    5.2   4.0    53    86    44    77    39    81 0.83 TPR/MLP1/MLP2-like proteinTPR_MLP1_2           PF07926.8    129 snap_masked-scaffold_25-processed-gene-0.21-mRNA-1 -            730      0.54   10.2  55.4   2   4   4.6e-05     0.034   14.1  14.1    56   122    79   145    75   151 0.95 TPR/MLP1/MLP2-like proteinTPR_MLP1_2           PF07926.8    129 snap_masked-scaffold_25-processed-gene-0.21-mRNA-1 -            730      0.54   10.2  55.4   3   4    0.0059       4.4    7.2   5.2    59   106   156   203   143   207 0.64 TPR/MLP1/MLP2-like proteinTPR_MLP1_2           PF07926.8    129 snap_masked-scaffold_25-processed-gene-0.21-mRNA-1 -            730      0.54   10.2  55.4   4   4      0.16   1.2e+02    2.6   6.8    74   127   263   316   234   318 0.77 TPR/MLP1/MLP2-like proteinHemagglutinin        PF00509.14   550 snap_masked-scaffold_25-processed-gene-0.21-mRNA-1 -            730      0.62    8.3   2.2   1   2       0.5   3.7e+02   -0.9   0.1   388   416    47    75    32    93 0.77 HaemagglutininHemagglutinin        PF00509.14   550 snap_masked-scaffold_25-processed-gene-0.21-mRNA-1 -            730      0.62    8.3   2.2   2   2    0.0013      0.97    7.6   0.6   364   416    94   146    86   157 0.91 HaemagglutininReo_sigmaC           PF04582.8    326 snap_masked-scaffold_25-processed-gene-0.21-mRNA-1 -            730      0.77    8.9  13.6   1   3     0.015        11    5.2   0.7    73   124    44    92    29   119 0.80 Reovirus sigma C capsid proteinReo_sigmaC           PF04582.8    326 snap_masked-scaffold_25-processed-gene-0.21-mRNA-1 -            730      0.77    8.9  13.6   2   3    0.0032       2.3    7.4   3.0    27   104   125   206   109   246 0.64 Reovirus sigma C capsid proteinReo_sigmaC           PF04582.8    326 snap_masked-scaffold_25-processed-gene-0.21-mRNA-1 -            730      0.77    8.9  13.6   3   3      0.07        52    3.0   0.4    46   145   225   335   213   362 0.48 Reovirus sigma C capsid proteinSHE3                 PF17078.1    228 snap_masked-scaffold_25-processed-gene-0.21-mRNA-1 -            730      0.94    9.0  24.2   1   3       0.1        74    2.8   5.4   126   188    55   117    45   126 0.46 SWI5-dependent HO expression protein 3SHE3                 PF17078.1    228 snap_masked-scaffold_25-processed-gene-0.21-mRNA-1 -            730      0.94    9.0  24.2   2   3   0.00044      0.32   10.5   6.5    67   152   120   205   113   228 0.90 SWI5-dependent HO expression protein 3SHE3                 PF17078.1    228 snap_masked-scaffold_25-processed-gene-0.21-mRNA-1 -            730      0.94    9.0  24.2   3   3     0.053        39    3.7   0.5    67   105   229   267   225   296 0.70 SWI5-dependent HO expression protein 3CAMP_factor          PF07373.7    220 snap_masked-scaffold_25-processed-gene-0.21-mRNA-1 -            730         2    7.8  10.8   1   3      0.73   5.4e+02   -0.1   0.3    45    61    63    79    49   127 0.54 CAMP factor (Cfa)CAMP_factor          PF07373.7    220 snap_masked-scaffold_25-processed-gene-0.21-mRNA-1 -            730         2    7.8  10.8   2   3   0.00021      0.16   11.4   0.2     5    55   152   204   148   220 0.81 CAMP factor (Cfa)CAMP_factor          PF07373.7    220 snap_masked-scaffold_25-processed-gene-0.21-mRNA-1 -            730         2    7.8  10.8   3   3      0.71   5.2e+02   -0.1   0.3    17    78   255   314   232   328 0.59 CAMP factor (Cfa)HOOK                 PF05622.8    702 snap_masked-scaffold_25-processed-gene-0.21-mRNA-1 -            730       2.6    6.0  42.3   1   2     0.011         8    4.3  30.9   391   571    47   220    40   266 0.71 HOOK proteinHOOK                 PF05622.8    702 snap_masked-scaffold_25-processed-gene-0.21-mRNA-1 -            730       2.6    6.0  42.3   2   2     0.016        12    3.8   8.0   468   567   240   339   221   362 0.73 HOOK proteinFez1                 PF06818.11   187 snap_masked-scaffold_25-processed-gene-0.21-mRNA-1 -            730       3.3    8.0  41.6   1   3      0.32   2.3e+02    2.0   6.3    86   147    47   103    13   105 0.62 Fez1Fez1                 PF06818.11   187 snap_masked-scaffold_25-processed-gene-0.21-mRNA-1 -            730       3.3    8.0  41.6   2   3    0.0037       2.7    8.3  24.5    11   152    63   189    44   192 0.64 Fez1Fez1                 PF06818.11   187 snap_masked-scaffold_25-processed-gene-0.21-mRNA-1 -            730       3.3    8.0  41.6   3   3      0.25   1.9e+02    2.3   9.4    73   148   200   291   189   406 0.68 Fez1TSC22                PF01166.14    57 snap_masked-scaffold_25-processed-gene-0.21-mRNA-1 -            730       4.9    7.4   9.5   1   3      0.58   4.3e+02    1.2   1.3    11    28    59    76    51    97 0.75 TSC-22/dip/bun familyTSC22                PF01166.14    57 snap_masked-scaffold_25-processed-gene-0.21-mRNA-1 -            730       4.9    7.4   9.5   2   3    0.0049       3.6    7.8   1.3    13    34   125   146   124   153 0.89 TSC-22/dip/bun familyTSC22                PF01166.14    57 snap_masked-scaffold_25-processed-gene-0.21-mRNA-1 -            730       4.9    7.4   9.5   3   3       9.8   7.3e+03   -2.7   0.0    20    34   227   241   224   245 0.58 TSC-22/dip/bun familyATG16                PF08614.7    200 snap_masked-scaffold_25-processed-gene-0.21-mRNA-1 -            730       6.6    6.7  52.4   1   4     0.036        27    4.8  12.1    57   141    20   119     2   120 0.67 Autophagy protein 16 (ATG16)ATG16                PF08614.7    200 snap_masked-scaffold_25-processed-gene-0.21-mRNA-1 -            730       6.6    6.7  52.4   2   4    0.0053       3.9    7.5  13.2   106   166   116   176   111   190 0.92 Autophagy protein 16 (ATG16)ATG16                PF08614.7    200 snap_masked-scaffold_25-processed-gene-0.21-mRNA-1 -            730       6.6    6.7  52.4   3   4       0.6   4.4e+02    0.8   3.5    67   113   221   267   176   271 0.46 Autophagy protein 16 (ATG16)ATG16                PF08614.7    200 snap_masked-scaffold_25-processed-gene-0.21-mRNA-1 -            730       6.6    6.7  52.4   4   4     0.063        47    4.0  13.0    77   180   252   359   218   362 0.79 Autophagy protein 16 (ATG16)Nup54                PF13874.2    140 snap_masked-scaffold_25-processed-gene-0.21-mRNA-1 -            730       6.9    6.6  27.0   1   3    0.0079       5.8    6.9   1.6    93   135    48    91    43    95 0.74 Nucleoporin complex subunit 54Nup54                PF13874.2    140 snap_masked-scaffold_25-processed-gene-0.21-mRNA-1 -            730       6.9    6.6  27.0   2   3     0.011       8.2    6.4   8.2    32   131   115   210   114   218 0.81 Nucleoporin complex subunit 54Nup54                PF13874.2    140 snap_masked-scaffold_25-processed-gene-0.21-mRNA-1 -            730       6.9    6.6  27.0   3   3     0.062        45    4.0   2.1    28    79   241   292   224   303 0.81 Nucleoporin complex subunit 54Mnd1                 PF03962.11   187 snap_masked-scaffold_25-processed-gene-0.21-mRNA-1 -            730       7.9    6.2  42.3   1   4    0.0014         1    9.1  16.5    61   153    40   137    36   143 0.80 Mnd1 familyMnd1                 PF03962.11   187 snap_masked-scaffold_25-processed-gene-0.21-mRNA-1 -            730       7.9    6.2  42.3   2   4       1.3   9.2e+02   -0.5  16.2    62   146   119   204   112   265 0.70 Mnd1 familyMnd1                 PF03962.11   187 snap_masked-scaffold_25-processed-gene-0.21-mRNA-1 -            730       7.9    6.2  42.3   3   4     0.061        45    3.7   6.9    70   144   222   294   212   330 0.70 Mnd1 familyMnd1                 PF03962.11   187 snap_masked-scaffold_25-processed-gene-0.21-mRNA-1 -            730       7.9    6.2  42.3   4   4      0.16   1.2e+02    2.4   4.5    68   143   270   349   258   363 0.57 Mnd1 familyTup_N                PF08581.6     77 snap_masked-scaffold_25-processed-gene-0.21-mRNA-1 -            730       8.8    6.6  20.6   1   5   5.9e-05     0.043   14.0   1.4    28    76    44    92    34    93 0.90 Tup N-terminalTup_N                PF08581.6     77 snap_masked-scaffold_25-processed-gene-0.21-mRNA-1 -            730       8.8    6.6  20.6   2   5       1.1   8.4e+02    0.2   3.7    24    63   111   150    97   167 0.70 Tup N-terminalTup_N                PF08581.6     77 snap_masked-scaffold_25-processed-gene-0.21-mRNA-1 -            730       8.8    6.6  20.6   3   5      0.76   5.6e+02    0.8   0.1     9    41   159   191   154   209 0.68 Tup N-terminalTup_N                PF08581.6     77 snap_masked-scaffold_25-processed-gene-0.21-mRNA-1 -            730       8.8    6.6  20.6   4   5       1.8   1.3e+03   -0.4   0.0    46    65   228   247   222   254 0.83 Tup N-terminalTup_N                PF08581.6     77 snap_masked-scaffold_25-processed-gene-0.21-mRNA-1 -            730       8.8    6.6  20.6   5   5       2.6   1.9e+03   -0.9   0.4    34    45   261   272   232   302 0.50 Tup N-terminalDUF3138              PF11336.4    510 snap_masked-scaffold_25-processed-gene-0.21-mRNA-1 -            730         9    4.6   6.9   1   2    0.0011      0.81    8.1   0.6    17   107    41   152    33   232 0.69 Protein of unknown function (DUF3138)DUF3138              PF11336.4    510 snap_masked-scaffold_25-processed-gene-0.21-mRNA-1 -            730         9    4.6   6.9   2   2      0.51   3.7e+02   -0.7   0.9    27    95   258   330   235   340 0.50 Protein of unknown function (DUF3138)DUF4131              PF13567.2    176 augustus_masked-scaffold_25-processed-gene-0.15-mRNA-1 -            359       5.1    6.5   6.2   1   2   0.00016       2.6    7.5   0.2    17    92    11   102     2   124 0.69 Domain of unknown function (DUF4131)DUF4131              PF13567.2    176 augustus_masked-scaffold_25-processed-gene-0.15-mRNA-1 -            359       5.1    6.5   6.2   2   2     0.019   3.1e+02    0.7   1.7    35    60   205   253   150   281 0.56 Domain of unknown function (DUF4131)PhyH                 PF05721.9    211 augustus_masked-scaffold_25-processed-gene-0.18-mRNA-1 -            290   1.5e-19   71.0   0.0   1   1   2.4e-23     2e-19   70.6   0.0     2   202    19   222    18   225 0.86 Phytanoyl-CoA dioxygenase (PhyH)DUF1479              PF07350.8    415 augustus_masked-scaffold_25-processed-gene-0.18-mRNA-1 -            290    0.0032   16.2   0.0   1   1   6.2e-07     0.005   15.5   0.0    57   104     4    51     2    67 0.87 Protein of unknown function (DUF1479)AA_permease          PF00324.17   479 maker-scaffold_25-augustus-gene-0.7-mRNA-1 -            543  2.5e-112  375.8  39.7   1   1  3.7e-116    3e-112  375.5  39.7     1   472    37   498    37   504 0.96 Amino acid permeaseAA_permease_2        PF13520.2    425 maker-scaffold_25-augustus-gene-0.7-mRNA-1 -            543   3.6e-28   98.4  43.7   1   1   5.5e-32   4.4e-28   98.1  43.7     8   420    40   480    33   489 0.76 Amino acid permeaseCu_bind_like         PF02298.13    84 augustus_masked-scaffold_25-processed-gene-0.13-mRNA-1 -            247   0.00023   21.0   0.2   1   1     1e-07   0.00084   19.2   0.2    19    84    71   145    65   145 0.80 Plastocyanin-like domainCAP_N                PF01213.15   304 augustus_masked-scaffold_25-processed-gene-0.13-mRNA-1 -            247      0.15   11.4   3.5   1   2    0.0036        29    4.0   0.1   231   252    31    47     8    89 0.61 Adenylate cyclase associated (CAP) N terminalCAP_N                PF01213.15   304 augustus_masked-scaffold_25-processed-gene-0.13-mRNA-1 -            247      0.15   11.4   3.5   2   2   0.00016       1.3    8.4   0.7   204   252   134   186   121   203 0.60 Adenylate cyclase associated (CAP) N terminalCHD5                 PF04420.10   159 augustus_masked-scaffold_25-processed-gene-0.16-mRNA-1 -            214   7.6e-58  194.7   1.0   1   1   2.1e-61   1.2e-57  194.1   1.0     2   159     8   176     7   176 0.94 CHD5-like proteinDUF2508              PF10704.5     71 augustus_masked-scaffold_25-processed-gene-0.16-mRNA-1 -            214     0.045   13.8   0.4   1   1   1.7e-05     0.092   12.8   0.1    13    40    89   116    79   124 0.85 Protein of unknown function (DUF2508)WXG100               PF06013.8     86 augustus_masked-scaffold_25-processed-gene-0.16-mRNA-1 -            214      0.16   12.1   2.0   1   1   4.9e-05      0.26   11.4   2.0    24    81    55   112    50   115 0.91 Proteins of 100 residues with WXGSMN                  PF06003.8    264 augustus_masked-scaffold_25-processed-gene-0.14-mRNA-1 -            137   2.8e-06   26.7   6.9   1   2    0.0002       3.3    6.7   1.0     7    27    11    31     7    76 0.67 Survival motor neuron protein (SMN)SMN                  PF06003.8    264 augustus_masked-scaffold_25-processed-gene-0.14-mRNA-1 -            137   2.8e-06   26.7   6.9   2   2   1.7e-09   2.7e-05   23.4   0.6   207   252    87   133    75   137 0.80 Survival motor neuron protein (SMN)SAP                  PF02037.23    35 augustus_masked-scaffold_27-processed-gene-0.19-mRNA-1 -            512   3.4e-13   48.9   0.6   1   2   6.3e-17   3.4e-13   48.9   0.6     2    35     5    38     4    38 0.96 SAP domainSAP                  PF02037.23    35 augustus_masked-scaffold_27-processed-gene-0.19-mRNA-1 -            512   3.4e-13   48.9   0.6   2   2      0.95   5.1e+03   -2.8   0.2    17    22   350   355   350   355 0.95 SAP domainLin-8                PF03353.11   306 augustus_masked-scaffold_27-processed-gene-0.19-mRNA-1 -            512       6.7    6.1   6.7   1   1    0.0016       8.7    5.7   6.7   132   242    46   172    26   252 0.54 Ras-mediated vulval-induction antagonistNop14                PF04147.8    831 augustus_masked-scaffold_27-processed-gene-0.19-mRNA-1 -            512       9.2    4.2  11.8   1   1    0.0023        13    3.8  11.8   279   437    34   220    10   232 0.37 Nop14-like familyDJ-1_PfpI            PF01965.20   165 snap_masked-scaffold_27-processed-gene-0.11-mRNA-1 -            281   9.4e-11   41.7   0.0   1   1   1.9e-14   1.5e-10   41.0   0.0     3   129    40   183    38   210 0.78 DJ-1/PfpI familyThiJ_like            PF17124.1    196 snap_masked-scaffold_27-processed-gene-0.11-mRNA-1 -            281     0.005   16.4   0.0   1   1   8.6e-07     0.007   15.9   0.0    74   160    99   183    72   205 0.82 ThiJ/PfpI family-likeTannase              PF07519.7    469 augustus_masked-scaffold_27-processed-gene-0.20-mRNA-1 -            584  5.4e-133  444.2   2.6   1   1  7.8e-137  6.3e-133  444.0   2.6     1   467    75   567    75   569 0.94 Tannase and feruloyl esteraseAbhydrolase_5        PF12695.3    145 augustus_masked-scaffold_27-processed-gene-0.20-mRNA-1 -            584   0.00093   19.0   0.1   1   2     0.011        88    2.9   0.1    66   129   196   260    57   278 0.84 Alpha/beta hydrolase familyAbhydrolase_5        PF12695.3    145 augustus_masked-scaffold_27-processed-gene-0.20-mRNA-1 -            584   0.00093   19.0   0.1   2   2   5.1e-06     0.042   13.7   0.0    25   145   332   498   308   498 0.66 Alpha/beta hydrolase familyGATase               PF00117.24   191 augustus_masked-scaffold_27-processed-gene-0.22-mRNA-1 -            508   1.1e-46  158.9   0.0   1   1   3.9e-50   1.6e-46  158.3   0.0     2   190   291   467   290   468 0.95 Glutamine amidotransferase class-ICPSase_sm_chain      PF00988.18   127 augustus_masked-scaffold_27-processed-gene-0.22-mRNA-1 -            508   5.2e-44  149.0   0.0   1   1   2.4e-47   9.9e-44  148.1   0.0     4   127   112   246   109   246 0.90 Carbamoyl-phosphate synthase small chain, CPSase domainPeptidase_C26        PF07722.9    216 augustus_masked-scaffold_27-processed-gene-0.22-mRNA-1 -            508   1.8e-05   24.4   0.1   1   1   3.4e-07    0.0014   18.3   0.1    96   127   348   379   308   450 0.79 Peptidase C26DJ-1_PfpI            PF01965.20   165 augustus_masked-scaffold_27-processed-gene-0.22-mRNA-1 -            508     0.037   13.7   0.0   1   1   1.6e-05     0.067   12.9   0.0    49   109   315   372   281   380 0.84 DJ-1/PfpI familyCK_II_beta           PF01214.14   182 maker-scaffold_7-augustus-gene-0.1310-mRNA-1 -            346     3e-75  251.9   0.0   1   1   1.2e-78   4.7e-75  251.2   0.0     1   182    10   256    10   256 0.93 Casein kinase II regulatory subunitRRN3                 PF05327.7    559 maker-scaffold_7-augustus-gene-0.1310-mRNA-1 -            346      0.39    9.1   1.2   1   1   0.00013      0.52    8.7   1.2   187   235    12    84    10   132 0.57 RNA polymerase I specific transcription initiation factor RRN3Spt5_N               PF11942.4     99 maker-scaffold_7-augustus-gene-0.1310-mRNA-1 -            346      0.68   10.8  11.3   1   2   4.1e-05      0.16   12.8   4.9     3    48    57    94    55   131 0.52 Spt5 transcription elongation factor, acidic N-terminalSpt5_N               PF11942.4     99 maker-scaffold_7-augustus-gene-0.1310-mRNA-1 -            346      0.68   10.8  11.3   2   2      0.15     6e+02    1.4   0.4    16    58   292   328   275   341 0.45 Spt5 transcription elongation factor, acidic N-terminalPGA2                 PF07543.8    138 maker-scaffold_7-augustus-gene-0.1310-mRNA-1 -            346       2.4    8.0   6.8   1   2   0.00059       2.4    8.0   1.2    78   112    62    97    41   109 0.58 Protein trafficking PGA2PGA2                 PF07543.8    138 maker-scaffold_7-augustus-gene-0.1310-mRNA-1 -            346       2.4    8.0   6.8   2   2     0.022        88    2.9   0.4    81   117   292   328   270   343 0.63 Protein trafficking PGA2Sec10                PF07393.7    711 maker-scaffold_7-augustus-gene-0.1289-mRNA-1 -            848  1.4e-223  744.2   0.5   1   1  3.2e-227  1.7e-223  743.9   0.5     2   708   108   842   107   845 0.97 Exocyst complex component Sec10Methyltransf_23      PF13489.2    165 maker-scaffold_7-augustus-gene-0.1289-mRNA-1 -            848     0.058   13.2   0.0   1   1   3.3e-05      0.18   11.6   0.0    61   124   341   426   310   471 0.77 Methyltransferase domainRubis-subs-bind      PF09273.7    118 maker-scaffold_7-augustus-gene-0.1289-mRNA-1 -            848       1.8    9.3   5.3   1   3         3   1.6e+04   -3.6   0.1    25    50    82   106    65   115 0.58 Rubisco LSMT substrate-bindingRubis-subs-bind      PF09273.7    118 maker-scaffold_7-augustus-gene-0.1289-mRNA-1 -            848       1.8    9.3   5.3   2   3      0.18   9.8e+02    0.4   0.0    76    98   241   261   238   289 0.80 Rubisco LSMT substrate-bindingRubis-subs-bind      PF09273.7    118 maker-scaffold_7-augustus-gene-0.1289-mRNA-1 -            848       1.8    9.3   5.3   3   3   0.00028       1.5    9.5   0.8    17    94   503   577   482   606 0.78 Rubisco LSMT substrate-bindingICL                  PF00463.17   526 augustus_masked-scaffold_7-processed-gene-0.485-mRNA-1 -            602  3.8e-198  659.1   0.0   1   1  5.4e-202  4.4e-198  658.9   0.0     2   526    78   601    77   601 0.99 Isocitrate lyase familyPEP_mutase           PF13714.2    239 augustus_masked-scaffold_7-processed-gene-0.485-mRNA-1 -            602   1.4e-11   44.2   0.0   1   2   6.5e-14   5.3e-10   39.0   0.0    29   146   158   309   126   321 0.86 Phosphoenolpyruvate phosphomutasePEP_mutase           PF13714.2    239 augustus_masked-scaffold_7-processed-gene-0.485-mRNA-1 -            602   1.4e-11   44.2   0.0   2   2    0.0069        56    2.9   0.0    85   123   336   375   332   450 0.81 Phosphoenolpyruvate phosphomutaseProteasome           PF00227.22   190 maker-scaffold_7-augustus-gene-0.1273-mRNA-1 -            197   1.2e-39  135.6   0.0   1   1   8.5e-44   1.4e-39  135.4   0.0     4   190     1   182     1   182 0.98 Proteasome subunitEF-hand_7            PF13499.2     66 augustus_masked-scaffold_7-processed-gene-0.395-mRNA-1 -            201   8.3e-15   54.7   3.6   1   1   3.8e-13   8.8e-10   38.6   3.6     4    66   138   197    46   197 0.82 EF-hand domain pairEF-hand_1            PF00036.28    29 augustus_masked-scaffold_7-processed-gene-0.395-mRNA-1 -            201   9.3e-15   52.9   7.1   1   3   0.00062       1.4    8.5   0.0     2    27    50    75    49    77 0.89 EF handEF-hand_1            PF00036.28    29 augustus_masked-scaffold_7-processed-gene-0.395-mRNA-1 -            201   9.3e-15   52.9   7.1   2   3   6.3e-09   1.5e-05   24.1   0.7     3    26   136   159   134   162 0.88 EF handEF-hand_1            PF00036.28    29 augustus_masked-scaffold_7-processed-gene-0.395-mRNA-1 -            201   9.3e-15   52.9   7.1   3   3   1.4e-08   3.2e-05   23.0   0.2     1    27   172   198   172   200 0.94 EF handEF-hand_8            PF13833.2     55 augustus_masked-scaffold_7-processed-gene-0.395-mRNA-1 -            201   3.4e-13   49.1  10.5   1   3    0.0043       9.9    6.0   0.1    28    48    50    70    45    76 0.87 EF-hand domain pairEF-hand_8            PF13833.2     55 augustus_masked-scaffold_7-processed-gene-0.395-mRNA-1 -            201   3.4e-13   49.1  10.5   2   3   3.7e-05     0.086   12.6   0.6    28    47   135   154   116   161 0.81 EF-hand domain pairEF-hand_8            PF13833.2     55 augustus_masked-scaffold_7-processed-gene-0.395-mRNA-1 -            201   3.4e-13   49.1  10.5   3   3   2.2e-15   5.1e-12   45.4   4.0     1    54   146   199   146   200 0.92 EF-hand domain pairEF-hand_6            PF13405.2     31 augustus_masked-scaffold_7-processed-gene-0.395-mRNA-1 -            201   8.1e-13   47.1   4.1   1   3   0.00014      0.33   10.9   0.0     2    31    50    78    49    78 0.89 EF-hand domainEF-hand_6            PF13405.2     31 augustus_masked-scaffold_7-processed-gene-0.395-mRNA-1 -            201   8.1e-13   47.1   4.1   2   3   3.4e-08   7.8e-05   22.2   0.4     3    25   136   158   134   166 0.89 EF-hand domainEF-hand_6            PF13405.2     31 augustus_masked-scaffold_7-processed-gene-0.395-mRNA-1 -            201   8.1e-13   47.1   4.1   3   3   1.4e-05     0.032   14.0   0.1     7    27   178   198   172   201 0.85 EF-hand domainEF-hand_5            PF13202.2     25 augustus_masked-scaffold_7-processed-gene-0.395-mRNA-1 -            201   3.3e-08   32.6   1.6   1   3     0.057   1.3e+02    2.2   0.0     2    14    51    63    50    73 0.81 EF handEF-hand_5            PF13202.2     25 augustus_masked-scaffold_7-processed-gene-0.395-mRNA-1 -            201   3.3e-08   32.6   1.6   2   3   4.3e-08   9.9e-05   21.6   0.2     2    24   136   158   135   159 0.89 EF handEF-hand_5            PF13202.2     25 augustus_masked-scaffold_7-processed-gene-0.395-mRNA-1 -            201   3.3e-08   32.6   1.6   3   3    0.0028       6.5    6.4   0.1     8    25   180   197   173   197 0.89 EF handEF-hand_9            PF14658.2     66 augustus_masked-scaffold_7-processed-gene-0.395-mRNA-1 -            201   0.00029   21.0   0.5   1   3      0.22   5.2e+02    0.9   0.0     2    14    52    64    51    96 0.80 EF-hand domainEF-hand_9            PF14658.2     66 augustus_masked-scaffold_7-processed-gene-0.395-mRNA-1 -            201   0.00029   21.0   0.5   2   3       2.3   5.2e+03   -2.3   0.0    21    58   112   118   105   134 0.59 EF-hand domainEF-hand_9            PF14658.2     66 augustus_masked-scaffold_7-processed-gene-0.395-mRNA-1 -            201   0.00029   21.0   0.5   3   3     2e-06    0.0046   17.1   0.3     3    64   138   199   136   200 0.88 EF-hand domainEF-hand_4            PF12763.3    104 augustus_masked-scaffold_7-processed-gene-0.395-mRNA-1 -            201    0.0028   17.4   1.6   1   3    0.0089        21    5.0   0.0    19    77    26    83    22   101 0.75 Cytoskeletal-regulatory complex EF handEF-hand_4            PF12763.3    104 augustus_masked-scaffold_7-processed-gene-0.395-mRNA-1 -            201    0.0028   17.4   1.6   2   3    0.0066        15    5.4   0.2    38    65   128   155   116   162 0.83 Cytoskeletal-regulatory complex EF handEF-hand_4            PF12763.3    104 augustus_masked-scaffold_7-processed-gene-0.395-mRNA-1 -            201    0.0028   17.4   1.6   3   3    0.0098        23    4.9   0.1    36    69   164   197   156   199 0.85 Cytoskeletal-regulatory complex EF handGlyco_hydro_88       PF07470.9    344 augustus_masked-scaffold_7-processed-gene-0.479-mRNA-1 -            378   1.9e-89  300.0   1.6   1   1   5.3e-93   2.1e-89  299.8   1.6     2   343    27   376    26   377 0.97 Glycosyl Hydrolase Family 88Glyco_hydro_76       PF03663.10   366 augustus_masked-scaffold_7-processed-gene-0.479-mRNA-1 -            378     0.003   17.0   6.8   1   3    0.0006       2.4    7.4   0.7   210   245    45    80    16    84 0.70 Glycosyl hydrolase family 76Glyco_hydro_76       PF03663.10   366 augustus_masked-scaffold_7-processed-gene-0.479-mRNA-1 -            378     0.003   17.0   6.8   2   3   1.9e-05     0.079   12.3   0.9   139   194    82   135    81   155 0.82 Glycosyl hydrolase family 76Glyco_hydro_76       PF03663.10   366 augustus_masked-scaffold_7-processed-gene-0.479-mRNA-1 -            378     0.003   17.0   6.8   3   3      0.47   1.9e+03   -2.1   0.0    84   147   147   209   135   215 0.65 Glycosyl hydrolase family 76Glyco_hydro_127      PF07944.8    519 augustus_masked-scaffold_7-processed-gene-0.479-mRNA-1 -            378     0.012   14.1   0.3   1   1   9.1e-06     0.037   12.4   0.3   117   211    49   132    14   160 0.74 Beta-L-arabinofuranosidase, GH127MiaE                 PF06175.7    240 augustus_masked-scaffold_7-processed-gene-0.479-mRNA-1 -            378      0.09   12.2   0.0   1   1   3.9e-05      0.16   11.4   0.0    44   109    98   165    91   172 0.74 tRNA-(MS[2]IO[6]A)-hydroxylase (MiaE)CHORD                PF04968.8     63 maker-scaffold_7-snap-gene-0.700-mRNA-1 -            330   1.6e-47  159.9  17.8   1   2   4.6e-28   3.7e-24   85.0   5.0     2    63     4    61     3    61 0.95 CHORDCHORD                PF04968.8     63 maker-scaffold_7-snap-gene-0.700-mRNA-1 -            330   1.6e-47  159.9  17.8   2   2   9.2e-27   7.5e-23   80.9   4.9     2    61   139   199   138   202 0.91 CHORDCS                   PF04969.12    79 maker-scaffold_7-snap-gene-0.700-mRNA-1 -            330   3.2e-13   50.2   0.2   1   1     6e-17   4.9e-13   49.6   0.2     2    79   225   302   224   302 0.97 CS domainGFA                  PF04828.10    93 maker-scaffold_7-augustus-gene-0.1362-mRNA-1 -            137   5.7e-27   93.8   0.3   1   1   4.7e-31   7.6e-27   93.3   0.3     2    93    25   119    24   119 0.95 Glutathione-dependent formaldehyde-activating enzymeABC2_membrane        PF01061.20   210 maker-scaffold_7-augustus-gene-0.1322-mRNA-1 -            637   1.6e-27   96.1  22.6   1   1   5.7e-30   2.8e-27   95.4  22.6     5   210   357   564   356   564 0.94 ABC-2 type transporterABC_tran             PF00005.23   137 maker-scaffold_7-augustus-gene-0.1322-mRNA-1 -            637   1.8e-25   89.9   0.0   1   1   5.5e-28   2.7e-25   89.3   0.0     5   137    49   199    45   199 0.90 ABC transporterAAA_21               PF13304.2    303 maker-scaffold_7-augustus-gene-0.1322-mRNA-1 -            637   5.7e-08   33.2   0.0   1   2   0.00021       0.1   12.6   0.0     2    20    58    76    57    96 0.84 AAA domain, putative AbiEii toxin, Type IV TA systemAAA_21               PF13304.2    303 maker-scaffold_7-augustus-gene-0.1322-mRNA-1 -            637   5.7e-08   33.2   0.0   2   2   3.9e-06    0.0019   18.3   0.0   237   284   171   215   145   227 0.91 AAA domain, putative AbiEii toxin, Type IV TA systemAAA_16               PF13191.2    177 maker-scaffold_7-augustus-gene-0.1322-mRNA-1 -            637   3.2e-06   27.6   0.4   1   1   1.8e-08   8.9e-06   26.2   0.1    19    97    52   130    34   196 0.67 AAA ATPase domainDUF258               PF03193.12   167 maker-scaffold_7-augustus-gene-0.1322-mRNA-1 -            637   7.4e-05   22.6   0.0   1   2   9.2e-07   0.00045   20.0   0.0    76   125    28    81    11    97 0.72 Protein of unknown function, DUF258DUF258               PF03193.12   167 maker-scaffold_7-augustus-gene-0.1322-mRNA-1 -            637   7.4e-05   22.6   0.0   2   2       2.1     1e+03   -0.7   0.0    17    42   220   245   208   250 0.83 Protein of unknown function, DUF258SMC_N                PF02463.15   220 maker-scaffold_7-augustus-gene-0.1322-mRNA-1 -            637   0.00043   19.6   0.0   1   1   1.7e-06   0.00083   18.7   0.0    26   197    57   227    48   248 0.78 RecF/RecN/SMC N terminal domainAAA_25               PF13481.2    194 maker-scaffold_7-augustus-gene-0.1322-mRNA-1 -            637   0.00046   19.7   0.0   1   1   1.6e-06   0.00081   18.9   0.0    27    66    47    90    30   148 0.83 AAA domainAAA_22               PF13401.2    137 maker-scaffold_7-augustus-gene-0.1322-mRNA-1 -            637   0.00086   19.4   0.0   1   1   3.4e-06    0.0017   18.5   0.0     5    32    55    82    52   147 0.81 AAA domainAAA_10               PF12846.3    304 maker-scaffold_7-augustus-gene-0.1322-mRNA-1 -            637     0.001   18.7   0.0   1   1   4.6e-06    0.0023   17.5   0.0     4    32    58    86    56   105 0.88 AAA-like domainAAA_29               PF13555.2     61 maker-scaffold_7-augustus-gene-0.1322-mRNA-1 -            637    0.0039   16.7   0.2   1   1   1.7e-05    0.0083   15.7   0.2    22    41    54    74    48    82 0.85 P-loop containing region of AAA domainAAA_17               PF13207.2    122 maker-scaffold_7-augustus-gene-0.1322-mRNA-1 -            637    0.0039   18.2   0.0   1   1   1.7e-05    0.0086   17.1   0.0     3    23    59    79    57   150 0.84 AAA domainAAA_30               PF13604.2    196 maker-scaffold_7-augustus-gene-0.1322-mRNA-1 -            637    0.0047   16.6   0.3   1   1   4.8e-05     0.024   14.3   0.3    19    78    56   110    46   232 0.60 AAA domaincobW                 PF02492.15   177 maker-scaffold_7-augustus-gene-0.1322-mRNA-1 -            637      0.01   15.3   0.1   1   1   3.7e-05     0.018   14.5   0.1     3    22    58    81    56   101 0.71 CobW/HypB/UreG, nucleotide-binding domainAAA_18               PF13238.2    129 maker-scaffold_7-augustus-gene-0.1322-mRNA-1 -            637     0.011   16.1   0.1   1   1   3.8e-05     0.019   15.3   0.1     2    23    59    88    58   117 0.81 AAA domainMMR_HSR1             PF01926.19   110 maker-scaffold_7-augustus-gene-0.1322-mRNA-1 -            637     0.012   15.6   0.1   1   1   5.5e-05     0.027   14.4   0.1     2    28    58    84    57    98 0.83 50S ribosome-binding GTPaseAAA_24               PF13479.2    205 maker-scaffold_7-augustus-gene-0.1322-mRNA-1 -            637     0.019   14.6   0.1   1   1   7.3e-05     0.036   13.7   0.1     5    27    57    79    54   111 0.90 AAA domainAAA_28               PF13521.2    163 maker-scaffold_7-augustus-gene-0.1322-mRNA-1 -            637      0.02   15.0   0.3   1   1   7.7e-05     0.038   14.1   0.3     3    22    59    78    57   105 0.83 AAA domainT2SSE                PF00437.16   277 maker-scaffold_7-augustus-gene-0.1322-mRNA-1 -            637     0.023   13.6   0.1   1   1   7.3e-05     0.036   13.0   0.1   126   153    51    78    19    91 0.85 Type II/IV secretion system proteinAAA_19               PF13245.2     76 maker-scaffold_7-augustus-gene-0.1322-mRNA-1 -            637     0.031   14.1   0.2   1   1   0.00019     0.092   12.6   0.2    11    37    56    81    47   113 0.76 Part of AAA domainAAA_33               PF13671.2    143 maker-scaffold_7-augustus-gene-0.1322-mRNA-1 -            637     0.038   14.0   0.0   1   1   0.00016     0.077   13.0   0.0     3    25    59    88    57   164 0.70 AAA domainNACHT                PF05729.8    166 maker-scaffold_7-augustus-gene-0.1322-mRNA-1 -            637     0.042   13.6   0.1   1   1   0.00018     0.087   12.6   0.1     2    25    57    80    56    90 0.84 NACHT domainAAA_5                PF07728.10   139 maker-scaffold_7-augustus-gene-0.1322-mRNA-1 -            637     0.044   13.6   0.0   1   2   0.00032      0.16   11.8   0.0     4    39    60   100    57   150 0.76 AAA domain (dynein-related subfamily)AAA_5                PF07728.10   139 maker-scaffold_7-augustus-gene-0.1322-mRNA-1 -            637     0.044   13.6   0.0   2   2       9.4   4.6e+03   -2.7   0.0    56    76   179   199   166   218 0.72 AAA domain (dynein-related subfamily)AAA_15               PF13175.2    405 maker-scaffold_7-augustus-gene-0.1322-mRNA-1 -            637     0.053   12.8   0.0   1   2     0.023        11    5.2   0.0    23    42    56    75    52    89 0.89 AAA ATPase domainAAA_15               PF13175.2    405 maker-scaffold_7-augustus-gene-0.1322-mRNA-1 -            637     0.053   12.8   0.0   2   2     0.019       9.1    5.5   0.0   361   402   190   231   181   233 0.92 AAA ATPase domainSRPRB                PF09439.6    181 maker-scaffold_7-augustus-gene-0.1322-mRNA-1 -            637     0.062   12.6   0.1   1   1   0.00022      0.11   11.8   0.1     6    42    58    94    54   108 0.76 Signal recognition particle receptor beta subunitSeptin               PF00735.14   281 maker-scaffold_7-augustus-gene-0.1322-mRNA-1 -            637     0.063   12.4   0.0   1   1   0.00019     0.094   11.8   0.0     7    37    58    88    55   131 0.64 SeptinIstB_IS21            PF01695.13   178 maker-scaffold_7-augustus-gene-0.1322-mRNA-1 -            637     0.065   12.9   0.0   1   1   0.00032      0.16   11.6   0.0    42    76    50    84    13    94 0.78 IstB-like ATP binding proteinMobB                 PF03205.10   133 maker-scaffold_7-augustus-gene-0.1322-mRNA-1 -            637     0.094   12.5   0.1   1   1   0.00036      0.18   11.6   0.1     1    27    57    83    57    91 0.86 Molybdopterin guanine dinucleotide synthesis protein BATP_bind_1           PF03029.13   238 maker-scaffold_7-augustus-gene-0.1322-mRNA-1 -            637      0.12   12.0   0.7   1   2   0.00055      0.27   10.8   0.3     1    25    60    84    60    90 0.87 Conserved hypothetical ATP binding proteinATP_bind_1           PF03029.13   238 maker-scaffold_7-augustus-gene-0.1322-mRNA-1 -            637      0.12   12.0   0.7   2   2       6.9   3.4e+03   -2.6   0.0   195   225   203   233   187   238 0.80 Conserved hypothetical ATP binding proteinAAA_23               PF13476.2    200 maker-scaffold_7-augustus-gene-0.1322-mRNA-1 -            637      0.14   12.5   0.0   1   1   0.00048      0.23   11.8   0.0    23    38    59    74    43    80 0.91 AAA domainArch_ATPase          PF01637.14   234 maker-scaffold_7-augustus-gene-0.1322-mRNA-1 -            637      0.16   11.7   0.0   1   1   0.00052      0.26   11.0   0.0    21    46    56    81    49   146 0.84 Archaeal ATPaseRNA_helicase         PF00910.18   107 maker-scaffold_7-augustus-gene-0.1322-mRNA-1 -            637      0.18   12.1   0.0   1   1   0.00071      0.35   11.1   0.0     3    26    60    83    58   104 0.78 RNA helicaseNTPase_1             PF03266.11   168 maker-scaffold_7-augustus-gene-0.1322-mRNA-1 -            637      0.19   11.5   0.1   1   1   0.00075      0.37   10.5   0.1     3    34    59    90    57   123 0.90 NTPaseRad17                PF03215.11   519 maker-scaffold_7-augustus-gene-0.1322-mRNA-1 -            637      0.26   10.0   0.0   1   1   0.00071      0.35    9.6   0.0    45    69    55    79    41   158 0.87 Rad17 cell cycle checkpoint proteinRibosomal_L16        PF00252.14   129 maker-scaffold_7-augustus-gene-0.1331-mRNA-1 -            221   1.1e-43  148.2   0.0   1   1   8.6e-48   1.4e-43  147.8   0.0     1   129    12   166    12   166 0.96 Ribosomal protein L16p/L10eAldedh               PF00171.18   462 maker-scaffold_7-snap-gene-0.724-mRNA-1 -            498  4.7e-183  608.8   0.0   1   1  3.3e-187  5.4e-183  608.6   0.0     3   462    29   490    27   490 0.99 Aldehyde dehydrogenase familyCu-oxidase_3         PF07732.11   119 maker-scaffold_7-augustus-gene-0.1347-mRNA-1 -            583     6e-46  155.2   7.2   1   3   8.4e-47   4.5e-43  145.9   0.1     2   118    75   192    74   193 0.95 Multicopper oxidaseCu-oxidase_3         PF07732.11   119 maker-scaffold_7-augustus-gene-0.1347-mRNA-1 -            583     6e-46  155.2   7.2   2   3      0.43   2.3e+03   -1.6   0.0    81   102   194   215   191   222 0.71 Multicopper oxidaseCu-oxidase_3         PF07732.11   119 maker-scaffold_7-augustus-gene-0.1347-mRNA-1 -            583     6e-46  155.2   7.2   3   3     5e-05      0.27   11.1   1.3    33   113   455   545   436   550 0.72 Multicopper oxidaseCu-oxidase           PF00394.18   159 maker-scaffold_7-augustus-gene-0.1347-mRNA-1 -            583     8e-39  133.2   1.0   1   3       2.6   1.4e+04   -4.1   0.0     8    20   167   179   166   185 0.73 Multicopper oxidaseCu-oxidase           PF00394.18   159 maker-scaffold_7-augustus-gene-0.1347-mRNA-1 -            583     8e-39  133.2   1.0   2   3   4.5e-40   2.5e-36  125.1   0.1     4   156   202   356   199   359 0.90 Multicopper oxidaseCu-oxidase           PF00394.18   159 maker-scaffold_7-augustus-gene-0.1347-mRNA-1 -            583     8e-39  133.2   1.0   3   3    0.0018       9.5    6.2   0.0    71   135   458   530   426   545 0.84 Multicopper oxidaseCu-oxidase_2         PF07731.10   137 maker-scaffold_7-augustus-gene-0.1347-mRNA-1 -            583     9e-35  119.2   9.9   1   3   0.00071       3.8    7.1   0.6    33   133    99   188    85   191 0.76 Multicopper oxidaseCu-oxidase_2         PF07731.10   137 maker-scaffold_7-augustus-gene-0.1347-mRNA-1 -            583     9e-35  119.2   9.9   2   3    0.0047        25    4.4   0.0    54   118   283   337   231   343 0.76 Multicopper oxidaseCu-oxidase_2         PF07731.10   137 maker-scaffold_7-augustus-gene-0.1347-mRNA-1 -            583     9e-35  119.2   9.9   3   3   1.9e-35     1e-31  109.3   2.3    23   136   433   549   409   550 0.86 Multicopper oxidaseApoLp-III            PF07464.7    146 maker-scaffold_7-augustus-gene-0.1330-mRNA-1 -            128    0.0023   17.9   2.0   1   1     1e-06    0.0033   17.4   2.0    51   134    24   111    22   121 0.84 Apolipophorin-III precursor (apoLp-III)HBM                  PF16591.1    254 maker-scaffold_7-augustus-gene-0.1330-mRNA-1 -            128     0.019   14.2   0.2   1   1   6.7e-06     0.022   14.0   0.2   130   180    68   114    24   124 0.72 Helical bimodular sensor domainLUC7                 PF03194.11   247 maker-scaffold_7-augustus-gene-0.1330-mRNA-1 -            128     0.025   14.1   0.0   1   1   8.8e-06     0.028   13.9   0.0    76   139    59   120    38   127 0.80 LUC7 N_terminusSNARE                PF05739.15    53 maker-scaffold_7-augustus-gene-0.1330-mRNA-1 -            128      0.29   11.0   1.9   1   2    0.0005       1.6    8.6   1.0     6    32    73    99    72   105 0.91 SNARE domainSNARE                PF05739.15    53 maker-scaffold_7-augustus-gene-0.1330-mRNA-1 -            128      0.29   11.0   1.9   2   2      0.17   5.4e+02    0.5   0.0     3    12   106   115   104   122 0.76 SNARE domainETRAMP               PF09716.6     83 maker-scaffold_7-augustus-gene-0.1330-mRNA-1 -            128       5.3    7.1   7.9   1   2      0.01        33    4.5   0.2    65    81     8    24     2    26 0.86 Malarial early transcribed membrane protein (ETRAMP)ETRAMP               PF09716.6     83 maker-scaffold_7-augustus-gene-0.1330-mRNA-1 -            128       5.3    7.1   7.9   2   2    0.0076        25    4.9   2.6    32    54    91   121    42   124 0.72 Malarial early transcribed membrane protein (ETRAMP)ATP_bind_3           PF01171.16   182 augustus_masked-scaffold_7-processed-gene-0.393-mRNA-1 -            402   8.4e-18   64.6   0.0   1   1   2.5e-21   1.4e-17   63.9   0.0     2   167    54   233    53   247 0.87 PP-loop familyzn-ribbon_14         PF16503.1     32 augustus_masked-scaffold_7-processed-gene-0.393-mRNA-1 -            402   1.7e-16   59.2   2.9   1   5       0.5   2.7e+03   -2.2   0.3     3     7     6    10     4    11 0.77 Zinc-ribbonzn-ribbon_14         PF16503.1     32 augustus_masked-scaffold_7-processed-gene-0.393-mRNA-1 -            402   1.7e-16   59.2   2.9   2   5      0.48   2.6e+03   -2.2   0.1    12    20    23    31    22    31 0.79 Zinc-ribbonzn-ribbon_14         PF16503.1     32 augustus_masked-scaffold_7-processed-gene-0.393-mRNA-1 -            402   1.7e-16   59.2   2.9   3   5     0.041   2.2e+02    1.3   0.2     1     8   139   146   139   152 0.83 Zinc-ribbonzn-ribbon_14         PF16503.1     32 augustus_masked-scaffold_7-processed-gene-0.393-mRNA-1 -            402   1.7e-16   59.2   2.9   4   5       1.6   8.4e+03   -3.8   0.0    20    29   248   257   248   258 0.81 Zinc-ribbonzn-ribbon_14         PF16503.1     32 augustus_masked-scaffold_7-processed-gene-0.393-mRNA-1 -            402   1.7e-16   59.2   2.9   5   5   3.2e-20   1.7e-16   59.2   2.9     1    31   370   400   370   401 0.95 Zinc-ribbonDZR                  PF12773.3     50 augustus_masked-scaffold_7-processed-gene-0.393-mRNA-1 -            402       7.1    6.6  10.3   1   3      0.01        55    3.8   0.9    15    35     6    29     3    37 0.62 Double zinc ribbonDZR                  PF12773.3     50 augustus_masked-scaffold_7-processed-gene-0.393-mRNA-1 -            402       7.1    6.6  10.3   2   3      0.25   1.3e+03   -0.7   0.1    15    19   141   145   133   151 0.69 Double zinc ribbonDZR                  PF12773.3     50 augustus_masked-scaffold_7-processed-gene-0.393-mRNA-1 -            402       7.1    6.6  10.3   3   3   0.00079       4.3    7.3   0.3    28    49   365   387   341   388 0.77 Double zinc ribbonTPR_14               PF13428.2     44 augustus_masked-scaffold_7-processed-gene-0.357-mRNA-1 -            685   1.1e-27   94.2  36.4   1  13       5.5   4.2e+03   -1.1   0.0    20    31    22    33    20    44 0.80 Tetratricopeptide repeatTPR_14               PF13428.2     44 augustus_masked-scaffold_7-processed-gene-0.357-mRNA-1 -            685   1.1e-27   94.2  36.4   2  13   5.8e-08   4.5e-05   23.7   0.1     4    43    75   114    72   115 0.94 Tetratricopeptide repeatTPR_14               PF13428.2     44 augustus_masked-scaffold_7-processed-gene-0.357-mRNA-1 -            685   1.1e-27   94.2  36.4   3  13   0.00013       0.1   13.3   0.1     3    42   108   147   108   150 0.93 Tetratricopeptide repeatTPR_14               PF13428.2     44 augustus_masked-scaffold_7-processed-gene-0.357-mRNA-1 -            685   1.1e-27   94.2  36.4   4  13   5.4e-05     0.042   14.5   0.1     2    43   174   214   173   215 0.91 Tetratricopeptide repeatTPR_14               PF13428.2     44 augustus_masked-scaffold_7-processed-gene-0.357-mRNA-1 -            685   1.1e-27   94.2  36.4   5  13      0.28   2.1e+02    2.9   0.0     2    34   207   239   206   241 0.89 Tetratricopeptide repeatTPR_14               PF13428.2     44 augustus_masked-scaffold_7-processed-gene-0.357-mRNA-1 -            685   1.1e-27   94.2  36.4   6  13   0.00021      0.17   12.6   0.7     4    42   246   284   243   286 0.89 Tetratricopeptide repeatTPR_14               PF13428.2     44 augustus_masked-scaffold_7-processed-gene-0.357-mRNA-1 -            685   1.1e-27   94.2  36.4   7  13   6.4e-06    0.0049   17.4   0.1     2    43   322   365   321   366 0.85 Tetratricopeptide repeatTPR_14               PF13428.2     44 augustus_masked-scaffold_7-processed-gene-0.357-mRNA-1 -            685   1.1e-27   94.2  36.4   8  13       1.8   1.4e+03    0.4   0.0     4    28   367   392   364   397 0.72 Tetratricopeptide repeatTPR_14               PF13428.2     44 augustus_masked-scaffold_7-processed-gene-0.357-mRNA-1 -            685   1.1e-27   94.2  36.4   9  13   4.2e-05     0.032   14.9   0.0     3    35   405   437   403   440 0.91 Tetratricopeptide repeatTPR_14               PF13428.2     44 augustus_masked-scaffold_7-processed-gene-0.357-mRNA-1 -            685   1.1e-27   94.2  36.4  10  13       2.4   1.9e+03    0.0   0.0     8    43   443   478   437   479 0.79 Tetratricopeptide repeatTPR_14               PF13428.2     44 augustus_masked-scaffold_7-processed-gene-0.357-mRNA-1 -            685   1.1e-27   94.2  36.4  11  13     0.048        37    5.3   0.4     3    30   472   499   471   508 0.84 Tetratricopeptide repeatTPR_14               PF13428.2     44 augustus_masked-scaffold_7-processed-gene-0.357-mRNA-1 -            685   1.1e-27   94.2  36.4  12  13   0.00023      0.18   12.5   0.1     3    41   508   545   505   548 0.84 Tetratricopeptide repeatTPR_14               PF13428.2     44 augustus_masked-scaffold_7-processed-gene-0.357-mRNA-1 -            685   1.1e-27   94.2  36.4  13  13       1.7   1.3e+03    0.5   0.1    18    30   585   597   580   608 0.83 Tetratricopeptide repeatHAT                  PF02184.12    32 augustus_masked-scaffold_7-processed-gene-0.357-mRNA-1 -            685   3.4e-25   87.6  74.4   1  15     0.018        14    5.6   1.1    18    30    70    82    55    84 0.82 HAT (Half-A-TPR) repeatHAT                  PF02184.12    32 augustus_masked-scaffold_7-processed-gene-0.357-mRNA-1 -            685   3.4e-25   87.6  74.4   2  15   4.7e-08   3.6e-05   23.5   0.4     1    27    86   113    86   117 0.90 HAT (Half-A-TPR) repeatHAT                  PF02184.12    32 augustus_masked-scaffold_7-processed-gene-0.357-mRNA-1 -            685   3.4e-25   87.6  74.4   3  15       2.5   1.9e+03   -1.2   0.2     3    31   122   151   120   152 0.74 HAT (Half-A-TPR) repeatHAT                  PF02184.12    32 augustus_masked-scaffold_7-processed-gene-0.357-mRNA-1 -            685   3.4e-25   87.6  74.4   4  15       3.3   2.6e+03   -1.6   0.1     7    30   160   183   159   185 0.81 HAT (Half-A-TPR) repeatHAT                  PF02184.12    32 augustus_masked-scaffold_7-processed-gene-0.357-mRNA-1 -            685   3.4e-25   87.6  74.4   5  15   4.2e-16   3.3e-13   49.2   2.0     2    32   188   218   187   218 0.97 HAT (Half-A-TPR) repeatHAT                  PF02184.12    32 augustus_masked-scaffold_7-processed-gene-0.357-mRNA-1 -            685   3.4e-25   87.6  74.4   6  15      0.53   4.1e+02    0.9   0.2    24    30   247   253   242   255 0.85 HAT (Half-A-TPR) repeatHAT                  PF02184.12    32 augustus_masked-scaffold_7-processed-gene-0.357-mRNA-1 -            685   3.4e-25   87.6  74.4   7  15     0.033        25    4.8   0.3     2    11   258   267   257   270 0.89 HAT (Half-A-TPR) repeatHAT                  PF02184.12    32 augustus_masked-scaffold_7-processed-gene-0.357-mRNA-1 -            685   3.4e-25   87.6  74.4   8  15       7.5   5.8e+03   -2.8   0.1    22    31   323   332   319   333 0.84 HAT (Half-A-TPR) repeatHAT                  PF02184.12    32 augustus_masked-scaffold_7-processed-gene-0.357-mRNA-1 -            685   3.4e-25   87.6  74.4   9  15    0.0066       5.1    7.0   0.1     2    12   336   346   335   353 0.90 HAT (Half-A-TPR) repeatHAT                  PF02184.12    32 augustus_masked-scaffold_7-processed-gene-0.357-mRNA-1 -            685   3.4e-25   87.6  74.4  10  15       9.4   7.3e+03   -3.1   1.3    23    31   367   375   366   376 0.93 HAT (Half-A-TPR) repeatHAT                  PF02184.12    32 augustus_masked-scaffold_7-processed-gene-0.357-mRNA-1 -            685   3.4e-25   87.6  74.4  11  15     0.011       8.4    6.3   0.0     3    11   381   389   379   393 0.87 HAT (Half-A-TPR) repeatHAT                  PF02184.12    32 augustus_masked-scaffold_7-processed-gene-0.357-mRNA-1 -            685   3.4e-25   87.6  74.4  12  15    0.0003      0.23   11.3   0.3     5    30   454   480   451   481 0.90 HAT (Half-A-TPR) repeatHAT                  PF02184.12    32 augustus_masked-scaffold_7-processed-gene-0.357-mRNA-1 -            685   3.4e-25   87.6  74.4  13  15      0.28   2.1e+02    1.8   0.0     3    11   486   494   484   498 0.87 HAT (Half-A-TPR) repeatHAT                  PF02184.12    32 augustus_masked-scaffold_7-processed-gene-0.357-mRNA-1 -            685   3.4e-25   87.6  74.4  14  15   4.1e-07   0.00031   20.5   0.2     2    30   521   549   520   551 0.96 HAT (Half-A-TPR) repeatHAT                  PF02184.12    32 augustus_masked-scaffold_7-processed-gene-0.357-mRNA-1 -            685   3.4e-25   87.6  74.4  15  15     0.033        26    4.8   0.4     4    12   585   593   582   594 0.92 HAT (Half-A-TPR) repeatTPR_19               PF14559.2     68 augustus_masked-scaffold_7-processed-gene-0.357-mRNA-1 -            685     2e-21   76.1   1.0   1   7   1.7e-08   1.3e-05   25.4   0.0     2    54    83   135    82   145 0.93 Tetratricopeptide repeatTPR_19               PF14559.2     68 augustus_masked-scaffold_7-processed-gene-0.357-mRNA-1 -            685     2e-21   76.1   1.0   2   7      0.22   1.7e+02    2.7   0.0    11    48   160   196   152   199 0.74 Tetratricopeptide repeatTPR_19               PF14559.2     68 augustus_masked-scaffold_7-processed-gene-0.357-mRNA-1 -            685     2e-21   76.1   1.0   3   7   0.00046      0.35   11.2   0.1    13    62   228   279   218   284 0.83 Tetratricopeptide repeatTPR_19               PF14559.2     68 augustus_masked-scaffold_7-processed-gene-0.357-mRNA-1 -            685     2e-21   76.1   1.0   4   7    0.0062       4.8    7.6   0.0    12    56   308   352   307   357 0.90 Tetratricopeptide repeatTPR_19               PF14559.2     68 augustus_masked-scaffold_7-processed-gene-0.357-mRNA-1 -            685     2e-21   76.1   1.0   5   7   1.6e-05     0.012   15.9   0.0     5    52   379   430   376   439 0.92 Tetratricopeptide repeatTPR_19               PF14559.2     68 augustus_masked-scaffold_7-processed-gene-0.357-mRNA-1 -            685     2e-21   76.1   1.0   6   7      0.19   1.5e+02    2.9   0.0    18    56   463   501   456   508 0.80 Tetratricopeptide repeatTPR_19               PF14559.2     68 augustus_masked-scaffold_7-processed-gene-0.357-mRNA-1 -            685     2e-21   76.1   1.0   7   7       2.4   1.8e+03   -0.7   0.0     3    28   518   542   517   552 0.71 Tetratricopeptide repeatSuf                  PF05843.10   296 augustus_masked-scaffold_7-processed-gene-0.357-mRNA-1 -            685   3.4e-21   76.3  10.5   1   5     2e-07   0.00016   21.6   0.3    24   136    61   189    53   200 0.84 Suppressor of forked protein (Suf)Suf                  PF05843.10   296 augustus_masked-scaffold_7-processed-gene-0.357-mRNA-1 -            685   3.4e-21   76.3  10.5   2   5   1.8e-07   0.00014   21.8   1.0     4   129   176   301   175   328 0.71 Suppressor of forked protein (Suf)Suf                  PF05843.10   296 augustus_masked-scaffold_7-processed-gene-0.357-mRNA-1 -            685   3.4e-21   76.3  10.5   3   5     0.033        26    4.5   0.0    85   143   380   439   332   445 0.80 Suppressor of forked protein (Suf)Suf                  PF05843.10   296 augustus_masked-scaffold_7-processed-gene-0.357-mRNA-1 -            685   3.4e-21   76.3  10.5   4   5   5.7e-07   0.00044   20.2   0.2    83   135   483   534   479   583 0.84 Suppressor of forked protein (Suf)Suf                  PF05843.10   296 augustus_masked-scaffold_7-processed-gene-0.357-mRNA-1 -            685   3.4e-21   76.3  10.5   5   5    0.0005      0.39   10.5   0.1    86   139   584   640   578   655 0.84 Suppressor of forked protein (Suf)TPR_16               PF13432.2     65 augustus_masked-scaffold_7-processed-gene-0.357-mRNA-1 -            685   2.5e-19   69.5  23.6   1   7   2.7e-05     0.021   15.6   0.0    18    64    59   105    58   106 0.93 Tetratricopeptide repeatTPR_16               PF13432.2     65 augustus_masked-scaffold_7-processed-gene-0.357-mRNA-1 -            685   2.5e-19   69.5  23.6   2   7    0.0026         2    9.2   0.0     5    57   114   166   110   178 0.90 Tetratricopeptide repeatTPR_16               PF13432.2     65 augustus_masked-scaffold_7-processed-gene-0.357-mRNA-1 -            685   2.5e-19   69.5  23.6   3   7      0.18   1.4e+02    3.4   0.0    11    43   187   218   178   239 0.81 Tetratricopeptide repeatTPR_16               PF13432.2     65 augustus_masked-scaffold_7-processed-gene-0.357-mRNA-1 -            685   2.5e-19   69.5  23.6   4   7   3.1e-05     0.024   15.4   0.6     2    38   248   284   247   300 0.84 Tetratricopeptide repeatTPR_16               PF13432.2     65 augustus_masked-scaffold_7-processed-gene-0.357-mRNA-1 -            685   2.5e-19   69.5  23.6   5   7   3.1e-05     0.024   15.4   0.2    17    62   307   352   307   355 0.94 Tetratricopeptide repeatTPR_16               PF13432.2     65 augustus_masked-scaffold_7-processed-gene-0.357-mRNA-1 -            685   2.5e-19   69.5  23.6   6   7    0.0026         2    9.3   0.0    10    63   378   435   372   437 0.73 Tetratricopeptide repeatTPR_16               PF13432.2     65 augustus_masked-scaffold_7-processed-gene-0.357-mRNA-1 -            685   2.5e-19   69.5  23.6   7   7   0.00044      0.34   11.7   0.4     4    61   477   536   474   539 0.85 Tetratricopeptide repeatTPR_2                PF07719.13    34 augustus_masked-scaffold_7-processed-gene-0.357-mRNA-1 -            685   1.8e-08   33.7  15.5   1   9    0.0001     0.078   13.0   0.0     4    33    75   104    73   105 0.92 Tetratricopeptide repeatTPR_2                PF07719.13    34 augustus_masked-scaffold_7-processed-gene-0.357-mRNA-1 -            685   1.8e-08   33.7  15.5   2   9       1.2   8.9e+02    0.3   0.0    11    32   116   137   114   138 0.84 Tetratricopeptide repeatTPR_2                PF07719.13    34 augustus_masked-scaffold_7-processed-gene-0.357-mRNA-1 -            685   1.8e-08   33.7  15.5   3   9    0.0042       3.3    7.9   0.9     8    33   250   275   244   276 0.84 Tetratricopeptide repeatTPR_2                PF07719.13    34 augustus_masked-scaffold_7-processed-gene-0.357-mRNA-1 -            685   1.8e-08   33.7  15.5   4   9      0.51   3.9e+02    1.4   0.0     2    32   322   352   321   353 0.85 Tetratricopeptide repeatTPR_2                PF07719.13    34 augustus_masked-scaffold_7-processed-gene-0.357-mRNA-1 -            685   1.8e-08   33.7  15.5   5   9       8.1   6.3e+03   -2.3   0.0    17    32   381   396   377   397 0.78 Tetratricopeptide repeatTPR_2                PF07719.13    34 augustus_masked-scaffold_7-processed-gene-0.357-mRNA-1 -            685   1.8e-08   33.7  15.5   6   9    0.0025       1.9    8.6   0.0     1    34   403   436   403   436 0.92 Tetratricopeptide repeatTPR_2                PF07719.13    34 augustus_masked-scaffold_7-processed-gene-0.357-mRNA-1 -            685   1.8e-08   33.7  15.5   7   9      0.29   2.3e+02    2.2   0.0     4    28   473   497   470   499 0.84 Tetratricopeptide repeatTPR_2                PF07719.13    34 augustus_masked-scaffold_7-processed-gene-0.357-mRNA-1 -            685   1.8e-08   33.7  15.5   8   9         4   3.1e+03   -1.4   0.1    14    30   519   535   517   537 0.83 Tetratricopeptide repeatTPR_2                PF07719.13    34 augustus_masked-scaffold_7-processed-gene-0.357-mRNA-1 -            685   1.8e-08   33.7  15.5   9   9       1.9   1.4e+03   -0.3   0.1    18    29   585   596   581   600 0.83 Tetratricopeptide repeatTPR_6                PF13174.2     33 augustus_masked-scaffold_7-processed-gene-0.357-mRNA-1 -            685   2.9e-08   33.7  17.2   1   8   0.00087      0.67   10.6   0.0     6    33    78   105    76   105 0.92 Tetratricopeptide repeatTPR_6                PF13174.2     33 augustus_masked-scaffold_7-processed-gene-0.357-mRNA-1 -            685   2.9e-08   33.7  17.2   2   8     0.075        58    4.5   0.0    12    31   181   204   173   205 0.76 Tetratricopeptide repeatTPR_6                PF13174.2     33 augustus_masked-scaffold_7-processed-gene-0.357-mRNA-1 -            685   2.9e-08   33.7  17.2   3   8     0.026        20    6.0   0.2     7    33   248   276   245   276 0.80 Tetratricopeptide repeatTPR_6                PF13174.2     33 augustus_masked-scaffold_7-processed-gene-0.357-mRNA-1 -            685   2.9e-08   33.7  17.2   4   8      0.15   1.1e+02    3.6   0.0    13    26   378   391   369   393 0.82 Tetratricopeptide repeatTPR_6                PF13174.2     33 augustus_masked-scaffold_7-processed-gene-0.357-mRNA-1 -            685   2.9e-08   33.7  17.2   5   8      0.42   3.2e+02    2.2   0.0     6    27   409   430   407   435 0.87 Tetratricopeptide repeatTPR_6                PF13174.2     33 augustus_masked-scaffold_7-processed-gene-0.357-mRNA-1 -            685   2.9e-08   33.7  17.2   6   8      0.38   2.9e+02    2.3   0.0     9    28   479   498   475   502 0.78 Tetratricopeptide repeatTPR_6                PF13174.2     33 augustus_masked-scaffold_7-processed-gene-0.357-mRNA-1 -            685   2.9e-08   33.7  17.2   7   8     0.021        16    6.3   0.1    12    29   518   535   507   535 0.75 Tetratricopeptide repeatTPR_6                PF13174.2     33 augustus_masked-scaffold_7-processed-gene-0.357-mRNA-1 -            685   2.9e-08   33.7  17.2   8   8       1.1   8.7e+02    0.8   0.0    16    27   584   595   577   597 0.83 Tetratricopeptide repeatTPR_17               PF13431.2     34 augustus_masked-scaffold_7-processed-gene-0.357-mRNA-1 -            685   8.4e-08   31.9   4.8   1   6     0.014        11    6.6   0.0     2    19    95   112    94   126 0.88 Tetratricopeptide repeatTPR_17               PF13431.2     34 augustus_masked-scaffold_7-processed-gene-0.357-mRNA-1 -            685   8.4e-08   31.9   4.8   2   6      0.39     3e+02    2.0   0.1    17    34   247   264   244   264 0.87 Tetratricopeptide repeatTPR_17               PF13431.2     34 augustus_masked-scaffold_7-processed-gene-0.357-mRNA-1 -            685   8.4e-08   31.9   4.8   3   6    0.0051         4    7.9   0.0     2    31   310   339   309   342 0.87 Tetratricopeptide repeatTPR_17               PF13431.2     34 augustus_masked-scaffold_7-processed-gene-0.357-mRNA-1 -            685   8.4e-08   31.9   4.8   4   6       1.1   8.9e+02    0.6   0.0    12    34   402   424   401   424 0.89 Tetratricopeptide repeatTPR_17               PF13431.2     34 augustus_masked-scaffold_7-processed-gene-0.357-mRNA-1 -            685   8.4e-08   31.9   4.8   5   6    0.0034       2.6    8.5   0.0     1    34   458   491   458   491 0.95 Tetratricopeptide repeatTPR_17               PF13431.2     34 augustus_masked-scaffold_7-processed-gene-0.357-mRNA-1 -            685   8.4e-08   31.9   4.8   6   6       1.6   1.3e+03    0.1   0.0     1    22   528   548   528   558 0.84 Tetratricopeptide repeatTPR_8                PF13181.2     34 augustus_masked-scaffold_7-processed-gene-0.357-mRNA-1 -            685   4.4e-07   29.4   0.0   1   5     0.035        27    5.1   0.0     8    33    79   104    72   105 0.78 Tetratricopeptide repeatTPR_8                PF13181.2     34 augustus_masked-scaffold_7-processed-gene-0.357-mRNA-1 -            685   4.4e-07   29.4   0.0   2   5      0.01       7.9    6.7   0.2     9    29   251   271   244   275 0.83 Tetratricopeptide repeatTPR_8                PF13181.2     34 augustus_masked-scaffold_7-processed-gene-0.357-mRNA-1 -            685   4.4e-07   29.4   0.0   3   5       2.9   2.3e+03   -0.9   0.0    16    30   380   394   379   397 0.85 Tetratricopeptide repeatTPR_8                PF13181.2     34 augustus_masked-scaffold_7-processed-gene-0.357-mRNA-1 -            685   4.4e-07   29.4   0.0   4   5      0.17   1.3e+02    2.9   0.0     1    34   403   436   403   436 0.93 Tetratricopeptide repeatTPR_8                PF13181.2     34 augustus_masked-scaffold_7-processed-gene-0.357-mRNA-1 -            685   4.4e-07   29.4   0.0   5   5     0.015        11    6.2   0.0     2    29   471   498   470   499 0.92 Tetratricopeptide repeatTPR_15               PF13429.2    280 augustus_masked-scaffold_7-processed-gene-0.357-mRNA-1 -            685   5.3e-07   29.0  15.4   1   7    0.0012      0.95    8.5   0.5   149   219    75   145    61   157 0.81 Tetratricopeptide repeatTPR_15               PF13429.2    280 augustus_masked-scaffold_7-processed-gene-0.357-mRNA-1 -            685   5.3e-07   29.0  15.4   2   7     0.013        10    5.1   0.1   112   171   142   198   135   211 0.81 Tetratricopeptide repeatTPR_15               PF13429.2    280 augustus_masked-scaffold_7-processed-gene-0.357-mRNA-1 -            685   5.3e-07   29.0  15.4   3   7    0.0022       1.7    7.6   0.1   144   181   241   278   205   285 0.78 Tetratricopeptide repeatTPR_15               PF13429.2    280 augustus_masked-scaffold_7-processed-gene-0.357-mRNA-1 -            685   5.3e-07   29.0  15.4   4   7     0.055        42    3.1   0.2   144   182   319   357   275   371 0.58 Tetratricopeptide repeatTPR_15               PF13429.2    280 augustus_masked-scaffold_7-processed-gene-0.357-mRNA-1 -            685   5.3e-07   29.0  15.4   5   7     0.088        68    2.4   0.7   125   190   336   408   320   426 0.58 Tetratricopeptide repeatTPR_15               PF13429.2    280 augustus_masked-scaffold_7-processed-gene-0.357-mRNA-1 -            685   5.3e-07   29.0  15.4   6   7     0.051        39    3.2   0.0   146   181   403   438   400   443 0.89 Tetratricopeptide repeatTPR_15               PF13429.2    280 augustus_masked-scaffold_7-processed-gene-0.357-mRNA-1 -            685   5.3e-07   29.0  15.4   7   7    0.0036       2.8    6.9   0.0   113   176   473   536   468   544 0.88 Tetratricopeptide repeatTPR_12               PF13424.2     79 augustus_masked-scaffold_7-processed-gene-0.357-mRNA-1 -            685   6.3e-07   29.5  19.2   1   7    0.0015       1.2    9.4   0.1     7    41    74   103    68   138 0.67 Tetratricopeptide repeatTPR_12               PF13424.2     79 augustus_masked-scaffold_7-processed-gene-0.357-mRNA-1 -            685   6.3e-07   29.5  19.2   2   7    0.0015       1.1    9.4   0.3    11    35   249   273   240   286 0.69 Tetratricopeptide repeatTPR_12               PF13424.2     79 augustus_masked-scaffold_7-processed-gene-0.357-mRNA-1 -            685   6.3e-07   29.5  19.2   3   7       2.4   1.9e+03   -0.9   0.0    50    75   324   349   313   359 0.70 Tetratricopeptide repeatTPR_12               PF13424.2     79 augustus_masked-scaffold_7-processed-gene-0.357-mRNA-1 -            685   6.3e-07   29.5  19.2   4   7     0.012         9    6.6   0.0    45    78   401   434   377   435 0.67 Tetratricopeptide repeatTPR_12               PF13424.2     79 augustus_masked-scaffold_7-processed-gene-0.357-mRNA-1 -            685   6.3e-07   29.5  19.2   5   7     0.014        10    6.4   0.7     4    73   469   532   466   534 0.65 Tetratricopeptide repeatTPR_12               PF13424.2     79 augustus_masked-scaffold_7-processed-gene-0.357-mRNA-1 -            685   6.3e-07   29.5  19.2   6   7     0.084        65    3.8   0.2    13    35   514   536   501   547 0.80 Tetratricopeptide repeatTPR_12               PF13424.2     79 augustus_masked-scaffold_7-processed-gene-0.357-mRNA-1 -            685   6.3e-07   29.5  19.2   7   7      0.93   7.2e+02    0.5   0.0    22    36   585   600   580   627 0.65 Tetratricopeptide repeatTPR_11               PF13414.2     68 augustus_masked-scaffold_7-processed-gene-0.357-mRNA-1 -            685   1.7e-06   27.9   9.5   1   7   7.5e-05     0.058   13.4   0.0     5    64    74   133    71   155 0.88 TPR repeatTPR_11               PF13414.2     68 augustus_masked-scaffold_7-processed-gene-0.357-mRNA-1 -            685   1.7e-06   27.9   9.5   2   7    0.0059       4.5    7.3   0.3    10    36   250   276   231   289 0.78 TPR repeatTPR_11               PF13414.2     68 augustus_masked-scaffold_7-processed-gene-0.357-mRNA-1 -            685   1.7e-06   27.9   9.5   3   7       2.6     2e+03   -1.2   0.1    25    65   309   349   307   352 0.73 TPR repeatTPR_11               PF13414.2     68 augustus_masked-scaffold_7-processed-gene-0.357-mRNA-1 -            685   1.7e-06   27.9   9.5   4   7      0.03        23    5.1   0.0    20    68   382   434   375   446 0.71 TPR repeatTPR_11               PF13414.2     68 augustus_masked-scaffold_7-processed-gene-0.357-mRNA-1 -            685   1.7e-06   27.9   9.5   5   7       2.2   1.7e+03   -0.9   0.0    26    65   459   498   456   503 0.72 TPR repeatTPR_11               PF13414.2     68 augustus_masked-scaffold_7-processed-gene-0.357-mRNA-1 -            685   1.7e-06   27.9   9.5   6   7      0.59   4.5e+02    0.9   0.1    16    65   483   534   468   537 0.60 TPR repeatTPR_11               PF13414.2     68 augustus_masked-scaffold_7-processed-gene-0.357-mRNA-1 -            685   1.7e-06   27.9   9.5   7   7      0.36   2.8e+02    1.6   0.0    14    44   517   546   506   560 0.79 TPR repeatTPR_7                PF13176.2     36 augustus_masked-scaffold_7-processed-gene-0.357-mRNA-1 -            685   0.00033   20.3  11.4   1   7     0.055        42    4.3   0.0    11    32    84   103    79   107 0.75 Tetratricopeptide repeatTPR_7                PF13176.2     36 augustus_masked-scaffold_7-processed-gene-0.357-mRNA-1 -            685   0.00033   20.3  11.4   2   7    0.0012      0.94    9.5   0.2     7    33   251   275   246   278 0.76 Tetratricopeptide repeatTPR_7                PF13176.2     36 augustus_masked-scaffold_7-processed-gene-0.357-mRNA-1 -            685   0.00033   20.3  11.4   3   7       1.2   9.1e+02    0.2   0.0    11    29   333   349   328   358 0.74 Tetratricopeptide repeatTPR_7                PF13176.2     36 augustus_masked-scaffold_7-processed-gene-0.357-mRNA-1 -            685   0.00033   20.3  11.4   4   7      0.18   1.4e+02    2.7   0.0     4    29   408   433   405   440 0.84 Tetratricopeptide repeatTPR_7                PF13176.2     36 augustus_masked-scaffold_7-processed-gene-0.357-mRNA-1 -            685   0.00033   20.3  11.4   5   7       5.9   4.6e+03   -2.0   0.0    10    32   481   501   476   504 0.72 Tetratricopeptide repeatTPR_7                PF13176.2     36 augustus_masked-scaffold_7-processed-gene-0.357-mRNA-1 -            685   0.00033   20.3  11.4   6   7      0.13     1e+02    3.2   0.1    10    29   517   536   514   558 0.85 Tetratricopeptide repeatTPR_7                PF13176.2     36 augustus_masked-scaffold_7-processed-gene-0.357-mRNA-1 -            685   0.00033   20.3  11.4   7   7       8.3   6.4e+03   -2.5   0.0     7    15   593   601   585   604 0.62 Tetratricopeptide repeatNRDE-2               PF08424.6    323 augustus_masked-scaffold_7-processed-gene-0.357-mRNA-1 -            685    0.0022   17.0  25.1   1   5    0.0017       1.3    7.9   1.2    85   116    90   121    24   139 0.80 NRDE-2, necessary for RNA interferenceNRDE-2               PF08424.6    323 augustus_masked-scaffold_7-processed-gene-0.357-mRNA-1 -            685    0.0022   17.0  25.1   2   5    0.0014         1    8.2   2.5    52   108    93   147    56   169 0.64 NRDE-2, necessary for RNA interferenceNRDE-2               PF08424.6    323 augustus_masked-scaffold_7-processed-gene-0.357-mRNA-1 -            685    0.0022   17.0  25.1   3   5    0.0016       1.2    8.0   0.2    47   140   259   357   171   363 0.80 NRDE-2, necessary for RNA interferenceNRDE-2               PF08424.6    323 augustus_masked-scaffold_7-processed-gene-0.357-mRNA-1 -            685    0.0022   17.0  25.1   4   5   0.00017      0.13   11.2   0.3     6   112   459   550   456   551 0.87 NRDE-2, necessary for RNA interferenceNRDE-2               PF08424.6    323 augustus_masked-scaffold_7-processed-gene-0.357-mRNA-1 -            685    0.0022   17.0  25.1   5   5     0.048        37    3.1   5.4     2    79   524   620   523   660 0.63 NRDE-2, necessary for RNA interferenceTPR_1                PF00515.24    34 augustus_masked-scaffold_7-processed-gene-0.357-mRNA-1 -            685    0.0042   16.7   3.0   1   5     0.014        11    5.9   0.0    13    33    84   104    83   105 0.93 Tetratricopeptide repeatTPR_1                PF00515.24    34 augustus_masked-scaffold_7-processed-gene-0.357-mRNA-1 -            685    0.0042   16.7   3.0   2   5     0.044        34    4.3   0.2    13    32   255   274   253   276 0.90 Tetratricopeptide repeatTPR_1                PF00515.24    34 augustus_masked-scaffold_7-processed-gene-0.357-mRNA-1 -            685    0.0042   16.7   3.0   3   5      0.17   1.3e+02    2.5   0.0     1    34   403   436   403   436 0.88 Tetratricopeptide repeatTPR_1                PF00515.24    34 augustus_masked-scaffold_7-processed-gene-0.357-mRNA-1 -            685    0.0042   16.7   3.0   4   5         5   3.8e+03   -2.2   0.0    23    34   458   469   451   469 0.83 Tetratricopeptide repeatTPR_1                PF00515.24    34 augustus_masked-scaffold_7-processed-gene-0.357-mRNA-1 -            685    0.0042   16.7   3.0   5   5       5.7   4.4e+03   -2.3   0.1    14    28   519   533   517   534 0.83 Tetratricopeptide repeatU3_assoc_6           PF08640.7     83 augustus_masked-scaffold_7-processed-gene-0.357-mRNA-1 -            685    0.0053   16.7  19.1   1   7   0.00012     0.093   12.7   0.7    27    63    56    92    47    94 0.91 U3 small nucleolar RNA-associated protein 6U3_assoc_6           PF08640.7     83 augustus_masked-scaffold_7-processed-gene-0.357-mRNA-1 -            685    0.0053   16.7  19.1   2   7     0.029        23    5.0   0.0    21    58    84   121    83   128 0.87 U3 small nucleolar RNA-associated protein 6U3_assoc_6           PF08640.7     83 augustus_masked-scaffold_7-processed-gene-0.357-mRNA-1 -            685    0.0053   16.7  19.1   3   7      0.48   3.7e+02    1.2   0.2    45    65   245   265   240   287 0.82 U3 small nucleolar RNA-associated protein 6U3_assoc_6           PF08640.7     83 augustus_masked-scaffold_7-processed-gene-0.357-mRNA-1 -            685    0.0053   16.7  19.1   4   7      0.26     2e+02    2.0   0.1    25    53   303   331   288   364 0.87 U3 small nucleolar RNA-associated protein 6U3_assoc_6           PF08640.7     83 augustus_masked-scaffold_7-processed-gene-0.357-mRNA-1 -            685    0.0053   16.7  19.1   5   7      0.59   4.6e+02    0.9   0.1    45    65   438   458   433   466 0.82 U3 small nucleolar RNA-associated protein 6U3_assoc_6           PF08640.7     83 augustus_masked-scaffold_7-processed-gene-0.357-mRNA-1 -            685    0.0053   16.7  19.1   6   7     0.058        45    4.1   0.0    25    63   452   490   450   493 0.92 U3 small nucleolar RNA-associated protein 6U3_assoc_6           PF08640.7     83 augustus_masked-scaffold_7-processed-gene-0.357-mRNA-1 -            685    0.0053   16.7  19.1   7   7      0.53   4.1e+02    1.0   0.5    34    56   530   552   522   572 0.84 U3 small nucleolar RNA-associated protein 6Mad3_BUB1_I          PF08311.8    124 augustus_masked-scaffold_7-processed-gene-0.357-mRNA-1 -            685    0.0085   15.9  14.9   1   7      0.11        89    3.0   0.2   100   123    75    98    57    99 0.76 Mad3/BUB1 homology region 1Mad3_BUB1_I          PF08311.8    124 augustus_masked-scaffold_7-processed-gene-0.357-mRNA-1 -            685    0.0085   15.9  14.9   2   7       1.6   1.2e+03   -0.7   0.0    18    51   104   133    90   151 0.59 Mad3/BUB1 homology region 1Mad3_BUB1_I          PF08311.8    124 augustus_masked-scaffold_7-processed-gene-0.357-mRNA-1 -            685    0.0085   15.9  14.9   3   7     0.045        35    4.3   0.1    98   123   244   269   208   270 0.92 Mad3/BUB1 homology region 1Mad3_BUB1_I          PF08311.8    124 augustus_masked-scaffold_7-processed-gene-0.357-mRNA-1 -            685    0.0085   15.9  14.9   4   7    0.0021       1.6    8.6   0.4     3   124   307   430   305   430 0.75 Mad3/BUB1 homology region 1Mad3_BUB1_I          PF08311.8    124 augustus_masked-scaffold_7-processed-gene-0.357-mRNA-1 -            685    0.0085   15.9  14.9   5   7     0.057        44    3.9   0.0    68   121   471   530   459   533 0.89 Mad3/BUB1 homology region 1Mad3_BUB1_I          PF08311.8    124 augustus_masked-scaffold_7-processed-gene-0.357-mRNA-1 -            685    0.0085   15.9  14.9   6   7       2.2   1.7e+03   -1.2   0.0    66    76   538   548   530   557 0.76 Mad3/BUB1 homology region 1Mad3_BUB1_I          PF08311.8    124 augustus_masked-scaffold_7-processed-gene-0.357-mRNA-1 -            685    0.0085   15.9  14.9   7   7       2.9   2.3e+03   -1.6   0.0    37    82   581   626   574   631 0.78 Mad3/BUB1 homology region 1Fis1_TPR_C           PF14853.2     53 augustus_masked-scaffold_7-processed-gene-0.357-mRNA-1 -            685     0.032   14.2   5.6   1   6      0.11        88    3.2   0.0    14    33    85   104    83   105 0.93 Fis1 C-terminal tetratricopeptide repeatFis1_TPR_C           PF14853.2     53 augustus_masked-scaffold_7-processed-gene-0.357-mRNA-1 -            685     0.032   14.2   5.6   2   6      0.98   7.6e+02    0.2   0.0    13    33   185   205   183   209 0.86 Fis1 C-terminal tetratricopeptide repeatFis1_TPR_C           PF14853.2     53 augustus_masked-scaffold_7-processed-gene-0.357-mRNA-1 -            685     0.032   14.2   5.6   3   6     0.018        14    5.7   0.2    12    35   254   277   246   284 0.82 Fis1 C-terminal tetratricopeptide repeatFis1_TPR_C           PF14853.2     53 augustus_masked-scaffold_7-processed-gene-0.357-mRNA-1 -            685     0.032   14.2   5.6   4   6       4.8   3.7e+03   -2.0   0.0    12    28   414   430   413   437 0.78 Fis1 C-terminal tetratricopeptide repeatFis1_TPR_C           PF14853.2     53 augustus_masked-scaffold_7-processed-gene-0.357-mRNA-1 -            685     0.032   14.2   5.6   5   6       6.4     5e+03   -2.4   0.0    13    36   448   471   447   472 0.90 Fis1 C-terminal tetratricopeptide repeatFis1_TPR_C           PF14853.2     53 augustus_masked-scaffold_7-processed-gene-0.357-mRNA-1 -            685     0.032   14.2   5.6   6   6      0.39     3e+02    1.4   0.1    15    30   520   535   519   538 0.93 Fis1 C-terminal tetratricopeptide repeatTPR_9                PF13371.2     73 augustus_masked-scaffold_7-processed-gene-0.357-mRNA-1 -            685     0.057   13.3   9.1   1   3    0.0063       4.9    7.2   0.3     4    60    81   137    79   141 0.85 Tetratricopeptide repeatTPR_9                PF13371.2     73 augustus_masked-scaffold_7-processed-gene-0.357-mRNA-1 -            685     0.057   13.3   9.1   2   3       0.8   6.2e+02    0.4   0.2    36    63   250   277   245   284 0.82 Tetratricopeptide repeatTPR_9                PF13371.2     73 augustus_masked-scaffold_7-processed-gene-0.357-mRNA-1 -            685     0.057   13.3   9.1   3   3    0.0039         3    7.9   0.0    10    63   380   437   376   443 0.83 Tetratricopeptide repeatLegionella_OMP       PF05150.8    288 augustus_masked-scaffold_7-processed-gene-0.357-mRNA-1 -            685      0.13   11.5   1.7   1   1   0.00039       0.3   10.3   1.7    63   165   158   262   139   272 0.88 Legionella pneumophila major outer membrane protein precursorCDC45                PF02724.10   627 augustus_masked-scaffold_7-processed-gene-0.357-mRNA-1 -            685       7.9    4.5  10.9   1   1      0.02        15    3.5  10.9   113   194   519   629   511   657 0.44 CDC45-like proteinHis_biosynth         PF00977.17   229 maker-scaffold_7-augustus-gene-0.1332-mRNA-1 -            552   2.1e-47  161.4   0.4   1   1   3.2e-50   8.7e-47  159.3   0.4     1   229   234   530   234   530 0.87 Histidine biosynthesis proteinGATase               PF00117.24   191 maker-scaffold_7-augustus-gene-0.1332-mRNA-1 -            552   1.9e-19   70.0   0.0   1   1   1.3e-22   3.5e-19   69.2   0.0     2   176     6   193     5   204 0.83 Glutamine amidotransferase class-ISNO                  PF01174.15   188 maker-scaffold_7-augustus-gene-0.1332-mRNA-1 -            552   6.9e-08   32.4   0.0   1   2   1.1e-09   2.9e-06   27.1   0.0     5    99    11   106     8   117 0.85 SNO glutamine amidotransferase familySNO                  PF01174.15   188 maker-scaffold_7-augustus-gene-0.1332-mRNA-1 -            552   6.9e-08   32.4   0.0   2   2      0.03        80    2.9   0.0   153   172   172   192   156   204 0.77 SNO glutamine amidotransferase familyGATase_3             PF07685.10   159 maker-scaffold_7-augustus-gene-0.1332-mRNA-1 -            552   1.1e-05   25.1   0.1   1   1   1.1e-08   3.1e-05   23.6   0.1     4    81    36   106    33   189 0.78 CobB/CobQ-like glutamine amidotransferase domainPeptidase_C26        PF07722.9    216 maker-scaffold_7-augustus-gene-0.1332-mRNA-1 -            552     0.056   13.1   0.1   1   2   0.00025      0.68    9.5   0.0    96   118    65    87    60    91 0.89 Peptidase C26Peptidase_C26        PF07722.9    216 maker-scaffold_7-augustus-gene-0.1332-mRNA-1 -            552     0.056   13.1   0.1   2   2       0.1   2.8e+02    0.9   0.0   204   216   178   190   161   190 0.84 Peptidase C26Dus                  PF01207.13   310 maker-scaffold_7-augustus-gene-0.1332-mRNA-1 -            552      0.12   11.3   0.0   1   2      0.37   9.9e+02   -1.6   0.0   177   197   312   335   276   364 0.59 Dihydrouridine synthase (Dus)Dus                  PF01207.13   310 maker-scaffold_7-augustus-gene-0.1332-mRNA-1 -            552      0.12   11.3   0.0   2   2   0.00015       0.4    9.6   0.0   138   209   447   517   436   541 0.83 Dihydrouridine synthase (Dus)Ribosomal_L50        PF10501.5    109 maker-scaffold_7-augustus-gene-0.1329-mRNA-1 -            399   5.3e-10   39.3   0.0   1   1     6e-14   9.8e-10   38.5   0.0    13   108   265   351   245   352 0.73 Ribosomal subunit 39SPeptidase_S8         PF00082.18   285 augustus_masked-scaffold_7-processed-gene-0.494-mRNA-1 -            539   1.1e-39  136.2   4.2   1   1   2.8e-43   1.5e-39  135.7   4.2     1   272   185   436   185   455 0.88 Subtilase familyInhibitor_I9         PF05922.12    82 augustus_masked-scaffold_7-processed-gene-0.494-mRNA-1 -            539   6.4e-21   74.6   1.0   1   2   6.9e-24   3.7e-20   72.2   0.2     1    82    42   135    42   135 0.94 Peptidase inhibitor I9Inhibitor_I9         PF05922.12    82 augustus_masked-scaffold_7-processed-gene-0.494-mRNA-1 -            539   6.4e-21   74.6   1.0   2   2      0.21   1.2e+03    0.1   0.0    51    72   469   490   462   490 0.89 Peptidase inhibitor I9Phasin               PF05597.7    126 augustus_masked-scaffold_7-processed-gene-0.494-mRNA-1 -            539     0.055   13.4   0.1   1   1   2.4e-05      0.13   12.2   0.1    54   101   492   539   456   539 0.75 Poly(hydroxyalcanoate) granule associated protein (phasin)Acetate_kinase       PF00871.13   390 augustus_masked-scaffold_7-processed-gene-0.356-mRNA-1 -            427   3.9e-89  298.9   0.0   1   1     3e-93   4.8e-89  298.6   0.0     2   369     5   385     4   413 0.86 Acetokinase familyDUF1620              PF07774.9    202 augustus_masked-scaffold_7-processed-gene-0.474-mRNA-1 -            980   6.4e-68  228.3   0.0   1   1   2.1e-71   1.1e-67  227.5   0.0     1   202   746   976   746   976 0.97 Protein of unknown function (DUF1620)PQQ_2                PF13360.2    238 augustus_masked-scaffold_7-processed-gene-0.474-mRNA-1 -            980   4.6e-15   55.8  10.3   1   2   2.4e-16   1.3e-12   47.8   3.6     3   236    65   327    62   329 0.78 PQQ-like domainPQQ_2                PF13360.2    238 augustus_masked-scaffold_7-processed-gene-0.474-mRNA-1 -            980   4.6e-15   55.8  10.3   2   2   4.3e-05      0.23   11.0   0.5     6   101   523   659   500   743 0.68 PQQ-like domainPQQ                  PF01011.17    38 augustus_masked-scaffold_7-processed-gene-0.474-mRNA-1 -            980   7.6e-05   22.3   2.2   1   6     0.012        65    3.6   0.0     4    30    59    85    57    87 0.85 PQQ enzyme repeatPQQ                  PF01011.17    38 augustus_masked-scaffold_7-processed-gene-0.474-mRNA-1 -            980   7.6e-05   22.3   2.2   2   6     0.076   4.1e+02    1.1   0.0    20    32   175   187   175   192 0.82 PQQ enzyme repeatPQQ                  PF01011.17    38 augustus_masked-scaffold_7-processed-gene-0.474-mRNA-1 -            980   7.6e-05   22.3   2.2   3   6     0.047   2.5e+02    1.7   0.0     7    21   210   224   208   226 0.81 PQQ enzyme repeatPQQ                  PF01011.17    38 augustus_masked-scaffold_7-processed-gene-0.474-mRNA-1 -            980   7.6e-05   22.3   2.2   4   6      0.57   3.1e+03   -1.7   0.0    16    30   319   333   318   334 0.90 PQQ enzyme repeatPQQ                  PF01011.17    38 augustus_masked-scaffold_7-processed-gene-0.474-mRNA-1 -            980   7.6e-05   22.3   2.2   5   6   0.00044       2.4    8.2   0.2     2    25   511   536   510   537 0.86 PQQ enzyme repeatPQQ                  PF01011.17    38 augustus_masked-scaffold_7-processed-gene-0.474-mRNA-1 -            980   7.6e-05   22.3   2.2   6   6      0.41   2.2e+03   -1.2   0.0    15    24   575   584   573   591 0.84 PQQ enzyme repeatp450                 PF00067.18   463 maker-scaffold_7-snap-gene-0.686-mRNA-1 -            533   1.3e-62  211.9   0.0   1   1   2.8e-66   2.2e-62  211.2   0.0    41   437    92   499    83   513 0.86 Cytochrome P450TDH                  PF03347.9    165 maker-scaffold_7-snap-gene-0.686-mRNA-1 -            533    0.0037   16.7   0.0   1   1   8.8e-07    0.0071   15.8   0.0    26    69   105   147    91   164 0.86 Vibrio thermostable direct hemolysinArm                  PF00514.19    41 maker-scaffold_7-augustus-gene-0.1327-mRNA-1 -            687    0.0043   16.9   2.4   1   2   0.00059       4.8    7.2   0.1    21    41   322   342   314   342 0.82 Armadillo/beta-catenin-like repeatArm                  PF00514.19    41 maker-scaffold_7-augustus-gene-0.1327-mRNA-1 -            687    0.0043   16.9   2.4   2   2   0.00035       2.9    7.9   0.3    14    41   457   485   456   485 0.93 Armadillo/beta-catenin-like repeatHEAT_2               PF13646.2     88 maker-scaffold_7-augustus-gene-0.1327-mRNA-1 -            687     0.013   15.8   2.4   1   3   7.5e-05      0.61   10.5   0.1    34    69   316   352   280   380 0.83 HEAT repeatsHEAT_2               PF13646.2     88 maker-scaffold_7-augustus-gene-0.1327-mRNA-1 -            687     0.013   15.8   2.4   2   3   0.00028       2.3    8.7   1.8    35    69   317   352   316   505 0.82 HEAT repeatsHEAT_2               PF13646.2     88 maker-scaffold_7-augustus-gene-0.1327-mRNA-1 -            687     0.013   15.8   2.4   3   3       1.2   9.5e+03   -2.9   0.0    26    37   564   575   531   586 0.44 HEAT repeatsDUF3464              PF11947.4    150 augustus_masked-scaffold_7-processed-gene-0.406-mRNA-1 -            592       2.3    7.9   7.1   1   1    0.0011       9.1    5.9   7.1     9    72   222   279   213   286 0.54 Protein of unknown function (DUF3464)Roughex              PF06020.7    365 augustus_masked-scaffold_7-processed-gene-0.406-mRNA-1 -            592       7.9    5.3  10.4   1   1    0.0017        14    4.5  10.4   270   302   215   248   199   273 0.64 Drosophila roughex proteinDUF3602              PF12223.4     81 maker-scaffold_7-augustus-gene-0.1293-mRNA-1 -            127   1.6e-21   76.6  11.5   1   2   8.9e-06      0.14   12.6   1.6    30    46     3    19     1    21 0.81 Protein of unknown function (DUF3602)DUF3602              PF12223.4     81 maker-scaffold_7-augustus-gene-0.1293-mRNA-1 -            127   1.6e-21   76.6  11.5   2   2   2.3e-25   3.8e-21   75.4   3.8     1    81    14    90    14    90 0.97 Protein of unknown function (DUF3602)ABC2_membrane        PF01061.20   210 augustus_masked-scaffold_7-processed-gene-0.416-mRNA-1 -           1517   3.5e-88  294.3  58.2   1   4   2.8e-45     2e-42  144.9  17.7     1   210   518   728   518   728 0.97 ABC-2 type transporterABC2_membrane        PF01061.20   210 augustus_masked-scaffold_7-processed-gene-0.416-mRNA-1 -           1517   3.5e-88  294.3  58.2   2   4       1.6   1.1e+03   -1.3   0.1    52    69   789   806   772   816 0.54 ABC-2 type transporterABC2_membrane        PF01061.20   210 augustus_masked-scaffold_7-processed-gene-0.416-mRNA-1 -           1517   3.5e-88  294.3  58.2   3   4     3e-51   2.1e-48  164.4  21.6     2   207  1194  1407  1193  1410 0.98 ABC-2 type transporterABC2_membrane        PF01061.20   210 augustus_masked-scaffold_7-processed-gene-0.416-mRNA-1 -           1517   3.5e-88  294.3  58.2   4   4       4.8   3.4e+03   -2.9   0.7    15   144  1480  1500  1469  1506 0.44 ABC-2 type transporterPDR_CDR              PF06422.8     98 augustus_masked-scaffold_7-processed-gene-0.416-mRNA-1 -           1517   3.2e-35  119.8   6.6   1   2     5e-33   3.5e-30  103.6   0.0     3    93   741   831   739   836 0.95 CDR ABC transporterPDR_CDR              PF06422.8     98 augustus_masked-scaffold_7-processed-gene-0.416-mRNA-1 -           1517   3.2e-35  119.8   6.6   2   2   2.3e-06    0.0016   18.1   0.8    32    76  1465  1508  1461  1514 0.87 CDR ABC transporterABC_tran             PF00005.23   137 augustus_masked-scaffold_7-processed-gene-0.416-mRNA-1 -           1517   2.3e-33  115.5   0.0   1   3   3.2e-16   2.2e-13   50.7   0.0     6   136   197   352   192   353 0.82 ABC transporterABC_tran             PF00005.23   137 augustus_masked-scaffold_7-processed-gene-0.416-mRNA-1 -           1517   2.3e-33  115.5   0.0   2   3       1.9   1.3e+03   -0.4   0.0    88   121   494   526   448   532 0.73 ABC transporterABC_tran             PF00005.23   137 augustus_masked-scaffold_7-processed-gene-0.416-mRNA-1 -           1517   2.3e-33  115.5   0.0   3   3   3.1e-19   2.2e-16   60.5   0.0     1   137   893  1044   893  1044 0.91 ABC transporterABC_trans_N          PF14510.2     85 augustus_masked-scaffold_7-processed-gene-0.416-mRNA-1 -           1517   1.1e-20   73.5   0.0   1   1   3.3e-23   2.3e-20   72.4   0.0     1    84    87   170    87   171 0.88 ABC-transporter extracellular N-terminalAAA_16               PF13191.2    177 augustus_masked-scaffold_7-processed-gene-0.416-mRNA-1 -           1517   6.5e-09   36.4   0.3   1   2     0.007       4.9    7.5   0.0     4    46   183   224   183   310 0.72 AAA ATPase domainAAA_16               PF13191.2    177 augustus_masked-scaffold_7-processed-gene-0.416-mRNA-1 -           1517   6.5e-09   36.4   0.3   2   2   1.8e-08   1.3e-05   25.6   0.3     8   169   888  1065   886  1075 0.56 AAA ATPase domainABC2_membrane_3      PF12698.3    345 augustus_masked-scaffold_7-processed-gene-0.416-mRNA-1 -           1517   8.2e-07   28.3   1.2   1   3      0.12        87    1.9   0.2   164   190   790   816   774   820 0.88 ABC-2 family transporter proteinABC2_membrane_3      PF12698.3    345 augustus_masked-scaffold_7-processed-gene-0.416-mRNA-1 -           1517   8.2e-07   28.3   1.2   2   3       4.2     3e+03   -3.1   4.6   251   296  1219  1266  1211  1270 0.60 ABC-2 family transporter proteinABC2_membrane_3      PF12698.3    345 augustus_masked-scaffold_7-processed-gene-0.416-mRNA-1 -           1517   8.2e-07   28.3   1.2   3   3   1.2e-09   8.2e-07   28.3   1.2   208   344  1295  1498  1272  1501 0.82 ABC-2 family transporter proteinAAA_25               PF13481.2    194 augustus_masked-scaffold_7-processed-gene-0.416-mRNA-1 -           1517   1.7e-06   27.7   0.1   1   2    0.0028         2    7.9   0.0    27    57   196   226   184   309 0.88 AAA domainAAA_25               PF13481.2    194 augustus_masked-scaffold_7-processed-gene-0.416-mRNA-1 -           1517   1.7e-06   27.7   0.1   2   2   5.6e-06     0.004   16.7   0.2    20    78   890   955   876  1075 0.73 AAA domainAAA_33               PF13671.2    143 augustus_masked-scaffold_7-processed-gene-0.416-mRNA-1 -           1517   9.8e-06   25.6   0.0   1   2     0.002       1.4    8.9   0.0     2    35   205   238   204   302 0.84 AAA domainAAA_33               PF13671.2    143 augustus_masked-scaffold_7-processed-gene-0.416-mRNA-1 -           1517   9.8e-06   25.6   0.0   2   2   5.6e-05      0.04   13.9   0.0     2    78   906   979   905  1012 0.73 AAA domainDUF258               PF03193.12   167 augustus_masked-scaffold_7-processed-gene-0.416-mRNA-1 -           1517   1.3e-05   25.0   0.0   1   2     0.065        46    3.7   0.0    99   123   201   226   187   254 0.84 Protein of unknown function, DUF258DUF258               PF03193.12   167 augustus_masked-scaffold_7-processed-gene-0.416-mRNA-1 -           1517   1.3e-05   25.0   0.0   2   2   1.7e-06    0.0012   18.6   0.0    77   123   881   947   846   975 0.70 Protein of unknown function, DUF258AAA_29               PF13555.2     61 augustus_masked-scaffold_7-processed-gene-0.416-mRNA-1 -           1517   0.00021   20.8   0.8   1   2     0.033        24    4.6   0.0    22    40   201   220   195   224 0.82 P-loop containing region of AAA domainAAA_29               PF13555.2     61 augustus_masked-scaffold_7-processed-gene-0.416-mRNA-1 -           1517   0.00021   20.8   0.8   2   2   4.3e-05      0.03   13.9   0.1    23    42   903   923   894   925 0.84 P-loop containing region of AAA domainAAA_22               PF13401.2    137 augustus_masked-scaffold_7-processed-gene-0.416-mRNA-1 -           1517    0.0014   18.8   0.1   1   2     0.052        37    4.4   0.0     4    26   201   223   199   251 0.89 AAA domainAAA_22               PF13401.2    137 augustus_masked-scaffold_7-processed-gene-0.416-mRNA-1 -           1517    0.0014   18.8   0.1   2   2   0.00049      0.34   11.0   0.1     5    41   903   930   900   970 0.84 AAA domainNACHT                PF05729.8    166 augustus_masked-scaffold_7-processed-gene-0.416-mRNA-1 -           1517    0.0023   17.8   0.0   1   2      0.02        14    5.4   0.0     2    25   204   227   203   239 0.83 NACHT domainNACHT                PF05729.8    166 augustus_masked-scaffold_7-processed-gene-0.416-mRNA-1 -           1517    0.0023   17.8   0.0   2   2    0.0012      0.86    9.4   0.1     3    31   906   934   904   946 0.87 NACHT domainAAA_19               PF13245.2     76 augustus_masked-scaffold_7-processed-gene-0.416-mRNA-1 -           1517    0.0069   16.2   0.3   1   2    0.0076       5.4    6.9   0.0    10    32   202   223   197   246 0.82 Part of AAA domainAAA_19               PF13245.2     76 augustus_masked-scaffold_7-processed-gene-0.416-mRNA-1 -           1517    0.0069   16.2   0.3   2   2     0.013       8.9    6.2   0.2    11    35   904   927   896   945 0.82 Part of AAA domainAAA_17               PF13207.2    122 augustus_masked-scaffold_7-processed-gene-0.416-mRNA-1 -           1517    0.0078   17.2   0.0   1   2     0.049        35    5.4   0.0     3    30   206   237   204   313 0.78 AAA domainAAA_17               PF13207.2    122 augustus_masked-scaffold_7-processed-gene-0.416-mRNA-1 -           1517    0.0078   17.2   0.0   2   2    0.0053       3.7    8.5   0.0     4    64   908   994   906  1054 0.63 AAA domaincobW                 PF02492.15   177 augustus_masked-scaffold_7-processed-gene-0.416-mRNA-1 -           1517    0.0079   15.7   0.3   1   2      0.18   1.3e+02    1.9   0.0     3    21   205   223   203   231 0.84 CobW/HypB/UreG, nucleotide-binding domaincobW                 PF02492.15   177 augustus_masked-scaffold_7-processed-gene-0.416-mRNA-1 -           1517    0.0079   15.7   0.3   2   2   0.00021      0.15   11.5   0.0     3    37   906   937   904   957 0.83 CobW/HypB/UreG, nucleotide-binding domainAAA_21               PF13304.2    303 augustus_masked-scaffold_7-processed-gene-0.416-mRNA-1 -           1517    0.0094   16.0   0.0   1   2    0.0023       1.6    8.7   0.0     1    20   905   924   905   940 0.91 AAA domain, putative AbiEii toxin, Type IV TA systemAAA_21               PF13304.2    303 augustus_masked-scaffold_7-processed-gene-0.416-mRNA-1 -           1517    0.0094   16.0   0.0   2   2      0.04        28    4.6   0.0   259   296  1035  1071  1019  1076 0.80 AAA domain, putative AbiEii toxin, Type IV TA systemAAA_18               PF13238.2    129 augustus_masked-scaffold_7-processed-gene-0.416-mRNA-1 -           1517     0.011   16.1   0.0   1   2      0.11        76    3.7   0.0     3    31   207   236   205   262 0.76 AAA domainAAA_18               PF13238.2    129 augustus_masked-scaffold_7-processed-gene-0.416-mRNA-1 -           1517     0.011   16.1   0.0   2   2    0.0019       1.3    9.4   0.0     3    32   908   939   907   963 0.79 AAA domainAAA                  PF00004.25   132 augustus_masked-scaffold_7-processed-gene-0.416-mRNA-1 -           1517     0.016   15.5   0.0   1   2    0.0041       2.9    8.2   0.0     2    41   206   254   205   269 0.77 ATPase family associated with various cellular activities (AAA)AAA                  PF00004.25   132 augustus_masked-scaffold_7-processed-gene-0.416-mRNA-1 -           1517     0.016   15.5   0.0   2   2     0.064        45    4.3   0.0     3    26   908   931   906   948 0.87 ATPase family associated with various cellular activities (AAA)AAA_23               PF13476.2    200 augustus_masked-scaffold_7-processed-gene-0.416-mRNA-1 -           1517     0.078   13.3   0.1   1   1   0.00033      0.24   11.8   0.1    21    39   905   923   898   926 0.90 AAA domainHalX                 PF08663.6     69 augustus_masked-scaffold_7-processed-gene-0.416-mRNA-1 -           1517     0.092   12.9   3.0   1   2     0.032        23    5.2   0.4    31    49   464   482   461   484 0.86 HalX domainHalX                 PF08663.6     69 augustus_masked-scaffold_7-processed-gene-0.416-mRNA-1 -           1517     0.092   12.9   3.0   2   2     0.007       4.9    7.4   0.1    32    50  1151  1169  1147  1185 0.84 HalX domainSMC_N                PF02463.15   220 augustus_masked-scaffold_7-processed-gene-0.416-mRNA-1 -           1517      0.12   11.7   0.0   1   3       1.1   7.6e+02   -0.8   0.0    26    44   204   222   199   276 0.88 RecF/RecN/SMC N terminal domainSMC_N                PF02463.15   220 augustus_masked-scaffold_7-processed-gene-0.416-mRNA-1 -           1517      0.12   11.7   0.0   2   3      0.21   1.5e+02    1.5   0.0    25    44   904   923   892   924 0.85 RecF/RecN/SMC N terminal domainSMC_N                PF02463.15   220 augustus_masked-scaffold_7-processed-gene-0.416-mRNA-1 -           1517      0.12   11.7   0.0   3   3     0.013       8.9    5.5   0.0   157   204  1032  1079  1023  1092 0.83 RecF/RecN/SMC N terminal domainAAA_10               PF12846.3    304 augustus_masked-scaffold_7-processed-gene-0.416-mRNA-1 -           1517      0.25   10.8   1.5   1   2     0.011       7.9    5.9   0.2     4    23   205   224   202   228 0.88 AAA-like domainAAA_10               PF12846.3    304 augustus_masked-scaffold_7-processed-gene-0.416-mRNA-1 -           1517      0.25   10.8   1.5   2   2     0.066        47    3.4   0.1     4    23   906   925   903   930 0.84 AAA-like domainBLOC1S3              PF15753.1    172 augustus_masked-scaffold_7-processed-gene-0.416-mRNA-1 -           1517         1    9.4   3.8   1   1    0.0033       2.3    8.2   3.8     7    52    27    76    24   103 0.75 Biogenesis of lysosome-related organelles complex 1 subunit 3Thiolase_N           PF00108.19   260 augustus_masked-scaffold_7-processed-gene-0.455-mRNA-1 -            419   2.2e-76  256.5   3.1   1   1   8.7e-80   3.5e-76  255.9   3.1     1   260    34   289    34   289 0.96 Thiolase, N-terminal domainThiolase_C           PF02803.14   123 augustus_masked-scaffold_7-processed-gene-0.455-mRNA-1 -            419   1.6e-41  140.7   0.1   1   1   8.5e-45   3.4e-41  139.6   0.1     4   120   299   415   296   418 0.96 Thiolase, C-terminal domainketoacyl-synt        PF00109.22   253 augustus_masked-scaffold_7-processed-gene-0.455-mRNA-1 -            419    0.0024   17.3   2.5   1   2   4.9e-06      0.02   14.4   0.7   174   223   118   168   108   176 0.81 Beta-ketoacyl synthase, N-terminal domainketoacyl-synt        PF00109.22   253 augustus_masked-scaffold_7-processed-gene-0.455-mRNA-1 -            419    0.0024   17.3   2.5   2   2     0.036   1.5e+02    1.7   0.0   234   253   272   291   248   291 0.73 Beta-ketoacyl synthase, N-terminal domainACP_syn_III_C        PF08541.6     90 augustus_masked-scaffold_7-processed-gene-0.455-mRNA-1 -            419     0.012   15.6   0.0   1   1     9e-06     0.036   14.1   0.0     1    83   327   414   327   418 0.87 3-Oxoacyl-[acyl-carrier-protein (ACP)] synthase III C terminalSHS2_Rpb7-N          PF03876.13    70 maker-scaffold_7-snap-gene-0.698-mRNA-1 -            144   1.2e-15   57.5   0.0   1   1   2.3e-19   1.8e-15   56.9   0.0     2    70     9    76     8    76 0.98 SHS2 domain found in N terminus of Rpb7p/Rpc25p/MJ0397S1                   PF00575.19    75 maker-scaffold_7-snap-gene-0.698-mRNA-1 -            144      0.15   12.3   0.0   1   1     3e-05      0.25   11.5   0.0    40    68    92   126    81   129 0.79 S1 RNA binding domainDHDPS                PF00701.18   289 maker-scaffold_7-augustus-gene-0.1343-mRNA-1 -            326     1e-24   86.6   0.0   1   1   1.8e-28   1.5e-24   86.1   0.0    22   237    35   260    26   301 0.81 Dihydrodipicolinate synthetase familyDUF818               PF05677.8    365 maker-scaffold_7-augustus-gene-0.1343-mRNA-1 -            326      0.14   10.9   0.1   1   1   3.4e-05      0.27   10.0   0.1   161   183   136   158   126   163 0.86 Chlamydia CHLPS protein (DUF818)F-box-like           PF12937.3     47 maker-scaffold_7-augustus-gene-0.1365-mRNA-1 -            960     0.087   12.6   0.1   1   2   5.3e-05      0.29   10.9   0.0     5    41   667   704   664   706 0.87 F-box-likeF-box-like           PF12937.3     47 maker-scaffold_7-augustus-gene-0.1365-mRNA-1 -            960     0.087   12.6   0.1   2   2       1.6   8.5e+03   -3.4   0.1    38    47   795   804   793   804 0.77 F-box-likeTFIIF_alpha          PF05793.8    528 maker-scaffold_7-augustus-gene-0.1365-mRNA-1 -            960      0.19   10.1   6.1   1   1   5.3e-05      0.29    9.6   6.1   349   488   764   910   712   916 0.63 Transcription initiation factor IIF, alpha subunit (TFIIF-alpha)CENP-B_dimeris       PF09026.6    100 maker-scaffold_7-augustus-gene-0.1365-mRNA-1 -            960       8.5    6.7  12.5   1   2   7.1e-05      0.38   11.0   6.3    16    50   761   797   751   809 0.66 Centromere protein B dimerisation domainCENP-B_dimeris       PF09026.6    100 maker-scaffold_7-augustus-gene-0.1365-mRNA-1 -            960       8.5    6.7  12.5   2   2       2.9   1.6e+04   -3.8   0.4    16    22   864   870   857   886 0.45 Centromere protein B dimerisation domainRCC1                 PF00415.14    50 maker-scaffold_7-augustus-gene-0.1307-mRNA-1 -           1669   7.8e-22   77.5   1.5   1   5   0.00055       1.5    9.4   0.0     3    31   243   271   242   276 0.92 Regulator of chromosome condensation (RCC1) repeatRCC1                 PF00415.14    50 maker-scaffold_7-augustus-gene-0.1307-mRNA-1 -           1669   7.8e-22   77.5   1.5   2   5   1.8e-09   4.9e-06   26.9   0.1     4    50   335   382   333   382 0.86 Regulator of chromosome condensation (RCC1) repeatRCC1                 PF00415.14    50 maker-scaffold_7-augustus-gene-0.1307-mRNA-1 -           1669   7.8e-22   77.5   1.5   3   5   6.4e-08   0.00017   22.0   0.0     1    49   385   442   385   443 0.79 Regulator of chromosome condensation (RCC1) repeatRCC1                 PF00415.14    50 maker-scaffold_7-augustus-gene-0.1307-mRNA-1 -           1669   7.8e-22   77.5   1.5   4   5   2.3e-05     0.062   13.8   0.0     4    49   448   498   446   499 0.76 Regulator of chromosome condensation (RCC1) repeatRCC1                 PF00415.14    50 maker-scaffold_7-augustus-gene-0.1307-mRNA-1 -           1669   7.8e-22   77.5   1.5   5   5       1.8   4.9e+03   -1.9   0.0    26    49   591   624   579   625 0.59 Regulator of chromosome condensation (RCC1) repeatBTB                  PF00651.27   110 maker-scaffold_7-augustus-gene-0.1307-mRNA-1 -           1669   8.8e-11   41.9   0.0   1   3     1e-05     0.028   14.5   0.0    11    87   776   869   771   883 0.88 BTB/POZ domainBTB                  PF00651.27   110 maker-scaffold_7-augustus-gene-0.1307-mRNA-1 -           1669   8.8e-11   41.9   0.0   2   3       3.8     1e+04   -3.4   0.0    87   108   895   916   894   918 0.89 BTB/POZ domainBTB                  PF00651.27   110 maker-scaffold_7-augustus-gene-0.1307-mRNA-1 -           1669   8.8e-11   41.9   0.0   3   3     1e-08   2.8e-05   24.1   0.0    12   103   928  1042   922  1046 0.89 BTB/POZ domainAnk_4                PF13637.2     54 maker-scaffold_7-augustus-gene-0.1307-mRNA-1 -           1669     2e-07   31.4   0.2   1   2       1.9   5.2e+03   -1.8   0.0    24    40    87   103    86   116 0.79 Ankyrin repeats (many copies)Ank_4                PF13637.2     54 maker-scaffold_7-augustus-gene-0.1307-mRNA-1 -           1669     2e-07   31.4   0.2   2   2   4.3e-10   1.2e-06   29.0   0.1    17    54   116   155   101   155 0.91 Ankyrin repeats (many copies)RCC1_2               PF13540.2     30 maker-scaffold_7-augustus-gene-0.1307-mRNA-1 -           1669   2.3e-07   30.3   7.5   1   4      0.33     9e+02   -0.3   0.0    16    27   240   251   239   252 0.84 Regulator of chromosome condensation (RCC1) repeatRCC1_2               PF13540.2     30 maker-scaffold_7-augustus-gene-0.1307-mRNA-1 -           1669   2.3e-07   30.3   7.5   2   4   2.2e-09     6e-06   25.8   0.3     2    30   370   398   369   398 0.94 Regulator of chromosome condensation (RCC1) repeatRCC1_2               PF13540.2     30 maker-scaffold_7-augustus-gene-0.1307-mRNA-1 -           1669   2.3e-07   30.3   7.5   3   4    0.0039        10    5.9   0.1    19    27   447   455   433   457 0.83 Regulator of chromosome condensation (RCC1) repeatRCC1_2               PF13540.2     30 maker-scaffold_7-augustus-gene-0.1307-mRNA-1 -           1669   2.3e-07   30.3   7.5   4   4       2.6   6.9e+03   -3.1   0.0     5    21   549   565   548   566 0.82 Regulator of chromosome condensation (RCC1) repeatAnk_2                PF12796.3     86 maker-scaffold_7-augustus-gene-0.1307-mRNA-1 -           1669   0.00085   20.0   0.1   1   1   8.1e-07    0.0022   18.7   0.1    22    78    90   157    81   164 0.72 Ankyrin repeats (3 copies)Ank_5                PF13857.2     56 maker-scaffold_7-augustus-gene-0.1307-mRNA-1 -           1669     0.037   14.4   0.2   1   2   0.00012      0.33   11.3   0.1    15    39   134   159   119   163 0.73 Ankyrin repeats (many copies)Ank_5                PF13857.2     56 maker-scaffold_7-augustus-gene-0.1307-mRNA-1 -           1669     0.037   14.4   0.2   2   2       1.4   3.7e+03   -1.5   0.0    42    55   177   190   174   191 0.85 Ankyrin repeats (many copies)BPL_N                PF09825.5    377 maker-scaffold_7-snap-gene-0.738-mRNA-1 -            668  4.3e-133  443.9   0.0   1   1  6.7e-137  5.4e-133  443.6   0.0     1   377     6   383     6   383 0.93 Biotin-protein ligase, N terminalBPL_LplA_LipB        PF03099.15   131 maker-scaffold_7-snap-gene-0.738-mRNA-1 -            668   1.8e-20   73.0   0.0   1   1   3.4e-24   2.7e-20   72.5   0.0     1   131   402   544   402   544 0.96 Biotin/lipoate A/B protein ligase familyGln-synt_C           PF00120.20   345 augustus_masked-scaffold_7-processed-gene-0.486-mRNA-1 -            478   5.8e-83  278.6   0.0   1   1   1.3e-86   6.9e-83  278.3   0.0     2   345   118   474   117   474 0.91 Glutamine synthetase, catalytic domainGST_N                PF02798.16    76 augustus_masked-scaffold_7-processed-gene-0.486-mRNA-1 -            478      0.04   14.1   0.0   1   1   1.6e-05     0.085   13.1   0.0    23    64   139   183   134   185 0.79 Glutathione S-transferase, N-terminal domainDUF1201              PF06716.7     54 augustus_masked-scaffold_7-processed-gene-0.486-mRNA-1 -            478     0.094   12.6   0.0   1   2   9.7e-05      0.53   10.2   0.0     2    46    29    76    28    81 0.81 Protein of unknown function (DUF1201)DUF1201              PF06716.7     54 augustus_masked-scaffold_7-processed-gene-0.486-mRNA-1 -            478     0.094   12.6   0.0   2   2      0.24   1.3e+03   -0.7   0.0    36    48   237   249   234   252 0.90 Protein of unknown function (DUF1201)Sugar_tr             PF00083.20   452 maker-scaffold_7-augustus-gene-0.1353-mRNA-1 -            509   2.9e-98  329.5  24.4   1   1  4.1e-102   3.3e-98  329.3  24.4     1   452    11   467    11   467 0.95 Sugar (and other) transporterMFS_1                PF07690.12   352 maker-scaffold_7-augustus-gene-0.1353-mRNA-1 -            509   1.8e-34  119.1  27.9   1   2   6.6e-29   5.4e-25   87.9   6.2     4   218    18   273     7   295 0.75 Major Facilitator SuperfamilyMFS_1                PF07690.12   352 maker-scaffold_7-augustus-gene-0.1353-mRNA-1 -            509   1.8e-34  119.1  27.9   2   2   1.8e-13   1.5e-09   37.2  13.8    30   176   298   456   278   466 0.78 Major Facilitator SuperfamilyDynamin_M            PF01031.16   284 maker-scaffold_7-augustus-gene-0.1269-mRNA-1 -            803  9.8e-109  362.8   0.0   1   1  6.8e-112  1.8e-108  361.9   0.0     1   283   236   522   236   523 0.97 Dynamin central regionDynamin_N            PF00350.19   168 maker-scaffold_7-augustus-gene-0.1269-mRNA-1 -            803   3.1e-57  193.2   0.0   1   3   1.1e-60   3.1e-57  193.2   0.0     1   168    33   227    33   227 0.94 Dynamin familyDynamin_N            PF00350.19   168 maker-scaffold_7-augustus-gene-0.1269-mRNA-1 -            803   3.1e-57  193.2   0.0   2   3       4.2   1.1e+04   -3.9   0.4    53    70   537   554   518   585 0.46 Dynamin familyDynamin_N            PF00350.19   168 maker-scaffold_7-augustus-gene-0.1269-mRNA-1 -            803   3.1e-57  193.2   0.0   3   3       3.8     1e+04   -3.8   0.1    48    80   756   790   738   797 0.64 Dynamin familyGED                  PF02212.14    92 maker-scaffold_7-augustus-gene-0.1269-mRNA-1 -            803   2.1e-30  104.6   2.5   1   1   2.3e-33   6.3e-30  103.1   2.5     1    92   711   802   711   802 0.98 Dynamin GTPase effector domainMMR_HSR1             PF01926.19   110 maker-scaffold_7-augustus-gene-0.1269-mRNA-1 -            803   6.8e-05   22.8   0.1   1   1   2.7e-07   0.00074   19.5   0.1     1    84    32   195    32   226 0.74 50S ribosome-binding GTPaseRibosomal_L11        PF00298.15    70 maker-scaffold_7-augustus-gene-0.1269-mRNA-1 -            803     0.048   14.1   0.2   1   1   4.5e-05      0.12   12.8   0.2    17    58   559   603   547   606 0.73 Ribosomal protein L11, RNA binding domainAtrophin-1           PF03154.11   982 maker-scaffold_7-augustus-gene-0.1269-mRNA-1 -            803     0.054   11.7   2.0   1   1   3.1e-05     0.083   11.1   2.0    73   181   520   618   511   689 0.68 Atrophin-1 familyPyr_redox_3          PF13738.2    214 maker-scaffold_7-augustus-gene-0.1346-mRNA-1 -            631   2.5e-18   66.8   0.0   1   1     7e-21   4.3e-18   66.0   0.0     1   212   214   416   214   418 0.81 Pyridine nucleotide-disulphide oxidoreductasePyr_redox_2          PF07992.10   292 maker-scaffold_7-augustus-gene-0.1346-mRNA-1 -            631   9.6e-18   64.1   0.0   1   2   5.9e-17   3.7e-14   52.4   0.0     3   178   213   421   211   437 0.68 Pyridine nucleotide-disulphide oxidoreductasePyr_redox_2          PF07992.10   292 maker-scaffold_7-augustus-gene-0.1346-mRNA-1 -            631   9.6e-18   64.1   0.0   2   2   0.00081       0.5    9.3   0.0   192   236   516   555   503   573 0.80 Pyridine nucleotide-disulphide oxidoreductaseFMO-like             PF00743.15   532 maker-scaffold_7-augustus-gene-0.1346-mRNA-1 -            631   3.6e-10   38.7   0.1   1   2   1.5e-11   9.1e-09   34.1   0.0    70   221   269   420   211   427 0.84 Flavin-binding monooxygenase-likeFMO-like             PF00743.15   532 maker-scaffold_7-augustus-gene-0.1346-mRNA-1 -            631   3.6e-10   38.7   0.1   2   2     0.066        42    2.2   0.0   292   331   515   555   454   559 0.87 Flavin-binding monooxygenase-likeK_oxygenase          PF13434.2    341 maker-scaffold_7-augustus-gene-0.1346-mRNA-1 -            631   8.1e-09   34.9   0.2   1   3       0.2   1.3e+02    1.4   0.0   187   203   207   223   160   246 0.80 L-lysine 6-monooxygenase (NADPH-requiring)K_oxygenase          PF13434.2    341 maker-scaffold_7-augustus-gene-0.1346-mRNA-1 -            631   8.1e-09   34.9   0.2   2   3   6.1e-09   3.8e-06   26.1   0.1   115   230   303   420   279   432 0.75 L-lysine 6-monooxygenase (NADPH-requiring)K_oxygenase          PF13434.2    341 maker-scaffold_7-augustus-gene-0.1346-mRNA-1 -            631   8.1e-09   34.9   0.2   3   3     0.048        30    3.4   0.0   326   340   541   555   521   556 0.88 L-lysine 6-monooxygenase (NADPH-requiring)SnoaL_2              PF12680.3    102 maker-scaffold_7-augustus-gene-0.1346-mRNA-1 -            631   9.6e-06   26.1   0.2   1   2   1.1e-07   6.7e-05   23.3   0.0     1    65    43   111    43   156 0.80 SnoaL-like domainSnoaL_2              PF12680.3    102 maker-scaffold_7-augustus-gene-0.1346-mRNA-1 -            631   9.6e-06   26.1   0.2   2   2       2.9   1.8e+03   -0.5   0.0    48    81   313   346   274   364 0.76 SnoaL-like domainNAD_binding_8        PF13450.2     68 maker-scaffold_7-augustus-gene-0.1346-mRNA-1 -            631   1.4e-05   25.1   0.0   1   2   1.4e-07     9e-05   22.5   0.0     1    36   215   250   215   277 0.87 NAD(P)-binding Rossmann-like domainNAD_binding_8        PF13450.2     68 maker-scaffold_7-augustus-gene-0.1346-mRNA-1 -            631   1.4e-05   25.1   0.0   2   2        10   6.2e+03   -2.6   0.0    12    28   394   414   387   418 0.61 NAD(P)-binding Rossmann-like domainGIDA                 PF01134.18   392 maker-scaffold_7-augustus-gene-0.1346-mRNA-1 -            631   6.9e-05   22.0   0.0   1   2   1.7e-05     0.011   14.7   0.0     2    31   213   242   212   253 0.89 Glucose inhibited division protein AGIDA                 PF01134.18   392 maker-scaffold_7-augustus-gene-0.1346-mRNA-1 -            631   6.9e-05   22.0   0.0   2   2     0.017        10    4.9   0.0   109   150   517   554   462   572 0.86 Glucose inhibited division protein AHI0933_like          PF03486.10   409 maker-scaffold_7-augustus-gene-0.1346-mRNA-1 -            631   8.4e-05   21.3   0.1   1   2   1.5e-06   0.00096   17.8   0.0     2    37   212   247   211   250 0.94 HI0933-like proteinHI0933_like          PF03486.10   409 maker-scaffold_7-augustus-gene-0.1346-mRNA-1 -            631   8.4e-05   21.3   0.1   2   2      0.19   1.2e+02    1.0   0.0   121   165   516   555   511   561 0.75 HI0933-like proteinFAD_binding_2        PF00890.20   417 maker-scaffold_7-augustus-gene-0.1346-mRNA-1 -            631   0.00013   21.0   0.3   1   2   1.4e-06   0.00088   18.3   0.1     2    35   213   246   212   249 0.94 FAD binding domainFAD_binding_2        PF00890.20   417 maker-scaffold_7-augustus-gene-0.1346-mRNA-1 -            631   0.00013   21.0   0.3   2   2      0.67   4.2e+02   -0.4   0.0   154   204   296   350   278   390 0.69 FAD binding domainPyr_redox            PF00070.23    81 maker-scaffold_7-augustus-gene-0.1346-mRNA-1 -            631   0.00015   22.1   0.3   1   2    0.0056       3.5    8.1   0.0     2    35   213   246   212   256 0.92 Pyridine nucleotide-disulphide oxidoreductasePyr_redox            PF00070.23    81 maker-scaffold_7-augustus-gene-0.1346-mRNA-1 -            631   0.00015   22.1   0.3   2   2   0.00076      0.47   10.9   0.0     1    34   384   417   384   437 0.89 Pyridine nucleotide-disulphide oxidoreductaseFAD_binding_3        PF01494.15   356 maker-scaffold_7-augustus-gene-0.1346-mRNA-1 -            631   0.00028   20.2   0.0   1   2     1e-06   0.00065   18.9   0.0     3    35   212   244   210   255 0.90 FAD binding domainFAD_binding_3        PF01494.15   356 maker-scaffold_7-augustus-gene-0.1346-mRNA-1 -            631   0.00028   20.2   0.0   2   2       8.6   5.3e+03   -3.8   0.0    16    42   397   423   385   425 0.71 FAD binding domainNAD_binding_9        PF13454.2    156 maker-scaffold_7-augustus-gene-0.1346-mRNA-1 -            631    0.0037   17.1   0.0   1   3    0.0056       3.5    7.4   0.0     2    45   215   252   214   261 0.82 FAD-NAD(P)-bindingNAD_binding_9        PF13454.2    156 maker-scaffold_7-augustus-gene-0.1346-mRNA-1 -            631    0.0037   17.1   0.0   2   3       1.4   8.7e+02   -0.3   0.0    95   155   284   347   277   348 0.51 FAD-NAD(P)-bindingNAD_binding_9        PF13454.2    156 maker-scaffold_7-augustus-gene-0.1346-mRNA-1 -            631    0.0037   17.1   0.0   3   3     0.023        14    5.5   0.0   136   155   535   554   521   555 0.89 FAD-NAD(P)-bindingDUF4440              PF14534.2    107 maker-scaffold_7-augustus-gene-0.1346-mRNA-1 -            631     0.005   17.1   0.0   1   1     2e-05     0.012   15.8   0.0     4   107    42   159    40   159 0.71 Domain of unknown function (DUF4440)AlaDh_PNT_C          PF01262.17   214 maker-scaffold_7-augustus-gene-0.1346-mRNA-1 -            631    0.0054   15.9   0.0   1   1   1.6e-05    0.0099   15.0   0.0    30    67   212   249   206   261 0.87 Alanine dehydrogenase/PNT, C-terminal domainShikimate_DH         PF01488.16   138 maker-scaffold_7-augustus-gene-0.1346-mRNA-1 -            631     0.013   15.4   0.0   1   2    0.0039       2.5    8.0   0.0     3    40   201   238   199   251 0.86 Shikimate / quinate 5-dehydrogenaseShikimate_DH         PF01488.16   138 maker-scaffold_7-augustus-gene-0.1346-mRNA-1 -            631     0.013   15.4   0.0   2   2     0.037        23    4.9   0.0     9    47   379   416   372   470 0.86 Shikimate / quinate 5-dehydrogenaseSnoaL_4              PF13577.2    127 maker-scaffold_7-augustus-gene-0.1346-mRNA-1 -            631     0.016   15.2   0.3   1   2   0.00014     0.089   12.8   0.1    10   124    40   160    34   163 0.71 SnoaL-like domainSnoaL_4              PF13577.2    127 maker-scaffold_7-augustus-gene-0.1346-mRNA-1 -            631     0.016   15.2   0.3   2   2       9.7     6e+03   -2.8   0.0    50   100   188   241   180   263 0.54 SnoaL-like domainThi4                 PF01946.13   235 maker-scaffold_7-augustus-gene-0.1346-mRNA-1 -            631     0.018   14.2   0.0   1   2   0.00062      0.39    9.8   0.0    16    46   209   238   197   259 0.78 Thi4 familyThi4                 PF01946.13   235 maker-scaffold_7-augustus-gene-0.1346-mRNA-1 -            631     0.018   14.2   0.0   2   2      0.17   1.1e+02    1.8   0.1   138   171   317   350   307   358 0.88 Thi4 familyDAO                  PF01266.20   336 maker-scaffold_7-augustus-gene-0.1346-mRNA-1 -            631     0.019   14.5   0.2   1   1   0.00022      0.14   11.6   0.2     2    35   213   247   212   568 0.93 FAD dependent oxidoreductase2-Hacid_dh_C         PF02826.15   178 maker-scaffold_7-augustus-gene-0.1346-mRNA-1 -            631      0.02   14.1   0.0   1   2   0.00014     0.085   12.1   0.0    21    73   193   247   184   260 0.77 D-isomer specific 2-hydroxyacid dehydrogenase, NAD binding domain2-Hacid_dh_C         PF02826.15   178 maker-scaffold_7-augustus-gene-0.1346-mRNA-1 -            631      0.02   14.1   0.0   2   2       1.8   1.1e+03   -1.4   0.0    33    71   379   417   368   435 0.79 D-isomer specific 2-hydroxyacid dehydrogenase, NAD binding domainTrkA_N               PF02254.14   116 maker-scaffold_7-augustus-gene-0.1346-mRNA-1 -            631     0.039   14.1   0.0   1   1   0.00017       0.1   12.7   0.0     1    48   213   260   213   265 0.89 TrkA-N domain3HCDH_N              PF02737.14   180 maker-scaffold_7-augustus-gene-0.1346-mRNA-1 -            631     0.041   13.6   0.0   1   2      0.14        88    2.8   0.0     2    42   213   253   212   259 0.86 3-hydroxyacyl-CoA dehydrogenase, NAD binding domain3HCDH_N              PF02737.14   180 maker-scaffold_7-augustus-gene-0.1346-mRNA-1 -            631     0.041   13.6   0.0   2   2    0.0023       1.4    8.6   0.0     3    53   386   436   384   458 0.84 3-hydroxyacyl-CoA dehydrogenase, NAD binding domainNAD_binding_7        PF13241.2    105 maker-scaffold_7-augustus-gene-0.1346-mRNA-1 -            631     0.076   13.3   0.0   1   2      0.12        72    3.8   0.0     9    39   212   242   205   297 0.86 Putative NAD(P)-bindingNAD_binding_7        PF13241.2    105 maker-scaffold_7-augustus-gene-0.1346-mRNA-1 -            631     0.076   13.3   0.0   2   2     0.011       6.8    7.1   0.0     4    51   379   427   378   472 0.73 Putative NAD(P)-bindingFAD_oxidored         PF12831.3    433 maker-scaffold_7-augustus-gene-0.1346-mRNA-1 -            631     0.087   12.0   0.0   1   1   0.00026      0.16   11.1   0.0     2    35   213   246   212   259 0.95 FAD dependent oxidoreductaseLycopene_cycl        PF05834.8    378 maker-scaffold_7-augustus-gene-0.1346-mRNA-1 -            631     0.092   11.7   0.0   1   1   0.00032       0.2   10.6   0.0     2    36   213   245   212   260 0.87 Lycopene cyclase proteinBig_3_4              PF13754.2     65 maker-scaffold_7-augustus-gene-0.1346-mRNA-1 -            631      0.23   11.8   1.3   1   2       4.3   2.7e+03   -1.2   0.0    18    27   150   159   131   167 0.81 Bacterial Ig-like domain (group 3)Big_3_4              PF13754.2     65 maker-scaffold_7-augustus-gene-0.1346-mRNA-1 -            631      0.23   11.8   1.3   2   2    0.0014       0.9    9.9   0.8    14    40   306   333   296   335 0.81 Bacterial Ig-like domain (group 3)SnoaL_3              PF13474.2    121 maker-scaffold_7-augustus-gene-0.1346-mRNA-1 -            631      0.26   11.4   1.3   1   2    0.0014      0.89    9.7   0.5     3    30    41    68    39    81 0.90 SnoaL-like domainSnoaL_3              PF13474.2    121 maker-scaffold_7-augustus-gene-0.1346-mRNA-1 -            631      0.26   11.4   1.3   2   2       3.5   2.2e+03   -1.2   0.0    94   111   142   159   119   161 0.76 SnoaL-like domainHLH                  PF00010.22    55 augustus_masked-scaffold_7-processed-gene-0.358-mRNA-1 -            543   2.6e-12   46.4   0.0   1   1   5.1e-16   8.3e-12   44.8   0.0     1    49   300   402   300   407 0.93 Helix-loop-helix DNA-binding domainATG13                PF10033.5    233 maker-scaffold_7-snap-gene-0.718-mRNA-1 -            960   1.1e-71  241.0   0.0   1   1     1e-75   1.7e-71  240.4   0.0     1   233    72   309    72   309 0.95 Autophagy-related protein 13Spermine_synth       PF01564.13   247 maker-scaffold_7-augustus-gene-0.1299-mRNA-1 -            293     1e-98  329.1   0.0   1   1  5.6e-102   1.3e-98  328.8   0.0     1   242    13   252    13   257 0.98 Spermine/spermidine synthaseMethyltransf_18      PF12847.3    110 maker-scaffold_7-augustus-gene-0.1299-mRNA-1 -            293   0.00025   21.7   0.0   1   1   2.5e-07   0.00058   20.5   0.0     3   105    89   197    87   201 0.80 Methyltransferase domainMethyltransf_31      PF13847.2    152 maker-scaffold_7-augustus-gene-0.1299-mRNA-1 -            293     0.001   18.8   0.0   1   1   7.9e-07    0.0018   17.9   0.0    10   127    94   228    85   266 0.74 Methyltransferase domainDUF43                PF01861.12   243 maker-scaffold_7-augustus-gene-0.1299-mRNA-1 -            293     0.013   14.6   0.0   1   1   8.7e-06      0.02   14.0   0.0    45   121    88   169    77   179 0.75 Protein of unknown function DUF43Methyltransf_26      PF13659.2    124 maker-scaffold_7-augustus-gene-0.1299-mRNA-1 -            293     0.019   15.5   0.0   1   1   1.4e-05     0.032   14.7   0.0     2   118    89   197    88   199 0.74 Methyltransferase domainMethyltransf_12      PF08242.8     99 maker-scaffold_7-augustus-gene-0.1299-mRNA-1 -            293     0.019   15.5   0.4   1   2       1.3     3e+03   -1.2   0.1    65    93    49    80    25    83 0.61 Methyltransferase domainMethyltransf_12      PF08242.8     99 maker-scaffold_7-augustus-gene-0.1299-mRNA-1 -            293     0.019   15.5   0.4   2   2     4e-05     0.094   13.3   0.0     1    71    92   165    92   169 0.72 Methyltransferase domainCHAP                 PF05257.12    83 maker-scaffold_7-augustus-gene-0.1299-mRNA-1 -            293     0.091   13.2   0.1   1   1   8.9e-05      0.21   12.1   0.1    27    73    20    62     9    99 0.75 CHAP domainNtox1                PF15500.2     96 augustus_masked-scaffold_7-processed-gene-0.496-mRNA-1 -            379     0.058   13.6   0.1   1   2       1.8   1.4e+04   -3.7   0.5    21    34    24    37    22    40 0.80 Putative RNase-like toxin, toxin_1Ntox1                PF15500.2     96 augustus_masked-scaffold_7-processed-gene-0.496-mRNA-1 -            379     0.058   13.6   0.1   2   2   7.1e-06     0.058   13.6   0.1    25    71   114   167   105   200 0.77 Putative RNase-like toxin, toxin_1SOG2                 PF10428.5    509 augustus_masked-scaffold_7-processed-gene-0.496-mRNA-1 -            379       1.3    8.1  18.0   1   1   0.00023       1.9    7.6  18.0   155   362   142   345    79   355 0.77 RAM signalling pathway proteinSyja_N               PF02383.14   261 augustus_masked-scaffold_7-processed-gene-0.338-mRNA-1 -           1319   4.5e-64  216.2   0.0   1   2   1.1e-67     9e-64  215.2   0.0     1   261   106   457   106   457 0.85 SacI homology domainSyja_N               PF02383.14   261 augustus_masked-scaffold_7-processed-gene-0.338-mRNA-1 -           1319   4.5e-64  216.2   0.0   2   2      0.41   3.3e+03   -3.0   0.0   133   147   837   852   835   854 0.81 SacI homology domainExo_endo_phos        PF03372.19   239 augustus_masked-scaffold_7-processed-gene-0.338-mRNA-1 -           1319   9.5e-23   81.2   0.0   1   1     2e-26   1.6e-22   80.4   0.0     1   239   674   959   674   959 0.75 Endonuclease/Exonuclease/phosphatase familyNUDIX                PF00293.24   135 augustus_masked-scaffold_7-processed-gene-0.491-mRNA-1 -            206     2e-17   63.2   0.0   1   1   1.9e-21   3.1e-17   62.6   0.0     4   120    57   177    55   184 0.80 NUDIX domainPkinase              PF00069.21   262 snap_masked-scaffold_7-processed-gene-0.883-mRNA-1 -           1004   4.8e-70  235.7   0.0   1   1   2.6e-73   7.1e-70  235.2   0.0     1   262    71   332    71   332 0.92 Protein kinase domainPkinase_Tyr          PF07714.13   260 snap_masked-scaffold_7-processed-gene-0.883-mRNA-1 -           1004   1.1e-39  136.1   0.0   1   1   6.1e-43   1.6e-39  135.6   0.0     3   258    73   328    71   329 0.88 Protein tyrosine kinaseKinase-like          PF14531.2    288 snap_masked-scaffold_7-processed-gene-0.883-mRNA-1 -           1004   5.8e-09   35.4   0.0   1   2    0.0037       9.9    5.2   0.0    16    46    73   103    63   127 0.86 Kinase-likeKinase-like          PF14531.2    288 snap_masked-scaffold_7-processed-gene-0.883-mRNA-1 -           1004   5.8e-09   35.4   0.0   2   2   4.1e-10   1.1e-06   28.0   0.0   132   259   155   282   146   320 0.72 Kinase-likePOLO_box             PF00659.14    60 snap_masked-scaffold_7-processed-gene-0.883-mRNA-1 -           1004   1.6e-05   24.8   0.1   1   2    0.0016       4.3    7.5   0.1     1    16   649   664   649   669 0.91 POLO box duplicated regionPOLO_box             PF00659.14    60 snap_masked-scaffold_7-processed-gene-0.883-mRNA-1 -           1004   1.6e-05   24.8   0.1   2   2   1.2e-05     0.031   14.3   0.0    15    32   828   845   820   905 0.83 POLO box duplicated regionKdo                  PF06293.10   207 snap_masked-scaffold_7-processed-gene-0.883-mRNA-1 -           1004   0.00015   21.0   0.0   1   2   1.5e-07    0.0004   19.6   0.0    99   155   148   204   134   219 0.83 Lipopolysaccharide kinase (Kdo/WaaP) familyKdo                  PF06293.10   207 snap_masked-scaffold_7-processed-gene-0.883-mRNA-1 -           1004   0.00015   21.0   0.0   2   2       2.2   5.8e+03   -3.7   0.0   178   200   369   396   365   400 0.71 Lipopolysaccharide kinase (Kdo/WaaP) familySushi_2              PF09014.6     88 snap_masked-scaffold_7-processed-gene-0.883-mRNA-1 -           1004     0.079   12.8   0.1   1   1   6.4e-05      0.17   11.7   0.1    10    60   103   154    94   158 0.83 Beta-2-glycoprotein-1 fifth domainDAO                  PF01266.20   336 maker-scaffold_7-snap-gene-0.760-mRNA-1 -            318   1.3e-33  116.9   3.2   1   2   2.6e-26   6.9e-23   81.7   0.8     3   193     4   198     2   212 0.73 FAD dependent oxidoreductaseDAO                  PF01266.20   336 maker-scaffold_7-snap-gene-0.760-mRNA-1 -            318   1.3e-33  116.9   3.2   2   2   1.8e-12   4.8e-09   36.1   0.1   266   336   212   303   192   303 0.86 FAD dependent oxidoreductaseNAD_binding_8        PF13450.2     68 maker-scaffold_7-snap-gene-0.760-mRNA-1 -            318   5.8e-06   26.3   0.5   1   2   9.2e-09   2.5e-05   24.3   0.1     1    38     5    44     5    75 0.85 NAD(P)-binding Rossmann-like domainNAD_binding_8        PF13450.2     68 maker-scaffold_7-snap-gene-0.760-mRNA-1 -            318   5.8e-06   26.3   0.5   2   2      0.68   1.8e+03   -0.9   0.0    33    55   106   128   105   138 0.75 NAD(P)-binding Rossmann-like domainPyr_redox            PF00070.23    81 maker-scaffold_7-snap-gene-0.760-mRNA-1 -            318   3.6e-05   24.1   0.0   1   2     6e-06     0.016   15.6   0.1     2    35     3    38     2    51 0.87 Pyridine nucleotide-disulphide oxidoreductasePyr_redox            PF00070.23    81 maker-scaffold_7-snap-gene-0.760-mRNA-1 -            318   3.6e-05   24.1   0.0   2   2    0.0061        16    6.0   0.0    18    78   124   185   113   188 0.76 Pyridine nucleotide-disulphide oxidoreductasePyr_redox_2          PF07992.10   292 maker-scaffold_7-snap-gene-0.760-mRNA-1 -            318    0.0068   15.4   0.1   1   2     3e-05     0.082   11.9   0.1   143   175     4    38     1    52 0.63 Pyridine nucleotide-disulphide oxidoreductasePyr_redox_2          PF07992.10   292 maker-scaffold_7-snap-gene-0.760-mRNA-1 -            318    0.0068   15.4   0.1   2   2     0.065   1.8e+02    0.9   0.0   154   211   120   177    78   199 0.75 Pyridine nucleotide-disulphide oxidoreductaseApbA                 PF02558.12   152 maker-scaffold_7-snap-gene-0.760-mRNA-1 -            318     0.032   13.7   0.1   1   1   2.5e-05     0.068   12.7   0.1     2    32     4    36     3    44 0.85 Ketopantoate reductase PanE/ApbAMCRA                 PF06100.7    496 maker-scaffold_7-snap-gene-0.760-mRNA-1 -            318      0.15   10.7   0.2   1   1   7.8e-05      0.21   10.2   0.2     4    44     3    41     1    55 0.79 MCRA familyTFIIA                PF03153.9    372 maker-scaffold_7-augustus-gene-0.1304-mRNA-1 -            598      0.11   12.5   0.1   1   3   1.3e-05      0.11   12.5   0.1   255   317   187   256    20   263 0.78 Transcription factor IIA, alpha/beta subunitTFIIA                PF03153.9    372 maker-scaffold_7-augustus-gene-0.1304-mRNA-1 -            598      0.11   12.5   0.1   2   3         2   1.6e+04   -8.3  17.4   286   329   398   441   276   457 0.65 Transcription factor IIA, alpha/beta subunitTFIIA                PF03153.9    372 maker-scaffold_7-augustus-gene-0.1304-mRNA-1 -            598      0.11   12.5   0.1   3   3      0.14   1.2e+03   -0.8   8.0    79   185   478   590   443   597 0.56 Transcription factor IIA, alpha/beta subunitCwf_Cwc_15           PF04889.8    223 maker-scaffold_7-augustus-gene-0.1304-mRNA-1 -            598      0.31   10.7  17.8   1   2    0.0016        13    5.4   3.3    66   123   189   256   168   267 0.45 Cwf15/Cwc15 cell cycle control proteinCwf_Cwc_15           PF04889.8    223 maker-scaffold_7-augustus-gene-0.1304-mRNA-1 -            598      0.31   10.7  17.8   2   2   4.3e-05      0.35   10.5   6.7    94   129   398   436   364   475 0.71 Cwf15/Cwc15 cell cycle control proteinMFS_1                PF07690.12   352 maker-scaffold_7-augustus-gene-0.1354-mRNA-1 -            620   4.2e-32  111.3  16.5   1   1   2.8e-35   7.4e-32  110.5  16.5     4   352   100   552    97   552 0.73 Major Facilitator SuperfamilySugar_tr             PF00083.20   452 maker-scaffold_7-augustus-gene-0.1354-mRNA-1 -            620   2.5e-08   33.1  11.6   1   3   3.7e-10     1e-06   27.8   6.8    43   176   132   262    97   281 0.80 Sugar (and other) transporterSugar_tr             PF00083.20   452 maker-scaffold_7-augustus-gene-0.1354-mRNA-1 -            620   2.5e-08   33.1  11.6   2   3     0.024        64    2.1   0.0   216   276   356   411   342   414 0.64 Sugar (and other) transporterSugar_tr             PF00083.20   452 maker-scaffold_7-augustus-gene-0.1354-mRNA-1 -            620   2.5e-08   33.1  11.6   3   3      0.11   3.1e+02   -0.2   0.6   118   176   515   580   489   593 0.70 Sugar (and other) transporterFolate_carrier       PF01770.14   412 maker-scaffold_7-augustus-gene-0.1354-mRNA-1 -            620   0.00018   20.3   0.0   1   1   1.1e-07   0.00031   19.5   0.0    65   187   159   288   144   414 0.72 Reduced folate carrierMFS_3                PF05977.9    522 maker-scaffold_7-augustus-gene-0.1354-mRNA-1 -            620    0.0015   16.9   2.8   1   3   5.5e-07    0.0015   16.9   2.8    46   178   136   263   121   295 0.67 Transmembrane secretion effectorMFS_3                PF05977.9    522 maker-scaffold_7-augustus-gene-0.1354-mRNA-1 -            620    0.0015   16.9   2.8   2   3      0.22   6.1e+02   -1.6   0.2   215   298   384   473   376   479 0.67 Transmembrane secretion effectorMFS_3                PF05977.9    522 maker-scaffold_7-augustus-gene-0.1354-mRNA-1 -            620    0.0015   16.9   2.8   3   3       1.4   3.9e+03   -4.3   0.0   150   163   546   559   541   561 0.84 Transmembrane secretion effectorMFS_2                PF13347.2    427 maker-scaffold_7-augustus-gene-0.1354-mRNA-1 -            620       1.4    7.3  22.9   1   4     2e-05     0.054   12.0   9.4   224   346    93   219    92   278 0.75 MFS/sugar transport proteinMFS_2                PF13347.2    427 maker-scaffold_7-augustus-gene-0.1354-mRNA-1 -            620       1.4    7.3  22.9   2   4     0.026        69    1.8   2.4    88   131   276   416   236   482 0.63 MFS/sugar transport proteinMFS_2                PF13347.2    427 maker-scaffold_7-augustus-gene-0.1354-mRNA-1 -            620       1.4    7.3  22.9   3   4    0.0054        15    4.0   0.3   220   318   385   499   358   562 0.56 MFS/sugar transport proteinMFS_2                PF13347.2    427 maker-scaffold_7-augustus-gene-0.1354-mRNA-1 -            620       1.4    7.3  22.9   4   4      0.29     8e+02   -1.7   0.1    66    91   567   592   529   599 0.72 MFS/sugar transport proteinFAM176               PF14851.2    144 maker-scaffold_7-augustus-gene-0.1354-mRNA-1 -            620       2.8    7.5   4.1   1   3      0.98   2.7e+03   -2.2   0.0    69   100    67    98    42   109 0.51 FAM176 familyFAM176               PF14851.2    144 maker-scaffold_7-augustus-gene-0.1354-mRNA-1 -            620       2.8    7.5   4.1   2   3         1   2.8e+03   -2.3   0.1    11    34   253   276   244   279 0.78 FAM176 familyFAM176               PF14851.2    144 maker-scaffold_7-augustus-gene-0.1354-mRNA-1 -            620       2.8    7.5   4.1   3   3   0.00057       1.6    8.3   0.6    61   104   342   382   317   393 0.49 FAM176 familyGlyoxalase           PF00903.21   128 maker-scaffold_7-snap-gene-0.745-mRNA-1 -            320   7.7e-38  129.4   0.1   1   2   1.4e-19   4.4e-16   59.1   0.0     2   128    12   149    11   149 0.92 Glyoxalase/Bleomycin resistance protein/Dioxygenase superfamilyGlyoxalase           PF00903.21   128 maker-scaffold_7-snap-gene-0.745-mRNA-1 -            320   7.7e-38  129.4   0.1   2   2   1.5e-22   4.9e-19   68.7   0.0     2   128   171   308   170   308 0.94 Glyoxalase/Bleomycin resistance protein/Dioxygenase superfamilyGlyoxalase_2         PF12681.3    111 maker-scaffold_7-snap-gene-0.745-mRNA-1 -            320   1.1e-24   87.4   0.0   1   2   2.8e-13   9.1e-10   39.3   0.0     1   111    17   150    17   150 0.71 Glyoxalase-like domainGlyoxalase_2         PF12681.3    111 maker-scaffold_7-snap-gene-0.745-mRNA-1 -            320   1.1e-24   87.4   0.0   2   2   3.4e-15   1.1e-11   45.5   0.0     1   111   176   309   176   309 0.85 Glyoxalase-like domainGlyoxalase_4         PF13669.2    109 maker-scaffold_7-snap-gene-0.745-mRNA-1 -            320   1.4e-14   54.2   0.0   1   3   1.9e-08   6.3e-05   23.1   0.0     4   102    16   132    13   139 0.71 Glyoxalase/Bleomycin resistance protein/Dioxygenase superfamilyGlyoxalase_4         PF13669.2    109 maker-scaffold_7-snap-gene-0.745-mRNA-1 -            320   1.4e-14   54.2   0.0   2   3   3.9e-09   1.3e-05   25.3   0.0     1    89   172   279   172   297 0.66 Glyoxalase/Bleomycin resistance protein/Dioxygenase superfamilyGlyoxalase_4         PF13669.2    109 maker-scaffold_7-snap-gene-0.745-mRNA-1 -            320   1.4e-14   54.2   0.0   3   3     4e-05      0.13   12.4   0.0     2    54   261   310   260   314 0.72 Glyoxalase/Bleomycin resistance protein/Dioxygenase superfamilyGlyoxalase_3         PF13468.2    175 maker-scaffold_7-snap-gene-0.745-mRNA-1 -            320   2.3e-05   24.4   0.0   1   2   0.00015      0.49   10.4   0.0    43   111    66   132    12   154 0.57 Glyoxalase-like domainGlyoxalase_3         PF13468.2    175 maker-scaffold_7-snap-gene-0.745-mRNA-1 -            320   2.3e-05   24.4   0.0   2   2   0.00018      0.58   10.1   0.0     2   100   172   281   171   299 0.67 Glyoxalase-like domainCppA_N               PF14506.2    124 maker-scaffold_7-snap-gene-0.745-mRNA-1 -            320     0.013   15.4   0.2   1   2     0.055   1.8e+02    2.0   0.0     1    28    12    38    12    41 0.88 CppA N-terminalCppA_N               PF14506.2    124 maker-scaffold_7-snap-gene-0.745-mRNA-1 -            320     0.013   15.4   0.2   2   2   8.6e-05      0.28   11.1   0.0     3    34   173   205   171   213 0.81 CppA N-terminalMeth_synt_2          PF01717.14   324 augustus_masked-scaffold_7-processed-gene-0.427-mRNA-1 -            362   1.6e-10   40.7   0.0   1   3   4.3e-06     0.023   13.8   0.0     6    77    10    81     5   112 0.92 Cobalamin-independent synthase, Catalytic domainMeth_synt_2          PF01717.14   324 augustus_masked-scaffold_7-processed-gene-0.427-mRNA-1 -            362   1.6e-10   40.7   0.0   2   3   0.00056       3.1    6.9   0.0   149   178   178   207   168   242 0.87 Cobalamin-independent synthase, Catalytic domainMeth_synt_2          PF01717.14   324 augustus_masked-scaffold_7-processed-gene-0.427-mRNA-1 -            362   1.6e-10   40.7   0.0   3   3   5.2e-07    0.0028   16.8   0.0   232   307   247   332   233   356 0.79 Cobalamin-independent synthase, Catalytic domainACD                  PF16671.1    386 augustus_masked-scaffold_7-processed-gene-0.427-mRNA-1 -            362    0.0041   15.4   0.1   1   2      0.17     9e+02   -2.2   0.0   188   214   182   208   179   211 0.89 Actin cross-linking domainACD                  PF16671.1    386 augustus_masked-scaffold_7-processed-gene-0.427-mRNA-1 -            362    0.0041   15.4   0.1   2   2     2e-06     0.011   14.0   0.0   126   174   289   338   282   352 0.88 Actin cross-linking domainMeth_synt_1          PF08267.8    313 augustus_masked-scaffold_7-processed-gene-0.427-mRNA-1 -            362     0.016   14.6   0.0   1   2     0.071   3.8e+02    0.2   0.0    26    61    29    64    19    69 0.89 Cobalamin-independent synthase, N-terminal domainMeth_synt_1          PF08267.8    313 augustus_masked-scaffold_7-processed-gene-0.427-mRNA-1 -            362     0.016   14.6   0.0   2   2     2e-05      0.11   11.9   0.0   172   219   173   220   152   253 0.85 Cobalamin-independent synthase, N-terminal domainHgmA                 PF04209.9    426 augustus_masked-scaffold_7-processed-gene-0.413-mRNA-1 -            428  7.2e-152  505.8   0.0   1   2   5.1e-62   8.3e-58  195.9   0.0    55   234     1   192     1   199 0.87 homogentisate 1,2-dioxygenaseHgmA                 PF04209.9    426 augustus_masked-scaffold_7-processed-gene-0.413-mRNA-1 -            428  7.2e-152  505.8   0.0   2   2   5.6e-96     9e-92  307.8   0.2   233   425   214   412   209   413 0.95 homogentisate 1,2-dioxygenaseTB2_DP1_HVA22        PF03134.15    91 snap_masked-scaffold_7-processed-gene-0.985-mRNA-1 -            351   2.1e-27   94.7   8.8   1   1   6.2e-31   3.4e-27   94.1   8.8     2    91     7    94     6    94 0.97 TB2/DP1, HVA22 familySUIM_assoc           PF16619.1     64 snap_masked-scaffold_7-processed-gene-0.985-mRNA-1 -            351     0.032   14.2   7.5   1   2     0.014        74    3.4   4.0    32    55   251   274   247   280 0.90 Unstructured region C-term to UIM in Ataxin3SUIM_assoc           PF16619.1     64 snap_masked-scaffold_7-processed-gene-0.985-mRNA-1 -            351     0.032   14.2   7.5   2   2   3.9e-05      0.21   11.5   0.1    21    41   290   351   285   351 0.81 Unstructured region C-term to UIM in Ataxin3CASP_C               PF08172.8    252 snap_masked-scaffold_7-processed-gene-0.985-mRNA-1 -            351     0.074   12.2   0.1   1   2      0.84   4.6e+03   -3.4   0.0   183   192    86    95    84    96 0.83 CASP C terminalCASP_C               PF08172.8    252 snap_masked-scaffold_7-processed-gene-0.985-mRNA-1 -            351     0.074   12.2   0.1   2   2   3.3e-05      0.18   11.0   0.1    59   128   201   270   166   276 0.71 CASP C terminalApbA                 PF02558.12   152 augustus_masked-scaffold_7-processed-gene-0.401-mRNA-1 -            348   5.9e-26   90.8   0.0   1   1   1.1e-29   8.7e-26   90.3   0.0    20   151    19   160    14   161 0.96 Ketopantoate reductase PanE/ApbAApbA_C               PF08546.7    125 augustus_masked-scaffold_7-processed-gene-0.401-mRNA-1 -            348   2.9e-25   88.7   0.0   1   2      0.65   5.3e+03   -2.6   0.0    14    54    99   139    95   154 0.74 Ketopantoate reductase PanE/ApbA C terminalApbA_C               PF08546.7    125 augustus_masked-scaffold_7-processed-gene-0.401-mRNA-1 -            348   2.9e-25   88.7   0.0   2   2   8.3e-29   6.8e-25   87.6   0.0     2   124   213   338   212   339 0.92 Ketopantoate reductase PanE/ApbA C terminalRIC1                 PF07064.9    248 maker-scaffold_7-augustus-gene-0.1279-mRNA-1 -           1141   8.9e-89  297.0   0.3   1   1   7.6e-93   1.2e-88  296.6   0.3     1   247   813  1060   813  1061 0.97 RIC1PHO4                 PF01384.16   353 augustus_masked-scaffold_7-processed-gene-0.410-mRNA-1 -            605  1.6e-105  352.9  18.7   1   1  1.2e-109  1.9e-105  352.7  18.7     1   352    24   591    24   592 0.98 Phosphate transporter familyGCS                  PF03074.12   365 maker-scaffold_7-augustus-gene-0.1298-mRNA-1 -            757  2.7e-172  573.1   0.0   1   1    2e-176  3.2e-172  572.8   0.0     1   363   257   659   257   661 0.98 Glutamate-cysteine ligaseTfb2                 PF03849.10   360 maker-scaffold_7-augustus-gene-0.1323-mRNA-1 -            524  8.3e-134  446.0   0.0   1   1  1.3e-137    1e-133  445.7   0.0     1   360    13   432    13   432 0.95 Transcription factor Tfb2Helicase_C_3         PF13625.2    127 maker-scaffold_7-augustus-gene-0.1323-mRNA-1 -            524   0.00063   19.7   0.3   1   2      0.68   5.5e+03   -2.7   0.0    51    68   235   252   230   257 0.77 Helicase conserved C-terminal domainHelicase_C_3         PF13625.2    127 maker-scaffold_7-augustus-gene-0.1323-mRNA-1 -            524   0.00063   19.7   0.3   2   2   2.6e-07    0.0021   18.0   0.1    23    70   384   431   362   479 0.79 Helicase conserved C-terminal domainVint                 PF14623.2    167 maker-scaffold_7-snap-gene-0.730-mRNA-1 -            689   2.6e-55  186.6   0.0   1   1   1.8e-58   4.1e-55  186.0   0.0     1   167   471   649   471   649 0.97 Hint-domainVwaint               PF14624.2     69 maker-scaffold_7-snap-gene-0.730-mRNA-1 -            689   5.9e-19   67.8   0.0   1   1   5.7e-22   1.3e-18   66.6   0.0     4    68   347   418   344   419 0.96 VWA / Hh  protein intein-likeVWA_3                PF13768.2    155 maker-scaffold_7-snap-gene-0.730-mRNA-1 -            689   6.5e-07   29.2   0.0   1   2   8.1e-10   1.9e-06   27.7   0.0    18   155    13   154     4   154 0.84 von Willebrand factor type A domainVWA_3                PF13768.2    155 maker-scaffold_7-snap-gene-0.730-mRNA-1 -            689   6.5e-07   29.2   0.0   2   2       1.9   4.4e+03   -2.7   0.0    92   129   435   473   417   474 0.64 von Willebrand factor type A domainVWA                  PF00092.24   175 maker-scaffold_7-snap-gene-0.730-mRNA-1 -            689   0.00035   20.7   0.0   1   1   2.5e-07   0.00058   20.0   0.0    16   129    11   119     4   158 0.79 von Willebrand factor type A domainHCV_NS1              PF01560.13   344 maker-scaffold_7-snap-gene-0.730-mRNA-1 -            689     0.063   11.8   0.2   1   1   4.6e-05      0.11   11.0   0.2    97   135   127   165   108   176 0.87 Hepatitis C virus non-structural protein E2/NS1Hint_2               PF13403.2    143 maker-scaffold_7-snap-gene-0.730-mRNA-1 -            689      0.14   12.3   0.1   1   1   0.00011      0.25   11.5   0.1     1    46   471   516   471   544 0.89 Hint domainHint                 PF01079.16   214 maker-scaffold_7-snap-gene-0.730-mRNA-1 -            689      0.18   11.1   0.0   1   1   0.00015      0.34   10.2   0.0     9    39   469   499   462   515 0.89 Hint moduleSH3_9                PF14604.2     49 maker-scaffold_7-augustus-gene-0.1270-mRNA-1 -            768   3.4e-26   90.7   4.2   1   2   1.6e-14   5.1e-11   42.1   0.7     2    49   621   669   620   669 0.97 Variant SH3 domainSH3_9                PF14604.2     49 maker-scaffold_7-augustus-gene-0.1270-mRNA-1 -            768   3.4e-26   90.7   4.2   2   2   7.2e-17   2.3e-13   49.6   0.2     1    48   717   764   717   765 0.98 Variant SH3 domainSH3_1                PF00018.24    48 maker-scaffold_7-augustus-gene-0.1270-mRNA-1 -            768   3.4e-23   80.8   4.3   1   2   9.7e-13   3.2e-09   36.1   0.3     2    48   620   665   619   665 0.96 SH3 domainSH3_1                PF00018.24    48 maker-scaffold_7-augustus-gene-0.1270-mRNA-1 -            768   3.4e-23   80.8   4.3   2   2   8.5e-16   2.8e-12   45.9   0.4     1    48   716   761   716   761 0.96 SH3 domainSH3_2                PF07653.13    55 maker-scaffold_7-augustus-gene-0.1270-mRNA-1 -            768   5.2e-14   51.5   0.2   1   2   1.9e-09   6.3e-06   25.6   0.2     2    54   618   670   617   671 0.91 Variant SH3 domainSH3_2                PF07653.13    55 maker-scaffold_7-augustus-gene-0.1270-mRNA-1 -            768   5.2e-14   51.5   0.2   2   2     1e-08   3.4e-05   23.3   0.0     4    53   717   765   715   766 0.89 Variant SH3 domainCofilin_ADF          PF00241.16   123 maker-scaffold_7-augustus-gene-0.1270-mRNA-1 -            768   9.4e-10   38.6   0.0   1   2   6.9e-13   2.2e-09   37.4   0.0    25   123    55   149    14   149 0.85 Cofilin/tropomyosin-type actin-binding proteinCofilin_ADF          PF00241.16   123 maker-scaffold_7-augustus-gene-0.1270-mRNA-1 -            768   9.4e-10   38.6   0.0   2   2       3.2     1e+04   -3.5   0.0    16    33   619   636   610   647 0.68 Cofilin/tropomyosin-type actin-binding proteinDUF1501              PF07394.8    392 maker-scaffold_7-augustus-gene-0.1270-mRNA-1 -            768      0.11   11.3   0.6   1   2      0.81   2.6e+03   -3.0   0.0   100   139    87   122    85   146 0.71 Protein of unknown function (DUF1501)DUF1501              PF07394.8    392 maker-scaffold_7-augustus-gene-0.1270-mRNA-1 -            768      0.11   11.3   0.6   2   2   8.4e-05      0.27   10.1   0.7    95   164   403   473   318   488 0.86 Protein of unknown function (DUF1501)Med14                PF08638.7    191 maker-scaffold_7-snap-gene-0.685-mRNA-1 -           1053   2.1e-59  200.0   0.0   1   1   2.3e-63   3.8e-59  199.2   0.0     1   191    51   266    51   266 0.98 Mediator complex subunit MED14Cyclin_N             PF00134.19   127 augustus_masked-scaffold_7-processed-gene-0.353-mRNA-1 -            536   9.4e-43  144.9   0.1   1   2     1e-45   2.3e-42  143.6   0.0     1   126   252   377   252   378 0.99 Cyclin, N-terminal domainCyclin_N             PF00134.19   127 augustus_masked-scaffold_7-processed-gene-0.353-mRNA-1 -            536   9.4e-43  144.9   0.1   2   2       3.1   7.1e+03   -3.4   0.0    74    84   422   432   407   439 0.74 Cyclin, N-terminal domainCyclin_C             PF02984.15   118 augustus_masked-scaffold_7-processed-gene-0.353-mRNA-1 -            536   2.3e-34  117.9   0.0   1   1   2.3e-37   5.3e-34  116.7   0.0     1   112   380   488   380   493 0.94 Cyclin, C-terminal domainSAGA-Tad1            PF12767.3    235 augustus_masked-scaffold_7-processed-gene-0.353-mRNA-1 -            536      0.24   11.0   3.0   1   1   0.00018      0.41   10.2   3.0    93   167   131   221    93   267 0.68 Transcriptional regulator of RNA polII, SAGA, subunitCMV_1a               PF12467.4    169 augustus_masked-scaffold_7-processed-gene-0.353-mRNA-1 -            536       1.3    9.2   8.6   1   1    0.0013         3    8.0   8.6    60   143    88   175    74   194 0.69 Cucumber mosaic virus 1a protein familyTFIIA                PF03153.9    372 augustus_masked-scaffold_7-processed-gene-0.353-mRNA-1 -            536       2.2    8.1  10.2   1   1    0.0013         3    7.7  10.2    75   247    27   223     2   273 0.40 Transcription factor IIA, alpha/beta subunitFSA_C                PF10479.5    706 augustus_masked-scaffold_7-processed-gene-0.353-mRNA-1 -            536       3.6    5.5  10.4   1   1    0.0021         5    5.0  10.4   547   611   103   168    45   199 0.52 Fragile site-associated protein C-terminusDicty_REP            PF05086.8    911 augustus_masked-scaffold_7-processed-gene-0.353-mRNA-1 -            536         7    4.3   9.8   1   1    0.0042       9.8    3.9   9.8   243   327   116   207    95   240 0.57 Dictyostelium (Slime Mold) REP proteinATG22                PF11700.4    467 augustus_masked-scaffold_7-processed-gene-0.373-mRNA-1 -            681  2.6e-187  622.9  11.4   1   1  3.7e-191    3e-187  622.7  11.4     1   466    96   630    96   631 0.99 Vacuole effluxer Atg22 likeMFS_2                PF13347.2    427 augustus_masked-scaffold_7-processed-gene-0.373-mRNA-1 -            681     0.028   13.0  20.3   1   2    0.0005       4.1    5.8   6.3   257   340   170   254   156   260 0.76 MFS/sugar transport proteinMFS_2                PF13347.2    427 augustus_masked-scaffold_7-processed-gene-0.373-mRNA-1 -            681     0.028   13.0  20.3   2   2   4.8e-06     0.039   12.5   6.0   148   309   344   520   319   618 0.73 MFS/sugar transport proteinBac_surface_Ag       PF01103.19   325 maker-scaffold_7-augustus-gene-0.1318-mRNA-1 -            532   1.3e-36  126.6   0.0   1   1   1.1e-40   1.7e-36  126.2   0.0     1   325   181   531   181   531 0.88 Surface antigenCyto_heme_lyase      PF01265.13   292 maker-scaffold_7-augustus-gene-0.1316-mRNA-1 -            354  1.3e-122  408.9   1.2   1   1  1.2e-125  1.9e-121  405.0   1.2     1   292     1   329     1   329 0.92 Cytochrome c/c1 heme lyaseRibosomal_S27e       PF01667.13    55 maker-scaffold_7-augustus-gene-0.1345-mRNA-1 -             83   3.2e-28   97.1   7.5   1   1   7.3e-32     4e-28   96.8   7.5     1    55    29    83    29    83 0.99 Ribosomal protein S27TF_Zn_Ribbon         PF08271.8     43 maker-scaffold_7-augustus-gene-0.1345-mRNA-1 -             83      0.14   11.5   3.7   1   1   3.9e-05      0.21   11.0   3.7     2    29    37    64    36    66 0.94 TFIIB zinc-bindingzf-C2HC5             PF06221.9     54 maker-scaffold_7-augustus-gene-0.1345-mRNA-1 -             83       1.1    9.1   7.1   1   2     0.052   2.8e+02    1.4   0.0     7    18    16    27    14    30 0.86 Putative zinc finger motif, C2HC5-typezf-C2HC5             PF06221.9     54 maker-scaffold_7-augustus-gene-0.1345-mRNA-1 -             83       1.1    9.1   7.1   2   2   0.00048       2.6    8.0   4.4     5    39    42    80    37    82 0.77 Putative zinc finger motif, C2HC5-typeAmidohydro_1         PF01979.16   344 snap_masked-scaffold_7-processed-gene-0.1015-mRNA-1 -            436   1.9e-41  142.4   0.0   1   1   5.6e-45   2.3e-41  142.1   0.0     1   343    71   423    71   424 0.88 Amidohydrolase familyAmidohydro_3         PF07969.7    474 snap_masked-scaffold_7-processed-gene-0.1015-mRNA-1 -            436   8.2e-16   58.3   0.1   1   2   4.4e-07    0.0018   17.6   0.1     2    23    64    85    63   101 0.91 Amidohydrolase familyAmidohydro_3         PF07969.7    474 snap_masked-scaffold_7-processed-gene-0.1015-mRNA-1 -            436   8.2e-16   58.3   0.1   2   2     2e-13     8e-10   38.5   0.0   230   452   201   402   186   424 0.80 Amidohydrolase familyThioredoxin_2        PF13098.2    111 snap_masked-scaffold_7-processed-gene-0.1015-mRNA-1 -            436     0.012   15.7   0.0   1   1   5.4e-06     0.022   14.9   0.0    53   107   233   310   218   313 0.70 Thioredoxin-like domainDHO_dh               PF01180.17   295 snap_masked-scaffold_7-processed-gene-0.1015-mRNA-1 -            436      0.11   11.5   0.0   1   1   4.1e-05      0.17   10.9   0.0   243   271   203   231   188   246 0.79 Dihydroorotate dehydrogenaseDioxygenase_C        PF00775.17   182 snap_masked-scaffold_7-processed-gene-0.1003-mRNA-1 -            324   1.2e-42  145.2   0.0   1   1   3.3e-46   1.8e-42  144.7   0.0     1   181   129   309   129   310 0.96 DioxygenaseDioxygenase_N        PF04444.10    75 snap_masked-scaffold_7-processed-gene-0.1003-mRNA-1 -            324     8e-23   80.0   0.1   1   1   2.4e-26   1.3e-22   79.3   0.1     2    68    44   114    43   117 0.91 Catechol dioxygenase N terminusCarboxypepD_reg      PF13620.2     82 snap_masked-scaffold_7-processed-gene-0.1003-mRNA-1 -            324    0.0071   16.4   0.0   1   1   5.8e-06     0.032   14.4   0.0     2    60   160   234   159   251 0.86 Carboxypeptidase regulatory-like domainDUF21                PF01595.16   184 maker-scaffold_7-snap-gene-0.746-mRNA-1 -            553   3.1e-31  108.2   0.0   1   1   5.8e-35   4.7e-31  107.6   0.0     4   182    39   213    36   215 0.89 Domain of unknown function DUF21CBS                  PF00571.24    57 maker-scaffold_7-snap-gene-0.746-mRNA-1 -            553   1.3e-08   35.0   1.5   1   2   2.1e-06     0.017   15.4   0.2     1    55   234   293   234   295 0.86 CBS domainCBS                  PF00571.24    57 maker-scaffold_7-snap-gene-0.746-mRNA-1 -            553   1.3e-08   35.0   1.5   2   2   4.7e-07    0.0038   17.5   0.1     9    55   308   357   300   359 0.79 CBS domainExonuc_VII_L         PF02601.11   306 maker-scaffold_7-snap-gene-0.710-mRNA-1 -            173     0.098   12.1   0.0   1   1   7.6e-06      0.12   11.7   0.0   119   207    47   133    44   167 0.64 Exonuclease VII, large subunitG6PD_C               PF02781.12   293 maker-scaffold_7-augustus-gene-0.1276-mRNA-1 -            503  1.8e-114  381.7   0.0   1   2      0.69   3.8e+03   -3.4   0.0    95   101    89    95    59   112 0.57 Glucose-6-phosphate dehydrogenase, C-terminal domainG6PD_C               PF02781.12   293 maker-scaffold_7-augustus-gene-0.1276-mRNA-1 -            503  1.8e-114  381.7   0.0   2   2  6.1e-118  3.3e-114  380.9   0.0     1   292   196   484   196   485 0.95 Glucose-6-phosphate dehydrogenase, C-terminal domainG6PD_N               PF00479.18   168 maker-scaffold_7-augustus-gene-0.1276-mRNA-1 -            503   2.7e-59  200.5   0.0   1   1   8.3e-63   4.5e-59  199.8   0.0     1   168    17   194    17   194 0.93 Glucose-6-phosphate dehydrogenase, NAD binding domainArAE_1_C             PF11728.4    166 maker-scaffold_7-augustus-gene-0.1276-mRNA-1 -            503    0.0042   17.0   0.1   1   1   1.4e-06    0.0076   16.1   0.1    58   131    39   113    26   119 0.92 Putative aromatic acid exporter C-terminal domainAbhydrolase_1        PF00561.16   256 augustus_masked-scaffold_7-processed-gene-0.492-mRNA-1 -            469   1.8e-45  155.5   0.0   1   1   2.6e-48   2.1e-44  151.9   0.0     1   236    78   430    78   457 0.89 alpha/beta hydrolase foldEsterase             PF00756.16   251 augustus_masked-scaffold_7-processed-gene-0.492-mRNA-1 -            469   7.3e-05   22.4   0.0   1   1   1.5e-08   0.00012   21.7   0.0   102   151   156   210   141   246 0.84 Putative esteraseEpimerase            PF01370.17   241 maker-scaffold_7-augustus-gene-0.1296-mRNA-1 -            376   3.4e-17   62.6   0.0   1   1   1.4e-20   4.6e-17   62.1   0.0     1   234     8   239     8   245 0.86 NAD dependent epimerase/dehydratase familyRmlD_sub_bind        PF04321.13   287 maker-scaffold_7-augustus-gene-0.1296-mRNA-1 -            376   1.4e-06   27.5   0.0   1   1   5.5e-10   1.8e-06   27.1   0.0     3   167     8   195     6   280 0.78 RmlD substrate binding domainGDP_Man_Dehyd        PF16363.1    332 maker-scaffold_7-augustus-gene-0.1296-mRNA-1 -            376   3.9e-05   23.0   0.0   1   1     2e-08   6.7e-05   22.3   0.0     1   236     9   230     9   239 0.80 GDP-mannose 4,6 dehydratase3Beta_HSD            PF01073.15   282 maker-scaffold_7-augustus-gene-0.1296-mRNA-1 -            376   0.00046   19.1   0.0   1   1   2.5e-07    0.0008   18.3   0.0     1   191     9   188     9   225 0.72 3-beta hydroxysteroid dehydrogenase/isomerase familySacchrp_dh_NADP      PF03435.14   130 maker-scaffold_7-augustus-gene-0.1296-mRNA-1 -            376     0.045   13.9   0.0   1   1   2.4e-05     0.079   13.1   0.0     1    44     8    51     8    98 0.78 Saccharopine dehydrogenase NADP binding domainDUF1752              PF08550.6     28 augustus_masked-scaffold_7-processed-gene-0.363-mRNA-1 -            694   1.5e-10   40.6   7.3   1   1   1.9e-14     3e-10   39.6   7.3     1    28   257   284   257   284 0.98 Fungal protein of unknown function (DUF1752)Zn_clus              PF00172.14    40 maker-scaffold_7-augustus-gene-0.1311-mRNA-1 -            770   5.1e-09   36.0  11.3   1   1   9.8e-13   7.9e-09   35.4  11.3     1    39   213   255   213   256 0.89 Fungal Zn(2)-Cys(6) binuclear cluster domainFungal_trans         PF04082.14   267 maker-scaffold_7-augustus-gene-0.1311-mRNA-1 -            770   1.8e-08   33.6   0.0   1   1     4e-12   3.3e-08   32.8   0.0    41   181   431   553   393   560 0.71 Fungal specific transcription factor domainPhyH                 PF05721.9    211 maker-scaffold_7-augustus-gene-0.1359-mRNA-1 -            338     5e-14   52.9   0.0   1   1   7.5e-18   1.2e-13   51.7   0.0     2   211    57   270    56   270 0.73 Phytanoyl-CoA dioxygenase (PhyH)CWC25                PF12542.4     92 augustus_masked-scaffold_7-processed-gene-0.482-mRNA-1 -            443   2.4e-27   95.4   0.3   1   4       0.8   6.5e+03   -2.1   0.4    22    42    28    47    16    61 0.47 Pre-mRNA splicing factorCWC25                PF12542.4     92 augustus_masked-scaffold_7-processed-gene-0.482-mRNA-1 -            443   2.4e-27   95.4   0.3   2   4   2.9e-31   2.4e-27   95.4   0.3     2    92    66   157    65   157 0.86 Pre-mRNA splicing factorCWC25                PF12542.4     92 augustus_masked-scaffold_7-processed-gene-0.482-mRNA-1 -            443   2.4e-27   95.4   0.3   3   4      0.59   4.8e+03   -1.7   0.7    37    42   277   282   233   314 0.52 Pre-mRNA splicing factorCWC25                PF12542.4     92 augustus_masked-scaffold_7-processed-gene-0.482-mRNA-1 -            443   2.4e-27   95.4   0.3   4   4      0.56   4.6e+03   -1.6   0.3    50    58   368   376   333   404 0.48 Pre-mRNA splicing factorCir_N                PF10197.5     37 augustus_masked-scaffold_7-processed-gene-0.482-mRNA-1 -            443   2.6e-12   46.6   2.4   1   4   3.2e-16   2.6e-12   46.6   2.4     1    37    11    47    11    47 0.99 N-terminal domain of CBF1 interacting co-repressor CIRCir_N                PF10197.5     37 augustus_masked-scaffold_7-processed-gene-0.482-mRNA-1 -            443   2.6e-12   46.6   2.4   2   4       1.5   1.2e+04   -3.6   0.5    22    34   248   260   247   261 0.76 N-terminal domain of CBF1 interacting co-repressor CIRCir_N                PF10197.5     37 augustus_masked-scaffold_7-processed-gene-0.482-mRNA-1 -            443   2.6e-12   46.6   2.4   3   4      0.72   5.8e+03   -2.5   0.6    20    33   283   296   281   298 0.82 N-terminal domain of CBF1 interacting co-repressor CIRCir_N                PF10197.5     37 augustus_masked-scaffold_7-processed-gene-0.482-mRNA-1 -            443   2.6e-12   46.6   2.4   4   4      0.97   7.9e+03   -3.0   0.7    20    31   363   374   361   379 0.60 N-terminal domain of CBF1 interacting co-repressor CIRWWbp                 PF10349.5    112 maker-scaffold_7-augustus-gene-0.1336-mRNA-1 -            265   1.9e-17   64.5   5.8   1   1   1.1e-20   3.1e-17   63.8   5.8     1   112   129   243   129   243 0.78 WW-domain ligand proteinVps36_ESCRT-II       PF11605.4     92 maker-scaffold_7-augustus-gene-0.1336-mRNA-1 -            265     0.011   15.7   0.0   1   2       0.6   1.6e+03   -0.8   0.0    35    70    30    64    24    72 0.68 Vacuolar protein sorting protein 36 Vps36Vps36_ESCRT-II       PF11605.4     92 maker-scaffold_7-augustus-gene-0.1336-mRNA-1 -            265     0.011   15.7   0.0   2   2   1.9e-05      0.05   13.6   0.0    36    77    72   113    65   119 0.73 Vacuolar protein sorting protein 36 Vps36GRAM                 PF02893.16    69 maker-scaffold_7-augustus-gene-0.1336-mRNA-1 -            265     0.024   14.2   0.0   1   1   1.6e-05     0.042   13.4   0.0    11    50    38    92    31   104 0.84 GRAM domainTMEM52               PF14979.2    149 maker-scaffold_7-augustus-gene-0.1336-mRNA-1 -            265     0.031   14.1   0.9   1   1     2e-05     0.053   13.3   0.9    78   125   195   242   173   254 0.92 Transmembrane 52PH_TFIIH             PF08567.7     87 maker-scaffold_7-augustus-gene-0.1336-mRNA-1 -            265      0.11   12.7   0.0   1   1   7.8e-05      0.21   11.8   0.0     2    46    68   113    67   116 0.85 TFIIH p62 subunit, N-terminal domainbPH_3                PF14470.2     96 maker-scaffold_7-augustus-gene-0.1336-mRNA-1 -            265      0.17   12.2   0.0   1   1   0.00012      0.33   11.2   0.0    19    49    69    99    38   100 0.81 Bacterial PH domainRan-binding          PF05508.7    311 augustus_masked-scaffold_7-processed-gene-0.398-mRNA-1 -            577  1.1e-109  366.0   0.0   1   1  9.8e-114  1.6e-109  365.5   0.0     1   310     1   355     1   356 0.95 RanGTP-binding proteinCholine_kinase       PF01633.16   213 maker-scaffold_7-augustus-gene-0.1284-mRNA-1 -            443   6.3e-48  163.0   0.0   1   2   6.6e-51   5.4e-47  160.0   0.0     3   212    80   337    78   338 0.90 Choline/ethanolamine kinaseCholine_kinase       PF01633.16   213 maker-scaffold_7-augustus-gene-0.1284-mRNA-1 -            443   6.3e-48  163.0   0.0   2   2     0.038   3.1e+02    0.7   0.0   164   177   394   407   369   411 0.88 Choline/ethanolamine kinaseAPH                  PF01636.19   240 maker-scaffold_7-augustus-gene-0.1284-mRNA-1 -            443   9.3e-07   28.9   1.8   1   1   6.4e-10   5.2e-06   26.4   1.7    25   212    84   323    73   355 0.73 Phosphotransferase enzyme familyAmidase              PF01425.17   451 snap_masked-scaffold_7-processed-gene-0.1027-mRNA-1 -            524  4.1e-109  365.4   0.1   1   1  6.8e-113  1.1e-108  364.0   0.1    50   451    62   512    50   512 0.94 AmidaseAldedh               PF00171.18   462 maker-scaffold_7-augustus-gene-0.1352-mRNA-1 -            536  1.4e-177  590.7   0.5   1   1  9.8e-182  1.6e-177  590.5   0.5     7   462    64   526    57   526 0.98 Aldehyde dehydrogenase familyRrnaAD               PF00398.16   265 augustus_masked-scaffold_7-processed-gene-0.438-mRNA-1 -            394     4e-64  216.1   0.0   1   1   4.3e-67   6.3e-64  215.5   0.0     2   224    35   250    34   272 0.96 Ribosomal RNA adenine dimethylaseMethyltransf_26      PF13659.2    124 augustus_masked-scaffold_7-processed-gene-0.438-mRNA-1 -            394   5.8e-08   33.3   0.0   1   1   7.3e-11   1.1e-07   32.4   0.0     2    83    65   157    64   200 0.69 Methyltransferase domainMethyltransf_18      PF12847.3    110 augustus_masked-scaffold_7-processed-gene-0.438-mRNA-1 -            394   8.1e-06   26.5   0.0   1   1   1.5e-08   2.2e-05   25.1   0.0     2    78    64   136    63   164 0.85 Methyltransferase domainMethyltransf_11      PF08241.8     95 augustus_masked-scaffold_7-processed-gene-0.438-mRNA-1 -            394     1e-05   25.9   0.2   1   2   3.6e-08   5.4e-05   23.6   0.0     1    67    68   135    68   144 0.95 Methyltransferase domainMethyltransf_11      PF08241.8     95 augustus_masked-scaffold_7-processed-gene-0.438-mRNA-1 -            394     1e-05   25.9   0.2   2   2       1.5   2.1e+03   -0.8   0.2    12    63   259   311   252   312 0.72 Methyltransferase domainCMAS                 PF02353.16   273 augustus_masked-scaffold_7-processed-gene-0.438-mRNA-1 -            394    0.0017   17.6   0.0   1   1   1.8e-06    0.0027   16.9   0.0    53   120    54   120    51   134 0.91 Mycolic acid cyclopropane synthetasePCMT                 PF01135.15   210 augustus_masked-scaffold_7-processed-gene-0.438-mRNA-1 -            394    0.0031   17.1   0.0   1   1   3.3e-06    0.0049   16.5   0.0    64   132    54   120    28   127 0.84 Protein-L-isoaspartate(D-aspartate) O-methyltransferase (PCMT)Methyltransf_12      PF08242.8     99 augustus_masked-scaffold_7-processed-gene-0.438-mRNA-1 -            394    0.0042   17.6   0.0   1   2   1.4e-05     0.021   15.4   0.0     1    72    68   134    68   143 0.86 Methyltransferase domainMethyltransf_12      PF08242.8     99 augustus_masked-scaffold_7-processed-gene-0.438-mRNA-1 -            394    0.0042   17.6   0.0   2   2       2.5   3.7e+03   -1.5   0.1    21    61   269   309   253   320 0.67 Methyltransferase domainMTS                  PF05175.10   169 augustus_masked-scaffold_7-processed-gene-0.438-mRNA-1 -            394     0.014   14.8   0.0   1   1   1.6e-05     0.023   14.1   0.0    31   108    63   139    50   148 0.78 Methyltransferase small domainMethyltransf_23      PF13489.2    165 augustus_masked-scaffold_7-processed-gene-0.438-mRNA-1 -            394     0.025   14.4   0.0   1   1   3.7e-05     0.055   13.3   0.0    19    63    60   104    42   145 0.75 Methyltransferase domainMethyltransf_2       PF00891.14   227 augustus_masked-scaffold_7-processed-gene-0.438-mRNA-1 -            394     0.032   13.3   0.0   1   1   3.5e-05     0.052   12.6   0.0    63   144    52   133    23   137 0.82 O-methyltransferaseMethyltransf_31      PF13847.2    152 augustus_masked-scaffold_7-processed-gene-0.438-mRNA-1 -            394     0.051   13.3   0.0   1   1   5.4e-05      0.08   12.6   0.0     2    83    62   138    61   196 0.85 Methyltransferase domainRNR_inhib            PF08591.6    107 augustus_masked-scaffold_7-processed-gene-0.464-mRNA-1 -            258   3.6e-31  108.5   0.0   1   2   7.1e-35   1.2e-30  106.9   0.0     1   106    54   154    54   155 0.89 Ribonucleotide reductase inhibitorRNR_inhib            PF08591.6    107 augustus_masked-scaffold_7-processed-gene-0.464-mRNA-1 -            258   3.6e-31  108.5   0.0   2   2      0.53   8.6e+03   -2.1   0.0    57    57   196   196   163   238 0.55 Ribonucleotide reductase inhibitorParA                 PF10609.5    246 augustus_masked-scaffold_7-processed-gene-0.405-mRNA-1 -            303   1.5e-92  309.3   0.0   1   1   1.2e-95   1.8e-92  309.1   0.0     2   245    47   290    46   291 0.97 NUBPL iron-transfer P-loop NTPaseCbiA                 PF01656.19   195 augustus_masked-scaffold_7-processed-gene-0.405-mRNA-1 -            303   2.8e-14   53.1   0.1   1   1   2.7e-17     4e-14   52.6   0.1     1   157    51   218    51   220 0.81 CobQ/CobB/MinD/ParA nucleotide binding domainAAA_31               PF13614.2    154 augustus_masked-scaffold_7-processed-gene-0.405-mRNA-1 -            303   9.3e-09   35.2   0.0   1   1   6.9e-11     1e-07   31.9   0.0     1    59    49   108    49   178 0.69 AAA domainMipZ                 PF09140.7    262 augustus_masked-scaffold_7-processed-gene-0.405-mRNA-1 -            303   0.00032   19.9   0.1   1   1   3.1e-07   0.00045   19.4   0.1     2    39    50    87    49   172 0.91 ATPase MipZArsA_ATPase          PF02374.11   306 augustus_masked-scaffold_7-processed-gene-0.405-mRNA-1 -            303    0.0012   18.0   0.2   1   2   8.2e-06     0.012   14.7   0.1     7    38    55    86    48   102 0.86 Anion-transporting ATPaseArsA_ATPase          PF02374.11   306 augustus_masked-scaffold_7-processed-gene-0.405-mRNA-1 -            303    0.0012   18.0   0.2   2   2      0.12   1.8e+02    1.0   0.0   115   140   144   170   139   178 0.83 Anion-transporting ATPaseAAA_26               PF13500.2    200 augustus_masked-scaffold_7-processed-gene-0.405-mRNA-1 -            303    0.0059   16.3   0.1   1   2     0.037        55    3.3   0.1     3    29    51    77    49    84 0.82 AAA domainAAA_26               PF13500.2    200 augustus_masked-scaffold_7-processed-gene-0.405-mRNA-1 -            303    0.0059   16.3   0.1   2   2   0.00019      0.27   10.8   0.0   133   197   184   261   163   264 0.70 AAA domainAAA_25               PF13481.2    194 augustus_masked-scaffold_7-processed-gene-0.405-mRNA-1 -            303    0.0061   16.1   0.5   1   1   1.5e-05     0.022   14.3   0.5    19    59    37    80    28   163 0.84 AAA domainFer4_NifH            PF00142.14   271 augustus_masked-scaffold_7-processed-gene-0.405-mRNA-1 -            303    0.0066   15.8   0.0   1   2    0.0001      0.15   11.3   0.0     7    30    56    79    49    97 0.86 4Fe-4S iron sulfur cluster binding proteins, NifH/frxC familyFer4_NifH            PF00142.14   271 augustus_masked-scaffold_7-processed-gene-0.405-mRNA-1 -            303    0.0066   15.8   0.0   2   2     0.091   1.3e+02    1.7   0.0   186   245   232   291   193   297 0.85 4Fe-4S iron sulfur cluster binding proteins, NifH/frxC familyCBP_BcsQ             PF06564.8    244 augustus_masked-scaffold_7-processed-gene-0.405-mRNA-1 -            303     0.087   12.2   0.5   1   2   0.00016      0.24   10.8   0.2     3    37    50    84    48    89 0.88 Cellulose biosynthesis protein BcsQCBP_BcsQ             PF06564.8    244 augustus_masked-scaffold_7-processed-gene-0.405-mRNA-1 -            303     0.087   12.2   0.5   2   2       0.8   1.2e+03   -1.3   0.0   110   146   149   186   126   194 0.58 Cellulose biosynthesis protein BcsQKinocilin            PF15033.2    123 augustus_masked-scaffold_7-processed-gene-0.405-mRNA-1 -            303      0.13   12.0   0.3   1   3       3.7   5.5e+03   -2.9   0.0    32    52    52    71    48    94 0.56 Kinocilin proteinKinocilin            PF15033.2    123 augustus_masked-scaffold_7-processed-gene-0.405-mRNA-1 -            303      0.13   12.0   0.3   2   3      0.67   9.8e+02   -0.5   0.0    14    44   171   202   168   223 0.66 Kinocilin proteinKinocilin            PF15033.2    123 augustus_masked-scaffold_7-processed-gene-0.405-mRNA-1 -            303      0.13   12.0   0.3   3   3    0.0006      0.89    9.3   0.0    63   108   241   287   235   300 0.83 Kinocilin proteinAAA_19               PF13245.2     76 augustus_masked-scaffold_7-processed-gene-0.405-mRNA-1 -            303      0.25   11.2   1.4   1   2     0.058        86    3.0   0.1    19    36    58    75    48    83 0.77 Part of AAA domainAAA_19               PF13245.2     76 augustus_masked-scaffold_7-processed-gene-0.405-mRNA-1 -            303      0.25   11.2   1.4   2   2     0.004       5.9    6.8   0.1    11    27   157   173   142   201 0.82 Part of AAA domainVAD1-2               PF15310.2    249 maker-scaffold_7-augustus-gene-0.1344-mRNA-1 -            439       2.3    7.9  12.4   1   2     0.023   1.9e+02    1.7   6.3    40    98    88   133    74   155 0.49 Vitamin A-deficiency (VAD) rat model signallingVAD1-2               PF15310.2    249 maker-scaffold_7-augustus-gene-0.1344-mRNA-1 -            439       2.3    7.9  12.4   2   2   7.2e-05      0.58    9.9   0.3    76   122   295   341   262   369 0.89 Vitamin A-deficiency (VAD) rat model signallingPresenilin           PF01080.13   399 maker-scaffold_7-augustus-gene-0.1344-mRNA-1 -            439       3.7    6.1   5.0   1   1   0.00061         5    5.6   5.0   272   310    72   125    25   189 0.52 PresenilinGlyco_hydro_2_N      PF02837.14   171 snap_masked-scaffold_7-processed-gene-0.976-mRNA-1 -            646     1e-22   80.5   0.1   1   1   6.9e-26   2.8e-22   79.1   0.1     3   171    83   241    81   241 0.87 Glycosyl hydrolases family 2, sugar binding domainGlyco_hydro_2        PF00703.17   109 snap_masked-scaffold_7-processed-gene-0.976-mRNA-1 -            646   8.3e-10   39.2   0.3   1   2   6.1e-13   2.5e-09   37.6   0.0    17   109   255   331   242   331 0.75 Glycosyl hydrolases family 2Glyco_hydro_2        PF00703.17   109 snap_masked-scaffold_7-processed-gene-0.976-mRNA-1 -            646   8.3e-10   39.2   0.3   2   2       1.1   4.6e+03   -1.8   0.0    69    87   611   631   544   633 0.62 Glycosyl hydrolases family 2Glyco_hydro_2_C      PF02836.13   302 snap_masked-scaffold_7-processed-gene-0.976-mRNA-1 -            646   9.5e-10   37.9   0.1   1   1   1.5e-12   6.3e-09   35.2   0.1    36   166   373   494   370   629 0.82 Glycosyl hydrolases family 2, TIM barrel domainMG1                  PF11974.4    103 snap_masked-scaffold_7-processed-gene-0.976-mRNA-1 -            646     0.062   13.2   0.1   1   2     4e-05      0.16   11.8   0.0    16    75   257   316   249   334 0.81 Alpha-2-macroglobulin MG1 domainMG1                  PF11974.4    103 snap_masked-scaffold_7-processed-gene-0.976-mRNA-1 -            646     0.062   13.2   0.1   2   2       2.2     9e+03   -3.4   0.0    42    54   592   604   587   605 0.77 Alpha-2-macroglobulin MG1 domainSelR                 PF01641.14   122 snap_masked-scaffold_7-processed-gene-0.1012-mRNA-1 -            128   1.9e-43  147.3   0.0   1   1   7.8e-47   2.1e-43  147.1   0.0     8   121     8   122     2   123 0.95 SelR domainDZR                  PF12773.3     50 snap_masked-scaffold_7-processed-gene-0.1012-mRNA-1 -            128     0.024   14.6   3.0   1   2   0.00074         2    8.4   0.2    28    50    35    59    14    59 0.80 Double zinc ribbonDZR                  PF12773.3     50 snap_masked-scaffold_7-processed-gene-0.1012-mRNA-1 -            128     0.024   14.6   3.0   2   2     0.004        11    6.1   0.1    29    38    85    94    67   101 0.69 Double zinc ribbonYippee-Mis18         PF03226.10    96 snap_masked-scaffold_7-processed-gene-0.1012-mRNA-1 -            128     0.025   14.7   0.4   1   1   2.2e-05     0.061   13.5   0.4     4    72    39   100    37   122 0.79 Yippee zinc-binding/DNA-binding /Mis18, centromere assemblyzinc_ribbon_10       PF10058.5     54 snap_masked-scaffold_7-processed-gene-0.1012-mRNA-1 -            128     0.052   13.2   0.2   1   2      0.27   7.2e+02   -0.1   0.1    40    51    33    44    25    46 0.73 Predicted integral membrane zinc-ribbon metal-binding proteinzinc_ribbon_10       PF10058.5     54 snap_masked-scaffold_7-processed-gene-0.1012-mRNA-1 -            128     0.052   13.2   0.2   2   2   9.4e-05      0.25   11.0   0.0    13    39    76   102    64   115 0.80 Predicted integral membrane zinc-ribbon metal-binding proteinTF_Zn_Ribbon         PF08271.8     43 snap_masked-scaffold_7-processed-gene-0.1012-mRNA-1 -            128      0.19   11.1   1.5   1   2     0.062   1.7e+02    1.7   0.1    15    25    33    43    26    47 0.83 TFIIB zinc-bindingTF_Zn_Ribbon         PF08271.8     43 snap_masked-scaffold_7-processed-gene-0.1012-mRNA-1 -            128      0.19   11.1   1.5   2   2   0.00069       1.9    7.9   0.3    17    26    83    92    76    95 0.89 TFIIB zinc-bindingzf-Mss51             PF13824.2     56 snap_masked-scaffold_7-processed-gene-0.1012-mRNA-1 -            128      0.32   11.0   4.2   1   3    0.0031       8.3    6.5   0.2    11    32    33    54    23    57 0.80 Zinc-finger of mitochondrial splicing suppressor 51zf-Mss51             PF13824.2     56 snap_masked-scaffold_7-processed-gene-0.1012-mRNA-1 -            128      0.32   11.0   4.2   2   3     0.088   2.4e+02    1.8   0.1    20    27    57    64    55    67 0.83 Zinc-finger of mitochondrial splicing suppressor 51zf-Mss51             PF13824.2     56 snap_masked-scaffold_7-processed-gene-0.1012-mRNA-1 -            128      0.32   11.0   4.2   3   3     0.048   1.3e+02    2.6   0.0    11    22    81    92    70    97 0.76 Zinc-finger of mitochondrial splicing suppressor 51Sel1                 PF08238.8     38 augustus_masked-scaffold_7-processed-gene-0.346-mRNA-1 -            780   1.8e-40  136.3  26.3   1   7   2.6e-07    0.0042   17.6   0.4     1    38   476   513   476   513 0.87 Sel1 repeatSel1                 PF08238.8     38 augustus_masked-scaffold_7-processed-gene-0.346-mRNA-1 -            780   1.8e-40  136.3  26.3   2   7   2.2e-08   0.00036   21.0   0.4     1    36   514   547   514   549 0.93 Sel1 repeatSel1                 PF08238.8     38 augustus_masked-scaffold_7-processed-gene-0.346-mRNA-1 -            780   1.8e-40  136.3  26.3   3   7   5.4e-07    0.0088   16.6   0.3     2    37   551   584   550   585 0.91 Sel1 repeatSel1                 PF08238.8     38 augustus_masked-scaffold_7-processed-gene-0.346-mRNA-1 -            780   1.8e-40  136.3  26.3   4   7   9.8e-11   1.6e-06   28.4   0.1     2    38   590   626   589   626 0.85 Sel1 repeatSel1                 PF08238.8     38 augustus_masked-scaffold_7-processed-gene-0.346-mRNA-1 -            780   1.8e-40  136.3  26.3   5   7   1.9e-08   0.00031   21.2   0.0     1    38   627   663   627   663 0.94 Sel1 repeatSel1                 PF08238.8     38 augustus_masked-scaffold_7-processed-gene-0.346-mRNA-1 -            780   1.8e-40  136.3  26.3   6   7   7.6e-09   0.00012   22.5   0.3     1    37   664   700   664   701 0.87 Sel1 repeatSel1                 PF08238.8     38 augustus_masked-scaffold_7-processed-gene-0.346-mRNA-1 -            780   1.8e-40  136.3  26.3   7   7   2.5e-09     4e-05   24.0   0.1     2    38   703   737   702   737 0.94 Sel1 repeatBUD22                PF09073.6    429 maker-scaffold_7-augustus-gene-0.1312-mRNA-1 -            524  2.4e-101  340.1  54.7   1   2     2e-45   3.2e-41  142.0  18.1     2   233    17   240    16   255 0.79 BUD22BUD22                PF09073.6    429 maker-scaffold_7-augustus-gene-0.1312-mRNA-1 -            524  2.4e-101  340.1  54.7   2   2   3.5e-64   5.6e-60  203.8  29.5   184   429   254   524   243   524 0.76 BUD22Peptidase_M20        PF01546.24   192 maker-scaffold_7-augustus-gene-0.1351-mRNA-1 -            458   1.4e-19   70.5   0.1   1   1   4.6e-23   2.5e-19   69.8   0.1     1   190   124   448   124   450 0.89 Peptidase family M20/M25/M40M20_dimer            PF07687.10   112 maker-scaffold_7-augustus-gene-0.1351-mRNA-1 -            458   0.00061   19.6   0.1   1   1     4e-07    0.0022   17.8   0.1     4    70   260   318   258   380 0.85 Peptidase dimerisation domainPeptidase_M28        PF04389.13   198 maker-scaffold_7-augustus-gene-0.1351-mRNA-1 -            458    0.0011   18.6   0.0   1   1   3.3e-07    0.0018   17.9   0.0    15    79   123   185   116   245 0.81 Peptidase family M28Proteasome           PF00227.22   190 maker-scaffold_7-snap-gene-0.709-mRNA-1 -            253   5.4e-48  162.9   0.1   1   1     9e-52   7.3e-48  162.4   0.1     7   190    36   219    32   219 0.94 Proteasome subunitProteasome_A_N       PF10584.5     23 maker-scaffold_7-snap-gene-0.709-mRNA-1 -            253     1e-11   44.1   0.2   1   1   2.7e-15   2.2e-11   43.1   0.2     1    23     6    28     6    28 0.99 Proteasome subunit A N-terminal signatureFungal_trans         PF04082.14   267 maker-scaffold_7-augustus-gene-0.1342-mRNA-1 -            656   4.1e-13   48.8   0.0   1   2   4.4e-16   7.1e-12   44.8   0.0     4   173    85   247    82   255 0.86 Fungal specific transcription factor domainFungal_trans         PF04082.14   267 maker-scaffold_7-augustus-gene-0.1342-mRNA-1 -            656   4.1e-13   48.8   0.0   2   2    0.0061        99    1.7   0.0   164   232   260   314   257   340 0.74 Fungal specific transcription factor domainPhyH                 PF05721.9    211 maker-scaffold_7-augustus-gene-0.1300-mRNA-1 -            341   3.8e-57  193.8   0.0   1   1   2.1e-60   1.7e-56  191.6   0.0     1   210    12   309    12   310 0.94 Phytanoyl-CoA dioxygenase (PhyH)Pox_P4A              PF03395.10   882 maker-scaffold_7-augustus-gene-0.1300-mRNA-1 -            341       0.2    9.1   0.0   1   1   2.9e-05      0.24    8.8   0.0   416   467    19    77     3   113 0.72 Poxvirus P4A proteinF_actin_cap_B        PF01115.13   238 maker-scaffold_7-augustus-gene-0.1295-mRNA-1 -            307  2.4e-101  338.2   0.0   1   1  3.8e-105  3.1e-101  337.9   0.0     1   238     6   279     6   279 0.93 F-actin capping protein, beta subunitP3A                  PF08727.7     59 maker-scaffold_7-augustus-gene-0.1295-mRNA-1 -            307      0.18   11.2   0.0   1   1   4.4e-05      0.36   10.3   0.0    17    40    32    55    22    57 0.91 Poliovirus 3A protein likeLRR_8                PF13855.2     61 augustus_masked-scaffold_7-processed-gene-0.419-mRNA-1 -           1169   5.2e-32  109.2  32.8   1  10      0.37   1.2e+03   -0.8   0.0     3    23   542   562   541   564 0.82 Leucine rich repeatLRR_8                PF13855.2     61 augustus_masked-scaffold_7-processed-gene-0.419-mRNA-1 -           1169   5.2e-32  109.2  32.8   2  10   1.6e-08   5.2e-05   22.8   3.4     3    61   590   647   588   647 0.93 Leucine rich repeatLRR_8                PF13855.2     61 augustus_masked-scaffold_7-processed-gene-0.419-mRNA-1 -           1169   5.2e-32  109.2  32.8   3  10   9.6e-09   3.1e-05   23.5   0.7     2    61   660   717   659   717 0.89 Leucine rich repeatLRR_8                PF13855.2     61 augustus_masked-scaffold_7-processed-gene-0.419-mRNA-1 -           1169   5.2e-32  109.2  32.8   4  10   7.5e-05      0.24   11.0   0.2     2    41   731   771   730   776 0.86 Leucine rich repeatLRR_8                PF13855.2     61 augustus_masked-scaffold_7-processed-gene-0.419-mRNA-1 -           1169   5.2e-32  109.2  32.8   5  10   2.4e-07   0.00078   19.0   0.5     3    60   757   811   755   812 0.91 Leucine rich repeatLRR_8                PF13855.2     61 augustus_masked-scaffold_7-processed-gene-0.419-mRNA-1 -           1169   5.2e-32  109.2  32.8   6  10   1.7e-08   5.5e-05   22.7   0.1     2    61   778   835   777   835 0.91 Leucine rich repeatLRR_8                PF13855.2     61 augustus_masked-scaffold_7-processed-gene-0.419-mRNA-1 -           1169   5.2e-32  109.2  32.8   7  10   1.2e-07   0.00038   20.0   0.5     3    61   968  1027   966  1027 0.92 Leucine rich repeatLRR_8                PF13855.2     61 augustus_masked-scaffold_7-processed-gene-0.419-mRNA-1 -           1169   5.2e-32  109.2  32.8   8  10   4.6e-07    0.0015   18.1   0.7    23    61  1013  1052  1011  1052 0.84 Leucine rich repeatLRR_8                PF13855.2     61 augustus_masked-scaffold_7-processed-gene-0.419-mRNA-1 -           1169   5.2e-32  109.2  32.8   9  10   2.9e-07   0.00095   18.7   0.1     1    60  1040  1097  1040  1098 0.90 Leucine rich repeatLRR_8                PF13855.2     61 augustus_masked-scaffold_7-processed-gene-0.419-mRNA-1 -           1169   5.2e-32  109.2  32.8  10  10   6.4e-07    0.0021   17.6   0.2     1    61  1065  1124  1065  1124 0.91 Leucine rich repeatLRR_4                PF12799.3     43 augustus_masked-scaffold_7-processed-gene-0.419-mRNA-1 -           1169   8.8e-27   92.4  36.6   1  14   4.7e-06     0.015   15.5   0.9     3    39   590   624   589   632 0.85 Leucine Rich repeats (2 copies)LRR_4                PF12799.3     43 augustus_masked-scaffold_7-processed-gene-0.419-mRNA-1 -           1169   8.8e-27   92.4  36.6   2  14   0.00026      0.84   10.0   3.0     4    38   614   651   613   658 0.77 Leucine Rich repeats (2 copies)LRR_4                PF12799.3     43 augustus_masked-scaffold_7-processed-gene-0.419-mRNA-1 -           1169   8.8e-27   92.4  36.6   3  14     1e-05     0.033   14.5   0.7     1    42   635   675   635   676 0.87 Leucine Rich repeats (2 copies)LRR_4                PF12799.3     43 augustus_masked-scaffold_7-processed-gene-0.419-mRNA-1 -           1169   8.8e-27   92.4  36.6   4  14   3.7e-07    0.0012   19.0   0.9     2    41   660   697   659   704 0.83 Leucine Rich repeats (2 copies)LRR_4                PF12799.3     43 augustus_masked-scaffold_7-processed-gene-0.419-mRNA-1 -           1169   8.8e-27   92.4  36.6   5  14    0.0011       3.4    8.1   1.6     1    26   682   711   682   726 0.76 Leucine Rich repeats (2 copies)LRR_4                PF12799.3     43 augustus_masked-scaffold_7-processed-gene-0.419-mRNA-1 -           1169   8.8e-27   92.4  36.6   6  14   0.00021      0.68   10.3   0.0     4    38   708   745   706   750 0.65 Leucine Rich repeats (2 copies)LRR_4                PF12799.3     43 augustus_masked-scaffold_7-processed-gene-0.419-mRNA-1 -           1169   8.8e-27   92.4  36.6   7  14    0.0036        12    6.4   0.1     3    39   757   793   755   799 0.78 Leucine Rich repeats (2 copies)LRR_4                PF12799.3     43 augustus_masked-scaffold_7-processed-gene-0.419-mRNA-1 -           1169   8.8e-27   92.4  36.6   8  14   0.00023      0.75   10.2   0.0     2    40   778   814   777   818 0.76 Leucine Rich repeats (2 copies)LRR_4                PF12799.3     43 augustus_masked-scaffold_7-processed-gene-0.419-mRNA-1 -           1169   8.8e-27   92.4  36.6   9  14   9.4e-05      0.31   11.4   0.0     2    39   801   836   797   844 0.82 Leucine Rich repeats (2 copies)LRR_4                PF12799.3     43 augustus_masked-scaffold_7-processed-gene-0.419-mRNA-1 -           1169   8.8e-27   92.4  36.6  10  14   5.2e-07    0.0017   18.6   1.4     3    42   992  1034   968  1035 0.88 Leucine Rich repeats (2 copies)LRR_4                PF12799.3     43 augustus_masked-scaffold_7-processed-gene-0.419-mRNA-1 -           1169   8.8e-27   92.4  36.6  11  14   3.5e-09   1.1e-05   25.5   1.3     1    41  1015  1055  1015  1060 0.86 Leucine Rich repeats (2 copies)LRR_4                PF12799.3     43 augustus_masked-scaffold_7-processed-gene-0.419-mRNA-1 -           1169   8.8e-27   92.4  36.6  12  14   1.5e-06     0.005   17.1   0.1     1    39  1040  1081  1040  1087 0.83 Leucine Rich repeats (2 copies)LRR_4                PF12799.3     43 augustus_masked-scaffold_7-processed-gene-0.419-mRNA-1 -           1169   8.8e-27   92.4  36.6  13  14   7.6e-05      0.25   11.7   0.8     1    38  1065  1098  1065  1111 0.86 Leucine Rich repeats (2 copies)LRR_4                PF12799.3     43 augustus_masked-scaffold_7-processed-gene-0.419-mRNA-1 -           1169   8.8e-27   92.4  36.6  14  14    0.0034        11    6.5   0.1     3    33  1088  1122  1086  1128 0.81 Leucine Rich repeats (2 copies)LRR_1                PF00560.29    23 augustus_masked-scaffold_7-processed-gene-0.419-mRNA-1 -           1169   2.4e-08   33.2  25.1   1  13         2   6.6e+03   -1.6   0.0     2    12   590   600   589   607 0.82 Leucine Rich RepeatLRR_1                PF00560.29    23 augustus_masked-scaffold_7-processed-gene-0.419-mRNA-1 -           1169   2.4e-08   33.2  25.1   2  13     0.096   3.1e+02    2.4   0.1     3    17   614   628   612   634 0.75 Leucine Rich RepeatLRR_1                PF00560.29    23 augustus_masked-scaffold_7-processed-gene-0.419-mRNA-1 -           1169   2.4e-08   33.2  25.1   3  13       2.5   8.2e+03   -1.9   0.1     1    22   636   657   636   658 0.81 Leucine Rich RepeatLRR_1                PF00560.29    23 augustus_masked-scaffold_7-processed-gene-0.419-mRNA-1 -           1169   2.4e-08   33.2  25.1   4  13     0.055   1.8e+02    3.2   0.0     1    18   660   677   660   678 0.86 Leucine Rich RepeatLRR_1                PF00560.29    23 augustus_masked-scaffold_7-processed-gene-0.419-mRNA-1 -           1169   2.4e-08   33.2  25.1   5  13    0.0034        11    6.8   0.3     1    20   683   702   683   711 0.87 Leucine Rich RepeatLRR_1                PF00560.29    23 augustus_masked-scaffold_7-processed-gene-0.419-mRNA-1 -           1169   2.4e-08   33.2  25.1   6  13     0.044   1.4e+02    3.4   0.1     1    17   731   747   731   773 0.81 Leucine Rich RepeatLRR_1                PF00560.29    23 augustus_masked-scaffold_7-processed-gene-0.419-mRNA-1 -           1169   2.4e-08   33.2  25.1   7  13         5   1.6e+04   -3.2   0.0     1    12   801   812   801   817 0.79 Leucine Rich RepeatLRR_1                PF00560.29    23 augustus_masked-scaffold_7-processed-gene-0.419-mRNA-1 -           1169   2.4e-08   33.2  25.1   8  13    0.0031        10    6.9   0.0     1    19   824   842   824   849 0.85 Leucine Rich RepeatLRR_1                PF00560.29    23 augustus_masked-scaffold_7-processed-gene-0.419-mRNA-1 -           1169   2.4e-08   33.2  25.1   9  13       2.5   8.1e+03   -1.9   0.0     2    12   992  1002   991  1006 0.86 Leucine Rich RepeatLRR_1                PF00560.29    23 augustus_masked-scaffold_7-processed-gene-0.419-mRNA-1 -           1169   2.4e-08   33.2  25.1  10  13    0.0016       5.1    7.9   0.2     1    17  1016  1032  1016  1039 0.89 Leucine Rich RepeatLRR_1                PF00560.29    23 augustus_masked-scaffold_7-processed-gene-0.419-mRNA-1 -           1169   2.4e-08   33.2  25.1  11  13     0.083   2.7e+02    2.6   0.0     1    20  1041  1060  1041  1064 0.84 Leucine Rich RepeatLRR_1                PF00560.29    23 augustus_masked-scaffold_7-processed-gene-0.419-mRNA-1 -           1169   2.4e-08   33.2  25.1  12  13      0.15   4.8e+02    1.8   0.1     2    14  1088  1100  1066  1108 0.80 Leucine Rich RepeatLRR_1                PF00560.29    23 augustus_masked-scaffold_7-processed-gene-0.419-mRNA-1 -           1169   2.4e-08   33.2  25.1  13  13     0.087   2.8e+02    2.5   0.0     2    13  1114  1125  1113  1141 0.81 Leucine Rich RepeatLRR_9                PF14580.2    175 augustus_masked-scaffold_7-processed-gene-0.419-mRNA-1 -           1169    0.0014   18.3   6.2   1   3     0.014        46    3.6   0.5    43   120   589   666   581   687 0.73 Leucine-rich repeatLRR_9                PF14580.2    175 augustus_masked-scaffold_7-processed-gene-0.419-mRNA-1 -           1169    0.0014   18.3   6.2   2   3    0.0015         5    6.7   0.1    64   115   730   782   716   845 0.55 Leucine-rich repeatLRR_9                PF14580.2    175 augustus_masked-scaffold_7-processed-gene-0.419-mRNA-1 -           1169    0.0014   18.3   6.2   3   3   0.00067       2.2    7.9   0.0    22    79  1018  1080  1008  1102 0.74 Leucine-rich repeatLRR_6                PF13516.2     20 augustus_masked-scaffold_7-processed-gene-0.419-mRNA-1 -           1169      0.77   10.7  10.6   1   7      0.25   8.2e+02    1.5   0.1     1    18   610   630   610   633 0.73 Leucine Rich repeatLRR_6                PF13516.2     20 augustus_masked-scaffold_7-processed-gene-0.419-mRNA-1 -           1169      0.77   10.7  10.6   2   7      0.93     3e+03   -0.2   0.2     3     3   660   660   634   675 0.53 Leucine Rich repeatLRR_6                PF13516.2     20 augustus_masked-scaffold_7-processed-gene-0.419-mRNA-1 -           1169      0.77   10.7  10.6   3   7      0.21   6.9e+02    1.8   0.0     1    16   681   695   681   704 0.68 Leucine Rich repeatLRR_6                PF13516.2     20 augustus_masked-scaffold_7-processed-gene-0.419-mRNA-1 -           1169      0.77   10.7  10.6   4   7       1.6   5.2e+03   -0.9   0.0     3    10   731   740   729   751 0.75 Leucine Rich repeatLRR_6                PF13516.2     20 augustus_masked-scaffold_7-processed-gene-0.419-mRNA-1 -           1169      0.77   10.7  10.6   5   7      0.15   4.9e+02    2.2   0.0     2    19   823   844   822   845 0.80 Leucine Rich repeatLRR_6                PF13516.2     20 augustus_masked-scaffold_7-processed-gene-0.419-mRNA-1 -           1169      0.77   10.7  10.6   6   7     0.064   2.1e+02    3.3   0.1     1    10  1014  1025  1014  1038 0.74 Leucine Rich repeatLRR_6                PF13516.2     20 augustus_masked-scaffold_7-processed-gene-0.419-mRNA-1 -           1169      0.77   10.7  10.6   7   7       3.2     1e+04   -1.8   0.0     2    10  1040  1050  1039  1056 0.75 Leucine Rich repeatDUF3801              PF12687.3    204 maker-scaffold_7-augustus-gene-0.1294-mRNA-1 -            466    0.0088   15.7   6.6   1   2       2.2     2e+03   -1.8   0.1   126   126    50    50     4   127 0.59 Protein of unknown function (DUF3801)DUF3801              PF12687.3    204 maker-scaffold_7-augustus-gene-0.1294-mRNA-1 -            466    0.0088   15.7   6.6   2   2   9.7e-06    0.0088   15.7   6.6    82   161   278   372   275   407 0.62 Protein of unknown function (DUF3801)zf-RING-like         PF08746.7     43 maker-scaffold_7-augustus-gene-0.1294-mRNA-1 -            466     0.013   15.6   0.9   1   1   3.9e-05     0.035   14.3   0.9    12    31   258   277   256   283 0.91 RING-like domainMacoilin             PF09726.5    671 maker-scaffold_7-augustus-gene-0.1294-mRNA-1 -            466      0.13   10.6   9.0   1   1    0.0003      0.27    9.6   9.0   264   352   304   391   282   426 0.50 Transmembrane proteinAndrogen_recep       PF02166.12   484 maker-scaffold_7-augustus-gene-0.1294-mRNA-1 -            466       0.2   10.3  19.3   1   2       4.1   3.7e+03   -3.7   0.0   235   263   182   210   179   216 0.82 Androgen receptorAndrogen_recep       PF02166.12   484 maker-scaffold_7-augustus-gene-0.1294-mRNA-1 -            466       0.2   10.3  19.3   2   2    0.0003      0.27    9.9  18.7    50   160   305   409   280   428 0.69 Androgen receptorDUF913               PF06025.8    367 maker-scaffold_7-augustus-gene-0.1294-mRNA-1 -            466      0.26   10.1   5.2   1   1   0.00048      0.43    9.3   5.2   248   308   284   363   254   395 0.50 Domain of Unknown Function (DUF913)Hid1                 PF12722.3    895 maker-scaffold_7-augustus-gene-0.1294-mRNA-1 -            466      0.45    8.2   8.4   1   1   0.00069      0.62    7.8   8.4   632   721   301   388   222   416 0.63 High-temperature-induced dauer-formation proteinNdc1_Nup             PF09531.6    599 maker-scaffold_7-augustus-gene-0.1294-mRNA-1 -            466       1.3    7.5   9.8   1   1    0.0023       2.1    6.8   9.8   346   439   297   404   291   453 0.47 Nucleoporin protein Ndc1-NupMembralin            PF09746.5    381 maker-scaffold_7-augustus-gene-0.1294-mRNA-1 -            466       1.3    7.8   3.8   1   1    0.0026       2.4    7.0   3.8   104   161   304   361   256   398 0.48 Tumour-associated proteinPHD                  PF00628.25    52 maker-scaffold_7-augustus-gene-0.1294-mRNA-1 -            466         2    8.2   9.7   1   1   0.00019      0.17   11.6   4.3     2    32   245   276   244   281 0.85 PHD-fingerMed15                PF09606.6    799 maker-scaffold_7-augustus-gene-0.1294-mRNA-1 -            466       2.1    6.5  29.6   1   1    0.0031       2.8    6.1  29.6   364   448   304   387   280   420 0.35 ARC105 or Med15 subunit of Mediator complex non-fungalGREB1                PF15782.1   1955 maker-scaffold_7-augustus-gene-0.1294-mRNA-1 -            466         4    4.3  12.9   1   1    0.0055         5    4.0  12.9  1187  1259   297   371   190   419 0.42 Gene regulated by oestrogen in breast cancerPAT1                 PF09770.5    847 maker-scaffold_7-augustus-gene-0.1294-mRNA-1 -            466       4.1    5.4  19.2   1   1    0.0087       7.8    4.5  19.2   219   293   300   376   279   421 0.45 Topoisomerase II-associated protein PAT1Peptidase_S64        PF08192.7    697 maker-scaffold_7-augustus-gene-0.1294-mRNA-1 -            466       4.4    5.5  11.5   1   1     0.009       8.1    4.6  11.5    28   103   307   379   280   421 0.34 Peptidase family S64Plasmodium_Vir       PF05795.7    381 maker-scaffold_7-augustus-gene-0.1294-mRNA-1 -            466       4.5    6.5   9.7   1   2      0.47   4.2e+02   -0.0   0.2   213   262     6    62     3   134 0.72 Plasmodium vivax Vir proteinPlasmodium_Vir       PF05795.7    381 maker-scaffold_7-augustus-gene-0.1294-mRNA-1 -            466       4.5    6.5   9.7   2   2   0.00074      0.67    9.2   4.5   204   288   286   374   226   409 0.61 Plasmodium vivax Vir proteinPinin_SDK_memA       PF04696.9    130 maker-scaffold_7-augustus-gene-0.1294-mRNA-1 -            466       9.1    6.2  26.0   1   2      0.16   1.4e+02    2.4   1.8    31    72    12    53     2    73 0.51 pinin/SDK/memA/ protein conserved regionPinin_SDK_memA       PF04696.9    130 maker-scaffold_7-augustus-gene-0.1294-mRNA-1 -            466       9.1    6.2  26.0   2   2    0.0014       1.3    9.0  16.7    35    96   303   360   280   373 0.51 pinin/SDK/memA/ protein conserved regionNeur_chan_memb       PF02932.12   238 maker-scaffold_7-augustus-gene-0.1294-mRNA-1 -            466       9.3    6.1  10.9   1   2       0.4   3.6e+02    0.9   0.2   179   215    22    58     2    62 0.70 Neurotransmitter-gated ion-channel transmembrane regionNeur_chan_memb       PF02932.12   238 maker-scaffold_7-augustus-gene-0.1294-mRNA-1 -            466       9.3    6.1  10.9   2   2    0.0027       2.4    8.0   5.4   110   196   304   355   196   409 0.57 Neurotransmitter-gated ion-channel transmembrane regionzf-RING_2            PF13639.2     44 maker-scaffold_7-augustus-gene-0.1294-mRNA-1 -            466       9.4    6.5   9.5   1   1   0.00066      0.59   10.3   4.1     6    32   247   276   242   285 0.67 Ring finger domainDUF4770              PF15994.1    182 maker-scaffold_7-augustus-gene-0.1294-mRNA-1 -            466       9.5    6.3  13.8   1   2      0.13   1.1e+02    2.8   0.5   105   144     6    43     1    87 0.61 Domain of unknown function (DUF4770)DUF4770              PF15994.1    182 maker-scaffold_7-augustus-gene-0.1294-mRNA-1 -            466       9.5    6.3  13.8   2   2    0.0046       4.1    7.5   7.1    77   117   304   340   291   401 0.55 Domain of unknown function (DUF4770)MOZ_SAS              PF01853.14   179 augustus_masked-scaffold_7-processed-gene-0.394-mRNA-1 -           1109     3e-75  251.7   0.0   1   1   2.3e-78   4.2e-75  251.2   0.0     1   174   582   761   582   766 0.96 MOZ/SAS familyPHD_4                PF16866.1     65 augustus_masked-scaffold_7-processed-gene-0.394-mRNA-1 -           1109     2e-11   43.6   7.8   1   1   2.7e-14   4.9e-11   42.4   7.8     2    65   191   250   190   250 0.89 PHD-fingerPHD                  PF00628.25    52 augustus_masked-scaffold_7-processed-gene-0.394-mRNA-1 -           1109   9.4e-06   25.3   9.5   1   2   5.2e-09   9.4e-06   25.3   9.5     1    51   195   251   195   252 0.86 PHD-fingerPHD                  PF00628.25    52 augustus_masked-scaffold_7-processed-gene-0.394-mRNA-1 -           1109   9.4e-06   25.3   9.5   2   2       1.2   2.1e+03   -1.4   0.1    43    50   554   561   544   563 0.72 PHD-fingerC1_1                 PF00130.18    53 augustus_masked-scaffold_7-processed-gene-0.394-mRNA-1 -           1109     0.013   15.3   1.4   1   1   2.2e-05     0.039   13.7   1.4    14    44   196   230   188   239 0.75 Phorbol esters/diacylglycerol binding domain (C1 domain)zf-HC5HC2H           PF13771.2     90 augustus_masked-scaffold_7-processed-gene-0.394-mRNA-1 -           1109      0.11   12.6   1.8   1   1   0.00016      0.29   11.3   1.8    30    86   187   246   168   251 0.76 PHD-like zinc-binding domainPHD_2                PF13831.2     36 augustus_masked-scaffold_7-processed-gene-0.394-mRNA-1 -           1109      0.13   11.7   0.9   1   1    0.0002      0.36   10.2   0.9     3    36   213   250   211   250 0.77 PHD-fingerzf-RING_9            PF13901.2    205 augustus_masked-scaffold_7-processed-gene-0.394-mRNA-1 -           1109      0.18   11.6   6.2   1   1   0.00017       0.3   10.9   6.2   155   201   193   250   190   254 0.82 Putative zinc-RING and/or ribbonzf-MIZ               PF02891.16    50 augustus_masked-scaffold_7-processed-gene-0.394-mRNA-1 -           1109      0.34   10.5   3.1   1   1    0.0004      0.72    9.5   3.1    22    49   224   251   217   252 0.91 MIZ/SP-RING zinc fingerzf-4CXXC_R1          PF10497.5     99 augustus_masked-scaffold_7-processed-gene-0.394-mRNA-1 -           1109       7.8    6.7   8.0   1   2   0.00094       1.7    8.8   4.1     7    64   194   251   189   274 0.70 Zinc-finger domain of monoamine-oxidase A repressor R1zf-4CXXC_R1          PF10497.5     99 augustus_masked-scaffold_7-processed-gene-0.394-mRNA-1 -           1109       7.8    6.7   8.0   2   2       8.9   1.6e+04   -3.9   0.1    34    67   555   565   541   578 0.61 Zinc-finger domain of monoamine-oxidase A repressor R1VHS                  PF00790.15   141 maker-scaffold_7-augustus-gene-0.1309-mRNA-1 -            724   1.5e-32  112.2   0.2   1   2   7.2e-19   1.5e-15   57.2   0.1     6    67     9    70     5    72 0.96 VHS domainVHS                  PF00790.15   141 maker-scaffold_7-augustus-gene-0.1309-mRNA-1 -            724   1.5e-32  112.2   0.2   2   2   2.4e-17   4.9e-14   52.2   0.0    67   139    95   165    93   167 0.92 VHS domainSH3_1                PF00018.24    48 maker-scaffold_7-augustus-gene-0.1309-mRNA-1 -            724   3.5e-16   58.4   0.0   1   1   4.1e-19   8.4e-16   57.2   0.0     1    47   274   318   274   319 0.97 SH3 domainSH3_9                PF14604.2     49 maker-scaffold_7-augustus-gene-0.1309-mRNA-1 -            724   1.1e-15   57.1   0.1   1   1   1.3e-18   2.7e-15   55.8   0.1     1    49   275   323   275   323 0.99 Variant SH3 domainSH3_2                PF07653.13    55 maker-scaffold_7-augustus-gene-0.1309-mRNA-1 -            724   1.1e-13   50.5   0.1   1   1     1e-16   2.1e-13   49.6   0.1     1    54   272   324   272   325 0.94 Variant SH3 domainGAT                  PF03127.10    75 maker-scaffold_7-augustus-gene-0.1309-mRNA-1 -            724     8e-10   38.8   3.4   1   3       3.8   7.7e+03   -2.8   0.0    47    63    18    38    13    39 0.75 GAT domainGAT                  PF03127.10    75 maker-scaffold_7-augustus-gene-0.1309-mRNA-1 -            724     8e-10   38.8   3.4   2   3       3.3   6.7e+03   -2.6   0.0     4    40   134   171   131   171 0.65 GAT domainGAT                  PF03127.10    75 maker-scaffold_7-augustus-gene-0.1309-mRNA-1 -            724     8e-10   38.8   3.4   3   3   5.4e-13   1.1e-09   38.4   1.7     2    74   348   419   347   420 0.93 GAT domainUIM                  PF02809.16    17 maker-scaffold_7-augustus-gene-0.1309-mRNA-1 -            724    0.0093   15.7   5.9   1   1   1.2e-05     0.024   14.4   5.9     2    16   189   203   188   203 0.96 Ubiquitin interaction motifHEAT_2               PF13646.2     88 maker-scaffold_7-augustus-gene-0.1309-mRNA-1 -            724      0.06   13.7   0.6   1   3   0.00024      0.49   10.8   0.1     8    80    22   111    15   120 0.74 HEAT repeatsHEAT_2               PF13646.2     88 maker-scaffold_7-augustus-gene-0.1309-mRNA-1 -            724      0.06   13.7   0.6   2   3      0.16   3.3e+02    1.7   0.0    14    49    92   132    84   138 0.67 HEAT repeatsHEAT_2               PF13646.2     88 maker-scaffold_7-augustus-gene-0.1309-mRNA-1 -            724      0.06   13.7   0.6   3   3       1.8   3.7e+03   -1.6   0.0    33    58   350   375   322   400 0.56 HEAT repeatsSUIM_assoc           PF16619.1     64 maker-scaffold_7-augustus-gene-0.1309-mRNA-1 -            724     0.076   13.0   0.0   1   4     0.061   1.2e+02    2.7  12.2    30    56   237   263   157   270 0.67 Unstructured region C-term to UIM in Ataxin3SUIM_assoc           PF16619.1     64 maker-scaffold_7-augustus-gene-0.1309-mRNA-1 -            724     0.076   13.0   0.0   2   4   3.7e-05     0.076   13.0   0.0    29    50   420   538   324   545 0.54 Unstructured region C-term to UIM in Ataxin3SUIM_assoc           PF16619.1     64 maker-scaffold_7-augustus-gene-0.1309-mRNA-1 -            724     0.076   13.0   0.0   3   4         8   1.6e+04   -6.4  12.7    35    60   549   574   546   579 0.60 Unstructured region C-term to UIM in Ataxin3SUIM_assoc           PF16619.1     64 maker-scaffold_7-augustus-gene-0.1309-mRNA-1 -            724     0.076   13.0   0.0   4   4       6.7   1.4e+04   -3.8   0.1    34    45   632   643   631   658 0.58 Unstructured region C-term to UIM in Ataxin3Chitin_bind_3        PF03067.11   169 augustus_masked-scaffold_7-processed-gene-0.414-mRNA-1 -            248   2.3e-14   54.2   1.0   1   1   1.8e-18   2.9e-14   53.9   1.0     1   169    18   245    18   245 0.68 Chitin binding domainCOX14                PF14880.2     59 maker-scaffold_7-augustus-gene-0.1328-mRNA-1 -            275   5.1e-19   67.7   0.2   1   2       2.3   9.3e+03   -3.6   0.5    42    55    51    64    50    66 0.60 Cytochrome oxidase c assemblyCOX14                PF14880.2     59 maker-scaffold_7-augustus-gene-0.1328-mRNA-1 -            275   5.1e-19   67.7   0.2   2   2   1.3e-22   5.1e-19   67.7   0.2     1    58    72   129    72   130 0.96 Cytochrome oxidase c assemblyPRP4                 PF08799.7     29 maker-scaffold_7-augustus-gene-0.1328-mRNA-1 -            275     0.023   14.1   0.4   1   1     1e-05      0.04   13.3   0.4    16    28    45    57    42    58 0.93 pre-mRNA processing factor 4 (PRP4) likeDUF2076              PF09849.5    258 maker-scaffold_7-augustus-gene-0.1328-mRNA-1 -            275     0.037   13.9   4.3   1   2      0.34   1.4e+03   -1.1   0.0   147   147    71    71    17   132 0.59 Uncharacterized protein conserved in bacteria (DUF2076)DUF2076              PF09849.5    258 maker-scaffold_7-augustus-gene-0.1328-mRNA-1 -            275     0.037   13.9   4.3   2   2   1.7e-05     0.069   13.0   3.3    81   141   207   268   194   273 0.65 Uncharacterized protein conserved in bacteria (DUF2076)GPHR_N               PF12537.4     68 maker-scaffold_7-augustus-gene-0.1328-mRNA-1 -            275      0.13   12.3   0.0   1   1   8.3e-05      0.34   10.9   0.0    10    55    86   129    83   141 0.77 The Golgi pH Regulator (GPHR) Family N-terminalDAHP_synth_1         PF00793.16   275 augustus_masked-scaffold_7-processed-gene-0.432-mRNA-1 -            378  9.5e-103  342.7   0.2   1   1  7.3e-107  1.2e-102  342.4   0.2     7   274    48   347    37   348 0.97 DAHP synthetase I familyHpcH_HpaI            PF03328.10   221 maker-scaffold_7-augustus-gene-0.1291-mRNA-1 -            296   2.1e-33  115.2   0.0   1   1   5.2e-37   2.8e-33  114.8   0.0     4   217    48   272    45   274 0.90 HpcH/HpaI aldolase/citrate lyase familyCENP-H               PF05837.8    104 maker-scaffold_7-augustus-gene-0.1291-mRNA-1 -            296     0.096   13.1   0.1   1   1   3.6e-05      0.19   12.1   0.1    29    93     4    69     1    70 0.82 Centromere protein H (CENP-H)DUF2156              PF09924.5    298 maker-scaffold_7-augustus-gene-0.1291-mRNA-1 -            296      0.14   11.0   0.0   1   1   3.9e-05      0.21   10.4   0.0    54   103   222   271   212   280 0.89 Uncharacterized conserved protein (DUF2156)Arylsulfotran_2      PF14269.2    299 augustus_masked-scaffold_7-processed-gene-0.434-mRNA-1 -            613   1.4e-49  169.0   0.3   1   1   2.3e-53   1.9e-49  168.5   0.3     2   298   140   429   139   430 0.91 Arylsulfotransferase (ASST)Arylsulfotrans       PF05935.7    477 augustus_masked-scaffold_7-processed-gene-0.434-mRNA-1 -            613     1e-20   73.8   0.1   1   2   7.7e-24   6.2e-20   71.2   0.0   104   440    90   414    74   424 0.81 Arylsulfotransferase (ASST)Arylsulfotrans       PF05935.7    477 augustus_masked-scaffold_7-processed-gene-0.434-mRNA-1 -            613     1e-20   73.8   0.1   2   2       0.1   8.2e+02   -1.7   0.0    77   147   476   540   453   551 0.67 Arylsulfotransferase (ASST)Metallophos          PF00149.24   197 snap_masked-scaffold_7-processed-gene-0.1018-mRNA-1 -            469   1.1e-06   29.2   1.2   1   1   1.8e-10   2.9e-06   27.8   1.2     8    92    90   188    83   364 0.59 Calcineurin-like phosphoesteraseWD40                 PF00400.28    37 maker-scaffold_7-augustus-gene-0.1297-mRNA-1 -            300   9.8e-32  108.4  14.5   1   7   2.1e-05     0.037   14.8   0.0     4    37    10    44     7    44 0.83 WD domain, G-beta repeatWD40                 PF00400.28    37 maker-scaffold_7-augustus-gene-0.1297-mRNA-1 -            300   9.8e-32  108.4  14.5   2   7   1.6e-10   2.9e-07   30.9   0.0     2    36    51    87    50    88 0.93 WD domain, G-beta repeatWD40                 PF00400.28    37 maker-scaffold_7-augustus-gene-0.1297-mRNA-1 -            300   9.8e-32  108.4  14.5   3   7   1.3e-05     0.023   15.4   0.2     3    37    94   129    92   129 0.85 WD domain, G-beta repeatWD40                 PF00400.28    37 maker-scaffold_7-augustus-gene-0.1297-mRNA-1 -            300   9.8e-32  108.4  14.5   4   7      0.16   2.9e+02    2.5   0.1    12    36   144   169   133   170 0.69 WD domain, G-beta repeatWD40                 PF00400.28    37 maker-scaffold_7-augustus-gene-0.1297-mRNA-1 -            300   9.8e-32  108.4  14.5   5   7   1.8e-08   3.2e-05   24.4   0.0     3    36   182   217   180   218 0.92 WD domain, G-beta repeatWD40                 PF00400.28    37 maker-scaffold_7-augustus-gene-0.1297-mRNA-1 -            300   9.8e-32  108.4  14.5   6   7   2.7e-08   4.8e-05   23.9   0.2     6    36   238   270   235   271 0.87 WD domain, G-beta repeatWD40                 PF00400.28    37 maker-scaffold_7-augustus-gene-0.1297-mRNA-1 -            300   9.8e-32  108.4  14.5   7   7      0.54   9.7e+02    0.8   0.0     2    17   276   291   275   298 0.71 WD domain, G-beta repeatGe1_WD40             PF16529.1    328 maker-scaffold_7-augustus-gene-0.1297-mRNA-1 -            300     1e-07   31.1   0.1   1   4   0.00014      0.25   10.2   0.0   177   214    50    88    32    97 0.83 WD40 region of Ge1, enhancer of mRNA-decapping proteinGe1_WD40             PF16529.1    328 maker-scaffold_7-augustus-gene-0.1297-mRNA-1 -            300     1e-07   31.1   0.1   2   4      0.14   2.5e+02    0.3   0.0    75    99   110   134    98   157 0.72 WD40 region of Ge1, enhancer of mRNA-decapping proteinGe1_WD40             PF16529.1    328 maker-scaffold_7-augustus-gene-0.1297-mRNA-1 -            300     1e-07   31.1   0.1   3   4    0.0031       5.5    5.7   0.0   184   217   187   221   168   233 0.81 WD40 region of Ge1, enhancer of mRNA-decapping proteinGe1_WD40             PF16529.1    328 maker-scaffold_7-augustus-gene-0.1297-mRNA-1 -            300     1e-07   31.1   0.1   4   4   0.00035      0.62    8.8   0.0   188   218   245   275   235   290 0.83 WD40 region of Ge1, enhancer of mRNA-decapping proteinNup160               PF11715.4    540 maker-scaffold_7-augustus-gene-0.1297-mRNA-1 -            300   0.00024   19.8   3.0   1   4     0.059   1.1e+02    1.1   0.0   235   257    77    99    63   118 0.83 Nucleoporin Nup120/160Nup160               PF11715.4    540 maker-scaffold_7-augustus-gene-0.1297-mRNA-1 -            300   0.00024   19.8   3.0   2   4     0.036        66    1.8   0.0   229   254   112   137    99   159 0.78 Nucleoporin Nup120/160Nup160               PF11715.4    540 maker-scaffold_7-augustus-gene-0.1297-mRNA-1 -            300   0.00024   19.8   3.0   3   4     0.026        47    2.3   0.0   229   250   201   222   183   227 0.81 Nucleoporin Nup120/160Nup160               PF11715.4    540 maker-scaffold_7-augustus-gene-0.1297-mRNA-1 -            300   0.00024   19.8   3.0   4   4   3.4e-05     0.061   11.8   0.2   228   257   253   282   243   294 0.88 Nucleoporin Nup120/160Coatomer_WDAD        PF04053.10   445 maker-scaffold_7-augustus-gene-0.1297-mRNA-1 -            300   0.00068   18.7   0.0   1   1   5.1e-07   0.00092   18.2   0.0    27   162    12   162     8   212 0.86 Coatomer WD associated regionNucleoporin_N        PF08801.7    431 maker-scaffold_7-augustus-gene-0.1297-mRNA-1 -            300    0.0036   16.1   0.3   1   3     0.001       1.9    7.1   0.0   197   238    98   139    33   156 0.67 Nup133 N terminal likeNucleoporin_N        PF08801.7    431 maker-scaffold_7-augustus-gene-0.1297-mRNA-1 -            300    0.0036   16.1   0.3   2   3     0.094   1.7e+02    0.6   0.0   196   232   186   221   151   231 0.71 Nup133 N terminal likeNucleoporin_N        PF08801.7    431 maker-scaffold_7-augustus-gene-0.1297-mRNA-1 -            300    0.0036   16.1   0.3   3   3     0.029        53    2.3   0.0    44   124   211   285   181   291 0.55 Nup133 N terminal likeUSP7_C2              PF14533.2    205 maker-scaffold_7-augustus-gene-0.1297-mRNA-1 -            300     0.004   16.8   0.0   1   2   1.2e-05     0.022   14.4   0.0    42   122    60   189    45   196 0.87 Ubiquitin-specific protease C-terminalUSP7_C2              PF14533.2    205 maker-scaffold_7-augustus-gene-0.1297-mRNA-1 -            300     0.004   16.8   0.0   2   2      0.35   6.4e+02   -0.2   0.0    64    83   265   284   253   296 0.85 Ubiquitin-specific protease C-terminalPALB2_WD40           PF16756.1    351 maker-scaffold_7-augustus-gene-0.1297-mRNA-1 -            300    0.0069   15.2   3.1   1   3      0.85   1.5e+03   -2.4   0.0   194   226    76   108    70   116 0.68 Partner and localizer of BRCA2 WD40 domainPALB2_WD40           PF16756.1    351 maker-scaffold_7-augustus-gene-0.1297-mRNA-1 -            300    0.0069   15.2   3.1   2   3   6.1e-06     0.011   14.5   0.1   301   350   122   169   112   170 0.90 Partner and localizer of BRCA2 WD40 domainPALB2_WD40           PF16756.1    351 maker-scaffold_7-augustus-gene-0.1297-mRNA-1 -            300    0.0069   15.2   3.1   3   3       0.2   3.6e+02   -0.3   0.3   298   338   238   277   235   293 0.58 Partner and localizer of BRCA2 WD40 domainHPS3_N               PF14761.2    210 maker-scaffold_7-augustus-gene-0.1297-mRNA-1 -            300    0.0082   15.7   0.0   1   3   1.4e-05     0.026   14.0   0.0    12    75    12    77     9    90 0.80 Hermansky-Pudlak syndrome 3HPS3_N               PF14761.2    210 maker-scaffold_7-augustus-gene-0.1297-mRNA-1 -            300    0.0082   15.7   0.0   2   3       4.5   8.1e+03   -3.9   0.0    32    50   117   136   106   140 0.65 Hermansky-Pudlak syndrome 3HPS3_N               PF14761.2    210 maker-scaffold_7-augustus-gene-0.1297-mRNA-1 -            300    0.0082   15.7   0.0   3   3         2   3.6e+03   -2.8   0.0    23    45   150   172   136   196 0.61 Hermansky-Pudlak syndrome 3DUF3312              PF11768.4    545 maker-scaffold_7-augustus-gene-0.1297-mRNA-1 -            300     0.075   11.2   0.0   1   1   7.7e-05      0.14   10.3   0.0   264   345    65   147    57   154 0.81 Protein of unknown function (DUF3312)Sugar_tr             PF00083.20   452 maker-scaffold_7-augustus-gene-0.1366-mRNA-1 -            567   8.6e-86  288.4  20.3   1   1   1.8e-89   9.9e-86  288.2  20.3     1   452    26   505    26   505 0.93 Sugar (and other) transporterMFS_1                PF07690.12   352 maker-scaffold_7-augustus-gene-0.1366-mRNA-1 -            567   8.1e-20   70.9  42.5   1   2   1.1e-16   6.1e-13   48.3  18.5     5   251    25   328    21   340 0.61 Major Facilitator SuperfamilyMFS_1                PF07690.12   352 maker-scaffold_7-augustus-gene-0.1366-mRNA-1 -            567   8.1e-20   70.9  42.5   2   2   3.6e-09   1.9e-05   23.6  22.3    17   177   323   495   305   503 0.78 Major Facilitator SuperfamilyOATP                 PF03137.16   487 maker-scaffold_7-augustus-gene-0.1366-mRNA-1 -            567   0.00055   18.4   2.2   1   1   2.1e-07    0.0012   17.3   1.2   116   220   129   235   107   365 0.64 Organic Anion Transporter Polypeptide (OATP) familyDUF3663              PF12404.4     77 maker-scaffold_7-snap-gene-0.728-mRNA-1 -            179     0.081   12.8   0.0   1   1   1.2e-05       0.2   11.6   0.0    29    69   107   143    96   151 0.88 PeptidaseMFS_1                PF07690.12   352 maker-scaffold_7-augustus-gene-0.1337-mRNA-1 -            399   2.9e-16   59.2  32.8   1   2   1.8e-20   2.9e-16   59.2  32.8    11   342     2   325     1   335 0.83 Major Facilitator SuperfamilyMFS_1                PF07690.12   352 maker-scaffold_7-augustus-gene-0.1337-mRNA-1 -            399   2.9e-16   59.2  32.8   2   2     0.019   3.1e+02   -0.1   0.3   123   176   321   381   319   393 0.58 Major Facilitator SuperfamilyPMP22_Claudin        PF00822.16   166 augustus_masked-scaffold_7-processed-gene-0.382-mRNA-1 -            179   0.00047   19.9   1.3   1   1   2.6e-07   0.00084   19.1   1.3    18   162     1   153     1   156 0.66 PMP-22/EMP/MP20/Claudin familyMARVEL               PF01284.19   144 augustus_masked-scaffold_7-processed-gene-0.382-mRNA-1 -            179     0.032   14.1   1.8   1   1   1.4e-05     0.045   13.6   1.8    34    97    57   121     2   164 0.83 Membrane-associating domainTmemb_170            PF10190.5    103 augustus_masked-scaffold_7-processed-gene-0.382-mRNA-1 -            179     0.074   13.5   4.7   1   1   3.2e-05       0.1   13.0   4.6     2    71    58   126    57   161 0.72 Putative transmembrane protein 170DUF4118              PF13493.2    105 augustus_masked-scaffold_7-processed-gene-0.382-mRNA-1 -            179     0.074   13.3   6.3   1   2     0.003       9.7    6.5   1.8     7    56    74   121    68   134 0.79 Domain of unknown function (DUF4118)DUF4118              PF13493.2    105 augustus_masked-scaffold_7-processed-gene-0.382-mRNA-1 -            179     0.074   13.3   6.3   2   2    0.0005       1.6    9.0   0.2    40    75   138   173   129   178 0.87 Domain of unknown function (DUF4118)Cation_efflux        PF01545.17   199 augustus_masked-scaffold_7-processed-gene-0.382-mRNA-1 -            179     0.098   12.2   2.3   1   1     4e-05      0.13   11.8   2.3    32   116    67   151    64   159 0.90 Cation efflux familyPex24p               PF06398.7    366 augustus_masked-scaffold_7-processed-gene-0.435-mRNA-1 -            688   2.2e-92  309.7   0.1   1   2   9.2e-96     5e-92  308.6   0.1     3   366    57   500    55   500 0.92 Integral peroxisomal membrane peroxinPex24p               PF06398.7    366 augustus_masked-scaffold_7-processed-gene-0.435-mRNA-1 -            688   2.2e-92  309.7   0.1   2   2      0.22   1.2e+03   -2.0   0.0   188   273   592   677   589   678 0.46 Integral peroxisomal membrane peroxinDUF4613              PF15390.2    682 augustus_masked-scaffold_7-processed-gene-0.435-mRNA-1 -            688      0.06   11.6   0.0   1   1   1.6e-05     0.088   11.0   0.0   517   612   184   279   143   315 0.65 Domain of unknown function (DUF4613)DUF3306              PF11748.4    121 augustus_masked-scaffold_7-processed-gene-0.435-mRNA-1 -            688       7.3    7.3  17.4   1   3     0.018        99    3.7   1.1    19    67   185   233   168   277 0.73 Protein of unknown function (DUF3306)DUF3306              PF11748.4    121 augustus_masked-scaffold_7-processed-gene-0.435-mRNA-1 -            688       7.3    7.3  17.4   2   3     6e-05      0.33   11.7   4.7     4    77   490   562   489   572 0.64 Protein of unknown function (DUF3306)DUF3306              PF11748.4    121 augustus_masked-scaffold_7-processed-gene-0.435-mRNA-1 -            688       7.3    7.3  17.4   3   3       1.3   7.1e+03   -2.3   0.1    41    41   639   639   605   660 0.44 Protein of unknown function (DUF3306)Methyltransf_23      PF13489.2    165 maker-scaffold_7-snap-gene-0.732-mRNA-1 -            199   3.7e-07   30.1   0.0   1   1   2.1e-10   4.9e-07   29.7   0.0     6   120    42   168    37   196 0.78 Methyltransferase domainMethyltransf_31      PF13847.2    152 maker-scaffold_7-snap-gene-0.732-mRNA-1 -            199   1.1e-05   25.2   0.0   1   1   9.8e-09   2.3e-05   24.1   0.0     3   113    62   170    60   192 0.83 Methyltransferase domainMethyltransf_12      PF08242.8     99 maker-scaffold_7-snap-gene-0.732-mRNA-1 -            199   6.7e-05   23.4   0.0   1   1   4.9e-08   0.00011   22.6   0.0     2    99    68   163    67   163 0.76 Methyltransferase domainMethyltransf_18      PF12847.3    110 maker-scaffold_7-snap-gene-0.732-mRNA-1 -            199   0.00053   20.7   0.0   1   1   9.3e-07    0.0022   18.7   0.0     2   105    63   163    62   168 0.84 Methyltransferase domainMethyltransf_11      PF08241.8     95 maker-scaffold_7-snap-gene-0.732-mRNA-1 -            199     0.002   18.6   0.0   1   1   1.7e-06    0.0041   17.6   0.0     2    93    68   163    67   164 0.81 Methyltransferase domainCheR                 PF01739.14   195 maker-scaffold_7-snap-gene-0.732-mRNA-1 -            199     0.016   14.6   0.0   1   1   9.7e-06     0.022   14.1   0.0   116   172   106   164    78   179 0.89 CheR methyltransferase, SAM binding domainUbie_methyltran      PF01209.14   233 maker-scaffold_7-snap-gene-0.732-mRNA-1 -            199     0.038   13.2   0.0   1   1   3.8e-05     0.088   12.0   0.0    30   161    40   175    24   180 0.73 ubiE/COQ5 methyltransferase familyRibosomal_S14        PF00253.17    54 maker-scaffold_7-augustus-gene-0.1355-mRNA-1 -            113   6.2e-22   77.0   1.1   1   2     0.024   3.8e+02    0.8   0.1    24    48    25    49    13    54 0.80 Ribosomal protein S14p/S29eRibosomal_S14        PF00253.17    54 maker-scaffold_7-augustus-gene-0.1355-mRNA-1 -            113   6.2e-22   77.0   1.1   2   2   3.8e-26   6.2e-22   77.0   1.1     1    54    59   112    59   112 0.98 Ribosomal protein S14p/S29ePAP_assoc            PF03828.15    62 maker-scaffold_7-snap-gene-0.777-mRNA-1 -           1270   5.9e-16   58.3   0.4   1   1   1.2e-19   1.9e-15   56.7   0.4     4    61   593   645   592   646 0.93 Cid1 family poly A polymeraseRibosomal_L36e       PF01158.14    96 maker-scaffold_7-augustus-gene-0.1272-mRNA-1 -            106   8.8e-41  137.9   6.0   1   1   1.2e-44   9.9e-41  137.8   6.0     1    96     8   103     8   103 0.98 Ribosomal protein L36ePBS_linker_poly      PF00427.17   126 maker-scaffold_7-augustus-gene-0.1272-mRNA-1 -            106     0.038   13.9   0.2   1   1   5.6e-06     0.045   13.6   0.2    26    92    27    98    19   105 0.80 Phycobilisome Linker polypeptideMet_10               PF02475.12   199 snap_masked-scaffold_7-processed-gene-0.905-mRNA-1 -            474   4.6e-74  248.4   0.0   1   1   3.8e-78   6.1e-74  247.9   0.0     2   199   150   407   149   407 0.97 Met-10+ like-proteinRas                  PF00071.18   162 maker-scaffold_7-augustus-gene-0.1356-mRNA-1 -            209   6.2e-53  178.6   0.0   1   1   3.6e-56   7.3e-53  178.3   0.0     1   160    13   194    13   196 0.98 Ras familyRoc                  PF08477.9    120 maker-scaffold_7-augustus-gene-0.1356-mRNA-1 -            209   1.8e-21   76.5   0.0   1   1   1.6e-24   3.2e-21   75.6   0.0     1   120    13   127    13   127 0.87 Ras of Complex, Roc, domain of DAPkinaseArf                  PF00025.17   175 maker-scaffold_7-augustus-gene-0.1356-mRNA-1 -            209   7.1e-09   35.2   0.0   1   1   5.8e-12   1.2e-08   34.5   0.0    13   145    10   161     4   193 0.71 ADP-ribosylation factor familyGtr1_RagA            PF04670.8    232 maker-scaffold_7-augustus-gene-0.1356-mRNA-1 -            209    0.0007   18.9   0.0   1   1   5.3e-07    0.0011   18.3   0.0     1    63    13    74    13   133 0.77 Gtr1/RagA G protein conserved regionSRPRB                PF09439.6    181 maker-scaffold_7-augustus-gene-0.1356-mRNA-1 -            209    0.0022   17.3   0.0   1   1   1.5e-06     0.003   16.9   0.0     3    88    11    97     9   124 0.75 Signal recognition particle receptor beta subunitMMR_HSR1             PF01926.19   110 maker-scaffold_7-augustus-gene-0.1356-mRNA-1 -            209    0.0055   16.7   0.0   1   1   4.1e-06    0.0083   16.1   0.0     2    92    14   100    13   146 0.70 50S ribosome-binding GTPaseG-alpha              PF00503.16   377 maker-scaffold_7-augustus-gene-0.1356-mRNA-1 -            209     0.012   14.6   0.0   1   1   1.2e-05     0.024   13.6   0.0    55    96    11    53     6   105 0.79 G-protein alpha subunitABC_tran             PF00005.23   137 maker-scaffold_7-augustus-gene-0.1356-mRNA-1 -            209      0.14   12.5   0.0   1   1    0.0001      0.21   12.0   0.0    11    38    11    38     7   111 0.88 ABC transporterWD40                 PF00400.28    37 maker-scaffold_7-snap-gene-0.690-mRNA-1 -            271   1.6e-12   47.5   2.7   1   4     2e-06    0.0041   17.8   0.0     5    37    11    46     8    46 0.72 WD domain, G-beta repeatWD40                 PF00400.28    37 maker-scaffold_7-snap-gene-0.690-mRNA-1 -            271   1.6e-12   47.5   2.7   2   4       1.1   2.2e+03   -0.4   0.0    14    30    56    81    48    84 0.71 WD domain, G-beta repeatWD40                 PF00400.28    37 maker-scaffold_7-snap-gene-0.690-mRNA-1 -            271   1.6e-12   47.5   2.7   3   4   9.9e-08    0.0002   21.9   0.1     6    37   149   182   145   182 0.85 WD domain, G-beta repeatWD40                 PF00400.28    37 maker-scaffold_7-snap-gene-0.690-mRNA-1 -            271   1.6e-12   47.5   2.7   4   4     0.045        91    4.0   0.1     8    29   196   216   186   219 0.68 WD domain, G-beta repeateIF2A                PF08662.7    194 maker-scaffold_7-snap-gene-0.690-mRNA-1 -            271     0.002   17.9   0.1   1   2    0.0013       2.7    7.7   0.0   124   160    39    76    20    85 0.86 Eukaryotic translation initiation factor eIF2AeIF2A                PF08662.7    194 maker-scaffold_7-snap-gene-0.690-mRNA-1 -            271     0.002   17.9   0.1   2   2     0.003       6.1    6.5   0.0    78   157   171   252   163   263 0.83 Eukaryotic translation initiation factor eIF2ANucleoporin_N        PF08801.7    431 maker-scaffold_7-snap-gene-0.690-mRNA-1 -            271    0.0098   14.6   0.0   1   2   0.00072       1.5    7.4   0.0   197   231    14    49     5    58 0.87 Nup133 N terminal likeNucleoporin_N        PF08801.7    431 maker-scaffold_7-snap-gene-0.690-mRNA-1 -            271    0.0098   14.6   0.0   2   2    0.0041       8.4    4.9   0.1   125   231    92   185    89   211 0.81 Nup133 N terminal likeGe1_WD40             PF16529.1    328 maker-scaffold_7-snap-gene-0.690-mRNA-1 -            271     0.011   14.6   0.0   1   2   0.00067       1.4    7.7   0.0   182   224    12    57     4    75 0.75 WD40 region of Ge1, enhancer of mRNA-decapping proteinGe1_WD40             PF16529.1    328 maker-scaffold_7-snap-gene-0.690-mRNA-1 -            271     0.011   14.6   0.0   2   2    0.0054        11    4.7   0.0   256   287   167   197   144   216 0.78 WD40 region of Ge1, enhancer of mRNA-decapping proteinPQQ_2                PF13360.2    238 maker-scaffold_7-snap-gene-0.690-mRNA-1 -            271     0.012   15.1   0.3   1   2   0.00011      0.23   11.0   0.2    25   135    61   222    21   232 0.65 PQQ-like domainPQQ_2                PF13360.2    238 maker-scaffold_7-snap-gene-0.690-mRNA-1 -            271     0.012   15.1   0.3   2   2     0.015        31    4.0   0.0    62   107   193   239   156   265 0.74 PQQ-like domainCytochrom_D1         PF02239.12   368 maker-scaffold_7-snap-gene-0.690-mRNA-1 -            271     0.033   12.6   0.0   1   2    0.0098        20    3.4   0.0    53    96    36    79    29   140 0.86 Cytochrome D1 heme domainCytochrom_D1         PF02239.12   368 maker-scaffold_7-snap-gene-0.690-mRNA-1 -            271     0.033   12.6   0.0   2   2    0.0006       1.2    7.4   0.0    43   119   162   239   114   243 0.78 Cytochrome D1 heme domainNup160               PF11715.4    540 maker-scaffold_7-snap-gene-0.690-mRNA-1 -            271     0.047   12.2   0.0   1   1   2.9e-05     0.059   11.9   0.0   231   257    31    57    17   119 0.78 Nucleoporin Nup120/160DUF1513              PF07433.7    307 maker-scaffold_7-snap-gene-0.690-mRNA-1 -            271     0.056   12.4   0.0   1   2    0.0032       6.6    5.6   0.0    74   117    37    78    11   106 0.71 Protein of unknown function (DUF1513)DUF1513              PF07433.7    307 maker-scaffold_7-snap-gene-0.690-mRNA-1 -            271     0.056   12.4   0.0   2   2    0.0057        11    4.8   0.0   215   271   195   253   184   269 0.82 Protein of unknown function (DUF1513)WD40                 PF00400.28    37 maker-scaffold_7-augustus-gene-0.1348-mRNA-1 -            434   2.6e-07   31.1   3.9   1   4   5.2e-05      0.28   12.0   0.1     2    37   117   152   116   152 0.77 WD domain, G-beta repeatWD40                 PF00400.28    37 maker-scaffold_7-augustus-gene-0.1348-mRNA-1 -            434   2.6e-07   31.1   3.9   2   4     8e-06     0.043   14.5   0.1     6    37   170   207   165   207 0.88 WD domain, G-beta repeatWD40                 PF00400.28    37 maker-scaffold_7-augustus-gene-0.1348-mRNA-1 -            434   2.6e-07   31.1   3.9   3   4       2.2   1.2e+04   -2.7   0.0    18    35   229   246   224   248 0.65 WD domain, G-beta repeatWD40                 PF00400.28    37 maker-scaffold_7-augustus-gene-0.1348-mRNA-1 -            434   2.6e-07   31.1   3.9   4   4     0.029   1.5e+02    3.3   0.0    15    37   285   309   277   309 0.86 WD domain, G-beta repeatPQQ_2                PF13360.2    238 maker-scaffold_7-augustus-gene-0.1348-mRNA-1 -            434   1.1e-06   28.4   0.0   1   1     3e-09   1.6e-05   24.5   0.0    27   206    50   318    30   330 0.86 PQQ-like domainPQQ_3                PF13570.2     40 maker-scaffold_7-augustus-gene-0.1348-mRNA-1 -            434    0.0034   17.7   0.1   1   3     0.017        92    3.6   0.0    11    33    87   108    82   123 0.88 PQQ-like domainPQQ_3                PF13570.2     40 maker-scaffold_7-augustus-gene-0.1348-mRNA-1 -            434    0.0034   17.7   0.1   2   3   0.00024       1.3    9.5   0.0     4    37   205   248   204   249 0.76 PQQ-like domainPQQ_3                PF13570.2     40 maker-scaffold_7-augustus-gene-0.1348-mRNA-1 -            434    0.0034   17.7   0.1   3   3      0.51   2.8e+03   -1.1   0.0    19    33   291   305   265   310 0.76 PQQ-like domainComplex1_LYR_1       PF13232.2     61 maker-scaffold_7-snap-gene-0.762-mRNA-1 -            109   1.7e-21   76.2   2.7   1   1   7.1e-25   2.3e-21   75.8   2.7     1    61    13    73    13    73 0.98 Complex1_LYR-likeComplex1_LYR         PF05347.11    59 maker-scaffold_7-snap-gene-0.762-mRNA-1 -            109   2.7e-20   72.0   2.9   1   1   1.3e-23   4.2e-20   71.4   2.9     1    58    13    70    13    71 0.98 Complex 1 protein (LYR family)Complex1_LYR_2       PF13233.2    104 maker-scaffold_7-snap-gene-0.762-mRNA-1 -            109      0.02   15.4   0.8   1   1   7.6e-06     0.025   15.1   0.8     1    73    15    76    15    93 0.77 Complex1_LYR-likeNA37                 PF04245.9    312 maker-scaffold_7-snap-gene-0.762-mRNA-1 -            109     0.099   11.9   0.0   1   1   3.1e-05       0.1   11.9   0.0   210   245    36    71    13    90 0.86 37-kD nucleoid-associated bacterial proteinVitD-bind_III        PF09164.6     65 maker-scaffold_7-snap-gene-0.762-mRNA-1 -            109      0.15   11.9   0.1   1   1   7.9e-05      0.26   11.2   0.1     5    40    28    64    24    67 0.91 Vitamin D binding protein, domain IIIGlyco_hydro_16       PF00722.17   177 maker-scaffold_7-augustus-gene-0.1357-mRNA-1 -            493   9.5e-38  129.3   3.6   1   1     2e-41   1.6e-37  128.5   3.6     8   175    56   230    50   232 0.84 Glycosyl hydrolases family 16CcmD                 PF04995.10    44 maker-scaffold_7-augustus-gene-0.1357-mRNA-1 -            493      0.18   11.8   3.3   1   2    0.0022        18    5.4   0.1     7    25     2    20     1    23 0.89 Heme exporter protein D (CcmD)CcmD                 PF04995.10    44 maker-scaffold_7-augustus-gene-0.1357-mRNA-1 -            493      0.18   11.8   3.3   2   2   0.00098         8    6.5   0.4    12    34   309   331   309   340 0.84 Heme exporter protein D (CcmD)CK_II_beta           PF01214.14   182 maker-scaffold_7-snap-gene-0.688-mRNA-1 -            292     8e-76  253.8   0.1   1   1   6.2e-80     1e-75  253.4   0.1     2   182    16   195    15   195 0.98 Casein kinase II regulatory subunitFructosamin_kin      PF03881.10   288 snap_masked-scaffold_7-processed-gene-0.1034-mRNA-1 -            244   1.2e-29  103.3   0.0   1   1   1.8e-33   1.5e-29  103.0   0.0    58   270     4   235     1   243 0.81 Fructosamine kinaseAPH                  PF01636.19   240 snap_masked-scaffold_7-processed-gene-0.1034-mRNA-1 -            244    0.0003   20.6   0.1   1   1   6.9e-08   0.00056   19.8   0.1    43   209     8   199     2   224 0.62 Phosphotransferase enzyme familyPkinase              PF00069.21   262 maker-scaffold_7-augustus-gene-0.1339-mRNA-1 -            935   1.3e-73  247.4   0.0   1   1     8e-77   2.2e-73  246.7   0.0     1   262   656   907   656   907 0.94 Protein kinase domainPkinase_Tyr          PF07714.13   260 maker-scaffold_7-augustus-gene-0.1339-mRNA-1 -            935   2.7e-47  161.1   0.0   1   1   2.1e-50   5.6e-47  160.1   0.0     5   257   660   902   657   905 0.93 Protein tyrosine kinasePBD                  PF00786.24    59 maker-scaffold_7-augustus-gene-0.1339-mRNA-1 -            935   1.6e-18   66.7   0.0   1   3   5.9e-22   1.6e-18   66.7   0.0     2    58   300   356   299   357 0.98 P21-Rho-binding domainPBD                  PF00786.24    59 maker-scaffold_7-augustus-gene-0.1339-mRNA-1 -            935   1.6e-18   66.7   0.0   2   3       4.2   1.1e+04   -3.3   0.0    36    50   448   462   446   466 0.63 P21-Rho-binding domainPBD                  PF00786.24    59 maker-scaffold_7-augustus-gene-0.1339-mRNA-1 -            935   1.6e-18   66.7   0.0   3   3         6   1.6e+04   -4.4   1.8    29    50   588   609   586   614 0.67 P21-Rho-binding domainKinase-like          PF14531.2    288 maker-scaffold_7-augustus-gene-0.1339-mRNA-1 -            935   3.5e-08   32.9   0.0   1   1   2.2e-11     6e-08   32.1   0.0   126   241   736   841   681   850 0.68 Kinase-likeAPH                  PF01636.19   240 maker-scaffold_7-augustus-gene-0.1339-mRNA-1 -            935     0.028   14.2   3.6   1   2       1.5   4.2e+03   -2.7   3.5    72   151   529   607   502   648 0.54 Phosphotransferase enzyme familyAPH                  PF01636.19   240 maker-scaffold_7-augustus-gene-0.1339-mRNA-1 -            935     0.028   14.2   3.6   2   2   1.3e-05     0.034   13.9   0.0   155   197   758   798   722   803 0.81 Phosphotransferase enzyme familyKdo                  PF06293.10   207 maker-scaffold_7-augustus-gene-0.1339-mRNA-1 -            935     0.031   13.5   0.1   1   2      0.83   2.3e+03   -2.4   0.0   130   156   683   709   682   718 0.83 Lipopolysaccharide kinase (Kdo/WaaP) familyKdo                  PF06293.10   207 maker-scaffold_7-augustus-gene-0.1339-mRNA-1 -            935     0.031   13.5   0.1   2   2     4e-05      0.11   11.7   0.0   129   174   761   802   728   809 0.78 Lipopolysaccharide kinase (Kdo/WaaP) familyVMA21                PF09446.6     64 maker-scaffold_7-snap-gene-0.683-mRNA-1 -            107   1.5e-15   56.9   8.7   1   1   1.3e-19   2.1e-15   56.4   8.7     2    64    33    87    32    87 0.97 VMA21-like domainNTF2                 PF02136.16   120 augustus_masked-scaffold_7-processed-gene-0.392-mRNA-1 -            118   3.3e-31  108.2   0.1   1   1   6.6e-35   3.6e-31  108.1   0.1     2   119     2   114     1   115 0.96 Nuclear transport factor 2 (NTF2) domainDUF4440              PF14534.2    107 augustus_masked-scaffold_7-processed-gene-0.392-mRNA-1 -            118     0.044   14.0   0.0   1   1   9.2e-06      0.05   13.9   0.0    12    93    12    93     2   106 0.68 Domain of unknown function (DUF4440)Mtr2                 PF10429.5    164 augustus_masked-scaffold_7-processed-gene-0.392-mRNA-1 -            118     0.066   13.0   0.0   1   1   1.5e-05     0.079   12.7   0.0    16    98     6    89     1   113 0.82 Nuclear pore RNA shuttling protein Mtr2dCMP_cyt_deam_1      PF00383.18   101 augustus_masked-scaffold_7-processed-gene-0.426-mRNA-1 -            267   1.8e-08   34.0   0.0   1   1   7.2e-12   3.9e-08   33.0   0.0    23    98    35   148    24   151 0.88 Cytidine and deoxycytidylate deaminase zinc-binding regionBd3614-deam          PF14439.2    136 augustus_masked-scaffold_7-processed-gene-0.426-mRNA-1 -            267      0.06   13.3   0.1   1   1   0.00016      0.84    9.6   0.1    12    94    41   139    35   150 0.57 Bd3614-like deaminaseHerpes_pp85          PF04637.8    524 augustus_masked-scaffold_7-processed-gene-0.426-mRNA-1 -            267       3.4    5.8   6.8   1   1    0.0011       6.1    5.0   6.8   463   510    67   111    41   123 0.66 Herpesvirus phosphoprotein 85 (HHV6-7 U14/HCMV UL25)Drf_GBD              PF06371.9    191 maker-scaffold_7-augustus-gene-0.1335-mRNA-1 -            943   9.5e-14   51.3  18.0   1   2   3.5e-06    0.0047   16.4   0.0     4    53   363   412   360   457 0.82 Diaphanous GTPase-binding DomainDrf_GBD              PF06371.9    191 maker-scaffold_7-augustus-gene-0.1335-mRNA-1 -            943   9.5e-14   51.3  18.0   2   2   1.6e-13   2.1e-10   40.3   0.0    56   159   561   665   542   695 0.86 Diaphanous GTPase-binding DomainTim54                PF11711.4    370 maker-scaffold_7-augustus-gene-0.1335-mRNA-1 -            943     0.015   14.0  12.9   1   1     2e-05     0.027   13.2  12.9   191   289   100   201    81   204 0.56 Inner membrane protein import complex subunit Tim54DUF4746              PF15928.1    299 maker-scaffold_7-augustus-gene-0.1335-mRNA-1 -            943     0.037   13.3   4.8   1   1   4.4e-05     0.059   12.6   4.8    73   125    98   148    48   243 0.57 Domain of unknown function (DUF4746)Roughex              PF06020.7    365 maker-scaffold_7-augustus-gene-0.1335-mRNA-1 -            943     0.049   12.6  20.9   1   1   6.4e-05     0.086   11.8  20.9   229   351    61   179    40   191 0.67 Drosophila roughex proteinSpoIIP               PF07454.7    266 maker-scaffold_7-augustus-gene-0.1335-mRNA-1 -            943      0.87    8.8  16.2   1   1    0.0011       1.5    8.1  16.2    20    66   103   149    73   185 0.52 Stage II sporulation protein P (SpoIIP)DUF2151              PF10221.5    685 maker-scaffold_7-augustus-gene-0.1335-mRNA-1 -            943       1.5    7.1   7.8   1   1    0.0016       2.2    6.6   7.8   560   626    68   167    44   204 0.52 Cell cycle and development regulatorCPSF100_C            PF13299.2    162 maker-scaffold_7-augustus-gene-0.1335-mRNA-1 -            943         2    8.4  10.4   1   1    0.0047       6.4    6.8  10.4    32    75   105   148    49   167 0.58 Cleavage and polyadenylation factor 2 C-terminalPIH1                 PF08190.8    324 maker-scaffold_7-augustus-gene-0.1335-mRNA-1 -            943       2.2    7.9  21.0   1   2    0.0007      0.95    9.1  17.9   154   218    97   161    76   204 0.53 pre-RNA processing PIH1/Nop17PIH1                 PF08190.8    324 maker-scaffold_7-augustus-gene-0.1335-mRNA-1 -            943       2.2    7.9  21.0   2   2       1.7   2.3e+03   -2.0   0.0   130   218   369   458   366   484 0.45 pre-RNA processing PIH1/Nop17eIF-3_zeta           PF05091.8    521 maker-scaffold_7-augustus-gene-0.1335-mRNA-1 -            943       2.8    6.7  22.2   1   1    0.0045         6    5.6  22.2   107   144    93   145    47   166 0.35 Eukaryotic translation initiation factor 3 subunit 7 (eIF-3)NARP1                PF12569.4    519 maker-scaffold_7-augustus-gene-0.1335-mRNA-1 -            943       5.2    5.8  15.5   1   1    0.0065       8.8    5.0  15.5   418   465   105   152    77   163 0.49 NMDA receptor-regulated protein 1V_ATPase_I           PF01496.15   735 maker-scaffold_7-augustus-gene-0.1335-mRNA-1 -            943       6.1    4.6   6.9   1   1    0.0064       8.6    4.1   6.9    68   113    94   139    46   201 0.56 V-type ATPase 116kDa subunit familySpore_coat_CotO      PF14153.2    192 maker-scaffold_7-augustus-gene-0.1335-mRNA-1 -            943         7    6.4  27.2   1   2     0.011        14    5.4  22.4    47   109    90   151    44   178 0.54 Spore coat protein CotOSpore_coat_CotO      PF14153.2    192 maker-scaffold_7-augustus-gene-0.1335-mRNA-1 -            943         7    6.4  27.2   2   2     0.071        96    2.7   0.1    20   122   394   504   386   509 0.60 Spore coat protein CotOFungal_trans         PF04082.14   267 maker-scaffold_7-snap-gene-0.691-mRNA-1 -            893   3.7e-16   58.8   0.0   1   1   9.1e-20   7.3e-16   57.8   0.0     2   195   209   395   209   460 0.83 Fungal specific transcription factor domainZn_clus              PF00172.14    40 maker-scaffold_7-snap-gene-0.691-mRNA-1 -            893   5.3e-09   35.9  11.1   1   2   6.8e-13   5.5e-09   35.9   9.7     2    40    46    83    45    83 0.96 Fungal Zn(2)-Cys(6) binuclear cluster domainZn_clus              PF00172.14    40 maker-scaffold_7-snap-gene-0.691-mRNA-1 -            893   5.3e-09   35.9  11.1   2   2       1.2   9.8e+03   -3.3   0.0    16    34   238   255   236   259 0.73 Fungal Zn(2)-Cys(6) binuclear cluster domainzf-C2H2_4            PF13894.2     24 maker-scaffold_7-snap-gene-0.693-mRNA-1 -           1240      0.19   12.5  28.7   1   6      0.01        82    4.3   1.7     3    24   895   919   893   919 0.83 C2H2-type zinc fingerzf-C2H2_4            PF13894.2     24 maker-scaffold_7-snap-gene-0.693-mRNA-1 -           1240      0.19   12.5  28.7   2   6      0.15   1.2e+03    0.6   0.3     2    24   948   972   948   973 0.77 C2H2-type zinc fingerzf-C2H2_4            PF13894.2     24 maker-scaffold_7-snap-gene-0.693-mRNA-1 -           1240      0.19   12.5  28.7   3   6    0.0014        11    7.0   0.4     2    23  1076  1096  1076  1100 0.77 C2H2-type zinc fingerzf-C2H2_4            PF13894.2     24 maker-scaffold_7-snap-gene-0.693-mRNA-1 -           1240      0.19   12.5  28.7   4   6    0.0079        64    4.6   0.6     2    23  1103  1129  1102  1130 0.76 C2H2-type zinc fingerzf-C2H2_4            PF13894.2     24 maker-scaffold_7-snap-gene-0.693-mRNA-1 -           1240      0.19   12.5  28.7   5   6   4.8e-05      0.39   11.6   0.1     2    24  1135  1164  1134  1164 0.94 C2H2-type zinc fingerzf-C2H2_4            PF13894.2     24 maker-scaffold_7-snap-gene-0.693-mRNA-1 -           1240      0.19   12.5  28.7   6   6       1.9   1.5e+04   -2.8   0.1     3    13  1210  1217  1210  1228 0.56 C2H2-type zinc fingerzf-C2H2              PF00096.22    23 maker-scaffold_7-snap-gene-0.693-mRNA-1 -           1240       2.2    8.8  30.1   1   6      0.22   1.7e+03   -0.4   1.9     3    23   895   919   894   919 0.85 Zinc finger, C2H2 typezf-C2H2              PF00096.22    23 maker-scaffold_7-snap-gene-0.693-mRNA-1 -           1240       2.2    8.8  30.1   2   6     0.019   1.5e+02    2.9   0.1     2    23   948   972   948   972 0.92 Zinc finger, C2H2 typezf-C2H2              PF00096.22    23 maker-scaffold_7-snap-gene-0.693-mRNA-1 -           1240       2.2    8.8  30.1   3   6   0.00041       3.3    8.2   0.8     2    23  1076  1096  1076  1096 0.86 Zinc finger, C2H2 typezf-C2H2              PF00096.22    23 maker-scaffold_7-snap-gene-0.693-mRNA-1 -           1240       2.2    8.8  30.1   4   6     0.017   1.3e+02    3.1   0.4     2    23  1103  1129  1103  1129 0.85 Zinc finger, C2H2 typezf-C2H2              PF00096.22    23 maker-scaffold_7-snap-gene-0.693-mRNA-1 -           1240       2.2    8.8  30.1   5   6   0.00086         7    7.2   0.3     2    23  1135  1164  1134  1165 0.89 Zinc finger, C2H2 typezf-C2H2              PF00096.22    23 maker-scaffold_7-snap-gene-0.693-mRNA-1 -           1240       2.2    8.8  30.1   6   6     0.011        91    3.7   0.1     3    20  1210  1227  1210  1228 0.94 Zinc finger, C2H2 typeSBDS                 PF01172.14    88 augustus_masked-scaffold_7-processed-gene-0.344-mRNA-1 -            114   7.6e-23   80.3   0.1   1   1   5.3e-27   8.6e-23   80.1   0.1     3    84     9    96     7   100 0.96 Shwachman-Bodian-Diamond syndrome (SBDS) proteinRomo1                PF10247.5     66 augustus_masked-scaffold_7-processed-gene-0.467-mRNA-1 -            110   6.3e-28   96.8  14.0   1   1   4.7e-32   7.6e-28   96.5  14.0     1    66    15    80    15    80 0.98 Reactive mitochondrial oxygen species modulator 1Rad60-SLD            PF11976.4     72 augustus_masked-scaffold_7-processed-gene-0.334-mRNA-1 -            105   3.8e-21   74.5   0.1   1   1   5.7e-25   4.6e-21   74.3   0.1     5    70    27    92    24    94 0.93 Ubiquitin-2 like Rad60 SUMO-likeubiquitin            PF00240.19    69 augustus_masked-scaffold_7-processed-gene-0.334-mRNA-1 -            105   2.6e-09   36.3   0.1   1   1     4e-13   3.2e-09   36.1   0.1     3    68    30    96    28    97 0.93 Ubiquitin familyMethyltransf_23      PF13489.2    165 snap_masked-scaffold_7-processed-gene-0.934-mRNA-1 -            408   2.9e-16   59.7   0.0   1   1   2.9e-19   7.9e-16   58.3   0.0    20   158   113   362    96   373 0.84 Methyltransferase domainMethyltransf_12      PF08242.8     99 snap_masked-scaffold_7-processed-gene-0.934-mRNA-1 -            408   0.00027   21.4   0.0   1   2    0.0072        20    5.8   0.0    21    42   183   204   152   228 0.86 Methyltransferase domainMethyltransf_12      PF08242.8     99 snap_masked-scaffold_7-processed-gene-0.934-mRNA-1 -            408   0.00027   21.4   0.0   2   2   4.5e-05      0.12   12.9   0.0    54    96   274   315   257   319 0.80 Methyltransferase domainMethyltransf_11      PF08241.8     95 snap_masked-scaffold_7-processed-gene-0.934-mRNA-1 -            408   0.00027   21.3   0.0   1   3      0.39     1e+03    0.2   0.1     1    31   120   152   120   165 0.59 Methyltransferase domainMethyltransf_11      PF08241.8     95 snap_masked-scaffold_7-processed-gene-0.934-mRNA-1 -            408   0.00027   21.3   0.0   2   3   0.00061       1.7    9.2   0.0     4    41   169   205   166   250 0.80 Methyltransferase domainMethyltransf_11      PF08241.8     95 snap_masked-scaffold_7-processed-gene-0.934-mRNA-1 -            408   0.00027   21.3   0.0   3   3    0.0025       6.8    7.2   0.0    57    90   282   315   271   320 0.91 Methyltransferase domainMethyltransf_18      PF12847.3    110 snap_masked-scaffold_7-processed-gene-0.934-mRNA-1 -            408   0.00034   21.3   0.0   1   2         6   1.6e+04   -3.4   0.0     4    16   118   130   116   142 0.80 Methyltransferase domainMethyltransf_18      PF12847.3    110 snap_masked-scaffold_7-processed-gene-0.934-mRNA-1 -            408   0.00034   21.3   0.0   2   2   3.1e-07   0.00083   20.0   0.0    24   107   174   321   150   324 0.77 Methyltransferase domainMethyltransf_31      PF13847.2    152 snap_masked-scaffold_7-processed-gene-0.934-mRNA-1 -            408     0.033   13.9   0.0   1   2     0.012        33    4.1   0.0    30    47   184   201   174   205 0.87 Methyltransferase domainMethyltransf_31      PF13847.2    152 snap_masked-scaffold_7-processed-gene-0.934-mRNA-1 -            408     0.033   13.9   0.0   2   2    0.0011       2.9    7.6   0.0    66   113   280   325   271   401 0.77 Methyltransferase domainUbie_methyltran      PF01209.14   233 snap_masked-scaffold_7-processed-gene-0.934-mRNA-1 -            408     0.038   13.2   0.0   1   2     0.017        45    3.1   0.0    62    94   162   204   135   222 0.75 ubiE/COQ5 methyltransferase familyUbie_methyltran      PF01209.14   233 snap_masked-scaffold_7-processed-gene-0.934-mRNA-1 -            408     0.038   13.2   0.0   2   2   0.00058       1.6    7.9   0.0   112   159   281   329   271   370 0.83 ubiE/COQ5 methyltransferase familySeipin               PF06775.10   198 maker-scaffold_7-snap-gene-0.713-mRNA-1 -            422   2.7e-50  171.0   0.1   1   1   6.2e-54   3.4e-50  170.6   0.1     2   198    52   275    51   275 0.96 Putative adipose-regulatory protein (Seipin)Trypan_PARP          PF05887.7    143 maker-scaffold_7-snap-gene-0.713-mRNA-1 -            422    0.0032   17.4   2.6   1   1   1.3e-06    0.0072   16.2   2.6    30   106   292   367   266   400 0.65 Procyclic acidic repetitive protein (PARP)Cwf_Cwc_15           PF04889.8    223 maker-scaffold_7-snap-gene-0.713-mRNA-1 -            422      0.61    9.7   6.9   1   1   0.00019         1    9.0   6.9    95   146   313   366   292   371 0.63 Cwf15/Cwc15 cell cycle control proteingpW                  PF02831.11    67 maker-scaffold_7-snap-gene-0.696-mRNA-1 -            119     0.099   12.3   0.1   1   1     1e-05      0.17   11.6   0.1    37    56    97   116    93   119 0.84 gpWBTB                  PF00651.27   110 maker-scaffold_7-augustus-gene-0.1313-mRNA-1 -            231   3.9e-22   78.4   0.0   1   1   3.3e-26   5.3e-22   78.0   0.0     1   108    25   133    25   135 0.91 BTB/POZ domainNAD_binding_4        PF07993.8    257 augustus_masked-scaffold_7-processed-gene-0.490-mRNA-1 -           1055   3.8e-30  104.8   0.0   1   1   2.6e-33   6.9e-30  103.9   0.0     1   256   693   927   693   928 0.92 Male sterility proteinAMP-binding          PF00501.24   421 augustus_masked-scaffold_7-processed-gene-0.490-mRNA-1 -           1055   2.9e-29  101.8   0.0   1   1   1.9e-32   5.2e-29  100.9   0.0     2   334    20   352    19   367 0.84 AMP-binding enzymeEpimerase            PF01370.17   241 augustus_masked-scaffold_7-processed-gene-0.490-mRNA-1 -           1055   4.6e-10   39.2   0.0   1   2       1.8   4.8e+03   -3.4   0.0   105   124   531   549   526   559 0.78 NAD dependent epimerase/dehydratase familyEpimerase            PF01370.17   241 augustus_masked-scaffold_7-processed-gene-0.490-mRNA-1 -           1055   4.6e-10   39.2   0.0   2   2   8.3e-13   2.3e-09   37.0   0.0     1   234   691   940   691   947 0.79 NAD dependent epimerase/dehydratase familyPP-binding           PF00550.21    67 augustus_masked-scaffold_7-processed-gene-0.490-mRNA-1 -           1055   4.8e-10   39.5   0.1   1   1   4.4e-13   1.2e-09   38.2   0.1    11    67   584   645   574   645 0.84 Phosphopantetheine attachment siteRmlD_sub_bind        PF04321.13   287 augustus_masked-scaffold_7-processed-gene-0.490-mRNA-1 -           1055     0.018   14.0   0.1   1   3      0.32   8.6e+02   -1.4   0.0     3    23   691   711   690   748 0.79 RmlD substrate binding domainRmlD_sub_bind        PF04321.13   287 augustus_masked-scaffold_7-processed-gene-0.490-mRNA-1 -           1055     0.018   14.0   0.1   2   3       1.9   5.1e+03   -3.9   0.0   117   139   838   861   836   868 0.70 RmlD substrate binding domainRmlD_sub_bind        PF04321.13   287 augustus_masked-scaffold_7-processed-gene-0.490-mRNA-1 -           1055     0.018   14.0   0.1   3   3   3.4e-05     0.091   11.7   0.0   186   247   921   982   918  1003 0.87 RmlD substrate binding domainKR                   PF08659.6    180 augustus_masked-scaffold_7-processed-gene-0.490-mRNA-1 -           1055     0.088   12.6   0.0   1   2    0.0003       0.8    9.4   0.0     3    90   691   781   690   799 0.60 KR domainKR                   PF08659.6    180 augustus_masked-scaffold_7-processed-gene-0.490-mRNA-1 -           1055     0.088   12.6   0.0   2   2      0.27   7.4e+02   -0.2   0.0    67   116   942   992   932  1010 0.73 KR domainGMC_oxred_N          PF00732.15   296 maker-scaffold_7-augustus-gene-0.1340-mRNA-1 -            654   2.5e-57  194.4   0.0   1   1   1.2e-60   3.2e-57  194.0   0.0     1   295    23   343    23   344 0.90 GMC oxidoreductaseGMC_oxred_C          PF05199.9    140 maker-scaffold_7-augustus-gene-0.1340-mRNA-1 -            654   3.7e-32  111.5   0.0   1   1   2.4e-35   6.4e-32  110.8   0.0     1   140   464   601   464   601 0.92 GMC oxidoreductaseNAD_binding_8        PF13450.2     68 maker-scaffold_7-augustus-gene-0.1340-mRNA-1 -            654    0.0041   17.2   0.5   1   1   4.2e-06     0.011   15.8   0.5     1    29    27    56    27    58 0.93 NAD(P)-binding Rossmann-like domainPyr_redox_2          PF07992.10   292 maker-scaffold_7-augustus-gene-0.1340-mRNA-1 -            654     0.021   13.8   0.2   1   2   6.2e-05      0.17   10.8   0.0     1    32    23    55    23    64 0.85 Pyridine nucleotide-disulphide oxidoreductasePyr_redox_2          PF07992.10   292 maker-scaffold_7-augustus-gene-0.1340-mRNA-1 -            654     0.021   13.8   0.2   2   2      0.09   2.4e+02    0.5   0.0   188   248   271   326   227   332 0.67 Pyridine nucleotide-disulphide oxidoreductaseLycopene_cycl        PF05834.8    378 maker-scaffold_7-augustus-gene-0.1340-mRNA-1 -            654      0.04   12.9   0.1   1   1     3e-05      0.08   11.9   0.1     1    35    24    57    24    66 0.90 Lycopene cyclase proteinFAD_binding_2        PF00890.20   417 maker-scaffold_7-augustus-gene-0.1340-mRNA-1 -            654     0.054   12.4   0.5   1   2   7.4e-05       0.2   10.6   0.3     1    32    24    56    24    59 0.88 FAD binding domainFAD_binding_2        PF00890.20   417 maker-scaffold_7-augustus-gene-0.1340-mRNA-1 -            654     0.054   12.4   0.5   2   2      0.27   7.2e+02   -1.2   0.0   157   204   252   306   244   319 0.75 FAD binding domainRhoGEF               PF00621.16   180 maker-scaffold_7-snap-gene-0.681-mRNA-1 -            264     0.054   13.4   0.0   1   1   4.8e-06     0.078   12.9   0.0    40   110    10   134     4   179 0.75 RhoGEF domainOATP                 PF03137.16   487 snap_masked-scaffold_7-processed-gene-0.1028-mRNA-1 -             68     0.044   12.1   0.1   1   1   3.2e-06     0.051   11.9   0.1   293   329     6    42     3    47 0.89 Organic Anion Transporter Polypeptide (OATP) familyUCR_hinge            PF02320.12    66 snap_masked-scaffold_7-processed-gene-0.1006-mRNA-1 -             84   9.8e-24   83.2   8.1   1   1   7.4e-28   1.2e-23   82.9   8.1     9    66    26    84    20    84 0.94 Ubiquinol-cytochrome C reductase hinge proteinABC_tran             PF00005.23   137 augustus_masked-scaffold_7-processed-gene-0.575-mRNA-1 -           1628   2.6e-50  170.4   0.1   1   2   8.9e-24   6.9e-21   75.1   0.1     1   135   497   629   497   631 0.93 ABC transporterABC_tran             PF00005.23   137 augustus_masked-scaffold_7-processed-gene-0.575-mRNA-1 -           1628   2.6e-50  170.4   0.1   2   2     3e-29   2.3e-26   92.8   0.0     1   136  1278  1420  1278  1421 0.92 ABC transporterABC2_membrane_3      PF12698.3    345 augustus_masked-scaffold_7-processed-gene-0.575-mRNA-1 -           1628   8.7e-24   84.1  39.2   1   2   3.2e-13   2.5e-10   39.9   6.1   147   335   213   428    91   438 0.64 ABC-2 family transporter proteinABC2_membrane_3      PF12698.3    345 augustus_masked-scaffold_7-processed-gene-0.575-mRNA-1 -           1628   8.7e-24   84.1  39.2   2   2     1e-18   7.7e-16   58.0  16.7    63   345   929  1216   832  1216 0.69 ABC-2 family transporter proteinAAA_21               PF13304.2    303 augustus_masked-scaffold_7-processed-gene-0.575-mRNA-1 -           1628     2e-22   80.6   0.1   1   4   8.8e-07   0.00068   19.8   0.2     2    24   510   532   509   560 0.77 AAA domain, putative AbiEii toxin, Type IV TA systemAAA_21               PF13304.2    303 augustus_masked-scaffold_7-processed-gene-0.575-mRNA-1 -           1628     2e-22   80.6   0.1   2   4   1.4e-07   0.00011   22.4   0.0   204   297   594   659   545   659 0.74 AAA domain, putative AbiEii toxin, Type IV TA systemAAA_21               PF13304.2    303 augustus_masked-scaffold_7-processed-gene-0.575-mRNA-1 -           1628     2e-22   80.6   0.1   3   4   0.00039       0.3   11.1   0.0     3    21  1292  1310  1290  1329 0.75 AAA domain, putative AbiEii toxin, Type IV TA systemAAA_21               PF13304.2    303 augustus_masked-scaffold_7-processed-gene-0.575-mRNA-1 -           1628     2e-22   80.6   0.1   4   4   2.4e-07   0.00019   21.6   0.0   236   299  1392  1451  1377  1455 0.94 AAA domain, putative AbiEii toxin, Type IV TA systemAAA_29               PF13555.2     61 augustus_masked-scaffold_7-processed-gene-0.575-mRNA-1 -           1628   1.4e-11   43.8   0.3   1   3      0.64     5e+02    0.4   0.0    27    44   256   273   247   277 0.80 P-loop containing region of AAA domainAAA_29               PF13555.2     61 augustus_masked-scaffold_7-processed-gene-0.575-mRNA-1 -           1628   1.4e-11   43.8   0.3   2   3   6.8e-08   5.3e-05   22.7   0.3    20    43   505   528   496   531 0.79 P-loop containing region of AAA domainAAA_29               PF13555.2     61 augustus_masked-scaffold_7-processed-gene-0.575-mRNA-1 -           1628   1.4e-11   43.8   0.3   3   3   1.1e-05    0.0086   15.6   0.0    13    39  1279  1305  1273  1308 0.86 P-loop containing region of AAA domainSMC_N                PF02463.15   220 augustus_masked-scaffold_7-processed-gene-0.575-mRNA-1 -           1628   3.4e-08   33.1   2.3   1   4    0.0001      0.08   12.2   0.0    25    44   508   527   500   533 0.89 RecF/RecN/SMC N terminal domainSMC_N                PF02463.15   220 augustus_masked-scaffold_7-processed-gene-0.575-mRNA-1 -           1628   3.4e-08   33.1   2.3   2   4   0.00054      0.42    9.9   0.0   117   197   577   659   567   674 0.84 RecF/RecN/SMC N terminal domainSMC_N                PF02463.15   220 augustus_masked-scaffold_7-processed-gene-0.575-mRNA-1 -           1628   3.4e-08   33.1   2.3   3   4     0.015        12    5.2   0.1    25    41  1289  1305  1267  1310 0.75 RecF/RecN/SMC N terminal domainSMC_N                PF02463.15   220 augustus_masked-scaffold_7-processed-gene-0.575-mRNA-1 -           1628   3.4e-08   33.1   2.3   4   4      0.13     1e+02    2.0   0.0   136   202  1392  1454  1322  1468 0.77 RecF/RecN/SMC N terminal domainAAA_23               PF13476.2    200 augustus_masked-scaffold_7-processed-gene-0.575-mRNA-1 -           1628   1.1e-06   29.2   1.9   1   2   8.2e-06    0.0064   16.9   0.1    15    40   502   528   496   530 0.79 AAA domainAAA_23               PF13476.2    200 augustus_masked-scaffold_7-processed-gene-0.575-mRNA-1 -           1628   1.1e-06   29.2   1.9   2   2   0.00045      0.35   11.2   0.2    20    39  1289  1308  1271  1310 0.80 AAA domainAAA_16               PF13191.2    177 augustus_masked-scaffold_7-processed-gene-0.575-mRNA-1 -           1628   4.9e-06   27.0   0.6   1   2   0.00015      0.11   12.8   0.1    21    49   504   534   498   684 0.65 AAA ATPase domainAAA_16               PF13191.2    177 augustus_masked-scaffold_7-processed-gene-0.575-mRNA-1 -           1628   4.9e-06   27.0   0.6   2   2   0.00035      0.27   11.6   0.0    24   106  1288  1362  1280  1450 0.58 AAA ATPase domainDUF258               PF03193.12   167 augustus_masked-scaffold_7-processed-gene-0.575-mRNA-1 -           1628   0.00014   21.7   0.4   1   2    0.0049       3.8    7.3   0.1    98   128   506   536   486   545 0.81 Protein of unknown function, DUF258DUF258               PF03193.12   167 augustus_masked-scaffold_7-processed-gene-0.575-mRNA-1 -           1628   0.00014   21.7   0.4   2   2   0.00017      0.13   12.0   0.0    68   123  1256  1312  1241  1329 0.74 Protein of unknown function, DUF258Zeta_toxin           PF06414.8    199 augustus_masked-scaffold_7-processed-gene-0.575-mRNA-1 -           1628   0.00081   18.6   0.1   1   3       1.3   9.8e+02   -1.3   0.0   139   183    94   142    79   155 0.77 Zeta toxinZeta_toxin           PF06414.8    199 augustus_masked-scaffold_7-processed-gene-0.575-mRNA-1 -           1628   0.00081   18.6   0.1   2   3    0.0039         3    6.9   0.1    17    51   508   542   498   544 0.87 Zeta toxinZeta_toxin           PF06414.8    199 augustus_masked-scaffold_7-processed-gene-0.575-mRNA-1 -           1628   0.00081   18.6   0.1   3   3     0.003       2.3    7.3   0.0    18    53  1290  1327  1280  1333 0.86 Zeta toxinAAA_25               PF13481.2    194 augustus_masked-scaffold_7-processed-gene-0.575-mRNA-1 -           1628    0.0018   17.8   1.2   1   3    0.0022       1.7    8.1   0.0    31    55   505   529   480   601 0.77 AAA domainAAA_25               PF13481.2    194 augustus_masked-scaffold_7-processed-gene-0.575-mRNA-1 -           1628    0.0018   17.8   1.2   2   3    0.0077         6    6.3   0.0    30    50  1285  1305  1267  1327 0.87 AAA domainAAA_25               PF13481.2    194 augustus_masked-scaffold_7-processed-gene-0.575-mRNA-1 -           1628    0.0018   17.8   1.2   3   3       1.3     1e+03   -1.0   0.2    86   139  1464  1511  1454  1516 0.66 AAA domainAAA_17               PF13207.2    122 augustus_masked-scaffold_7-processed-gene-0.575-mRNA-1 -           1628    0.0021   19.0   0.1   1   2    0.0015       1.2   10.1   0.1     1    60   509   567   509   632 0.69 AAA domainAAA_17               PF13207.2    122 augustus_masked-scaffold_7-processed-gene-0.575-mRNA-1 -           1628    0.0021   19.0   0.1   2   2     0.028        22    6.1   0.0     2    64  1291  1357  1290  1442 0.68 AAA domainAAA_15               PF13175.2    405 augustus_masked-scaffold_7-processed-gene-0.575-mRNA-1 -           1628    0.0035   16.7   0.0   1   3    0.0035       2.7    7.2   0.0    22    42   507   527   471   542 0.84 AAA ATPase domainAAA_15               PF13175.2    405 augustus_masked-scaffold_7-processed-gene-0.575-mRNA-1 -           1628    0.0035   16.7   0.0   2   3     0.047        36    3.5   0.0    23    53  1289  1317  1270  1336 0.80 AAA ATPase domainAAA_15               PF13175.2    405 augustus_masked-scaffold_7-processed-gene-0.575-mRNA-1 -           1628    0.0035   16.7   0.0   3   3      0.15   1.2e+02    1.8   0.0   361   401  1412  1450  1394  1452 0.91 AAA ATPase domainAAA_30               PF13604.2    196 augustus_masked-scaffold_7-processed-gene-0.575-mRNA-1 -           1628     0.012   15.3   1.4   1   2    0.0026         2    8.0   0.2    18    48   507   537   501   555 0.77 AAA domainAAA_30               PF13604.2    196 augustus_masked-scaffold_7-processed-gene-0.575-mRNA-1 -           1628     0.012   15.3   1.4   2   2     0.023        17    5.0   0.0    19    44  1289  1314  1277  1430 0.70 AAA domainAAA_28               PF13521.2    163 augustus_masked-scaffold_7-processed-gene-0.575-mRNA-1 -           1628     0.015   15.4   0.4   1   3    0.0043       3.3    7.8   0.8     2    20   510   528   509   546 0.80 AAA domainAAA_28               PF13521.2    163 augustus_masked-scaffold_7-processed-gene-0.575-mRNA-1 -           1628     0.015   15.4   0.4   2   3       4.5   3.5e+03   -2.0   0.0    46    95   924   971   915   982 0.72 AAA domainAAA_28               PF13521.2    163 augustus_masked-scaffold_7-processed-gene-0.575-mRNA-1 -           1628     0.015   15.4   0.4   3   3     0.085        65    3.6   0.0     3    20  1292  1309  1290  1358 0.80 AAA domainAAA_22               PF13401.2    137 augustus_masked-scaffold_7-processed-gene-0.575-mRNA-1 -           1628     0.026   14.7   0.3   1   3       5.5   4.2e+03   -2.2   0.0    37    71    73   113    62   131 0.70 AAA domainAAA_22               PF13401.2    137 augustus_masked-scaffold_7-processed-gene-0.575-mRNA-1 -           1628     0.026   14.7   0.3   2   3     0.011       8.7    6.5   0.1     4    29   506   531   503   675 0.83 AAA domainAAA_22               PF13401.2    137 augustus_masked-scaffold_7-processed-gene-0.575-mRNA-1 -           1628     0.026   14.7   0.3   3   3     0.051        39    4.4   0.0     5    24  1288  1307  1285  1316 0.84 AAA domainAAA_19               PF13245.2     76 augustus_masked-scaffold_7-processed-gene-0.575-mRNA-1 -           1628     0.028   14.2   5.5   1   4      0.57   4.4e+02    0.8   0.0     6    30    72    98    67   110 0.76 Part of AAA domainAAA_19               PF13245.2     76 augustus_masked-scaffold_7-processed-gene-0.575-mRNA-1 -           1628     0.028   14.2   5.5   2   4    0.0063       4.9    7.0   0.5     8    35   506   531   500   544 0.82 Part of AAA domainAAA_19               PF13245.2     76 augustus_masked-scaffold_7-processed-gene-0.575-mRNA-1 -           1628     0.028   14.2   5.5   3   4     0.086        67    3.4   0.0    11    27  1289  1305  1283  1327 0.82 Part of AAA domainAAA_19               PF13245.2     76 augustus_masked-scaffold_7-processed-gene-0.575-mRNA-1 -           1628     0.028   14.2   5.5   4   4      0.65     5e+02    0.6   0.0    39    62  1437  1461  1405  1479 0.77 Part of AAA domainArgK                 PF03308.12   267 augustus_masked-scaffold_7-processed-gene-0.575-mRNA-1 -           1628     0.046   12.5   0.8   1   2   0.00026       0.2   10.4   0.1    19    50   497   528   485   539 0.86 ArgK proteinArgK                 PF03308.12   267 augustus_masked-scaffold_7-processed-gene-0.575-mRNA-1 -           1628     0.046   12.5   0.8   2   2      0.55   4.3e+02   -0.4   0.1    30    49  1289  1308  1279  1321 0.82 ArgK proteinAAA_10               PF12846.3    304 augustus_masked-scaffold_7-processed-gene-0.575-mRNA-1 -           1628     0.048   13.2   0.2   1   2    0.0043       3.3    7.1   0.0     4    31   510   537   507   563 0.83 AAA-like domainAAA_10               PF12846.3    304 augustus_masked-scaffold_7-processed-gene-0.575-mRNA-1 -           1628     0.048   13.2   0.2   2   2     0.063        49    3.3   0.0     5    20  1292  1307  1289  1318 0.88 AAA-like domainMukB                 PF04310.8    226 augustus_masked-scaffold_7-processed-gene-0.575-mRNA-1 -           1628     0.059   13.0   0.3   1   2     0.015        12    5.5   0.1    28    46   509   527   501   535 0.86 MukB N-terminalMukB                 PF04310.8    226 augustus_masked-scaffold_7-processed-gene-0.575-mRNA-1 -           1628     0.059   13.0   0.3   2   2     0.025        19    4.8   0.0    27    44  1289  1306  1270  1314 0.84 MukB N-terminalSRP54                PF00448.18   196 augustus_masked-scaffold_7-processed-gene-0.575-mRNA-1 -           1628     0.072   12.6   1.1   1   2     0.011       8.7    5.8   0.3     3    24   509   530   507   540 0.84 SRP54-type protein, GTPase domainSRP54                PF00448.18   196 augustus_masked-scaffold_7-processed-gene-0.575-mRNA-1 -           1628     0.072   12.6   1.1   2   2     0.017        13    5.2   0.0     3    19  1290  1306  1288  1321 0.90 SRP54-type protein, GTPase domainAAA_33               PF13671.2    143 augustus_masked-scaffold_7-processed-gene-0.575-mRNA-1 -           1628       0.1   12.6   0.0   1   2     0.041        32    4.5   0.0     2    20   510   528   509   595 0.88 AAA domainAAA_33               PF13671.2    143 augustus_masked-scaffold_7-processed-gene-0.575-mRNA-1 -           1628       0.1   12.6   0.0   2   2     0.031        24    4.9   0.0     4    39  1293  1334  1290  1383 0.73 AAA domainABC2_membrane        PF01061.20   210 augustus_masked-scaffold_7-processed-gene-0.506-mRNA-1 -           1499   2.7e-90  301.2  53.2   1   3   3.4e-46   3.1e-43  147.5  18.5     1   210   506   716   506   716 0.98 ABC-2 type transporterABC2_membrane        PF01061.20   210 augustus_masked-scaffold_7-processed-gene-0.506-mRNA-1 -           1499   2.7e-90  301.2  53.2   2   3   2.1e-51   1.9e-48  164.6  20.4     2   207  1177  1391  1176  1394 0.97 ABC-2 type transporterABC2_membrane        PF01061.20   210 augustus_masked-scaffold_7-processed-gene-0.506-mRNA-1 -           1499   2.7e-90  301.2  53.2   3   3       1.2   1.1e+03   -1.3   0.4     7    38  1458  1489  1453  1492 0.73 ABC-2 type transporterPDR_CDR              PF06422.8     98 augustus_masked-scaffold_7-processed-gene-0.506-mRNA-1 -           1499   1.5e-35  120.9   0.0   1   2   5.4e-33   4.9e-30  103.2   0.0     2    94   727   821   726   825 0.94 CDR ABC transporterPDR_CDR              PF06422.8     98 augustus_masked-scaffold_7-processed-gene-0.506-mRNA-1 -           1499   1.5e-35  120.9   0.0   2   2   3.5e-05     0.032   14.0   0.1    43    79  1461  1497  1449  1499 0.88 CDR ABC transporterABC_tran             PF00005.23   137 augustus_masked-scaffold_7-processed-gene-0.506-mRNA-1 -           1499   3.4e-31  108.4   0.0   1   3   5.9e-15   5.3e-12   46.3   0.0     5   136   183   339   179   340 0.82 ABC transporterABC_tran             PF00005.23   137 augustus_masked-scaffold_7-processed-gene-0.506-mRNA-1 -           1499   3.4e-31  108.4   0.0   2   3       4.6   4.1e+03   -1.9   0.0    85   121   479   514   437   516 0.73 ABC transporterABC_tran             PF00005.23   137 augustus_masked-scaffold_7-processed-gene-0.506-mRNA-1 -           1499   3.4e-31  108.4   0.0   3   3     9e-19   8.1e-16   58.6   0.0     1   137   875  1026   875  1026 0.90 ABC transporterABC_trans_N          PF14510.2     85 augustus_masked-scaffold_7-processed-gene-0.506-mRNA-1 -           1499   8.3e-18   64.2   0.0   1   1   2.4e-20   2.2e-17   62.9   0.0     1    84    73   155    69   156 0.89 ABC-transporter extracellular N-terminalAAA_25               PF13481.2    194 augustus_masked-scaffold_7-processed-gene-0.506-mRNA-1 -           1499   1.9e-07   30.8   0.0   1   2     0.021        19    4.6   0.0    27    55   183   211   159   251 0.87 AAA domainAAA_25               PF13481.2    194 augustus_masked-scaffold_7-processed-gene-0.506-mRNA-1 -           1499   1.9e-07   30.8   0.0   2   2   4.2e-08   3.8e-05   23.3   0.0    16   121   868   984   861  1007 0.68 AAA domainAAA_16               PF13191.2    177 augustus_masked-scaffold_7-processed-gene-0.506-mRNA-1 -           1499   1.8e-06   28.5   0.2   1   2      0.83   7.5e+02    0.3   0.0    25    45   190   210   175   248 0.79 AAA ATPase domainAAA_16               PF13191.2    177 augustus_masked-scaffold_7-processed-gene-0.506-mRNA-1 -           1499   1.8e-06   28.5   0.2   2   2   2.6e-08   2.3e-05   24.8   0.2    11   170   873  1048   869  1058 0.61 AAA ATPase domainAAA_33               PF13671.2    143 augustus_masked-scaffold_7-processed-gene-0.506-mRNA-1 -           1499   0.00023   21.2   0.0   1   2     0.018        16    5.5   0.0     2    33   192   223   191   285 0.87 AAA domainAAA_33               PF13671.2    143 augustus_masked-scaffold_7-processed-gene-0.506-mRNA-1 -           1499   0.00023   21.2   0.0   2   2   7.8e-05     0.071   13.1   0.0     2    93   888   978   887   992 0.73 AAA domainDUF258               PF03193.12   167 augustus_masked-scaffold_7-processed-gene-0.506-mRNA-1 -           1499   0.00037   20.3   0.0   1   2      0.33     3e+02    1.1   0.0   100   123   190   213   177   236 0.86 Protein of unknown function, DUF258DUF258               PF03193.12   167 augustus_masked-scaffold_7-processed-gene-0.506-mRNA-1 -           1499   0.00037   20.3   0.0   2   2     7e-06    0.0063   16.3   0.0    86   125   871   911   831   952 0.82 Protein of unknown function, DUF258AAA_29               PF13555.2     61 augustus_masked-scaffold_7-processed-gene-0.506-mRNA-1 -           1499     0.008   15.7   0.2   1   2       6.7     6e+03   -3.1   0.0    24    39   191   206   186   209 0.77 P-loop containing region of AAA domainAAA_29               PF13555.2     61 augustus_masked-scaffold_7-processed-gene-0.506-mRNA-1 -           1499     0.008   15.7   0.2   2   2   3.5e-05     0.031   13.8   0.1    23    42   886   905   876   907 0.85 P-loop containing region of AAA domainAAA_21               PF13304.2    303 augustus_masked-scaffold_7-processed-gene-0.506-mRNA-1 -           1499     0.011   15.8   0.0   1   2    0.0009      0.81    9.7   0.0     1    27   887   908   887   937 0.77 AAA domain, putative AbiEii toxin, Type IV TA systemAAA_21               PF13304.2    303 augustus_masked-scaffold_7-processed-gene-0.506-mRNA-1 -           1499     0.011   15.8   0.0   2   2     0.073        66    3.4   0.0   259   291  1017  1048   994  1057 0.79 AAA domain, putative AbiEii toxin, Type IV TA systemAAA_18               PF13238.2    129 augustus_masked-scaffold_7-processed-gene-0.506-mRNA-1 -           1499     0.013   15.9   0.1   1   2      0.23     2e+02    2.3   0.0     5    31   196   222   195   244 0.72 AAA domainAAA_18               PF13238.2    129 augustus_masked-scaffold_7-processed-gene-0.506-mRNA-1 -           1499     0.013   15.9   0.1   2   2   0.00055      0.49   10.8   0.0     3    42   890   931   889   956 0.83 AAA domaincobW                 PF02492.15   177 augustus_masked-scaffold_7-processed-gene-0.506-mRNA-1 -           1499     0.013   14.9   1.1   1   2      0.22     2e+02    1.3   0.1     4    21   193   210   190   218 0.81 CobW/HypB/UreG, nucleotide-binding domaincobW                 PF02492.15   177 augustus_masked-scaffold_7-processed-gene-0.506-mRNA-1 -           1499     0.013   14.9   1.1   2   2   0.00012      0.11   11.9   0.1     3    44   888   923   886   938 0.83 CobW/HypB/UreG, nucleotide-binding domainAAA_22               PF13401.2    137 augustus_masked-scaffold_7-processed-gene-0.506-mRNA-1 -           1499     0.014   15.6   0.1   1   2      0.28   2.5e+02    1.8   0.0     6    27   190   211   186   244 0.85 AAA domainAAA_22               PF13401.2    137 augustus_masked-scaffold_7-processed-gene-0.506-mRNA-1 -           1499     0.014   15.6   0.1   2   2    0.0005      0.45   10.6   0.0     5    43   885   914   882   988 0.75 AAA domainSMC_N                PF02463.15   220 augustus_masked-scaffold_7-processed-gene-0.506-mRNA-1 -           1499     0.014   14.7   0.0   1   3      0.19   1.8e+02    1.3   0.0   110   188    74   359    23   388 0.67 RecF/RecN/SMC N terminal domainSMC_N                PF02463.15   220 augustus_masked-scaffold_7-processed-gene-0.506-mRNA-1 -           1499     0.014   14.7   0.0   2   3      0.17   1.5e+02    1.5   0.0    26    44   887   905   876   913 0.89 RecF/RecN/SMC N terminal domainSMC_N                PF02463.15   220 augustus_masked-scaffold_7-processed-gene-0.506-mRNA-1 -           1499     0.014   14.7   0.0   3   3    0.0027       2.5    7.3   0.0   157   205  1014  1062  1005  1075 0.83 RecF/RecN/SMC N terminal domainAAA_23               PF13476.2    200 augustus_masked-scaffold_7-processed-gene-0.506-mRNA-1 -           1499      0.12   12.7   0.0   1   1   0.00027      0.24   11.7   0.0    21    39   887   905   883   936 0.94 AAA domainAAA_28               PF13521.2    163 augustus_masked-scaffold_7-processed-gene-0.506-mRNA-1 -           1499      0.15   12.1   0.2   1   2      0.15   1.3e+02    2.6   0.0     3    24   193   216   191   237 0.82 AAA domainAAA_28               PF13521.2    163 augustus_masked-scaffold_7-processed-gene-0.506-mRNA-1 -           1499      0.15   12.1   0.2   2   2    0.0064       5.8    7.0   0.0     3    26   889   913   887   951 0.85 AAA domainAAA_19               PF13245.2     76 augustus_masked-scaffold_7-processed-gene-0.506-mRNA-1 -           1499      0.18   11.6   0.8   1   2      0.18   1.7e+02    2.1   0.2    11    27   191   206   183   212 0.73 Part of AAA domainAAA_19               PF13245.2     76 augustus_masked-scaffold_7-processed-gene-0.506-mRNA-1 -           1499      0.18   11.6   0.8   2   2     0.006       5.4    6.9   0.0    11    35   886   909   878   953 0.85 Part of AAA domainABC2_membrane_3      PF12698.3    345 augustus_masked-scaffold_7-processed-gene-0.506-mRNA-1 -           1499       3.7    6.4  38.9   1   2   0.00022       0.2   10.6  14.7   205   339   600   788   513   794 0.68 ABC-2 family transporter proteinABC2_membrane_3      PF12698.3    345 augustus_masked-scaffold_7-processed-gene-0.506-mRNA-1 -           1499       3.7    6.4  38.9   2   2      0.23     2e+02    0.7  16.2   210   317  1280  1393  1191  1490 0.71 ABC-2 family transporter proteinPRP1_N               PF06424.8    138 augustus_masked-scaffold_7-processed-gene-0.617-mRNA-1 -            933   1.8e-49  167.6   8.6   1   1   6.3e-52   4.9e-49  166.2   8.6     1   138    12   173    12   173 0.86 PRP1 splicing factor, N-terminalTPR_14               PF13428.2     44 augustus_masked-scaffold_7-processed-gene-0.617-mRNA-1 -            933   8.5e-28   94.5  41.6   1  13    0.0019       1.5    9.7   0.0    13    44   278   309   274   309 0.91 Tetratricopeptide repeatTPR_14               PF13428.2     44 augustus_masked-scaffold_7-processed-gene-0.617-mRNA-1 -            933   8.5e-28   94.5  41.6   2  13    0.0004      0.31   11.8   0.0     4    40   303   339   302   343 0.94 Tetratricopeptide repeatTPR_14               PF13428.2     44 augustus_masked-scaffold_7-processed-gene-0.617-mRNA-1 -            933   8.5e-28   94.5  41.6   3  13         2   1.6e+03    0.2   0.0    20    44   350   374   344   374 0.82 Tetratricopeptide repeatTPR_14               PF13428.2     44 augustus_masked-scaffold_7-processed-gene-0.617-mRNA-1 -            933   8.5e-28   94.5  41.6   4  13     0.022        17    6.4   0.4    15    44   407   436   393   436 0.88 Tetratricopeptide repeatTPR_14               PF13428.2     44 augustus_masked-scaffold_7-processed-gene-0.617-mRNA-1 -            933   8.5e-28   94.5  41.6   5  13   0.00037      0.28   11.9   0.3    10    44   432   466   427   466 0.92 Tetratricopeptide repeatTPR_14               PF13428.2     44 augustus_masked-scaffold_7-processed-gene-0.617-mRNA-1 -            933   8.5e-28   94.5  41.6   6  13   9.1e-05     0.071   13.8   0.2     3    43   569   609   567   610 0.93 Tetratricopeptide repeatTPR_14               PF13428.2     44 augustus_masked-scaffold_7-processed-gene-0.617-mRNA-1 -            933   8.5e-28   94.5  41.6   7  13   9.6e-08   7.4e-05   23.1   0.4     2    44   602   644   601   644 0.96 Tetratricopeptide repeatTPR_14               PF13428.2     44 augustus_masked-scaffold_7-processed-gene-0.617-mRNA-1 -            933   8.5e-28   94.5  41.6   8  13   3.8e-05     0.029   15.0   0.6     3    37   637   671   635   675 0.91 Tetratricopeptide repeatTPR_14               PF13428.2     44 augustus_masked-scaffold_7-processed-gene-0.617-mRNA-1 -            933   8.5e-28   94.5  41.6   9  13   0.00014      0.11   13.2   0.2    13    44   680   711   674   711 0.91 Tetratricopeptide repeatTPR_14               PF13428.2     44 augustus_masked-scaffold_7-processed-gene-0.617-mRNA-1 -            933   8.5e-28   94.5  41.6  10  13   9.2e-06    0.0071   16.9   0.0     2    44   703   745   702   745 0.95 Tetratricopeptide repeatTPR_14               PF13428.2     44 augustus_masked-scaffold_7-processed-gene-0.617-mRNA-1 -            933   8.5e-28   94.5  41.6  11  13   0.00014      0.11   13.2   0.6     2    43   737   778   736   779 0.92 Tetratricopeptide repeatTPR_14               PF13428.2     44 augustus_masked-scaffold_7-processed-gene-0.617-mRNA-1 -            933   8.5e-28   94.5  41.6  12  13    0.0022       1.7    9.5   0.2     9    39   778   808   775   811 0.91 Tetratricopeptide repeatTPR_14               PF13428.2     44 augustus_masked-scaffold_7-processed-gene-0.617-mRNA-1 -            933   8.5e-28   94.5  41.6  13  13     0.018        14    6.6   0.0     4    39   838   875   835   881 0.77 Tetratricopeptide repeatTPR_19               PF14559.2     68 augustus_masked-scaffold_7-processed-gene-0.617-mRNA-1 -            933     4e-21   75.1  21.9   1  10    0.0062       4.8    7.6   0.0     5    50   280   325   277   329 0.92 Tetratricopeptide repeatTPR_19               PF14559.2     68 augustus_masked-scaffold_7-processed-gene-0.617-mRNA-1 -            933     4e-21   75.1  21.9   2  10       0.2   1.5e+02    2.8   0.1     4    34   406   436   405   437 0.93 Tetratricopeptide repeatTPR_19               PF14559.2     68 augustus_masked-scaffold_7-processed-gene-0.617-mRNA-1 -            933     4e-21   75.1  21.9   3  10    0.0054       4.2    7.8   0.1     7    43   439   475   436   488 0.86 Tetratricopeptide repeatTPR_19               PF14559.2     68 augustus_masked-scaffold_7-processed-gene-0.617-mRNA-1 -            933     4e-21   75.1  21.9   4  10       4.4   3.4e+03   -1.5   0.0     4    20   546   562   543   576 0.75 Tetratricopeptide repeatTPR_19               PF14559.2     68 augustus_masked-scaffold_7-processed-gene-0.617-mRNA-1 -            933     4e-21   75.1  21.9   5  10   0.00035      0.27   11.6   0.1    13    50   589   626   588   627 0.94 Tetratricopeptide repeatTPR_19               PF14559.2     68 augustus_masked-scaffold_7-processed-gene-0.617-mRNA-1 -            933     4e-21   75.1  21.9   6  10   8.8e-10   6.8e-07   29.6   0.9     2    61   612   670   611   673 0.94 Tetratricopeptide repeatTPR_19               PF14559.2     68 augustus_masked-scaffold_7-processed-gene-0.617-mRNA-1 -            933     4e-21   75.1  21.9   7  10    0.0019       1.5    9.2   0.9     5    47   682   724   680   731 0.94 Tetratricopeptide repeatTPR_19               PF14559.2     68 augustus_masked-scaffold_7-processed-gene-0.617-mRNA-1 -            933     4e-21   75.1  21.9   8  10   3.8e-05     0.029   14.7   0.0     5    61   716   772   714   777 0.95 Tetratricopeptide repeatTPR_19               PF14559.2     68 augustus_masked-scaffold_7-processed-gene-0.617-mRNA-1 -            933     4e-21   75.1  21.9   9  10     0.041        32    5.0   0.0     4    27   783   806   780   809 0.83 Tetratricopeptide repeatTPR_19               PF14559.2     68 augustus_masked-scaffold_7-processed-gene-0.617-mRNA-1 -            933     4e-21   75.1  21.9  10  10     0.011       8.7    6.8   0.0     4    40   848   884   845   911 0.82 Tetratricopeptide repeatTPR_16               PF13432.2     65 augustus_masked-scaffold_7-processed-gene-0.617-mRNA-1 -            933     9e-18   64.5  26.3   1   9     0.025        20    6.1   0.0    11    63   280   332   275   334 0.84 Tetratricopeptide repeatTPR_16               PF13432.2     65 augustus_masked-scaffold_7-processed-gene-0.617-mRNA-1 -            933     9e-18   64.5  26.3   2   9       2.2   1.7e+03   -0.1   0.1    18    38   397   417   392   438 0.50 Tetratricopeptide repeatTPR_16               PF13432.2     65 augustus_masked-scaffold_7-processed-gene-0.617-mRNA-1 -            933     9e-18   64.5  26.3   3   9      0.03        23    5.9   0.2     7    45   433   471   428   476 0.88 Tetratricopeptide repeatTPR_16               PF13432.2     65 augustus_masked-scaffold_7-processed-gene-0.617-mRNA-1 -            933     9e-18   64.5  26.3   4   9   1.2e-07   8.9e-05   23.1   1.0     1    64   605   668   605   669 0.96 Tetratricopeptide repeatTPR_16               PF13432.2     65 augustus_masked-scaffold_7-processed-gene-0.617-mRNA-1 -            933     9e-18   64.5  26.3   5   9   4.8e-07   0.00037   21.1   0.1    10    54   681   725   677   735 0.90 Tetratricopeptide repeatTPR_16               PF13432.2     65 augustus_masked-scaffold_7-processed-gene-0.617-mRNA-1 -            933     9e-18   64.5  26.3   6   9   5.9e-06    0.0046   17.6   0.1     4    63   709   768   708   770 0.91 Tetratricopeptide repeatTPR_16               PF13432.2     65 augustus_masked-scaffold_7-processed-gene-0.617-mRNA-1 -            933     9e-18   64.5  26.3   7   9    0.0019       1.5    9.6   1.0    15    62   754   801   740   803 0.88 Tetratricopeptide repeatTPR_16               PF13432.2     65 augustus_masked-scaffold_7-processed-gene-0.617-mRNA-1 -            933     9e-18   64.5  26.3   8   9     0.037        28    5.6   0.0     5    61   778   834   774   836 0.85 Tetratricopeptide repeatTPR_16               PF13432.2     65 augustus_masked-scaffold_7-processed-gene-0.617-mRNA-1 -            933     9e-18   64.5  26.3   9   9    0.0086       6.6    7.6   0.0     3    41   841   881   839   889 0.87 Tetratricopeptide repeatTPR_11               PF13414.2     68 augustus_masked-scaffold_7-processed-gene-0.617-mRNA-1 -            933   2.1e-13   50.1   9.7   1   8         2   1.6e+03   -0.8   0.0     3    42   300   340   293   357 0.78 TPR repeatTPR_11               PF13414.2     68 augustus_masked-scaffold_7-processed-gene-0.617-mRNA-1 -            933   2.1e-13   50.1   9.7   2   8    0.0025       1.9    8.5   0.1    16    46   406   436   395   454 0.74 TPR repeatTPR_11               PF13414.2     68 augustus_masked-scaffold_7-processed-gene-0.617-mRNA-1 -            933   2.1e-13   50.1   9.7   3   8      0.96   7.4e+02    0.2   0.0    20    52   440   472   438   487 0.78 TPR repeatTPR_11               PF13414.2     68 augustus_masked-scaffold_7-processed-gene-0.617-mRNA-1 -            933   2.1e-13   50.1   9.7   4   8      0.78     6e+02    0.5   0.0    15    68   545   598   534   598 0.75 TPR repeatTPR_11               PF13414.2     68 augustus_masked-scaffold_7-processed-gene-0.617-mRNA-1 -            933   2.1e-13   50.1   9.7   5   8   0.00026       0.2   11.6   0.4     5    68   603   666   599   666 0.86 TPR repeatTPR_11               PF13414.2     68 augustus_masked-scaffold_7-processed-gene-0.617-mRNA-1 -            933   2.1e-13   50.1   9.7   6   8   3.2e-06    0.0025   17.8   0.0    16    60   681   725   675   733 0.91 TPR repeatTPR_11               PF13414.2     68 augustus_masked-scaffold_7-processed-gene-0.617-mRNA-1 -            933   2.1e-13   50.1   9.7   7   8      0.36   2.8e+02    1.6   0.0    18    65   751   798   744   801 0.83 TPR repeatTPR_11               PF13414.2     68 augustus_masked-scaffold_7-processed-gene-0.617-mRNA-1 -            933   2.1e-13   50.1   9.7   8   8     0.012       9.5    6.3   0.0    13    47   845   881   826   895 0.65 TPR repeatTPR_2                PF07719.13    34 augustus_masked-scaffold_7-processed-gene-0.617-mRNA-1 -            933     8e-12   44.2  12.7   1  11       3.1   2.4e+03   -1.0   0.0     1    33   300   332   300   333 0.88 Tetratricopeptide repeatTPR_2                PF07719.13    34 augustus_masked-scaffold_7-processed-gene-0.617-mRNA-1 -            933     8e-12   44.2  12.7   2  11         7   5.4e+03   -2.1   0.1    18    32   393   407   386   409 0.74 Tetratricopeptide repeatTPR_2                PF07719.13    34 augustus_masked-scaffold_7-processed-gene-0.617-mRNA-1 -            933     8e-12   44.2  12.7   3  11       5.6   4.3e+03   -1.8   0.0    16    33   438   455   431   456 0.75 Tetratricopeptide repeatTPR_2                PF07719.13    34 augustus_masked-scaffold_7-processed-gene-0.617-mRNA-1 -            933     8e-12   44.2  12.7   4  11       2.4   1.9e+03   -0.7   0.1    13    29   545   561   540   563 0.82 Tetratricopeptide repeatTPR_2                PF07719.13    34 augustus_masked-scaffold_7-processed-gene-0.617-mRNA-1 -            933     8e-12   44.2  12.7   5  11    0.0015       1.2    9.3   0.0     3    34   603   634   601   634 0.95 Tetratricopeptide repeatTPR_2                PF07719.13    34 augustus_masked-scaffold_7-processed-gene-0.617-mRNA-1 -            933     8e-12   44.2  12.7   6  11       0.6   4.7e+02    1.2   0.2    14    34   648   668   643   668 0.88 Tetratricopeptide repeatTPR_2                PF07719.13    34 augustus_masked-scaffold_7-processed-gene-0.617-mRNA-1 -            933     8e-12   44.2  12.7   7  11     0.012       9.1    6.6   0.0    14    33   681   700   679   701 0.92 Tetratricopeptide repeatTPR_2                PF07719.13    34 augustus_masked-scaffold_7-processed-gene-0.617-mRNA-1 -            933     8e-12   44.2  12.7   8  11   0.00013     0.097   12.7   0.0     1    32   702   733   702   735 0.89 Tetratricopeptide repeatTPR_2                PF07719.13    34 augustus_masked-scaffold_7-processed-gene-0.617-mRNA-1 -            933     8e-12   44.2  12.7   9  11      0.39     3e+02    1.8   0.0    12    33   781   802   778   803 0.88 Tetratricopeptide repeatTPR_2                PF07719.13    34 augustus_masked-scaffold_7-processed-gene-0.617-mRNA-1 -            933     8e-12   44.2  12.7  10  11     0.002       1.6    8.9   0.0     8    32   842   866   836   868 0.85 Tetratricopeptide repeatTPR_2                PF07719.13    34 augustus_masked-scaffold_7-processed-gene-0.617-mRNA-1 -            933     8e-12   44.2  12.7  11  11       5.7   4.4e+03   -1.8   0.0    15    30   911   926   908   927 0.86 Tetratricopeptide repeatTPR_17               PF13431.2     34 augustus_masked-scaffold_7-processed-gene-0.617-mRNA-1 -            933   3.5e-10   39.4   8.3   1  11     0.037        29    5.2   0.0     4    33   291   320   288   321 0.86 Tetratricopeptide repeatTPR_17               PF13431.2     34 augustus_masked-scaffold_7-processed-gene-0.617-mRNA-1 -            933   3.5e-10   39.4   8.3   2  11       6.7   5.2e+03   -1.8   0.0     2    16   354   368   353   370 0.84 Tetratricopeptide repeatTPR_17               PF13431.2     34 augustus_masked-scaffold_7-processed-gene-0.617-mRNA-1 -            933   3.5e-10   39.4   8.3   3  11       2.3   1.7e+03   -0.4   0.0     6    21   405   420   405   424 0.88 Tetratricopeptide repeatTPR_17               PF13431.2     34 augustus_masked-scaffold_7-processed-gene-0.617-mRNA-1 -            933   3.5e-10   39.4   8.3   4  11      0.15   1.1e+02    3.3   0.0     3    23   417   437   415   452 0.81 Tetratricopeptide repeatTPR_17               PF13431.2     34 augustus_masked-scaffold_7-processed-gene-0.617-mRNA-1 -            933   3.5e-10   39.4   8.3   5  11     0.027        21    5.7   0.0     3    32   591   620   589   621 0.83 Tetratricopeptide repeatTPR_17               PF13431.2     34 augustus_masked-scaffold_7-processed-gene-0.617-mRNA-1 -            933   3.5e-10   39.4   8.3   6  11      0.15   1.2e+02    3.3   0.2     3    34   625   656   623   656 0.89 Tetratricopeptide repeatTPR_17               PF13431.2     34 augustus_masked-scaffold_7-processed-gene-0.617-mRNA-1 -            933   3.5e-10   39.4   8.3   7  11   0.00018      0.14   12.5   0.0     3    34   692   723   690   723 0.95 Tetratricopeptide repeatTPR_17               PF13431.2     34 augustus_masked-scaffold_7-processed-gene-0.617-mRNA-1 -            933   3.5e-10   39.4   8.3   8  11       1.7   1.3e+03    0.0   0.0    10    33   767   790   764   791 0.89 Tetratricopeptide repeatTPR_17               PF13431.2     34 augustus_masked-scaffold_7-processed-gene-0.617-mRNA-1 -            933   3.5e-10   39.4   8.3   9  11      0.46   3.5e+02    1.8   0.0     2    19   858   875   857   886 0.74 Tetratricopeptide repeatTPR_17               PF13431.2     34 augustus_masked-scaffold_7-processed-gene-0.617-mRNA-1 -            933   3.5e-10   39.4   8.3  10  11       5.8   4.5e+03   -1.7   0.0     4    18   894   908   893   911 0.86 Tetratricopeptide repeatTPR_17               PF13431.2     34 augustus_masked-scaffold_7-processed-gene-0.617-mRNA-1 -            933   3.5e-10   39.4   8.3  11  11       5.7   4.4e+03   -1.6   0.0     4    13   907   916   906   917 0.88 Tetratricopeptide repeatTPR_15               PF13429.2    280 augustus_masked-scaffold_7-processed-gene-0.617-mRNA-1 -            933   1.2e-07   31.1  25.3   1   5     0.021        16    4.4   0.0   121   193   277   347   270   369 0.82 Tetratricopeptide repeatTPR_15               PF13429.2    280 augustus_masked-scaffold_7-processed-gene-0.617-mRNA-1 -            933   1.2e-07   31.1  25.3   2   5      0.17   1.3e+02    1.4   0.6   138   181   449   491   413   499 0.74 Tetratricopeptide repeatTPR_15               PF13429.2    280 augustus_masked-scaffold_7-processed-gene-0.617-mRNA-1 -            933   1.2e-07   31.1  25.3   3   5   4.3e-06    0.0033   16.5   5.4   111   238   568   692   544   703 0.82 Tetratricopeptide repeatTPR_15               PF13429.2    280 augustus_masked-scaffold_7-processed-gene-0.617-mRNA-1 -            933   1.2e-07   31.1  25.3   4   5      0.01       7.9    5.4   0.0   144   182   700   738   691   746 0.86 Tetratricopeptide repeatTPR_15               PF13429.2    280 augustus_masked-scaffold_7-processed-gene-0.617-mRNA-1 -            933   1.2e-07   31.1  25.3   5   5   0.00018      0.14   11.2   1.6   138   198   760   822   739   878 0.77 Tetratricopeptide repeatTPR_6                PF13174.2     33 augustus_masked-scaffold_7-processed-gene-0.617-mRNA-1 -            933   3.5e-07   30.3   4.3   1   8      0.65     5e+02    1.6   0.0    14    33   280   299   273   299 0.78 Tetratricopeptide repeatTPR_6                PF13174.2     33 augustus_masked-scaffold_7-processed-gene-0.617-mRNA-1 -            933   3.5e-07   30.3   4.3   2   8       3.2   2.5e+03   -0.6   0.0     5    33   430   456   428   456 0.71 Tetratricopeptide repeatTPR_6                PF13174.2     33 augustus_masked-scaffold_7-processed-gene-0.617-mRNA-1 -            933   3.5e-07   30.3   4.3   3   8       7.6   5.9e+03   -1.8   0.0    14    24   547   557   541   565 0.77 Tetratricopeptide repeatTPR_6                PF13174.2     33 augustus_masked-scaffold_7-processed-gene-0.617-mRNA-1 -            933   3.5e-07   30.3   4.3   4   8     0.086        66    4.3   0.0     4    32   603   633   602   634 0.91 Tetratricopeptide repeatTPR_6                PF13174.2     33 augustus_masked-scaffold_7-processed-gene-0.617-mRNA-1 -            933   3.5e-07   30.3   4.3   5   8      0.16   1.2e+02    3.5   0.0    10    32   643   667   637   668 0.72 Tetratricopeptide repeatTPR_6                PF13174.2     33 augustus_masked-scaffold_7-processed-gene-0.617-mRNA-1 -            933   3.5e-07   30.3   4.3   6   8       1.3   9.8e+02    0.7   0.0    10    31   677   699   674   701 0.79 Tetratricopeptide repeatTPR_6                PF13174.2     33 augustus_masked-scaffold_7-processed-gene-0.617-mRNA-1 -            933   3.5e-07   30.3   4.3   7   8      0.17   1.3e+02    3.4   0.0     6    24   708   726   705   735 0.87 Tetratricopeptide repeatTPR_6                PF13174.2     33 augustus_masked-scaffold_7-processed-gene-0.617-mRNA-1 -            933   3.5e-07   30.3   4.3   8   8    0.0084       6.5    7.5   0.0    10    33   780   803   777   803 0.89 Tetratricopeptide repeatTPR_1                PF00515.24    34 augustus_masked-scaffold_7-processed-gene-0.617-mRNA-1 -            933   1.1e-06   28.0   0.4   1   4      0.89   6.9e+02    0.2   0.0     3    34   603   634   602   634 0.91 Tetratricopeptide repeatTPR_1                PF00515.24    34 augustus_masked-scaffold_7-processed-gene-0.617-mRNA-1 -            933   1.1e-06   28.0   0.4   2   4     0.077        60    3.6   0.0    14    32   681   699   679   700 0.93 Tetratricopeptide repeatTPR_1                PF00515.24    34 augustus_masked-scaffold_7-processed-gene-0.617-mRNA-1 -            933   1.1e-06   28.0   0.4   3   4   0.00022      0.17   11.6   0.1     1    24   702   725   702   731 0.93 Tetratricopeptide repeatTPR_1                PF00515.24    34 augustus_masked-scaffold_7-processed-gene-0.617-mRNA-1 -            933   1.1e-06   28.0   0.4   4   4     0.024        19    5.2   0.0    16    33   850   867   844   868 0.91 Tetratricopeptide repeatTPR_7                PF13176.2     36 augustus_masked-scaffold_7-processed-gene-0.617-mRNA-1 -            933   7.4e-05   22.4   0.4   1   5       4.8   3.7e+03   -1.7   0.0    12    26   546   560   544   567 0.79 Tetratricopeptide repeatTPR_7                PF13176.2     36 augustus_masked-scaffold_7-processed-gene-0.617-mRNA-1 -            933   7.4e-05   22.4   0.4   2   5       2.4   1.9e+03   -0.8   0.0     9    27   677   696   675   699 0.79 Tetratricopeptide repeatTPR_7                PF13176.2     36 augustus_masked-scaffold_7-processed-gene-0.617-mRNA-1 -            933   7.4e-05   22.4   0.4   3   5     0.028        21    5.3   0.0     6    24   709   727   704   748 0.79 Tetratricopeptide repeatTPR_7                PF13176.2     36 augustus_masked-scaffold_7-processed-gene-0.617-mRNA-1 -            933   7.4e-05   22.4   0.4   4   5      0.78     6e+02    0.7   0.0     7    28   778   800   772   805 0.82 Tetratricopeptide repeatTPR_7                PF13176.2     36 augustus_masked-scaffold_7-processed-gene-0.617-mRNA-1 -            933   7.4e-05   22.4   0.4   5   5    0.0029       2.2    8.4   0.0     3    34   839   870   836   871 0.90 Tetratricopeptide repeatANAPC3               PF12895.3     82 augustus_masked-scaffold_7-processed-gene-0.617-mRNA-1 -            933   0.00013   21.9   0.1   1   4     0.061        47    4.1   0.0    31    70   431   471   429   476 0.90 Anaphase-promoting complex, cyclosome, subunit 3ANAPC3               PF12895.3     82 augustus_masked-scaffold_7-processed-gene-0.617-mRNA-1 -            933   0.00013   21.9   0.1   2   4     0.049        38    4.4   0.0    12    62   590   641   584   661 0.80 Anaphase-promoting complex, cyclosome, subunit 3ANAPC3               PF12895.3     82 augustus_masked-scaffold_7-processed-gene-0.617-mRNA-1 -            933   0.00013   21.9   0.1   3   4    0.0056       4.3    7.4   0.0    20    80   663   726   647   728 0.79 Anaphase-promoting complex, cyclosome, subunit 3ANAPC3               PF12895.3     82 augustus_masked-scaffold_7-processed-gene-0.617-mRNA-1 -            933   0.00013   21.9   0.1   4   4         2   1.6e+03   -0.8   0.0     4    39   850   885   848   902 0.78 Anaphase-promoting complex, cyclosome, subunit 3TPR_21               PF09976.5    145 augustus_masked-scaffold_7-processed-gene-0.617-mRNA-1 -            933   0.00016   21.6  16.4   1   4       2.3   1.8e+03   -1.2   0.4    37    68   215   245   176   247 0.72 Tetratricopeptide repeatTPR_21               PF09976.5    145 augustus_masked-scaffold_7-processed-gene-0.617-mRNA-1 -            933   0.00016   21.6  16.4   2   4   0.00039      0.31   11.0   0.0    49    94   267   312   259   339 0.80 Tetratricopeptide repeatTPR_21               PF09976.5    145 augustus_masked-scaffold_7-processed-gene-0.617-mRNA-1 -            933   0.00016   21.6  16.4   3   4   0.00032      0.24   11.4   1.2    51   114   604   664   568   670 0.84 Tetratricopeptide repeatTPR_21               PF09976.5    145 augustus_masked-scaffold_7-processed-gene-0.617-mRNA-1 -            933   0.00016   21.6  16.4   4   4    0.0069       5.3    7.0   0.1   117   142   701   726   680   729 0.77 Tetratricopeptide repeatTPR_8                PF13181.2     34 augustus_masked-scaffold_7-processed-gene-0.617-mRNA-1 -            933   0.00019   21.2   8.6   1   4       6.9   5.3e+03   -2.1   0.1    14    24   546   556   544   559 0.84 Tetratricopeptide repeatTPR_8                PF13181.2     34 augustus_masked-scaffold_7-processed-gene-0.617-mRNA-1 -            933   0.00019   21.2   8.6   2   4      0.82   6.4e+02    0.8   0.0     3    34   603   634   601   634 0.92 Tetratricopeptide repeatTPR_8                PF13181.2     34 augustus_masked-scaffold_7-processed-gene-0.617-mRNA-1 -            933   0.00019   21.2   8.6   3   4   1.1e-05    0.0083   16.0   0.0     1    25   702   726   702   733 0.90 Tetratricopeptide repeatTPR_8                PF13181.2     34 augustus_masked-scaffold_7-processed-gene-0.617-mRNA-1 -            933   0.00019   21.2   8.6   4   4       0.1        79    3.6   0.0     9    31   843   865   837   868 0.78 Tetratricopeptide repeatTPR_12               PF13424.2     79 augustus_masked-scaffold_7-processed-gene-0.617-mRNA-1 -            933   0.00025   21.2  13.5   1   6         4   3.1e+03   -1.6   0.0    23    51   387   416   381   422 0.61 Tetratricopeptide repeatTPR_12               PF13424.2     79 augustus_masked-scaffold_7-processed-gene-0.617-mRNA-1 -            933   0.00025   21.2  13.5   2   6       1.5   1.2e+03   -0.2   0.0    51    76   605   630   569   633 0.71 Tetratricopeptide repeatTPR_12               PF13424.2     79 augustus_masked-scaffold_7-processed-gene-0.617-mRNA-1 -            933   0.00025   21.2  13.5   3   6     0.019        15    5.9   0.1     5    77   635   698   631   699 0.65 Tetratricopeptide repeatTPR_12               PF13424.2     79 augustus_masked-scaffold_7-processed-gene-0.617-mRNA-1 -            933   0.00025   21.2  13.5   4   6   0.00011     0.085   13.1   0.4    16    70   679   725   673   731 0.92 Tetratricopeptide repeatTPR_12               PF13424.2     79 augustus_masked-scaffold_7-processed-gene-0.617-mRNA-1 -            933   0.00025   21.2  13.5   5   6      0.32   2.4e+02    2.0   0.1    46    75   769   798   735   801 0.62 Tetratricopeptide repeatTPR_12               PF13424.2     79 augustus_masked-scaffold_7-processed-gene-0.617-mRNA-1 -            933   0.00025   21.2  13.5   6   6    0.0028       2.2    8.5   0.0    11    37   841   867   830   887 0.74 Tetratricopeptide repeatWzy_C_2              PF11846.4    188 augustus_masked-scaffold_7-processed-gene-0.617-mRNA-1 -            933   0.00036   20.4   1.5   1   3     0.018        14    5.4   0.0   149   184   429   464   379   468 0.91 Virulence factor membrane-bound polymerase, C-terminalWzy_C_2              PF11846.4    188 augustus_masked-scaffold_7-processed-gene-0.617-mRNA-1 -            933   0.00036   20.4   1.5   2   3    0.0016       1.2    8.9   0.1   129   180   654   705   636   710 0.82 Virulence factor membrane-bound polymerase, C-terminalWzy_C_2              PF11846.4    188 augustus_masked-scaffold_7-processed-gene-0.617-mRNA-1 -            933   0.00036   20.4   1.5   3   3      0.26     2e+02    1.6   0.0   155   184   748   777   735   781 0.85 Virulence factor membrane-bound polymerase, C-terminalNARP1                PF12569.4    519 augustus_masked-scaffold_7-processed-gene-0.617-mRNA-1 -            933   0.00044   19.2   6.8   1   6      0.45   3.4e+02   -0.2   0.8   420   447   114   141    80   174 0.58 NMDA receptor-regulated protein 1NARP1                PF12569.4    519 augustus_masked-scaffold_7-processed-gene-0.617-mRNA-1 -            933   0.00044   19.2   6.8   2   6      0.18   1.4e+02    1.1   0.0   208   254   435   480   418   487 0.84 NMDA receptor-regulated protein 1NARP1                PF12569.4    519 augustus_masked-scaffold_7-processed-gene-0.617-mRNA-1 -            933   0.00044   19.2   6.8   3   6     0.019        15    4.3   0.0   207   256   578   627   563   631 0.85 NMDA receptor-regulated protein 1NARP1                PF12569.4    519 augustus_masked-scaffold_7-processed-gene-0.617-mRNA-1 -            933   0.00044   19.2   6.8   4   6    0.0074       5.7    5.7   0.0   206   252   678   724   630   730 0.78 NMDA receptor-regulated protein 1NARP1                PF12569.4    519 augustus_masked-scaffold_7-processed-gene-0.617-mRNA-1 -            933   0.00044   19.2   6.8   5   6    0.0012      0.95    8.2   0.0   203   288   709   794   704   800 0.94 NMDA receptor-regulated protein 1NARP1                PF12569.4    519 augustus_masked-scaffold_7-processed-gene-0.617-mRNA-1 -            933   0.00044   19.2   6.8   6   6      0.45   3.5e+02   -0.2   0.0   221   261   826   866   797   872 0.82 NMDA receptor-regulated protein 1TPR_9                PF13371.2     73 augustus_masked-scaffold_7-processed-gene-0.617-mRNA-1 -            933    0.0042   17.0  10.2   1   6      0.88   6.8e+02    0.3   0.0    17    65   288   336   277   344 0.84 Tetratricopeptide repeatTPR_9                PF13371.2     73 augustus_masked-scaffold_7-processed-gene-0.617-mRNA-1 -            933    0.0042   17.0  10.2   2   6       1.5   1.1e+03   -0.4   0.0     5    44   433   472   430   475 0.84 Tetratricopeptide repeatTPR_9                PF13371.2     73 augustus_masked-scaffold_7-processed-gene-0.617-mRNA-1 -            933    0.0042   17.0  10.2   3   6       4.8   3.7e+03   -2.1   0.0    50    52   574   576   544   612 0.56 Tetratricopeptide repeatTPR_9                PF13371.2     73 augustus_masked-scaffold_7-processed-gene-0.617-mRNA-1 -            933    0.0042   17.0  10.2   4   6      0.56   4.3e+02    0.9   0.1    13    72   585   644   576   645 0.80 Tetratricopeptide repeatTPR_9                PF13371.2     73 augustus_masked-scaffold_7-processed-gene-0.617-mRNA-1 -            933    0.0042   17.0  10.2   5   6   0.00085      0.66   10.0   0.0    11    52   684   725   681   733 0.91 Tetratricopeptide repeatTPR_9                PF13371.2     73 augustus_masked-scaffold_7-processed-gene-0.617-mRNA-1 -            933    0.0042   17.0  10.2   6   6     0.017        13    5.8   0.8     7    60   748   801   743   811 0.90 Tetratricopeptide repeatSuf                  PF05843.10   296 augustus_masked-scaffold_7-processed-gene-0.617-mRNA-1 -            933     0.022   14.6   0.5   1   4       2.4   1.9e+03   -1.6   0.2   171   208   122   159   100   202 0.54 Suppressor of forked protein (Suf)Suf                  PF05843.10   296 augustus_masked-scaffold_7-processed-gene-0.617-mRNA-1 -            933     0.022   14.6   0.5   2   4     0.014        11    5.8   0.0    10    44   432   466   427   475 0.90 Suppressor of forked protein (Suf)Suf                  PF05843.10   296 augustus_masked-scaffold_7-processed-gene-0.617-mRNA-1 -            933     0.022   14.6   0.5   3   4     0.076        59    3.3   0.0    16    67   616   666   603   676 0.81 Suppressor of forked protein (Suf)Suf                  PF05843.10   296 augustus_masked-scaffold_7-processed-gene-0.617-mRNA-1 -            933     0.022   14.6   0.5   4   4       5.2     4e+03   -2.7   0.0     4    28   773   797   770   805 0.86 Suppressor of forked protein (Suf)DUF3808              PF10300.5    477 augustus_masked-scaffold_7-processed-gene-0.617-mRNA-1 -            933     0.051   12.2   0.4   1   4    0.0019       1.5    7.3   0.0   246   296   276   327   257   336 0.84 Protein of unknown function (DUF3808)DUF3808              PF10300.5    477 augustus_masked-scaffold_7-processed-gene-0.617-mRNA-1 -            933     0.051   12.2   0.4   2   4       2.9   2.2e+03   -3.1   0.0   252   283   440   471   435   488 0.81 Protein of unknown function (DUF3808)DUF3808              PF10300.5    477 augustus_masked-scaffold_7-processed-gene-0.617-mRNA-1 -            933     0.051   12.2   0.4   3   4      0.36   2.8e+02   -0.1   0.0   246   293   611   659   553   684 0.53 Protein of unknown function (DUF3808)DUF3808              PF10300.5    477 augustus_masked-scaffold_7-processed-gene-0.617-mRNA-1 -            933     0.051   12.2   0.4   4   4      0.38   2.9e+02   -0.2   0.0   248   292   681   725   652   731 0.85 Protein of unknown function (DUF3808)TPR_20               PF14561.2     90 augustus_masked-scaffold_7-processed-gene-0.617-mRNA-1 -            933       1.2    9.4   6.8   1   2     0.056        43    4.4   0.0    10    41   589   620   582   624 0.91 Tetratricopeptide repeatTPR_20               PF14561.2     90 augustus_masked-scaffold_7-processed-gene-0.617-mRNA-1 -            933       1.2    9.4   6.8   2   2     0.024        19    5.5   0.8    11    53   624   665   622   688 0.83 Tetratricopeptide repeatadh_short            PF00106.21   195 augustus_masked-scaffold_7-processed-gene-0.627-mRNA-1 -            360   5.5e-50  169.4   1.0   1   1   3.2e-53   7.5e-50  169.0   1.0     1   188   112   305   112   310 0.93 short chain dehydrogenaseadh_short_C2         PF13561.2    241 augustus_masked-scaffold_7-processed-gene-0.627-mRNA-1 -            360     3e-27   96.0   0.1   1   1   1.6e-30   3.8e-27   95.7   0.1     6   241   121   358   118   358 0.91 Enoyl-(Acyl carrier protein) reductaseKR                   PF08659.6    180 augustus_masked-scaffold_7-processed-gene-0.627-mRNA-1 -            360   1.5e-12   47.7   0.4   1   1   8.4e-16     2e-12   47.3   0.4     2   173   113   294   112   301 0.81 KR domainEpimerase            PF01370.17   241 augustus_masked-scaffold_7-processed-gene-0.627-mRNA-1 -            360   1.7e-05   24.3   0.2   1   1   1.6e-08   3.8e-05   23.1   0.2     1   161   114   285   114   336 0.77 NAD dependent epimerase/dehydratase familyPolysacc_synt_2      PF02719.11   294 augustus_masked-scaffold_7-processed-gene-0.627-mRNA-1 -            360     0.016   14.2   0.0   1   1   9.1e-06     0.021   13.8   0.0     1    74   114   182   114   231 0.79 Polysaccharide biosynthesis proteinPA                   PF02225.18    91 augustus_masked-scaffold_7-processed-gene-0.627-mRNA-1 -            360     0.088   12.7   0.2   1   2   0.00012      0.29   11.1   0.0    24    86   108   174    81   179 0.74 PA domainPA                   PF02225.18    91 augustus_masked-scaffold_7-processed-gene-0.627-mRNA-1 -            360     0.088   12.7   0.2   2   2       1.4   3.4e+03   -2.0   0.0    29    43   246   272   231   281 0.59 PA domainDnaB_2               PF07261.7     74 augustus_masked-scaffold_7-processed-gene-0.627-mRNA-1 -            360      0.15   11.6   0.1   1   1   0.00016      0.37   10.4   0.1    40    65   263   290   248   290 0.71 Replication initiation and membrane attachmentIsy1                 PF06246.8    248 augustus_masked-scaffold_7-processed-gene-0.670-mRNA-1 -            243   3.3e-87  292.1   2.4   1   1   2.2e-91   3.7e-87  291.9   2.4     1   248     1   242     1   242 0.94 Isy1-like splicing familyRibosomal_L7Ae       PF01248.22    95 maker-scaffold_7-augustus-gene-0.1397-mRNA-1 -            286     8e-20   70.2   0.4   1   2   3.2e-23   1.8e-19   69.2   0.0     3    79   122   198   120   206 0.94 Ribosomal protein L7Ae/L30e/S12e/Gadd45 familyRibosomal_L7Ae       PF01248.22    95 maker-scaffold_7-augustus-gene-0.1397-mRNA-1 -            286     8e-20   70.2   0.4   2   2       1.1   5.9e+03   -3.1   0.1    17    32   264   279   257   280 0.83 Ribosomal protein L7Ae/L30e/S12e/Gadd45 familyRNase_P_pop3         PF08228.7    158 maker-scaffold_7-augustus-gene-0.1397-mRNA-1 -            286     0.026   14.4   0.0   1   1   9.1e-06     0.049   13.5   0.0    32   100   111   177    98   194 0.74 RNase P subunit Pop3CLPTM1               PF05602.8    427 maker-scaffold_7-augustus-gene-0.1397-mRNA-1 -            286     0.042   12.8   0.5   1   1     1e-05     0.055   12.4   0.5   119   172    84   137    44   159 0.77 Cleft lip and palate transmembrane protein 1 (CLPTM1)5_nucleotid_C        PF02872.14   158 maker-scaffold_7-augustus-gene-0.1439-mRNA-1 -            603   9.3e-05   22.6   0.0   1   2      0.27   4.4e+03   -2.4   0.0   115   147   336   367   322   373 0.81 5'-nucleotidase, C-terminal domain5_nucleotid_C        PF02872.14   158 maker-scaffold_7-augustus-gene-0.1439-mRNA-1 -            603   9.3e-05   22.6   0.0   2   2     2e-08   0.00032   20.9   0.0    20    67   375   430   364   441 0.92 5'-nucleotidase, C-terminal domainPam16                PF03656.9    127 augustus_masked-scaffold_7-processed-gene-0.613-mRNA-1 -            140   6.5e-33  113.3   0.1   1   1   1.4e-36   7.7e-33  113.1   0.1     1   121     1   126     1   131 0.91 Pam16GMC_oxred_N          PF00732.15   296 augustus_masked-scaffold_7-processed-gene-0.613-mRNA-1 -            140     0.088   12.0   0.1   1   1   1.8e-05     0.096   11.9   0.1    48    90    13    55     3   114 0.87 GMC oxidoreductaseT2SSK                PF03934.9    280 augustus_masked-scaffold_7-processed-gene-0.613-mRNA-1 -            140     0.098   11.9   0.0   1   1   2.8e-05      0.15   11.2   0.0   196   257    49   111    49   126 0.82 Type II secretion system (T2SS), protein KSET                  PF00856.24   141 snap_masked-scaffold_7-processed-gene-0.1140-mRNA-1 -            203   0.00025   21.3   0.0   1   1   1.1e-06     0.018   15.2   0.0     2   141    80   194    79   194 0.61 SET domainBeach                PF02138.14   272 augustus_masked-scaffold_7-processed-gene-0.672-mRNA-1 -           2611  3.4e-115  384.0   0.0   1   1  1.9e-118  5.2e-115  383.4   0.0     1   272  1994  2273  1994  2273 0.97 Beige/BEACH domainPH_BEACH             PF14844.2    100 augustus_masked-scaffold_7-processed-gene-0.672-mRNA-1 -           2611   2.3e-10   40.3   0.0   1   1   1.8e-13   4.9e-10   39.3   0.0    33    99  1875  1941  1821  1942 0.75 PH domain associated with Beige/BEACHLaminin_G_3          PF13385.2    152 augustus_masked-scaffold_7-processed-gene-0.672-mRNA-1 -           2611   7.9e-10   38.9   0.0   1   1   6.4e-13   1.7e-09   37.8   0.0    14   110   381   487   365   520 0.70 Concanavalin A-like lectin/glucanases superfamilyDUF4800              PF16057.1    254 augustus_masked-scaffold_7-processed-gene-0.672-mRNA-1 -           2611    0.0005   19.3   0.3   1   1   3.8e-07     0.001   18.3   0.3   171   240  1640  1707  1610  1716 0.86 Domain of unknown function (DUF4800)DUF4704              PF15787.1    279 augustus_masked-scaffold_7-processed-gene-0.672-mRNA-1 -           2611      0.11   11.6   0.0   1   1   0.00013      0.35    9.9   0.0    62   184   727   872   711   874 0.77 Domain of unknown function (DUF4704)WD40                 PF00400.28    37 augustus_masked-scaffold_7-processed-gene-0.672-mRNA-1 -           2611      0.33   11.7   3.6   1   4      0.21   5.8e+02    1.5   0.0    10    35  2381  2407  2376  2408 0.79 WD domain, G-beta repeatWD40                 PF00400.28    37 augustus_masked-scaffold_7-processed-gene-0.672-mRNA-1 -           2611      0.33   11.7   3.6   2   4     0.024        66    4.5   0.0     7    37  2426  2458  2421  2458 0.71 WD domain, G-beta repeatWD40                 PF00400.28    37 augustus_masked-scaffold_7-processed-gene-0.672-mRNA-1 -           2611      0.33   11.7   3.6   3   4      0.13   3.6e+02    2.1   0.1    14    36  2517  2548  2503  2549 0.65 WD domain, G-beta repeatWD40                 PF00400.28    37 augustus_masked-scaffold_7-processed-gene-0.672-mRNA-1 -           2611      0.33   11.7   3.6   4   4         1   2.8e+03   -0.7   0.0    13    34  2581  2602  2563  2605 0.76 WD domain, G-beta repeatOCD_Mu_crystall      PF02423.11   318 maker-scaffold_7-augustus-gene-0.1430-mRNA-1 -            472   2.6e-08   33.0   0.0   1   3     3e-08   0.00016   20.6   0.0    39   175    52   191    18   200 0.72 Ornithine cyclodeaminase/mu-crystallin familyOCD_Mu_crystall      PF02423.11   318 maker-scaffold_7-augustus-gene-0.1430-mRNA-1 -            472   2.6e-08   33.0   0.0   2   3   6.4e-05      0.35    9.6   0.0   189   241   224   282   221   319 0.84 Ornithine cyclodeaminase/mu-crystallin familyOCD_Mu_crystall      PF02423.11   318 maker-scaffold_7-augustus-gene-0.1430-mRNA-1 -            472   2.6e-08   33.0   0.0   3   3      0.34   1.9e+03   -2.6   0.0   291   306   437   452   433   462 0.84 Ornithine cyclodeaminase/mu-crystallin familyShikimate_DH         PF01488.16   138 maker-scaffold_7-augustus-gene-0.1430-mRNA-1 -            472      0.01   15.7   0.0   1   3   0.00034       1.8    8.5   0.0    12    56   143   190   136   200 0.78 Shikimate / quinate 5-dehydrogenaseShikimate_DH         PF01488.16   138 maker-scaffold_7-augustus-gene-0.1430-mRNA-1 -            472      0.01   15.7   0.0   2   3    0.0063        34    4.3   0.0    69   106   222   258   208   269 0.81 Shikimate / quinate 5-dehydrogenaseShikimate_DH         PF01488.16   138 maker-scaffold_7-augustus-gene-0.1430-mRNA-1 -            472      0.01   15.7   0.0   3   3       2.3   1.2e+04   -3.9   0.0    66    94   305   333   297   341 0.67 Shikimate / quinate 5-dehydrogenaseHerpes_DNAp_acc      PF04929.8    400 maker-scaffold_7-augustus-gene-0.1430-mRNA-1 -            472       5.2    5.8   6.6   1   1    0.0014       7.8    5.2   6.6   294   384   338   429   313   435 0.70 Herpes DNA replication accessory factorPhosducin            PF02114.12   265 maker-scaffold_7-augustus-gene-0.1434-mRNA-1 -            261   1.2e-14   53.8   0.1   1   1   5.1e-18   1.7e-14   53.3   0.1    93   255    70   229    50   237 0.86 PhosducinDUF3350              PF11830.4     64 maker-scaffold_7-augustus-gene-0.1434-mRNA-1 -            261    0.0054   16.9   0.1   1   2   0.00011      0.36   11.0   0.1    22    50    73   101    59   103 0.81 Domain of unknown function (DUF3350)DUF3350              PF11830.4     64 maker-scaffold_7-augustus-gene-0.1434-mRNA-1 -            261    0.0054   16.9   0.1   2   2     0.046   1.5e+02    2.6   0.0    37    48   138   149   125   150 0.81 Domain of unknown function (DUF3350)Thioredoxin          PF00085.16   104 maker-scaffold_7-augustus-gene-0.1434-mRNA-1 -            261     0.012   15.4   0.0   1   1   6.6e-06     0.021   14.6   0.0     5    91   105   191   101   204 0.81 ThioredoxinDUF3503              PF12011.4    174 maker-scaffold_7-augustus-gene-0.1434-mRNA-1 -            261     0.031   14.0   0.0   1   1   1.5e-05     0.048   13.4   0.0    16    57    76   117    70   167 0.74 Domain of unknown function (DUF3503)TMA7                 PF09072.6     62 maker-scaffold_7-augustus-gene-0.1434-mRNA-1 -            261      0.64   10.7   8.0   1   2   0.00037       1.2    9.9   2.5    21    47    72    99    60   103 0.72 Translation machinery associated TMA7TMA7                 PF09072.6     62 maker-scaffold_7-augustus-gene-0.1434-mRNA-1 -            261      0.64   10.7   8.0   2   2     0.041   1.3e+02    3.3   0.3    22    51   216   248   210   258 0.73 Translation machinery associated TMA7DUF1445              PF07286.8    143 augustus_masked-scaffold_7-processed-gene-0.578-mRNA-1 -            292     2e-59  199.5   0.0   1   1   1.6e-63   2.6e-59  199.1   0.0     1   143   125   282   125   282 0.99 Protein of unknown function (DUF1445)adh_short            PF00106.21   195 maker-scaffold_7-augustus-gene-0.1438-mRNA-1 -            250   5.3e-46  156.4   2.6   1   1   3.7e-49   6.7e-46  156.1   2.6     1   193     4   190     4   192 0.97 short chain dehydrogenaseadh_short_C2         PF13561.2    241 maker-scaffold_7-augustus-gene-0.1438-mRNA-1 -            250   6.8e-18   65.4   1.9   1   1   4.5e-21   8.2e-18   65.1   1.9     5   192    12   190    10   232 0.89 Enoyl-(Acyl carrier protein) reductaseKR                   PF08659.6    180 maker-scaffold_7-augustus-gene-0.1438-mRNA-1 -            250   1.7e-11   44.2   1.3   1   1   1.3e-14   2.4e-11   43.7   1.3     2   177     5   176     4   178 0.92 KR domainEpimerase            PF01370.17   241 maker-scaffold_7-augustus-gene-0.1438-mRNA-1 -            250   0.00088   18.7   0.3   1   2   1.4e-06    0.0025   17.2   0.1     2    61     7    69     6    79 0.85 NAD dependent epimerase/dehydratase familyEpimerase            PF01370.17   241 maker-scaffold_7-augustus-gene-0.1438-mRNA-1 -            250   0.00088   18.7   0.3   2   2      0.86   1.5e+03   -1.8   0.0   140   162   146   169   134   179 0.73 NAD dependent epimerase/dehydratase familyPP2C_2               PF13672.2    210 maker-scaffold_7-augustus-gene-0.1438-mRNA-1 -            250    0.0043   16.6   0.2   1   1     4e-06    0.0072   15.8   0.2    20    99   124   210   118   225 0.73 Protein phosphatase 2CGDP_Man_Dehyd        PF16363.1    332 maker-scaffold_7-augustus-gene-0.1438-mRNA-1 -            250     0.006   15.9   0.0   1   1   4.7e-06    0.0084   15.4   0.0     2    73     8    70     7   136 0.86 GDP-mannose 4,6 dehydrataseNmrA                 PF05368.9    233 maker-scaffold_7-augustus-gene-0.1438-mRNA-1 -            250     0.034   13.5   0.3   1   1   3.7e-05     0.066   12.6   0.3     2    69     7    75     6    94 0.86 NmrA-like familyHHH_5                PF14520.2     57 maker-scaffold_7-augustus-gene-0.1438-mRNA-1 -            250     0.045   14.2   1.2   1   3     0.024        44    4.6   0.1     8    22    13    27     9    45 0.87 Helix-hairpin-helix domainHHH_5                PF14520.2     57 maker-scaffold_7-augustus-gene-0.1438-mRNA-1 -            250     0.045   14.2   1.2   2   3     0.046        84    3.7   0.0     9    30    62    82    61   108 0.53 Helix-hairpin-helix domainHHH_5                PF14520.2     57 maker-scaffold_7-augustus-gene-0.1438-mRNA-1 -            250     0.045   14.2   1.2   3   3       0.3   5.4e+02    1.1   0.0    18    42   104   128   101   130 0.83 Helix-hairpin-helix domainTrkA_N               PF02254.14   116 maker-scaffold_7-augustus-gene-0.1438-mRNA-1 -            250     0.047   13.8   0.1   1   2   7.3e-05      0.13   12.4   0.1     2    58     7    67     6    76 0.81 TrkA-N domainTrkA_N               PF02254.14   116 maker-scaffold_7-augustus-gene-0.1438-mRNA-1 -            250     0.047   13.8   0.1   2   2       3.3     6e+03   -2.7   0.0    56    66    94   104    81   133 0.58 TrkA-N domainLIM                  PF00412.18    58 snap_masked-scaffold_7-processed-gene-0.1069-mRNA-1 -            829   4.9e-23   81.0  25.5   1   3   2.8e-10   1.5e-06   28.2   1.0     1    40   608   647   608   654 0.94 LIM domainLIM                  PF00412.18    58 snap_masked-scaffold_7-processed-gene-0.1069-mRNA-1 -            829   4.9e-23   81.0  25.5   2   3   1.5e-11   7.9e-08   32.3   6.2     1    56   704   758   704   760 0.95 LIM domainLIM                  PF00412.18    58 snap_masked-scaffold_7-processed-gene-0.1069-mRNA-1 -            829   4.9e-23   81.0  25.5   3   3   3.2e-11   1.8e-07   31.2   3.4     1    53   767   822   767   826 0.79 LIM domainDUF2321              PF10083.5    156 snap_masked-scaffold_7-processed-gene-0.1069-mRNA-1 -            829    0.0086   15.6   0.3   1   3      0.19     1e+03   -0.9   0.5    36    50   604   618   593   643 0.69 Uncharacterized protein conserved in bacteria (DUF2321)DUF2321              PF10083.5    156 snap_masked-scaffold_7-processed-gene-0.1069-mRNA-1 -            829    0.0086   15.6   0.3   2   3   1.6e-06    0.0086   15.6   0.3    22    81   685   742   677   748 0.72 Uncharacterized protein conserved in bacteria (DUF2321)DUF2321              PF10083.5    156 snap_masked-scaffold_7-processed-gene-0.1069-mRNA-1 -            829    0.0086   15.6   0.3   3   3      0.23   1.2e+03   -1.2   1.6    28    61   751   784   739   800 0.57 Uncharacterized protein conserved in bacteria (DUF2321)zf-dskA_traR         PF01258.13    36 snap_masked-scaffold_7-processed-gene-0.1069-mRNA-1 -            829     0.078   12.8   0.3   1   6      0.14   7.5e+02    0.1   0.1     6    13   608   615   600   616 0.78 Prokaryotic dksA/traR C4-type zinc fingerzf-dskA_traR         PF01258.13    36 snap_masked-scaffold_7-processed-gene-0.1069-mRNA-1 -            829     0.078   12.8   0.3   2   6         3   1.6e+04   -4.4   2.2     6    10   636   640   628   644 0.59 Prokaryotic dksA/traR C4-type zinc fingerzf-dskA_traR         PF01258.13    36 snap_masked-scaffold_7-processed-gene-0.1069-mRNA-1 -            829     0.078   12.8   0.3   3   6      0.41   2.2e+03   -1.5   0.1    24    32   701   709   699   711 0.80 Prokaryotic dksA/traR C4-type zinc fingerzf-dskA_traR         PF01258.13    36 snap_masked-scaffold_7-processed-gene-0.1069-mRNA-1 -            829     0.078   12.8   0.3   4   6   1.4e-05     0.078   12.8   0.3     3    34   725   759   724   761 0.90 Prokaryotic dksA/traR C4-type zinc fingerzf-dskA_traR         PF01258.13    36 snap_masked-scaffold_7-processed-gene-0.1069-mRNA-1 -            829     0.078   12.8   0.3   5   6      0.23   1.3e+03   -0.7   2.7     6    32   767   798   762   801 0.61 Prokaryotic dksA/traR C4-type zinc fingerzf-dskA_traR         PF01258.13    36 snap_masked-scaffold_7-processed-gene-0.1069-mRNA-1 -            829     0.078   12.8   0.3   6   6      0.64   3.4e+03   -2.1   0.2    25    33   817   825   816   826 0.81 Prokaryotic dksA/traR C4-type zinc fingerMatE                 PF01554.14   161 snap_masked-scaffold_7-processed-gene-0.1242-mRNA-1 -            498   1.2e-07   31.4  20.2   1   3   2.4e-08   0.00013   21.5   4.1    72   160    96   183    24   184 0.82 MatEMatE                 PF01554.14   161 snap_masked-scaffold_7-processed-gene-0.1242-mRNA-1 -            498   1.2e-07   31.4  20.2   2   3    0.0029        16    5.0   0.7     5    86   269   350   266   356 0.84 MatEMatE                 PF01554.14   161 snap_masked-scaffold_7-processed-gene-0.1242-mRNA-1 -            498   1.2e-07   31.4  20.2   3   3   2.9e-06     0.015   14.8   1.9    72   152   354   434   349   444 0.83 MatEPolysacc_synt_C      PF14667.2    142 snap_masked-scaffold_7-processed-gene-0.1242-mRNA-1 -            498    0.0014   18.5   0.2   1   4      0.41   2.2e+03   -1.5   0.2    88   103    98   113    84   125 0.52 Polysaccharide biosynthesis C-terminal domainPolysacc_synt_C      PF14667.2    142 snap_masked-scaffold_7-processed-gene-0.1242-mRNA-1 -            498    0.0014   18.5   0.2   2   4   2.7e-07    0.0014   18.5   0.2     2    69   138   215   137   234 0.77 Polysaccharide biosynthesis C-terminal domainPolysacc_synt_C      PF14667.2    142 snap_masked-scaffold_7-processed-gene-0.1242-mRNA-1 -            498    0.0014   18.5   0.2   3   4       1.4   7.6e+03   -3.3   4.3    92   104   340   354   264   385 0.62 Polysaccharide biosynthesis C-terminal domainPolysacc_synt_C      PF14667.2    142 snap_masked-scaffold_7-processed-gene-0.1242-mRNA-1 -            498    0.0014   18.5   0.2   4   4      0.55     3e+03   -1.9   1.6    92   110   465   483   404   494 0.54 Polysaccharide biosynthesis C-terminal domainUL40                 PF10682.5    214 snap_masked-scaffold_7-processed-gene-0.1242-mRNA-1 -            498     0.033   13.7   0.1   1   1   1.1e-05      0.06   12.9   0.1    18    72   132   187   124   218 0.83 Glycoprotein of human cytomegalovirus HHV-5S-methyl_trans       PF02574.12   264 snap_masked-scaffold_7-processed-gene-0.1236-mRNA-1 -            341   9.4e-54  182.9   0.0   1   1   6.6e-58   1.1e-53  182.7   0.0     1   262     5   334     5   336 0.83 Homocysteine S-methyltransferaseGlyco_hydro_81       PF03639.9    682 maker-scaffold_7-augustus-gene-0.1433-mRNA-1 -            880  2.2e-249  829.6  21.4   1   2  6.7e-101   1.1e-96  325.0   0.2     3   369   166   531   164   546 0.95 Glycosyl hydrolase family 81Glyco_hydro_81       PF03639.9    682 maker-scaffold_7-augustus-gene-0.1433-mRNA-1 -            880  2.2e-249  829.6  21.4   2   2  2.6e-156  4.2e-152  508.1  15.9   369   682   554   869   551   869 0.99 Glycosyl hydrolase family 81ENTH                 PF01417.16   125 snap_masked-scaffold_7-processed-gene-0.1142-mRNA-1 -            567   4.6e-46  155.8   0.7   1   2   1.7e-14   6.7e-11   42.2   0.0     1    34    15    48    15    54 0.91 ENTH domainENTH                 PF01417.16   125 snap_masked-scaffold_7-processed-gene-0.1142-mRNA-1 -            567   4.6e-46  155.8   0.7   2   2   4.3e-36   1.8e-32  111.9   0.1    32   125    68   160    59   160 0.96 ENTH domainDUF1720              PF08226.7     75 snap_masked-scaffold_7-processed-gene-0.1142-mRNA-1 -            567   1.3e-08   34.9  38.3   1   3   3.3e-12   1.3e-08   34.9  38.3     2    75   272   356   271   356 0.97 Domain of unknown function (DUF1720)DUF1720              PF08226.7     75 snap_masked-scaffold_7-processed-gene-0.1142-mRNA-1 -            567   1.3e-08   34.9  38.3   2   3         4   1.6e+04   -4.9  40.0     1    64   349   451   339   462 0.64 Domain of unknown function (DUF1720)DUF1720              PF08226.7     75 snap_masked-scaffold_7-processed-gene-0.1142-mRNA-1 -            567   1.3e-08   34.9  38.3   3   3         4   1.6e+04   -9.8  24.1    28    64   503   557   445   566 0.69 Domain of unknown function (DUF1720)UIM                  PF02809.16    17 snap_masked-scaffold_7-processed-gene-0.1142-mRNA-1 -            567     0.014   15.1   4.2   1   2     0.031   1.3e+02    2.7   0.6     1    13   201   213   201   214 0.92 Ubiquitin interaction motifUIM                  PF02809.16    17 snap_masked-scaffold_7-processed-gene-0.1142-mRNA-1 -            567     0.014   15.1   4.2   2   2     2e-05      0.08   12.8   0.2     3    16   234   247   234   247 0.97 Ubiquitin interaction motifDUF1510              PF07423.7    216 snap_masked-scaffold_7-processed-gene-0.1142-mRNA-1 -            567       4.2    6.6   7.6   1   2   0.00035       1.4    8.1   0.1   115   146    16    47     5    50 0.83 Protein of unknown function (DUF1510)DUF1510              PF07423.7    216 snap_masked-scaffold_7-processed-gene-0.1142-mRNA-1 -            567       4.2    6.6   7.6   2   2      0.14   5.6e+02   -0.3   3.8    59   136   211   272   162   292 0.42 Protein of unknown function (DUF1510)Nup96                PF12110.4    290 maker-scaffold_7-augustus-gene-0.1398-mRNA-1 -           2003   8.1e-99  330.3   1.6   1   2  2.2e-102   1.2e-98  329.7   0.9     1   289  1535  1835  1535  1836 0.98 Nuclear protein 96Nup96                PF12110.4    290 maker-scaffold_7-augustus-gene-0.1398-mRNA-1 -           2003   8.1e-99  330.3   1.6   2   2      0.61   3.3e+03   -3.2   0.0    16    41  1866  1891  1864  1896 0.82 Nuclear protein 96Nucleoporin2         PF04096.10   137 maker-scaffold_7-augustus-gene-0.1398-mRNA-1 -           2003   1.3e-43  148.3   0.0   1   1   4.5e-47   2.4e-43  147.5   0.0     2   137   928  1065   927  1065 0.97 Nucleoporin autopeptidaseNucleoporin_FG       PF13634.2    101 maker-scaffold_7-augustus-gene-0.1398-mRNA-1 -           2003   1.8e-20   73.4 386.9   1  10         3   1.6e+04  -22.2  87.6    10   100    36   126     1   140 0.45 Nucleoporin FG repeat regionNucleoporin_FG       PF13634.2    101 maker-scaffold_7-augustus-gene-0.1398-mRNA-1 -           2003   1.8e-20   73.4 386.9   2  10   9.2e-05       0.5   11.0  46.1     9    89    77   157    75   159 0.73 Nucleoporin FG repeat regionNucleoporin_FG       PF13634.2    101 maker-scaffold_7-augustus-gene-0.1398-mRNA-1 -           2003   1.8e-20   73.4 386.9   3  10     0.012        65    4.1  36.1     5    95   143   229   141   236 0.67 Nucleoporin FG repeat regionNucleoporin_FG       PF13634.2    101 maker-scaffold_7-augustus-gene-0.1398-mRNA-1 -           2003   1.8e-20   73.4 386.9   4  10   3.8e-06     0.021   15.4  40.3     6    87   261   333   257   336 0.83 Nucleoporin FG repeat regionNucleoporin_FG       PF13634.2    101 maker-scaffold_7-augustus-gene-0.1398-mRNA-1 -           2003   1.8e-20   73.4 386.9   5  10   1.6e-06    0.0089   16.6  48.9     5    85   333   410   324   417 0.42 Nucleoporin FG repeat regionNucleoporin_FG       PF13634.2    101 maker-scaffold_7-augustus-gene-0.1398-mRNA-1 -           2003   1.8e-20   73.4 386.9   6  10   3.7e-08    0.0002   21.9  55.8     8   101   378   475   376   476 0.75 Nucleoporin FG repeat regionNucleoporin_FG       PF13634.2    101 maker-scaffold_7-augustus-gene-0.1398-mRNA-1 -           2003   1.8e-20   73.4 386.9   7  10   6.5e-09   3.5e-05   24.3  28.3    35    98   474   534   472   536 0.77 Nucleoporin FG repeat regionNucleoporin_FG       PF13634.2    101 maker-scaffold_7-augustus-gene-0.1398-mRNA-1 -           2003   1.8e-20   73.4 386.9   8  10   1.2e-12   6.5e-09   36.2  47.4     3   101   511   607   510   607 0.84 Nucleoporin FG repeat regionNucleoporin_FG       PF13634.2    101 maker-scaffold_7-augustus-gene-0.1398-mRNA-1 -           2003   1.8e-20   73.4 386.9   9  10   5.5e-06      0.03   14.9  30.9     3    92   596   685   594   697 0.66 Nucleoporin FG repeat regionNucleoporin_FG       PF13634.2    101 maker-scaffold_7-augustus-gene-0.1398-mRNA-1 -           2003   1.8e-20   73.4 386.9  10  10         3   1.6e+04   -5.1   7.1    20    73   723   762   712   809 0.42 Nucleoporin FG repeat regionCOX16                PF14138.2     79 maker-scaffold_7-augustus-gene-0.1435-mRNA-1 -            117   1.8e-30  105.1   0.0   1   1   4.8e-34   2.6e-30  104.6   0.0     2    79    32   110    31   110 0.95 Cytochrome c oxidase assembly protein COX16DUF676               PF05057.10   219 maker-scaffold_7-augustus-gene-0.1435-mRNA-1 -            117      0.16   11.3   0.0   1   1   3.1e-05      0.17   11.3   0.0   114   165    25    74     8    99 0.85 Putative serine esterase (DUF676)Arc_PepC_II          PF06847.7     92 maker-scaffold_7-augustus-gene-0.1435-mRNA-1 -            117      0.17   12.3   0.8   1   2   6.6e-05      0.36   11.2   0.2    62    84    24    46     5    50 0.82 Archaeal Peptidase A24 C-terminus Type IIArc_PepC_II          PF06847.7     92 maker-scaffold_7-augustus-gene-0.1435-mRNA-1 -            117      0.17   12.3   0.8   2   2      0.38   2.1e+03   -0.8   0.0    33    33    71    71    47   105 0.52 Archaeal Peptidase A24 C-terminus Type IIperoxidase           PF00141.19   229 maker-scaffold_7-augustus-gene-0.1370-mRNA-1 -            360   1.5e-48  165.3   0.0   1   1   1.7e-52   2.7e-48  164.5   0.0    12   229   108   324    89   324 0.91 PeroxidaseABC_membrane         PF00664.19   274 augustus_masked-scaffold_7-processed-gene-0.656-mRNA-1 -           1366   1.4e-84  283.9  29.2   1   2   1.3e-45   7.4e-43  147.0   4.9     2   253    54   316    53   336 0.88 ABC transporter transmembrane regionABC_membrane         PF00664.19   274 augustus_masked-scaffold_7-processed-gene-0.656-mRNA-1 -           1366   1.4e-84  283.9  29.2   2   2   2.9e-44   1.6e-41  142.6  16.4     1   271   759  1032   759  1034 0.97 ABC transporter transmembrane regionABC_tran             PF00005.23   137 augustus_masked-scaffold_7-processed-gene-0.656-mRNA-1 -           1366   5.9e-66  221.0   0.0   1   2   1.5e-32   8.6e-30  103.9   0.0     1   137   409   598   409   598 0.95 ABC transporterABC_tran             PF00005.23   137 augustus_masked-scaffold_7-processed-gene-0.656-mRNA-1 -           1366   5.9e-66  221.0   0.0   2   2   7.4e-36   4.1e-33  114.6   0.0     1   135  1125  1274  1125  1276 0.87 ABC transporterSMC_N                PF02463.15   220 augustus_masked-scaffold_7-processed-gene-0.656-mRNA-1 -           1366   1.8e-15   56.9   0.7   1   4     0.013       7.2    5.8   0.1    24    41   419   436   407   442 0.80 RecF/RecN/SMC N terminal domainSMC_N                PF02463.15   220 augustus_masked-scaffold_7-processed-gene-0.656-mRNA-1 -           1366   1.8e-15   56.9   0.7   2   4     1e-06   0.00058   19.2   0.0   113   212   452   642   438   648 0.70 RecF/RecN/SMC N terminal domainSMC_N                PF02463.15   220 augustus_masked-scaffold_7-processed-gene-0.656-mRNA-1 -           1366   1.8e-15   56.9   0.7   3   4     0.013       7.5    5.8   0.0    23    43  1134  1154  1125  1165 0.84 RecF/RecN/SMC N terminal domainSMC_N                PF02463.15   220 augustus_masked-scaffold_7-processed-gene-0.656-mRNA-1 -           1366   1.8e-15   56.9   0.7   4   4   3.5e-07    0.0002   20.8   0.0   120   210  1214  1317  1195  1323 0.79 RecF/RecN/SMC N terminal domainAAA_21               PF13304.2    303 augustus_masked-scaffold_7-processed-gene-0.656-mRNA-1 -           1366   1.3e-09   38.6   0.2   1   3   0.00022      0.12   12.3   0.0     2   276   422   606   421   623 0.63 AAA domain, putative AbiEii toxin, Type IV TA systemAAA_21               PF13304.2    303 augustus_masked-scaffold_7-processed-gene-0.656-mRNA-1 -           1366   1.3e-09   38.6   0.2   2   3   0.00078      0.44   10.6   0.0     2    24  1138  1160  1137  1202 0.77 AAA domain, putative AbiEii toxin, Type IV TA systemAAA_21               PF13304.2    303 augustus_masked-scaffold_7-processed-gene-0.656-mRNA-1 -           1366   1.3e-09   38.6   0.2   3   3     0.012       6.6    6.7   0.0   234   265  1245  1273  1244  1305 0.86 AAA domain, putative AbiEii toxin, Type IV TA systemAAA_29               PF13555.2     61 augustus_masked-scaffold_7-processed-gene-0.656-mRNA-1 -           1366   6.7e-09   35.2   0.1   1   2   7.6e-05     0.042   13.4   0.0    16    39   413   436   408   447 0.79 P-loop containing region of AAA domainAAA_29               PF13555.2     61 augustus_masked-scaffold_7-processed-gene-0.656-mRNA-1 -           1366   6.7e-09   35.2   0.1   2   2   1.6e-06   0.00091   18.8   0.0    13    49  1126  1162  1123  1167 0.84 P-loop containing region of AAA domainAAA_17               PF13207.2    122 augustus_masked-scaffold_7-processed-gene-0.656-mRNA-1 -           1366   7.6e-09   36.6   0.0   1   2     3e-06    0.0017   19.4   0.0     3    29   423   453   423   517 0.80 AAA domainAAA_17               PF13207.2    122 augustus_masked-scaffold_7-processed-gene-0.656-mRNA-1 -           1366   7.6e-09   36.6   0.0   2   2   0.00012      0.07   14.1   0.0     2    23  1138  1159  1137  1240 0.82 AAA domainAAA_16               PF13191.2    177 augustus_masked-scaffold_7-processed-gene-0.656-mRNA-1 -           1366   1.2e-08   35.5   5.9   1   2   1.1e-05    0.0064   16.9   0.7    14   168   407   614   406   623 0.56 AAA ATPase domainAAA_16               PF13191.2    177 augustus_masked-scaffold_7-processed-gene-0.656-mRNA-1 -           1366   1.2e-08   35.5   5.9   2   2   1.1e-06   0.00061   20.2   0.5    25   168  1136  1296  1123  1307 0.62 AAA ATPase domainAPS_kinase           PF01583.16   158 augustus_masked-scaffold_7-processed-gene-0.656-mRNA-1 -           1366   7.8e-08   32.2   0.1   1   2     1e-05    0.0059   16.4   0.0     7    49   424   465   419   485 0.81 Adenylylsulphate kinaseAPS_kinase           PF01583.16   158 augustus_masked-scaffold_7-processed-gene-0.656-mRNA-1 -           1366   7.8e-08   32.2   0.1   2   2   9.3e-05     0.052   13.3   0.0     5    46  1138  1178  1134  1182 0.88 Adenylylsulphate kinaseAAA_30               PF13604.2    196 augustus_masked-scaffold_7-processed-gene-0.656-mRNA-1 -           1366   1.5e-06   28.0   0.9   1   4     0.023        13    5.4   0.0    20    44   421   445   414   466 0.85 AAA domainAAA_30               PF13604.2    196 augustus_masked-scaffold_7-processed-gene-0.656-mRNA-1 -           1366   1.5e-06   28.0   0.9   2   4      0.93   5.2e+02    0.1   0.1    90   118   583   613   515   646 0.70 AAA domainAAA_30               PF13604.2    196 augustus_masked-scaffold_7-processed-gene-0.656-mRNA-1 -           1366   1.5e-06   28.0   0.9   3   4   5.5e-06    0.0031   17.2   0.0    17   105  1134  1277  1129  1302 0.72 AAA domainAAA_30               PF13604.2    196 augustus_masked-scaffold_7-processed-gene-0.656-mRNA-1 -           1366   1.5e-06   28.0   0.9   4   4       2.8   1.5e+03   -1.4   0.2    59   116  1283  1335  1276  1345 0.65 AAA domainAAA_22               PF13401.2    137 augustus_masked-scaffold_7-processed-gene-0.656-mRNA-1 -           1366   3.9e-06   27.0   0.8   1   3    0.0018         1    9.5   0.0     8    37   422   451   418   507 0.71 AAA domainAAA_22               PF13401.2    137 augustus_masked-scaffold_7-processed-gene-0.656-mRNA-1 -           1366   3.9e-06   27.0   0.8   2   3      0.91   5.1e+02    0.7   0.1    53   104   548   601   523   625 0.62 AAA domainAAA_22               PF13401.2    137 augustus_masked-scaffold_7-processed-gene-0.656-mRNA-1 -           1366   3.9e-06   27.0   0.8   3   3   0.00041      0.23   11.6   0.1     6    38  1136  1189  1132  1309 0.57 AAA domainDUF258               PF03193.12   167 augustus_masked-scaffold_7-processed-gene-0.656-mRNA-1 -           1366   1.2e-05   25.1   0.0   1   2     0.023        13    5.5   0.0    99   117   419   437   387   458 0.83 Protein of unknown function, DUF258DUF258               PF03193.12   167 augustus_masked-scaffold_7-processed-gene-0.656-mRNA-1 -           1366   1.2e-05   25.1   0.0   2   2   7.8e-06    0.0044   16.8   0.0    88   121  1123  1157  1109  1176 0.80 Protein of unknown function, DUF258Zeta_toxin           PF06414.8    199 augustus_masked-scaffold_7-processed-gene-0.656-mRNA-1 -           1366   8.6e-05   21.8   0.1   1   2   0.00059      0.33   10.1   0.0    21    56   424   459   419   467 0.91 Zeta toxinZeta_toxin           PF06414.8    199 augustus_masked-scaffold_7-processed-gene-0.656-mRNA-1 -           1366   8.6e-05   21.8   0.1   2   2    0.0013      0.71    9.0   0.0    19    53  1138  1172  1125  1188 0.88 Zeta toxinAAA_23               PF13476.2    200 augustus_masked-scaffold_7-processed-gene-0.656-mRNA-1 -           1366   0.00018   21.9   0.0   1   2     0.018        10    6.4   0.0    11    36   410   436   406   442 0.84 AAA domainAAA_23               PF13476.2    200 augustus_masked-scaffold_7-processed-gene-0.656-mRNA-1 -           1366   0.00018   21.9   0.0   2   2   0.00021      0.12   12.7   0.0    12    39  1127  1155  1122  1177 0.83 AAA domainAAA_10               PF12846.3    304 augustus_masked-scaffold_7-processed-gene-0.656-mRNA-1 -           1366   0.00023   20.8   0.8   1   3     0.005       2.8    7.4   0.0     3    19   421   437   419   480 0.92 AAA-like domainAAA_10               PF12846.3    304 augustus_masked-scaffold_7-processed-gene-0.656-mRNA-1 -           1366   0.00023   20.8   0.8   2   3      0.43   2.4e+02    1.0   0.1   218   270   585   636   568   652 0.81 AAA-like domainAAA_10               PF12846.3    304 augustus_masked-scaffold_7-processed-gene-0.656-mRNA-1 -           1366   0.00023   20.8   0.8   3   3    0.0021       1.2    8.6   0.0     4    21  1138  1155  1135  1172 0.84 AAA-like domainAAA_25               PF13481.2    194 augustus_masked-scaffold_7-processed-gene-0.656-mRNA-1 -           1366   0.00024   20.7   0.5   1   4    0.0018         1    8.8   0.0    29    51   415   437   391   439 0.84 AAA domainAAA_25               PF13481.2    194 augustus_masked-scaffold_7-processed-gene-0.656-mRNA-1 -           1366   0.00024   20.7   0.5   2   4       1.3   7.5e+02   -0.5   0.0   140   161   585   607   541   638 0.71 AAA domainAAA_25               PF13481.2    194 augustus_masked-scaffold_7-processed-gene-0.656-mRNA-1 -           1366   0.00024   20.7   0.5   3   4    0.0059       3.3    7.1   0.0    30    50  1132  1152  1106  1174 0.80 AAA domainAAA_25               PF13481.2    194 augustus_masked-scaffold_7-processed-gene-0.656-mRNA-1 -           1366   0.00024   20.7   0.5   4   4       5.7   3.2e+03   -2.6   0.0   134   176  1259  1301  1251  1309 0.58 AAA domainABC_ATPase           PF09818.5    448 augustus_masked-scaffold_7-processed-gene-0.656-mRNA-1 -           1366    0.0007   18.4   0.7   1   3       8.4   4.7e+03   -4.1   0.0   241   263   415   437   412   444 0.75 Predicted ATPase of the ABC classABC_ATPase           PF09818.5    448 augustus_masked-scaffold_7-processed-gene-0.656-mRNA-1 -           1366    0.0007   18.4   0.7   2   3     0.044        25    3.4   0.1   322   396   569   642   540   650 0.77 Predicted ATPase of the ABC classABC_ATPase           PF09818.5    448 augustus_masked-scaffold_7-processed-gene-0.656-mRNA-1 -           1366    0.0007   18.4   0.7   3   3   6.7e-05     0.037   12.7   0.0   300   354  1224  1279  1210  1355 0.80 Predicted ATPase of the ABC classAAA_33               PF13671.2    143 augustus_masked-scaffold_7-processed-gene-0.656-mRNA-1 -           1366    0.0016   18.5   0.0   1   2    0.0031       1.8    8.6   0.0     4    17   424   437   422   487 0.86 AAA domainAAA_33               PF13671.2    143 augustus_masked-scaffold_7-processed-gene-0.656-mRNA-1 -           1366    0.0016   18.5   0.0   2   2     0.011       5.9    6.9   0.0     4    17  1140  1153  1137  1184 0.81 AAA domainDUF3987              PF13148.2    363 augustus_masked-scaffold_7-processed-gene-0.656-mRNA-1 -           1366    0.0025   16.8   0.0   1   2    0.0063       3.6    6.5   0.0    37    62   417   442   407   446 0.82 Protein of unknown function (DUF3987)DUF3987              PF13148.2    363 augustus_masked-scaffold_7-processed-gene-0.656-mRNA-1 -           1366    0.0025   16.8   0.0   2   2    0.0026       1.4    7.8   0.0    39    68  1135  1164  1120  1172 0.87 Protein of unknown function (DUF3987)AAA_18               PF13238.2    129 augustus_masked-scaffold_7-processed-gene-0.656-mRNA-1 -           1366    0.0046   17.3   0.3   1   2    0.0077       4.3    7.7   0.0     3    18   424   439   423   472 0.78 AAA domainAAA_18               PF13238.2    129 augustus_masked-scaffold_7-processed-gene-0.656-mRNA-1 -           1366    0.0046   17.3   0.3   2   2     0.012       6.4    7.1   0.1     1    19  1138  1156  1138  1183 0.86 AAA domainAAA                  PF00004.25   132 augustus_masked-scaffold_7-processed-gene-0.656-mRNA-1 -           1366    0.0057   16.9   0.5   1   4      0.11        64    3.8   0.0     3    35   424   458   422   479 0.76 ATPase family associated with various cellular activities (AAA)AAA                  PF00004.25   132 augustus_masked-scaffold_7-processed-gene-0.656-mRNA-1 -           1366    0.0057   16.9   0.5   2   4      0.37   2.1e+02    2.2   0.0    46    98   576   634   547   650 0.64 ATPase family associated with various cellular activities (AAA)AAA                  PF00004.25   132 augustus_masked-scaffold_7-processed-gene-0.656-mRNA-1 -           1366    0.0057   16.9   0.5   3   4     0.055        31    4.9   0.0     3    21  1140  1158  1138  1202 0.83 ATPase family associated with various cellular activities (AAA)AAA                  PF00004.25   132 augustus_masked-scaffold_7-processed-gene-0.656-mRNA-1 -           1366    0.0057   16.9   0.5   4   4       1.7   9.5e+02    0.0   0.0    51   118  1259  1312  1210  1349 0.74 ATPase family associated with various cellular activities (AAA)PRK                  PF00485.14   197 augustus_masked-scaffold_7-processed-gene-0.656-mRNA-1 -           1366    0.0093   15.6   0.1   1   2     0.091        51    3.4   0.0     3    24   423   444   422   465 0.75 Phosphoribulokinase / Uridine kinase familyPRK                  PF00485.14   197 augustus_masked-scaffold_7-processed-gene-0.656-mRNA-1 -           1366    0.0093   15.6   0.1   2   2    0.0012      0.66    9.5   0.0     2    39  1138  1175  1137  1189 0.71 Phosphoribulokinase / Uridine kinase familySbcCD_C              PF13558.2     90 augustus_masked-scaffold_7-processed-gene-0.656-mRNA-1 -           1366     0.011   15.8   2.1   1   2     0.024        13    5.9   0.1    63    82   587   606   566   613 0.79 Putative exonuclease SbcCD, C subunitSbcCD_C              PF13558.2     90 augustus_masked-scaffold_7-processed-gene-0.656-mRNA-1 -           1366     0.011   15.8   2.1   2   2     0.013       7.2    6.8   0.2    19    84  1234  1286  1226  1292 0.69 Putative exonuclease SbcCD, C subunitAAA_5                PF07728.10   139 augustus_masked-scaffold_7-processed-gene-0.656-mRNA-1 -           1366     0.013   15.3   0.1   1   3     0.075        42    3.9   0.0     4    30   424   451   422   483 0.77 AAA domain (dynein-related subfamily)AAA_5                PF07728.10   139 augustus_masked-scaffold_7-processed-gene-0.656-mRNA-1 -           1366     0.013   15.3   0.1   2   3       1.9     1e+03   -0.6   0.0    60    83   581   605   566   651 0.80 AAA domain (dynein-related subfamily)AAA_5                PF07728.10   139 augustus_masked-scaffold_7-processed-gene-0.656-mRNA-1 -           1366     0.013   15.3   0.1   3   3     0.011       6.3    6.6   0.0     4    27  1140  1165  1138  1183 0.80 AAA domain (dynein-related subfamily)NB-ARC               PF00931.18   288 augustus_masked-scaffold_7-processed-gene-0.656-mRNA-1 -           1366     0.013   14.4   0.1   1   2     0.046        26    3.6   0.0    22    38   422   438   412   443 0.87 NB-ARC domainNB-ARC               PF00931.18   288 augustus_masked-scaffold_7-processed-gene-0.656-mRNA-1 -           1366     0.013   14.4   0.1   2   2    0.0018         1    8.2   0.0    17    39  1133  1155  1122  1164 0.82 NB-ARC domainMMR_HSR1             PF01926.19   110 augustus_masked-scaffold_7-processed-gene-0.656-mRNA-1 -           1366     0.015   15.3   0.3   1   2      0.14        78    3.3   0.0     3    16   423   436   421   464 0.85 50S ribosome-binding GTPaseMMR_HSR1             PF01926.19   110 augustus_masked-scaffold_7-processed-gene-0.656-mRNA-1 -           1366     0.015   15.3   0.3   2   2    0.0018      0.99    9.4   0.0     2    19  1138  1155  1137  1177 0.83 50S ribosome-binding GTPaseDUF87                PF01935.13   229 augustus_masked-scaffold_7-processed-gene-0.656-mRNA-1 -           1366     0.015   15.2   0.5   1   2   0.00079      0.44   10.4   0.1    26    59   422   453   419   456 0.87 Domain of unknown function DUF87DUF87                PF01935.13   229 augustus_masked-scaffold_7-processed-gene-0.656-mRNA-1 -           1366     0.015   15.2   0.5   2   2      0.22   1.2e+02    2.4   0.0    26    48  1138  1159  1127  1168 0.78 Domain of unknown function DUF87AAA_14               PF13173.2    131 augustus_masked-scaffold_7-processed-gene-0.656-mRNA-1 -           1366     0.053   13.4   0.0   1   2     0.057        32    4.4   0.0     5    52   422   468   418   482 0.77 AAA domainAAA_14               PF13173.2    131 augustus_masked-scaffold_7-processed-gene-0.656-mRNA-1 -           1366     0.053   13.4   0.0   2   2     0.019        11    6.0   0.0     2    40  1135  1172  1134  1188 0.77 AAA domainSemialdhyde_dhC      PF02774.14   184 augustus_masked-scaffold_7-processed-gene-0.656-mRNA-1 -           1366      0.11   12.4   0.0   1   3       4.6   2.6e+03   -1.8   0.0    96   118   249   270   230   287 0.67 Semialdehyde dehydrogenase, dimerisation domainSemialdhyde_dhC      PF02774.14   184 augustus_masked-scaffold_7-processed-gene-0.656-mRNA-1 -           1366      0.11   12.4   0.0   2   3       0.5   2.8e+02    1.3   0.0    16    37   421   451   414   477 0.72 Semialdehyde dehydrogenase, dimerisation domainSemialdhyde_dhC      PF02774.14   184 augustus_masked-scaffold_7-processed-gene-0.656-mRNA-1 -           1366      0.11   12.4   0.0   3   3    0.0079       4.4    7.2   0.0    20    92  1141  1235  1125  1245 0.88 Semialdehyde dehydrogenase, dimerisation domainATP_bind_1           PF03029.13   238 augustus_masked-scaffold_7-processed-gene-0.656-mRNA-1 -           1366      0.22   11.1   0.6   1   2     0.091        51    3.4   0.0     1    20   424   443   424   456 0.72 Conserved hypothetical ATP binding proteinATP_bind_1           PF03029.13   238 augustus_masked-scaffold_7-processed-gene-0.656-mRNA-1 -           1366      0.22   11.1   0.6   2   2     0.023        13    5.3   0.1     2    21  1141  1160  1140  1168 0.85 Conserved hypothetical ATP binding proteinFungal_trans         PF04082.14   267 maker-scaffold_7-augustus-gene-0.1407-mRNA-1 -            837     6e-13   48.3   0.1   1   1   4.7e-16   1.5e-12   47.0   0.1    36   190   327   467   304   503 0.83 Fungal specific transcription factor domainZn_clus              PF00172.14    40 maker-scaffold_7-augustus-gene-0.1407-mRNA-1 -            837   4.2e-05   23.5  11.0   1   1   2.2e-08   7.3e-05   22.7  11.0     5    34    90   117    89   120 0.92 Fungal Zn(2)-Cys(6) binuclear cluster domainMenin                PF05053.9    680 maker-scaffold_7-augustus-gene-0.1407-mRNA-1 -            837     0.013   13.8   1.8   1   1   8.4e-06     0.027   12.7   1.8   494   582   676   763   657   787 0.65 MeninVac_ImportDeg        PF09783.5    162 maker-scaffold_7-augustus-gene-0.1407-mRNA-1 -            837       0.1   12.3   0.0   1   1   9.7e-05      0.32   10.7   0.0    36   101   415   502   407   532 0.75 Vacuolar import and degradation proteinArAE_1_C             PF11728.4    166 maker-scaffold_7-augustus-gene-0.1407-mRNA-1 -            837       3.7    7.4   7.4   1   2   2.8e-05     0.092   12.6   0.6    73   133   550   612   495   617 0.88 Putative aromatic acid exporter C-terminal domainArAE_1_C             PF11728.4    166 maker-scaffold_7-augustus-gene-0.1407-mRNA-1 -            837       3.7    7.4   7.4   2   2         5   1.6e+04   -5.1   1.1    54    63   721   730   703   739 0.48 Putative aromatic acid exporter C-terminal domainPEPCK_ATP            PF01293.16   465 maker-scaffold_7-snap-gene-0.818-mRNA-1 -            561  2.7e-215  715.3   0.0   1   1  3.8e-219  3.1e-215  715.1   0.0     3   465    47   513    45   513 0.99 Phosphoenolpyruvate carboxykinaseAAA_18               PF13238.2    129 maker-scaffold_7-snap-gene-0.818-mRNA-1 -            561     0.013   15.9   0.0   1   2   0.00088       7.1    7.0   0.0    37   100   100   157    86   175 0.75 AAA domainAAA_18               PF13238.2    129 maker-scaffold_7-snap-gene-0.818-mRNA-1 -            561     0.013   15.9   0.0   2   2    0.0013        11    6.4   0.0     3    40   261   312   260   350 0.68 AAA domainFHA                  PF00498.22    69 augustus_masked-scaffold_7-processed-gene-0.642-mRNA-1 -            779     4e-17   62.2   0.1   1   1   3.3e-20   1.3e-16   60.5   0.1     2    69   200   277   199   277 0.88 FHA domainYhfZ_C               PF14503.2    236 augustus_masked-scaffold_7-processed-gene-0.642-mRNA-1 -            779    0.0023   17.4   0.1   1   1   1.4e-06    0.0055   16.1   0.1    61   136   215   295   207   309 0.82 YhfZ C-terminal domainEMP24_GP25L          PF01105.20   182 augustus_masked-scaffold_7-processed-gene-0.642-mRNA-1 -            779     0.033   14.0   2.7   1   1     2e-05      0.08   12.7   2.7    51   152   531   644   489   648 0.74 emp24/gp25L/p24 family/GOLDXhlA                 PF10779.5     67 augustus_masked-scaffold_7-processed-gene-0.642-mRNA-1 -            779      0.16   12.1   1.3   1   1   0.00013      0.55   10.3   1.3     3    44   602   643   600   647 0.87 Haemolysin XhlAKH_1                 PF00013.25    62 augustus_masked-scaffold_7-processed-gene-0.626-mRNA-1 -           1269   1.8e-90  296.5  26.2   1  14    0.0024       7.8    6.2   0.0    21    60   144   182   143   184 0.87 KH domainKH_1                 PF00013.25    62 augustus_masked-scaffold_7-processed-gene-0.626-mRNA-1 -           1269   1.8e-90  296.5  26.2   2  14   9.6e-16   3.1e-12   45.9   0.4     2    53   194   254   193   263 0.74 KH domainKH_1                 PF00013.25    62 augustus_masked-scaffold_7-processed-gene-0.626-mRNA-1 -           1269   1.8e-90  296.5  26.2   3  14   1.4e-06    0.0045   16.6   0.0     6    62   281   354   278   354 0.90 KH domainKH_1                 PF00013.25    62 augustus_masked-scaffold_7-processed-gene-0.626-mRNA-1 -           1269   1.8e-90  296.5  26.2   4  14   7.4e-06     0.024   14.2   0.0     3    56   369   420   367   424 0.90 KH domainKH_1                 PF00013.25    62 augustus_masked-scaffold_7-processed-gene-0.626-mRNA-1 -           1269   1.8e-90  296.5  26.2   5  14      0.55   1.8e+03   -1.4   0.0    25    39   541   555   540   570 0.75 KH domainKH_1                 PF00013.25    62 augustus_masked-scaffold_7-processed-gene-0.626-mRNA-1 -           1269   1.8e-90  296.5  26.2   6  14      0.43   1.4e+03   -1.0   0.0    43    57   650   664   634   667 0.75 KH domainKH_1                 PF00013.25    62 augustus_masked-scaffold_7-processed-gene-0.626-mRNA-1 -           1269   1.8e-90  296.5  26.2   7  14   1.4e-13   4.5e-10   39.0   0.0     1    62   686   741   686   741 0.93 KH domainKH_1                 PF00013.25    62 augustus_masked-scaffold_7-processed-gene-0.626-mRNA-1 -           1269   1.8e-90  296.5  26.2   8  14   1.4e-09   4.6e-06   26.1   0.0     2    62   754   841   753   841 0.84 KH domainKH_1                 PF00013.25    62 augustus_masked-scaffold_7-processed-gene-0.626-mRNA-1 -           1269   1.8e-90  296.5  26.2   9  14   1.4e-15   4.5e-12   45.4   0.3     1    61   854   914   854   915 0.88 KH domainKH_1                 PF00013.25    62 augustus_masked-scaffold_7-processed-gene-0.626-mRNA-1 -           1269   1.8e-90  296.5  26.2  10  14   2.1e-06     0.007   16.0   0.6     1    30   927   956   927   996 0.66 KH domainKH_1                 PF00013.25    62 augustus_masked-scaffold_7-processed-gene-0.626-mRNA-1 -           1269   1.8e-90  296.5  26.2  11  14   1.4e-14   4.4e-11   42.2   0.5     1    62  1008  1068  1008  1068 0.94 KH domainKH_1                 PF00013.25    62 augustus_masked-scaffold_7-processed-gene-0.626-mRNA-1 -           1269   1.8e-90  296.5  26.2  12  14       1.1   3.5e+03   -2.3   0.1     8    20  1119  1131  1116  1132 0.85 KH domainKH_1                 PF00013.25    62 augustus_masked-scaffold_7-processed-gene-0.626-mRNA-1 -           1269   1.8e-90  296.5  26.2  13  14      0.26   8.4e+02   -0.3   0.0    47    62  1164  1180  1147  1180 0.83 KH domainKH_1                 PF00013.25    62 augustus_masked-scaffold_7-processed-gene-0.626-mRNA-1 -           1269   1.8e-90  296.5  26.2  14  14   4.8e-16   1.6e-12   46.9   0.1     8    62  1201  1254  1196  1254 0.91 KH domainKH_3                 PF13014.2     43 augustus_masked-scaffold_7-processed-gene-0.626-mRNA-1 -           1269   6.2e-63  207.7  17.3   1  13      0.42   1.4e+03   -0.9   0.0    12    28   144   160   143   175 0.83 KH domainKH_3                 PF13014.2     43 augustus_masked-scaffold_7-processed-gene-0.626-mRNA-1 -           1269   6.2e-63  207.7  17.3   2  13   4.2e-14   1.4e-10   40.6   0.2     5    37   206   243   203   248 0.79 KH domainKH_3                 PF13014.2     43 augustus_masked-scaffold_7-processed-gene-0.626-mRNA-1 -           1269   6.2e-63  207.7  17.3   3  13     0.095   3.1e+02    1.2   0.0     7    28   291   312   285   340 0.78 KH domainKH_3                 PF13014.2     43 augustus_masked-scaffold_7-processed-gene-0.626-mRNA-1 -           1269   6.2e-63  207.7  17.3   4  13   0.00016      0.52   10.0   0.0     2    43   377   414   376   414 0.96 KH domainKH_3                 PF13014.2     43 augustus_masked-scaffold_7-processed-gene-0.626-mRNA-1 -           1269   6.2e-63  207.7  17.3   5  13     0.056   1.8e+02    1.9   0.0    17    39   542   561   540   564 0.77 KH domainKH_3                 PF13014.2     43 augustus_masked-scaffold_7-processed-gene-0.626-mRNA-1 -           1269   6.2e-63  207.7  17.3   6  13   1.1e-08   3.7e-05   23.3   0.0     4    28   698   722   695   736 0.88 KH domainKH_3                 PF13014.2     43 augustus_masked-scaffold_7-processed-gene-0.626-mRNA-1 -           1269   6.2e-63  207.7  17.3   7  13   1.2e-07   0.00039   20.0   0.0     1    32   762   796   762   802 0.83 KH domainKH_3                 PF13014.2     43 augustus_masked-scaffold_7-processed-gene-0.626-mRNA-1 -           1269   6.2e-63  207.7  17.3   8  13   1.5e-11     5e-08   32.4   0.1     1    41   863   899   863   903 0.87 KH domainKH_3                 PF13014.2     43 augustus_masked-scaffold_7-processed-gene-0.626-mRNA-1 -           1269   6.2e-63  207.7  17.3   9  13   0.00013      0.43   10.3   0.0     1    17   936   952   936   956 0.89 KH domainKH_3                 PF13014.2     43 augustus_masked-scaffold_7-processed-gene-0.626-mRNA-1 -           1269   6.2e-63  207.7  17.3  10  13      0.62     2e+03   -1.4   0.0    24    43   968   984   966   984 0.75 KH domainKH_3                 PF13014.2     43 augustus_masked-scaffold_7-processed-gene-0.626-mRNA-1 -           1269   6.2e-63  207.7  17.3  11  13   7.1e-10   2.3e-06   27.1   0.3     1    43  1017  1056  1017  1056 0.94 KH domainKH_3                 PF13014.2     43 augustus_masked-scaffold_7-processed-gene-0.626-mRNA-1 -           1269   6.2e-63  207.7  17.3  12  13       2.3   7.6e+03   -3.3   0.0     9    24  1093  1108  1093  1109 0.88 KH domainKH_3                 PF13014.2     43 augustus_masked-scaffold_7-processed-gene-0.626-mRNA-1 -           1269   6.2e-63  207.7  17.3  13  13   2.9e-13   9.6e-10   37.9   0.3     2    43  1204  1242  1203  1242 0.97 KH domainKH_2                 PF07650.13    78 augustus_masked-scaffold_7-processed-gene-0.626-mRNA-1 -           1269     1e-11   44.4  26.2   1   8   4.4e-06     0.014   15.0   1.0    16    59   186   226   175   231 0.83 KH domainKH_2                 PF07650.13    78 augustus_masked-scaffold_7-processed-gene-0.626-mRNA-1 -           1269     1e-11   44.4  26.2   2   8   4.3e-05      0.14   11.9   0.1    39    69   699   728   696   733 0.90 KH domainKH_2                 PF07650.13    78 augustus_masked-scaffold_7-processed-gene-0.626-mRNA-1 -           1269     1e-11   44.4  26.2   3   8     0.096   3.1e+02    1.1   0.0    39    53   766   780   763   787 0.87 KH domainKH_2                 PF07650.13    78 augustus_masked-scaffold_7-processed-gene-0.626-mRNA-1 -           1269     1e-11   44.4  26.2   4   8   6.3e-06     0.021   14.5   0.0    23    64   851   892   847   908 0.84 KH domainKH_2                 PF07650.13    78 augustus_masked-scaffold_7-processed-gene-0.626-mRNA-1 -           1269     1e-11   44.4  26.2   5   8     0.047   1.5e+02    2.1   0.1    26    54   927   955   899   958 0.71 KH domainKH_2                 PF07650.13    78 augustus_masked-scaffold_7-processed-gene-0.626-mRNA-1 -           1269     1e-11   44.4  26.2   6   8    0.0046        15    5.4   0.7    15    55   996  1037   983  1055 0.81 KH domainKH_2                 PF07650.13    78 augustus_masked-scaffold_7-processed-gene-0.626-mRNA-1 -           1269     1e-11   44.4  26.2   7   8       2.6   8.4e+03   -3.4   0.1    44    63  1170  1189  1167  1193 0.66 KH domainKH_2                 PF07650.13    78 augustus_masked-scaffold_7-processed-gene-0.626-mRNA-1 -           1269     1e-11   44.4  26.2   8   8    0.0047        15    5.3   0.0    38    59  1206  1227  1201  1240 0.87 KH domainSLS                  PF14611.2    212 augustus_masked-scaffold_7-processed-gene-0.626-mRNA-1 -           1269   3.6e-08   33.4   5.3   1   9      0.28   9.2e+02   -0.6   0.1    71   126   172   247   143   317 0.59 Mitochondrial inner-membrane-bound regulatorSLS                  PF14611.2    212 augustus_masked-scaffold_7-processed-gene-0.626-mRNA-1 -           1269   3.6e-08   33.4   5.3   2   9      0.36   1.2e+03   -1.0   0.0    60    90   331   361   326   381 0.84 Mitochondrial inner-membrane-bound regulatorSLS                  PF14611.2    212 augustus_masked-scaffold_7-processed-gene-0.626-mRNA-1 -           1269   3.6e-08   33.4   5.3   3   9      0.21     7e+02   -0.2   0.0   110   126   465   491   404   643 0.64 Mitochondrial inner-membrane-bound regulatorSLS                  PF14611.2    212 augustus_masked-scaffold_7-processed-gene-0.626-mRNA-1 -           1269   3.6e-08   33.4   5.3   4   9   7.5e-05      0.24   11.1   0.0    29   127   689   792   672   813 0.75 Mitochondrial inner-membrane-bound regulatorSLS                  PF14611.2    212 augustus_masked-scaffold_7-processed-gene-0.626-mRNA-1 -           1269   3.6e-08   33.4   5.3   5   9     0.018        57    3.3   0.0    28    76   856   908   815   915 0.59 Mitochondrial inner-membrane-bound regulatorSLS                  PF14611.2    212 augustus_masked-scaffold_7-processed-gene-0.626-mRNA-1 -           1269   3.6e-08   33.4   5.3   6   9     0.008        26    4.4   0.0    28    84   856   916   838   995 0.79 Mitochondrial inner-membrane-bound regulatorSLS                  PF14611.2    212 augustus_masked-scaffold_7-processed-gene-0.626-mRNA-1 -           1269   3.6e-08   33.4   5.3   7   9    0.0067        22    4.7   0.1    28    96   925  1009   899  1037 0.80 Mitochondrial inner-membrane-bound regulatorSLS                  PF14611.2    212 augustus_masked-scaffold_7-processed-gene-0.626-mRNA-1 -           1269   3.6e-08   33.4   5.3   8   9     0.015        49    3.5   0.0    66   101  1051  1086  1046  1122 0.76 Mitochondrial inner-membrane-bound regulatorSLS                  PF14611.2    212 augustus_masked-scaffold_7-processed-gene-0.626-mRNA-1 -           1269   3.6e-08   33.4   5.3   9   9     0.029        95    2.6   0.0    34    87  1202  1258  1174  1264 0.72 Mitochondrial inner-membrane-bound regulatorKH_4                 PF13083.2     73 augustus_masked-scaffold_7-processed-gene-0.626-mRNA-1 -           1269     0.068   12.9  16.9   1   8    0.0078        25    4.7   0.3    35    55   198   218   185   220 0.82 KH domainKH_4                 PF13083.2     73 augustus_masked-scaffold_7-processed-gene-0.626-mRNA-1 -           1269     0.068   12.9  16.9   2   8     0.032   1.1e+02    2.7   0.0    19    55   672   711   660   712 0.59 KH domainKH_4                 PF13083.2     73 augustus_masked-scaffold_7-processed-gene-0.626-mRNA-1 -           1269     0.068   12.9  16.9   3   8      0.96   3.1e+03   -2.0   0.0    33    56   756   779   747   780 0.73 KH domainKH_4                 PF13083.2     73 augustus_masked-scaffold_7-processed-gene-0.626-mRNA-1 -           1269     0.068   12.9  16.9   4   8   0.00087       2.8    7.7   0.0    25    56   849   880   842   886 0.88 KH domainKH_4                 PF13083.2     73 augustus_masked-scaffold_7-processed-gene-0.626-mRNA-1 -           1269     0.068   12.9  16.9   5   8     0.094   3.1e+02    1.2   0.3     4    52   901   949   898   951 0.89 KH domainKH_4                 PF13083.2     73 augustus_masked-scaffold_7-processed-gene-0.626-mRNA-1 -           1269     0.068   12.9  16.9   6   8     0.085   2.8e+02    1.4   0.5    10    50   991  1028   986  1030 0.60 KH domainKH_4                 PF13083.2     73 augustus_masked-scaffold_7-processed-gene-0.626-mRNA-1 -           1269     0.068   12.9  16.9   7   8       2.4   7.8e+03   -3.3   0.0    37    48  1119  1130  1117  1131 0.83 KH domainKH_4                 PF13083.2     73 augustus_masked-scaffold_7-processed-gene-0.626-mRNA-1 -           1269     0.068   12.9  16.9   8   8      0.74   2.4e+03   -1.6   0.0    42    55  1206  1219  1171  1220 0.77 KH domainUQ_con               PF00179.22   140 maker-scaffold_7-augustus-gene-0.1412-mRNA-1 -            267   4.8e-19   68.2   0.1   1   1   4.1e-23   6.7e-19   67.8   0.1     1   112     9   119     9   139 0.91 Ubiquitin-conjugating enzymePALP                 PF00291.21   293 maker-scaffold_7-augustus-gene-0.1443-mRNA-1 -            346   2.5e-40  138.6   0.4   1   1   1.9e-44   3.1e-40  138.3   0.4     5   291    20   333    17   335 0.84 Pyridoxal-phosphate dependent enzymeAltA1                PF16541.1    108 maker-scaffold_7-augustus-gene-0.1373-mRNA-1 -            171   3.7e-05   24.4   0.8   1   1   3.2e-09   5.3e-05   23.9   0.8     1   100    46   145    46   156 0.78 Alternaria alternata allergen 1ABC2_membrane        PF01061.20   210 augustus_masked-scaffold_7-processed-gene-0.607-mRNA-1 -           1486   2.5e-72  242.6  53.0   1   3   5.9e-39   3.8e-36  124.3  24.7     2   210   501   710   500   710 0.98 ABC-2 type transporterABC2_membrane        PF01061.20   210 augustus_masked-scaffold_7-processed-gene-0.607-mRNA-1 -           1486   2.5e-72  242.6  53.0   2   3       3.4   2.2e+03   -2.3   0.1    17    67   769   785   750   806 0.53 ABC-2 type transporterABC2_membrane        PF01061.20   210 augustus_masked-scaffold_7-processed-gene-0.607-mRNA-1 -           1486   2.5e-72  242.6  53.0   3   3   6.6e-40   4.3e-37  127.4  16.3     2   207  1166  1373  1165  1376 0.95 ABC-2 type transporterABC_tran             PF00005.23   137 augustus_masked-scaffold_7-processed-gene-0.607-mRNA-1 -           1486   1.5e-34  119.3   0.0   1   2   2.2e-18   1.4e-15   57.9   0.0     4   136   179   334   176   335 0.88 ABC transporterABC_tran             PF00005.23   137 augustus_masked-scaffold_7-processed-gene-0.607-mRNA-1 -           1486   1.5e-34  119.3   0.0   2   2   7.9e-19   5.1e-16   59.3   0.0     1   137   864  1015   864  1015 0.90 ABC transporterPDR_CDR              PF06422.8     98 augustus_masked-scaffold_7-processed-gene-0.607-mRNA-1 -           1486   9.8e-29   99.0   0.0   1   2   5.4e-28   3.5e-25   87.6   0.0     1    92   722   811   722   816 0.95 CDR ABC transporterPDR_CDR              PF06422.8     98 augustus_masked-scaffold_7-processed-gene-0.607-mRNA-1 -           1486   9.8e-29   99.0   0.0   2   2    0.0038       2.4    8.0   0.0    32    69  1431  1467  1426  1473 0.87 CDR ABC transporterAAA_16               PF13191.2    177 augustus_masked-scaffold_7-processed-gene-0.607-mRNA-1 -           1486   2.4e-07   31.3   0.6   1   2    0.0034       2.2    8.6   0.0     4    50   167   210   166   237 0.81 AAA ATPase domainAAA_16               PF13191.2    177 augustus_masked-scaffold_7-processed-gene-0.607-mRNA-1 -           1486   2.4e-07   31.3   0.6   2   2   9.6e-07   0.00063   20.1   0.1    12   150   864  1012   859  1046 0.55 AAA ATPase domainAAA_21               PF13304.2    303 augustus_masked-scaffold_7-processed-gene-0.607-mRNA-1 -           1486   5.1e-06   26.7   0.0   1   2   8.8e-05     0.057   13.5   0.0     1    25   876   897   876   948 0.77 AAA domain, putative AbiEii toxin, Type IV TA systemAAA_21               PF13304.2    303 augustus_masked-scaffold_7-processed-gene-0.607-mRNA-1 -           1486   5.1e-06   26.7   0.0   2   2   0.00073      0.47   10.4   0.0   259   296  1006  1042   992  1042 0.90 AAA domain, putative AbiEii toxin, Type IV TA systemAAA_25               PF13481.2    194 augustus_masked-scaffold_7-processed-gene-0.607-mRNA-1 -           1486   7.3e-06   25.6   0.1   1   2    0.0069       4.4    6.7   0.0    15    56   166   209   153   256 0.76 AAA domainAAA_25               PF13481.2    194 augustus_masked-scaffold_7-processed-gene-0.607-mRNA-1 -           1486   7.3e-06   25.6   0.1   2   2     1e-05    0.0065   16.0   0.0    26    55   867   896   854   919 0.82 AAA domainAAA_33               PF13671.2    143 augustus_masked-scaffold_7-processed-gene-0.607-mRNA-1 -           1486   8.8e-06   25.8   0.3   1   3    0.0063       4.1    7.4   0.0     1    39   188   227   188   304 0.70 AAA domainAAA_33               PF13671.2    143 augustus_masked-scaffold_7-processed-gene-0.607-mRNA-1 -           1486   8.8e-06   25.8   0.3   2   3       7.7     5e+03   -2.6   0.0   107   140   475   510   473   512 0.73 AAA domainAAA_33               PF13671.2    143 augustus_masked-scaffold_7-processed-gene-0.607-mRNA-1 -           1486   8.8e-06   25.8   0.3   3   3   2.7e-05     0.018   15.1   0.0     2    66   877   948   876   962 0.70 AAA domainABC_trans_N          PF14510.2     85 augustus_masked-scaffold_7-processed-gene-0.607-mRNA-1 -           1486   7.8e-05   22.7   0.0   1   1   2.9e-07   0.00019   21.4   0.0    30    84    99   151    61   152 0.81 ABC-transporter extracellular N-terminalAAA_29               PF13555.2     61 augustus_masked-scaffold_7-processed-gene-0.607-mRNA-1 -           1486   0.00023   20.6   1.0   1   2       0.1        66    3.2   0.1    22    41   186   205   178   208 0.82 P-loop containing region of AAA domainAAA_29               PF13555.2     61 augustus_masked-scaffold_7-processed-gene-0.607-mRNA-1 -           1486   0.00023   20.6   1.0   2   2   1.6e-05      0.01   15.4   0.1    22    42   874   894   869   896 0.84 P-loop containing region of AAA domainABC2_membrane_3      PF12698.3    345 augustus_masked-scaffold_7-processed-gene-0.607-mRNA-1 -           1486   0.00024   20.2  36.1   1   3       2.8   1.8e+03   -2.4   0.1   210   245   520   561   519   585 0.75 ABC-2 family transporter proteinABC2_membrane_3      PF12698.3    345 augustus_masked-scaffold_7-processed-gene-0.607-mRNA-1 -           1486   0.00024   20.2  36.1   2   3   1.5e-06   0.00097   18.2  14.8   201   344   590   785   581   786 0.79 ABC-2 family transporter proteinABC2_membrane_3      PF12698.3    345 augustus_masked-scaffold_7-processed-gene-0.607-mRNA-1 -           1486   0.00024   20.2  36.1   3   3   0.00019      0.12   11.3   9.1   216   314  1270  1372  1217  1465 0.82 ABC-2 family transporter proteinDUF258               PF03193.12   167 augustus_masked-scaffold_7-processed-gene-0.607-mRNA-1 -           1486   0.00043   20.1   0.2   1   2       3.3   2.2e+03   -1.7   0.1   100   123   187   210   180   220 0.85 Protein of unknown function, DUF258DUF258               PF03193.12   167 augustus_masked-scaffold_7-processed-gene-0.607-mRNA-1 -           1486   0.00043   20.1   0.2   2   2     3e-06    0.0019   18.0   0.0    86   124   860   899   829   922 0.82 Protein of unknown function, DUF258AAA_28               PF13521.2    163 augustus_masked-scaffold_7-processed-gene-0.607-mRNA-1 -           1486   0.00068   19.8   0.1   1   2     0.087        56    3.8   0.0     2    31   189   221   188   251 0.80 AAA domainAAA_28               PF13521.2    163 augustus_masked-scaffold_7-processed-gene-0.607-mRNA-1 -           1486   0.00068   19.8   0.1   2   2   0.00012     0.077   13.1   0.0     3    23   878   905   876   962 0.67 AAA domainAAA_22               PF13401.2    137 augustus_masked-scaffold_7-processed-gene-0.607-mRNA-1 -           1486   0.00069   19.8   0.1   1   2     0.034        22    5.2   0.0     6    32   187   213   184   244 0.88 AAA domainAAA_22               PF13401.2    137 augustus_masked-scaffold_7-processed-gene-0.607-mRNA-1 -           1486   0.00069   19.8   0.1   2   2   0.00037      0.24   11.5   0.0     6    51   875   934   871  1044 0.69 AAA domaincobW                 PF02492.15   177 augustus_masked-scaffold_7-processed-gene-0.607-mRNA-1 -           1486   0.00084   18.8   0.7   1   2     0.012       7.9    5.9   0.1     3    27   189   213   187   216 0.89 CobW/HypB/UreG, nucleotide-binding domaincobW                 PF02492.15   177 augustus_masked-scaffold_7-processed-gene-0.607-mRNA-1 -           1486   0.00084   18.8   0.7   2   2   0.00032      0.21   11.0   0.1     3    37   877   908   875   918 0.86 CobW/HypB/UreG, nucleotide-binding domainSMC_N                PF02463.15   220 augustus_masked-scaffold_7-processed-gene-0.607-mRNA-1 -           1486    0.0022   17.3   0.0   1   3       1.3   8.1e+02   -0.9   0.0   123   187   282   353   238   374 0.79 RecF/RecN/SMC N terminal domainSMC_N                PF02463.15   220 augustus_masked-scaffold_7-processed-gene-0.607-mRNA-1 -           1486    0.0022   17.3   0.0   2   3       0.2   1.3e+02    1.7   0.0    25    44   875   894   870   896 0.89 RecF/RecN/SMC N terminal domainSMC_N                PF02463.15   220 augustus_masked-scaffold_7-processed-gene-0.607-mRNA-1 -           1486    0.0022   17.3   0.0   3   3   0.00024      0.16   11.3   0.0   156   206  1002  1052   996  1065 0.85 RecF/RecN/SMC N terminal domainAAA_18               PF13238.2    129 augustus_masked-scaffold_7-processed-gene-0.607-mRNA-1 -           1486    0.0029   18.0   0.0   1   2      0.13        87    3.5   0.0     1    22   189   210   189   240 0.84 AAA domainAAA_18               PF13238.2    129 augustus_masked-scaffold_7-processed-gene-0.607-mRNA-1 -           1486    0.0029   18.0   0.0   2   2   0.00041      0.27   11.6   0.0     3    30   879   908   878  1007 0.76 AAA domainNACHT                PF05729.8    166 augustus_masked-scaffold_7-processed-gene-0.607-mRNA-1 -           1486    0.0043   16.8   0.7   1   2    0.0044       2.9    7.7   0.0     2    22   188   208   187   213 0.91 NACHT domainNACHT                PF05729.8    166 augustus_masked-scaffold_7-processed-gene-0.607-mRNA-1 -           1486    0.0043   16.8   0.7   2   2    0.0062       4.1    7.2   0.1     3    22   877   896   875   907 0.89 NACHT domainAAA_17               PF13207.2    122 augustus_masked-scaffold_7-processed-gene-0.607-mRNA-1 -           1486    0.0094   16.9   0.1   1   2       1.3   8.3e+02    1.0   0.0     2    24   189   212   188   251 0.75 AAA domainAAA_17               PF13207.2    122 augustus_masked-scaffold_7-processed-gene-0.607-mRNA-1 -           1486    0.0094   16.9   0.1   2   2   0.00047       0.3   12.1   0.0     4    34   879   911   877   994 0.63 AAA domainAAA_10               PF12846.3    304 augustus_masked-scaffold_7-processed-gene-0.607-mRNA-1 -           1486     0.046   13.2   0.2   1   2     0.003       1.9    7.9   0.0     4    29   189   214   186   389 0.82 AAA-like domainAAA_10               PF12846.3    304 augustus_masked-scaffold_7-processed-gene-0.607-mRNA-1 -           1486     0.046   13.2   0.2   2   2     0.092        60    3.0   0.1     4    21   877   894   874   900 0.84 AAA-like domainAAA_19               PF13245.2     76 augustus_masked-scaffold_7-processed-gene-0.607-mRNA-1 -           1486     0.051   13.4   2.9   1   2    0.0079       5.1    7.0   0.0    11    33   187   209   181   236 0.80 Part of AAA domainAAA_19               PF13245.2     76 augustus_masked-scaffold_7-processed-gene-0.607-mRNA-1 -           1486     0.051   13.4   2.9   2   2     0.023        15    5.5   0.2    11    33   875   896   870   911 0.85 Part of AAA domainAAA_24               PF13479.2    205 augustus_masked-scaffold_7-processed-gene-0.607-mRNA-1 -           1486      0.09   12.4   0.7   1   2    0.0098       6.4    6.4   0.0     5    23   188   206   186   210 0.89 AAA domainAAA_24               PF13479.2    205 augustus_masked-scaffold_7-processed-gene-0.607-mRNA-1 -           1486      0.09   12.4   0.7   2   2     0.055        36    3.9   0.1     7    23   878   894   876   902 0.83 AAA domainAAA                  PF00004.25   132 augustus_masked-scaffold_7-processed-gene-0.607-mRNA-1 -           1486      0.14   12.5   0.1   1   2     0.032        20    5.4   0.1     1    21   189   209   189   236 0.88 ATPase family associated with various cellular activities (AAA)AAA                  PF00004.25   132 augustus_masked-scaffold_7-processed-gene-0.607-mRNA-1 -           1486      0.14   12.5   0.1   2   2     0.072        47    4.3   0.0     3    24   879   900   877   931 0.87 ATPase family associated with various cellular activities (AAA)Arch_ATPase          PF01637.14   234 augustus_masked-scaffold_7-processed-gene-0.607-mRNA-1 -           1486      0.16   11.7   0.0   1   2     0.048        31    4.2   0.0    19    49   185   213   182   244 0.83 Archaeal ATPaseArch_ATPase          PF01637.14   234 augustus_masked-scaffold_7-processed-gene-0.607-mRNA-1 -           1486      0.16   11.7   0.0   2   2     0.029        19    4.9   0.0    20    56   874   911   864   978 0.75 Archaeal ATPaseTsaE                 PF02367.13   125 augustus_masked-scaffold_7-processed-gene-0.607-mRNA-1 -           1486      0.19   11.6   0.7   1   2      0.18   1.2e+02    2.6   0.1    19    42   186   209   166   218 0.79 Threonylcarbamoyl adenosine biosynthesis protein TsaETsaE                 PF02367.13   125 augustus_masked-scaffold_7-processed-gene-0.607-mRNA-1 -           1486      0.19   11.6   0.7   2   2    0.0092         6    6.8   0.1    18    42   871   897   857   908 0.76 Threonylcarbamoyl adenosine biosynthesis protein TsaEAAA_15               PF13175.2    405 augustus_masked-scaffold_7-processed-gene-0.607-mRNA-1 -           1486      0.24   10.7   0.0   1   2    0.0068       4.4    6.5   0.0    23    42   875   894   862   907 0.83 AAA ATPase domainAAA_15               PF13175.2    405 augustus_masked-scaffold_7-processed-gene-0.607-mRNA-1 -           1486      0.24   10.7   0.0   2   2      0.19   1.3e+02    1.7   0.0   362   402  1007  1046   993  1047 0.86 AAA ATPase domainCitrate_synt         PF00285.17   352 augustus_masked-scaffold_7-processed-gene-0.662-mRNA-1 -            473  4.6e-106  354.8   0.0   1   1  3.3e-110  5.4e-106  354.6   0.0     1   352    74   458    74   458 0.91 Citrate synthaseDUF946               PF06101.7    542 augustus_masked-scaffold_7-processed-gene-0.621-mRNA-1 -            503   1.6e-12   46.4   0.2   1   2     0.032   2.6e+02   -0.5   0.0   241   263    86   108    71   131 0.84 Plant protein of unknown function (DUF946)DUF946               PF06101.7    542 augustus_masked-scaffold_7-processed-gene-0.621-mRNA-1 -            503   1.6e-12   46.4   0.2   2   2     1e-15   8.1e-12   44.1   0.2   364   430   275   338   249   343 0.88 Plant protein of unknown function (DUF946)NPF                  PF16601.1    191 augustus_masked-scaffold_7-processed-gene-0.621-mRNA-1 -            503      0.33   11.4   1.4   1   1   6.8e-05      0.55   10.7   1.4   133   180   159   205   149   209 0.78 Rabosyn-5 repeating NPF sequence-motifE1-E2_ATPase         PF00122.16   221 snap_masked-scaffold_7-processed-gene-0.1086-mRNA-1 -            997   2.2e-63  213.4   7.4   1   2      0.49   1.1e+03   -1.5   0.0   142   170    52    80    43    88 0.77 E1-E2 ATPaseE1-E2_ATPase         PF00122.16   221 snap_masked-scaffold_7-processed-gene-0.1086-mRNA-1 -            997   2.2e-63  213.4   7.4   2   2   2.7e-66   6.3e-63  211.9   6.5     1   221    91   338    91   338 0.97 E1-E2 ATPaseCation_ATPase_C      PF00689.17   182 snap_masked-scaffold_7-processed-gene-0.1086-mRNA-1 -            997   2.2e-46  157.7   6.7   1   2       4.3     1e+04   -4.1   0.0    60    80   262   282   259   299 0.63 Cation transporting ATPase, C-terminusCation_ATPase_C      PF00689.17   182 snap_masked-scaffold_7-processed-gene-0.1086-mRNA-1 -            997   2.2e-46  157.7   6.7   2   2   9.4e-50   2.2e-46  157.7   6.7     1   182   774   977   774   977 0.93 Cation transporting ATPase, C-terminusHydrolase            PF00702.22   213 snap_masked-scaffold_7-processed-gene-0.1086-mRNA-1 -            997   2.2e-23   83.6   0.9   1   2       2.6   6.1e+03   -2.8   0.0    65    72   212   222   166   320 0.60 haloacid dehalogenase-like hydrolaseHydrolase            PF00702.22   213 snap_masked-scaffold_7-processed-gene-0.1086-mRNA-1 -            997   2.2e-23   83.6   0.9   2   2   2.1e-25   4.9e-22   79.2   0.4     3   213   345   705   343   705 0.68 haloacid dehalogenase-like hydrolaseHAD                  PF12710.3    217 snap_masked-scaffold_7-processed-gene-0.1086-mRNA-1 -            997   5.7e-20   72.2   0.0   1   1   7.7e-23   1.8e-19   70.6   0.0     1   217   346   702   346   702 0.55 haloacid dehalogenase-like hydrolaseCation_ATPase        PF13246.2     93 snap_masked-scaffold_7-processed-gene-0.1086-mRNA-1 -            997   4.5e-19   68.3   0.0   1   1   3.8e-22   8.9e-19   67.4   0.0     1    92   415   516   415   517 0.90 Cation transport ATPase (P-type)Cation_ATPase_N      PF00690.22    69 snap_masked-scaffold_7-processed-gene-0.1086-mRNA-1 -            997   5.4e-19   67.5   0.0   1   1   5.8e-22   1.3e-18   66.2   0.0     4    69     7    72     4    72 0.97 Cation transporter/ATPase, N-terminusHydrolase_3          PF08282.8    255 snap_masked-scaffold_7-processed-gene-0.1086-mRNA-1 -            997     3e-09   36.9   0.2   1   2    0.0029       6.7    6.2   0.0    22    53   599   630   592   634 0.89 haloacid dehalogenase-like hydrolaseHydrolase_3          PF08282.8    255 snap_masked-scaffold_7-processed-gene-0.1086-mRNA-1 -            997     3e-09   36.9   0.2   2   2   7.3e-10   1.7e-06   27.8   0.3   197   249   679   731   675   737 0.84 haloacid dehalogenase-like hydrolaseArfGap               PF01412.14   117 maker-scaffold_7-augustus-gene-0.1429-mRNA-1 -            590   8.9e-40  135.2   0.4   1   1   1.6e-43   1.3e-39  134.7   0.4     3   116    19   129    17   130 0.91 Putative GTPase activating protein for Arfpartial_CstF         PF15861.1     62 maker-scaffold_7-augustus-gene-0.1429-mRNA-1 -            590      0.18   11.5   0.1   1   1   3.8e-05      0.31   10.7   0.1    29    53    65    89    61    98 0.87 Partial cleavage stimulation factor domainzf-CCCH_2            PF14608.2     19 maker-scaffold_7-augustus-gene-0.1377-mRNA-1 -            519   4.1e-13   49.0  54.3   1   5   1.9e-05       0.1   13.0   5.8     2    18   331   346   331   346 0.99 RNA-binding, Nab2-type zinc fingerzf-CCCH_2            PF14608.2     19 maker-scaffold_7-augustus-gene-0.1377-mRNA-1 -            519   4.1e-13   49.0  54.3   2   5   0.00014      0.74   10.2   3.0     2    19   363   379   363   379 0.92 RNA-binding, Nab2-type zinc fingerzf-CCCH_2            PF14608.2     19 maker-scaffold_7-augustus-gene-0.1377-mRNA-1 -            519   4.1e-13   49.0  54.3   3   5   2.8e-10   1.5e-06   28.2  10.9     1    19   392   409   392   409 0.97 RNA-binding, Nab2-type zinc fingerzf-CCCH_2            PF14608.2     19 maker-scaffold_7-augustus-gene-0.1377-mRNA-1 -            519   4.1e-13   49.0  54.3   4   5   1.3e-07    0.0007   19.8   0.6     2    18   414   429   413   430 0.94 RNA-binding, Nab2-type zinc fingerzf-CCCH_2            PF14608.2     19 maker-scaffold_7-augustus-gene-0.1377-mRNA-1 -            519   4.1e-13   49.0  54.3   5   5      0.12   6.4e+02    0.9   4.0     2    18   434   448   433   448 0.83 RNA-binding, Nab2-type zinc fingerNab2                 PF11517.4    101 maker-scaffold_7-augustus-gene-0.1377-mRNA-1 -            519   0.00043   20.4   0.0   1   1   1.4e-07   0.00074   19.6   0.0    26    59    30    65     7    88 0.79 Nuclear abundant poly(A) RNA-bind protein 2 (Nab2)Disintegrin          PF00200.19    78 maker-scaffold_7-augustus-gene-0.1377-mRNA-1 -            519       4.4    7.9  21.3   1   2     0.064   3.4e+02    1.9  10.9    13    67   330   400   311   405 0.72 DisintegrinDisintegrin          PF00200.19    78 maker-scaffold_7-augustus-gene-0.1377-mRNA-1 -            519       4.4    7.9  21.3   2   2    0.0004       2.2    8.9   9.3    12    60   391   439   384   448 0.79 DisintegrinReo_sigmaC           PF04582.8    326 snap_masked-scaffold_7-processed-gene-0.1205-mRNA-1 -            689      0.35   10.1   5.4   1   1   0.00015      0.83    8.9   5.4    51   169   107   233    69   238 0.76 Reovirus sigma C capsid proteinDUF572               PF04502.9    330 snap_masked-scaffold_7-processed-gene-0.1205-mRNA-1 -            689       1.3    8.3  24.0   1   2   0.00013      0.72    9.2  21.8   114   254   100   253    35   315 0.60 Family of unknown function (DUF572)DUF572               PF04502.9    330 snap_masked-scaffold_7-processed-gene-0.1205-mRNA-1 -            689       1.3    8.3  24.0   2   2      0.43   2.3e+03   -2.3   0.0   189   216   397   420   381   537 0.44 Family of unknown function (DUF572)TAN                  PF11640.4    148 snap_masked-scaffold_7-processed-gene-0.1205-mRNA-1 -            689         2    8.5  11.3   1   2   2.5e-05      0.14   12.3   5.2    11    79   129   195   121   198 0.86 Telomere-length maintenance and DNA damage repairTAN                  PF11640.4    148 snap_masked-scaffold_7-processed-gene-0.1205-mRNA-1 -            689         2    8.5  11.3   2   2       2.2   1.2e+04   -3.7   0.0   126   135   408   417   392   427 0.40 Telomere-length maintenance and DNA damage repairSuf                  PF05843.10   296 maker-scaffold_7-augustus-gene-0.1371-mRNA-1 -            532   5.8e-06   26.4   1.5   1   2   9.9e-08   0.00054   19.9   0.1    41   133    81   173    37   220 0.89 Suppressor of forked protein (Suf)Suf                  PF05843.10   296 maker-scaffold_7-augustus-gene-0.1371-mRNA-1 -            532   5.8e-06   26.4   1.5   2   2    0.0028        15    5.3   0.2   108   136   280   308   232   339 0.83 Suppressor of forked protein (Suf)TPR_14               PF13428.2     44 maker-scaffold_7-augustus-gene-0.1371-mRNA-1 -            532     5e-05   23.6  10.6   1   7       2.1   1.2e+04   -2.5   0.0    20    30    56    66    48    74 0.77 Tetratricopeptide repeatTPR_14               PF13428.2     44 maker-scaffold_7-augustus-gene-0.1371-mRNA-1 -            532     5e-05   23.6  10.6   2   7     0.018        98    4.0   0.0     4    42    78   116    75   118 0.87 Tetratricopeptide repeatTPR_14               PF13428.2     44 maker-scaffold_7-augustus-gene-0.1371-mRNA-1 -            532     5e-05   23.6  10.6   3   7         3   1.6e+04   -3.4   0.1     4    24   150   170   149   175 0.69 Tetratricopeptide repeatTPR_14               PF13428.2     44 maker-scaffold_7-augustus-gene-0.1371-mRNA-1 -            532     5e-05   23.6  10.6   4   7   0.00013      0.68   10.7   0.1     4    43   282   321   280   322 0.97 Tetratricopeptide repeatTPR_14               PF13428.2     44 maker-scaffold_7-augustus-gene-0.1371-mRNA-1 -            532     5e-05   23.6  10.6   5   7    0.0057        31    5.6   0.0     2    42   352   392   351   394 0.89 Tetratricopeptide repeatTPR_14               PF13428.2     44 maker-scaffold_7-augustus-gene-0.1371-mRNA-1 -            532     5e-05   23.6  10.6   6   7     0.012        64    4.6   0.2     8    33   392   416   386   427 0.80 Tetratricopeptide repeatTPR_14               PF13428.2     44 maker-scaffold_7-augustus-gene-0.1371-mRNA-1 -            532     5e-05   23.6  10.6   7   7      0.24   1.3e+03    0.5   0.5     3    24   425   447   423   449 0.80 Tetratricopeptide repeatTPR_16               PF13432.2     65 maker-scaffold_7-augustus-gene-0.1371-mRNA-1 -            532      0.53   11.1   5.3   1   4      0.17   9.4e+02    0.7   0.1    21    41    61    85    53   103 0.69 Tetratricopeptide repeatTPR_16               PF13432.2     65 maker-scaffold_7-augustus-gene-0.1371-mRNA-1 -            532      0.53   11.1   5.3   2   4      0.62   3.3e+03   -1.0   0.2    13    44   288   326   287   342 0.63 Tetratricopeptide repeatTPR_16               PF13432.2     65 maker-scaffold_7-augustus-gene-0.1371-mRNA-1 -            532      0.53   11.1   5.3   3   4   0.00026       1.4    9.7   0.0    11    56   365   410   355   418 0.90 Tetratricopeptide repeatTPR_16               PF13432.2     65 maker-scaffold_7-augustus-gene-0.1371-mRNA-1 -            532      0.53   11.1   5.3   4   4    0.0054        29    5.5   0.8     3    54   391   447   389   449 0.73 Tetratricopeptide repeatGTP_EFTU             PF00009.23   193 maker-scaffold_7-augustus-gene-0.1401-mRNA-1 -            758   8.3e-42  142.8   0.0   1   1   5.4e-45   1.5e-41  142.0   0.0     1   187   299   517   299   552 0.89 Elongation factor Tu GTP binding domainGTP_EFTU_D3          PF03143.13   112 maker-scaffold_7-augustus-gene-0.1401-mRNA-1 -            758   2.7e-34  117.7   0.3   1   2   1.9e-24   5.2e-21   74.9   0.0     4    99   619   715   616   720 0.93 Elongation factor Tu C-terminal domainGTP_EFTU_D3          PF03143.13   112 maker-scaffold_7-augustus-gene-0.1401-mRNA-1 -            758   2.7e-34  117.7   0.3   2   2   1.1e-13   2.8e-10   40.3   0.1    78   111   721   754   715   755 0.93 Elongation factor Tu C-terminal domainGTP_EFTU_D2          PF03144.21    74 maker-scaffold_7-augustus-gene-0.1401-mRNA-1 -            758   2.2e-07   31.1   0.4   1   1     2e-10   5.5e-07   29.8   0.4     1    74   543   611   543   611 0.88 Elongation factor Tu domain 2MMR_HSR1             PF01926.19   110 maker-scaffold_7-augustus-gene-0.1401-mRNA-1 -            758    0.0034   17.3   0.9   1   2       4.3   1.2e+04   -3.7   0.1    50    58   190   197   170   214 0.56 50S ribosome-binding GTPaseMMR_HSR1             PF01926.19   110 maker-scaffold_7-augustus-gene-0.1401-mRNA-1 -            758    0.0034   17.3   0.9   2   2   9.6e-06     0.026   14.5   0.1     2   110   304   448   303   448 0.64 50S ribosome-binding GTPaseG-alpha              PF00503.16   377 maker-scaffold_7-augustus-gene-0.1401-mRNA-1 -            758      0.42    9.5   3.4   1   2       1.2   3.1e+03   -3.2   0.2    26    67   126   171    76   180 0.43 G-protein alpha subunitG-alpha              PF00503.16   377 maker-scaffold_7-augustus-gene-0.1401-mRNA-1 -            758      0.42    9.5   3.4   2   2   4.7e-05      0.13   11.2   0.3    21    71   257   317   227   337 0.60 G-protein alpha subunitMitofilin            PF09731.5    623 maker-scaffold_7-augustus-gene-0.1401-mRNA-1 -            758         4    6.0   9.0   1   1    0.0024       6.5    5.3   9.0    85   203   127   297    72   341 0.57 Mitochondrial inner membrane proteinCupin_8              PF13621.2    253 augustus_masked-scaffold_7-processed-gene-0.570-mRNA-1 -            357   3.6e-22   79.3   0.0   1   1   1.3e-25   5.3e-22   78.7   0.0    98   248   194   357   156   357 0.79 Cupin-like domainCupin_2              PF07883.7     71 augustus_masked-scaffold_7-processed-gene-0.570-mRNA-1 -            357     0.015   14.9   0.0   1   1   8.7e-06     0.035   13.7   0.0    38    63   320   345   307   350 0.84 Cupin domainCupin_4              PF08007.8    319 augustus_masked-scaffold_7-processed-gene-0.570-mRNA-1 -            357     0.044   13.1   0.0   1   1   1.7e-05     0.071   12.4   0.0   174   201   317   346   306   355 0.78 Cupin superfamily proteinJmjC                 PF02373.18   114 augustus_masked-scaffold_7-processed-gene-0.570-mRNA-1 -            357     0.054   13.8   0.0   1   2      0.87   3.5e+03   -1.7   0.0     4    18   234   248   232   258 0.82 JmjC domain, hydroxylaseJmjC                 PF02373.18   114 augustus_masked-scaffold_7-processed-gene-0.570-mRNA-1 -            357     0.054   13.8   0.0   2   2   5.3e-05      0.22   11.9   0.0    83   110   321   348   313   354 0.78 JmjC domain, hydroxylaseLactonase            PF10282.5    344 maker-scaffold_7-augustus-gene-0.1425-mRNA-1 -            382   2.5e-14   53.2   0.0   1   1   8.4e-18   1.4e-13   50.7   0.0     2   278     6   309     5   316 0.78 Lactonase, 7-bladed beta-propellerNop                  PF01798.14   230 augustus_masked-scaffold_7-processed-gene-0.535-mRNA-1 -            626   5.5e-67  225.5   0.0   1   1   8.9e-71   7.2e-67  225.1   0.0     2   230   161   402   160   402 0.91 snoRNA binding domain, fibrillarinPrp31_C              PF09785.5    120 augustus_masked-scaffold_7-processed-gene-0.535-mRNA-1 -            626   2.3e-43  147.8   1.6   1   2      0.77   6.3e+03   -2.0   0.0    50    94    47    92    43   113 0.59 Prp31 C terminal domainPrp31_C              PF09785.5    120 augustus_masked-scaffold_7-processed-gene-0.535-mRNA-1 -            626   2.3e-43  147.8   1.6   2   2   6.4e-47   5.2e-43  146.7   0.3     1   120   409   561   409   561 0.84 Prp31 C terminal domainAIRC                 PF00731.16   148 maker-scaffold_7-augustus-gene-0.1381-mRNA-1 -            616   8.9e-60  200.4   2.4   1   2       1.5   2.3e+03   -2.1   0.0     5    30    49    74    49    81 0.88 AIR carboxylaseAIRC                 PF00731.16   148 maker-scaffold_7-augustus-gene-0.1381-mRNA-1 -            616   8.9e-60  200.4   2.4   2   2   7.6e-63   1.1e-59  200
[truncated: 1,200,000 more chars]
